# Discovery of Compound A – a selective activator of the glucocorticoid receptor with anti-inflammatory and anti-cancer activity

**DOI:** 10.18632/oncotarget.5078

**Published:** 2015-10-02

**Authors:** Ekaterina Lesovaya, Alexander Yemelyanov, Amanda C. Swart, Pieter Swart, Guy Haegeman, Irina Budunova

**Affiliations:** ^1^ Department of Chemical Carcinogenesis, N.N. Blokhin Russian Cancer Research Center, Moscow, Russia; ^2^ Pulmonary Division, Feinberg School of Medicine, Northwestern University, Chicago, IL, USA; ^3^ Department of Biochemistry, Stellenbosch University, Stellenbosch, South Africa; ^4^ LEGEST, University of Ghent, Ghent, Belgium; ^5^ Department of Dermatology, Feinberg School of Medicine, Northwestern University, Chicago, IL, USA

**Keywords:** Review, Compound A, selective glucocorticoid receptor activator (SEGRA), inflammation, cancer, autoimmune diseases

## Abstract

Glucocorticoids are among the most effective anti-inflammatory drugs, and are widely used for cancer therapy. Unfortunately, chronic treatment with glucocorticoids results in multiple side effects. Thus, there was an intensive search for selective glucocorticoid receptor (GR) activators (SEGRA), which retain therapeutic potential of glucocorticoids, but with fewer adverse effects. GR regulates gene expression by transactivation (TA), by binding as homodimer to gene promoters, or transrepression (TR), via diverse mechanisms including negative interaction between monomeric GR and other transcription factors. It is well accepted that metabolic and atrophogenic effects of glucocorticoids are mediated by GR TA. Here we summarized the results of extensive international collaboration that led to discovery and characterization of Compound A (CpdA), a unique SEGRA with a proven “dissociating” GR ligand profile, preventing GR dimerization and shifting GR activity towards TR both *in vitro* and *in vivo*. We outlined here the unusual story of compound's discovery, and presented a comprehensive overview of CpdA ligand properties, its anti-inflammatory effects in numerous animal models of inflammation and autoimmune diseases, as well as its anti-cancer effects. Finally, we presented mechanistic analysis of CpdA and glucocorticoid effects in skin, muscle, bone, and regulation of glucose and fat metabolism to explain decreased CpdA side effects compared to glucocorticoids. Overall, the results obtained by our and other laboratories underline translational potential of CpdA and its derivatives for treatment of inflammation, autoimmune diseases and cancer.

## INTRODUCTION

Synthetic glucocorticoids (Gcs) are the most frequently prescribed anti-inflammatory drugs, and have been used worldwide in the treatment of asthma, rheumatoid arthritis, inflammatory bowel disease, psoriasis, and other inflammatory and autoimmune diseases since early 1960s [1–3]. They are also extensively used as immunosupressants in the clinical management of organ transplantation, and as anti-cancer drugs, especially for the treatment of patients with hematological malignancies (leukemia, lymphoma, multiple myeloma).

Unfortunately chronic treatment with Gcs is associated with multiple adverse effects, including altered glucose metabolism, steroid-induced diabetes, osteoporosis, skin and muscle atrophy, alteration of behavior and mood. These clinical conditions motivated the active development of selective GR activators (SEGRA), novel glucocorticoid receptor (GR) ligands that preserve the therapeutic activity of Gcs, with fewer adverse effects. Many candidate SEGRA molecules have been synthesized by industry and academia or identified by chemical library screening, with some, such as Mapracorat and PF-04171327, having reached clinical trials [4–10].

**Figure 1:**
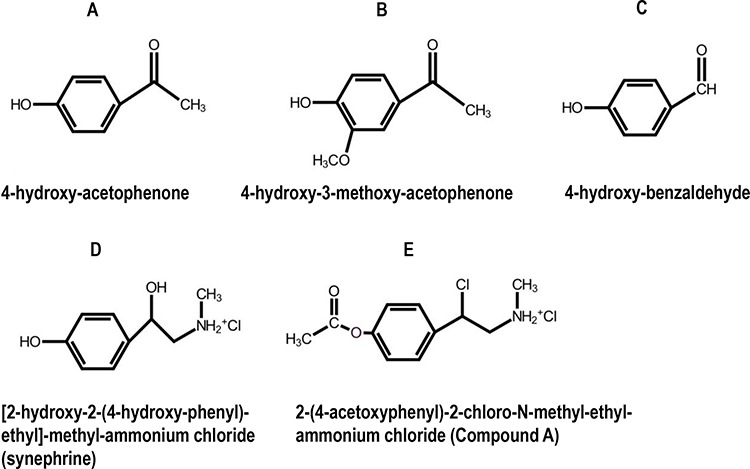
Chemical structures of selected phenolic compounds from S. tuberculatiformis, and their synthetic analog, Compound A **A–C.** Chemical structures of selected bio-active phenolic compounds isolated from S. tuberculatiformis Botch extracts, **D.** an inactive compound, [2-(hydroxy)-2-(4-hydroxyphenyl)-ethyl]-methyl ammonium chloride (synephrine), and **E.** a synthetic analog 2-(4-acetoxyphenyl)-2-chloro-N-ethyl ammonium chloride (Compound A). See also [111] for additional details.

This review is focused on Compound A (CpdA), a non-steroidal SEGRA, which is a stable synthetic analogue of a natural compound found in *Salsola tuberculatiformis* Botschantzev (Fig. 1). We discuss the unusual history of CpdA discovery, its GR ligand properties, anti-inflammatory and anti-cancer effects along with decreased metabolic and atrophogenic side effects, as compared to classical Gcs.

## DISCOVERY OF COMPOUND A

Plants belonging to the genus Salsola (Family: Chenopodiaceae) are found in the arid and semi-arid regions of our planet with more than 60 different Salsola species found in Namibia and in the Republic of South Africa [11]. This genus is first mentioned in San folklore as traditional medicinal plants with Salsola aqueous extracts being used as oral contraceptives by San women [12]. During the investigation of the unusual biological effects of Salsola, it was confirmed that at least one member of this genus indeed contains active contraceptive compound(s). The chemical nature of these natural products has however, baffled scientists over many years.

In 1961 de Lange described a rare syndrome of prolonged gestation which sporadically occurred among Karakul sheep in the Keetmanshoop district in Namibia [13]. *Salsola tuberculata*, later renamed Salsola tuberculatiformis Botschantzev was subsequently identified to be responsible for this syndrome [14] [15]. This shrub is also known to local inhabitants as “gannabos”. In our investigations, using various derivatizing reagents to isolate the labile compounds in the form of their stabilized derivatives, we showed that treatment with trimethylammonium acetyl hydrazide chloride (Girard-T reagent), used for the isolation of aldehydes and ketones [16], removed all the contraceptive activity from active plant extracts [17]. The derivatives formed in this manner were isolated by solvent partitioning and then decomposed under acidic conditions to yield three compounds in pure form (Fig. 1A–1C). These compounds were identified by proton NMR and EI-mass spectrometry as 4-hydroxy-acetophenone (Fig. 1A), 4-hydroxy-3-methoxy- acetophenone (Fig. 1B) and 4-hydroxy-benzaldehyde (Fig. 1C) [17]. Although synthetic samples of both ketones did show some biological activity in rats, neither A or B, nor a mixture of A+B, could, at the levels found in the extract, account for the contraceptive activity of the plant. The aldehyde (Fig. 1C) was inactive. In later experiments without derivatizing reagents, we were able to isolate another pure compound which consistently appeared in active fractions. The compound was identified by NMR, UV and EI-mass spectrometry as 1-(4- hydroxyphenyl)-2-methyl-aminoethanol or synephrine (Fig. 1D). This structure was subsequently confirmed by spectroscopic comparison with synthetic compounds as well as by the preparation of the same acylated derivatives from natural and synthetic material. Synthetic synephrine did not, however, show biological activity similar to that of the active plant extract.

These data suggested that compounds identified in active Salsola extracts probably arose as breakdown products from one or more, yet unknown, active precursor(s), pointing to the presence of phenolic compound(s) that contained a labile functional group. The labile group apparently decomposed under appropriate conditions to aldehydes, ketones or alcohols. Our results further suggested that the long duration of the biological tests for contraceptive activity was not compatible with the testing of the active compound(s), necessitating a more rapid and sensitive screening method for the evaluation of plant extracts or fractions. We developed a micro-assay based on the interference of the test compound with the substrate-induced difference spectrum of sheep adrenal cytochrome P450-dependent steroid 11β-hydroxylase (CYP11B1), the enzyme catalyzing the biosynthesis of cortisol and corticosterone from deoxycortisol and deoxycorticosterone, respectively [18–21]. We isolated a single compound to homogeneity using this assay and named it S2 [21]. Structure determination by NMR and mass spectrometry indicated the presence of an unstable aziridine, after which we subsequently synthesized a more stable aziridine precursor, Compound A (CpdA, Fig. 1E).

We showed that CpdA, similar to extracts prepared from dried plant material, caused contraception in rats [20] and interacted with two glucocorticoid-binding proteins: CYP11B1 and corticosteroid-binding globulin in plasma (CBG), while also being able to displace Gcs from CBG [22]. The fact that CpdA interacted with Gcs-binding proteins, suggested that it could also bind to GR, a well known transcription factor (TF) that mediates the biological effects of glucocorticoid hormones [23–25].

## COMPOUND A ACTS AS A SELECTIVE GR ACTIVATOR (SEGRA) AND AN ANTAGONIST OF THE ANDROGEN RECEPTOR

The GR belongs to a superfamily of nuclear hormone receptors and shares a characteristic three-domain structure: a variable N-terminal region with transactivation domain responsible for gene activation, a conserved central DNA-binding domain with dimerization and nuclear localization sequences, and a conserved C-terminal ligand-binding domain [26–29].

In the absence of Gcs, the GR resides in cytoplasm bound to chaperones: heat shock proteins and immunophillins that control GR nuclear import [30, 31]. Upon Gcs binding, the GR translocates to the nucleus, where it regulates gene expression via (i) transactivation (TA), that requires GR-homodimer binding to palindromic glucocorticoid-responsive elements (GRE), and (ii) transrepression (TR), mediated via different mechanisms including negative interaction between GR and other TFs, such as NF-κB, AP-1, p53, STATs, IRF-3, CREB [12, 26–28, 32–34]. This interaction may occur in the nucleus or, as we have reported, in the cytoplasm [35], and does not require GR dimerization, as it could be mediated by the monomeric GR [26–28, 33, 36].

We and others reported that CpdA acts as a GR ligand. It binds to the GR with high affinity, with IC_50_ in the nM range, as determined by competitive ligand–binding assays in human and rodent cells expressing endogenous GR [35–37]. The effects of CpdA on major steps of GR activation: phosphorylation, dimerization, nuclear translocation and GR-DNA binding have been extensively studied. We showed, in fibroblasts, epithelial and lymphoid cells with endogenous or exogenous GR, that CpdA induced modest GR nuclear translocation [35, 37, 39]. Using different approaches including fluorescence resonance energy transfer (FRET), we and others discovered that CpdA, unlike Gcs, did not elicit ligand-induced GR dimerization [35–38]. Furthermore, CpdA did not induce GR phosphorylation at Ser211, critical for GR TA activity [37]. Consequently, CpdA did not significantly affect or even inhibited constitutive and Gcs-induced GR DNA binding and activation of endogenous genes and GRE. Luciferase reporters [35–37, 39]. In contrast, CpdA and classical Gcs have remarkably similar TR profiles, suppressing the activity of many pro-proliferative and anti-apoptotic TFs. Depending on cell context, CpdA has been shown to inhibit NF-κB, AP-1, Ets-1, Elk-1, SRF, NFATc [35, 37, 39]. Using cells with GR knockdown or lacking GR, we proved that CpdA inhibitory effects on TFs are GR-dependent [35, 37, 39]. Interestingly, in contrast to Gcs that usually do not interfere with TF nuclear translocation, CpdA significantly decreased/delayed nuclear import of NF-κB and AP-1 proteins [35, 40].

**Figure 2:**
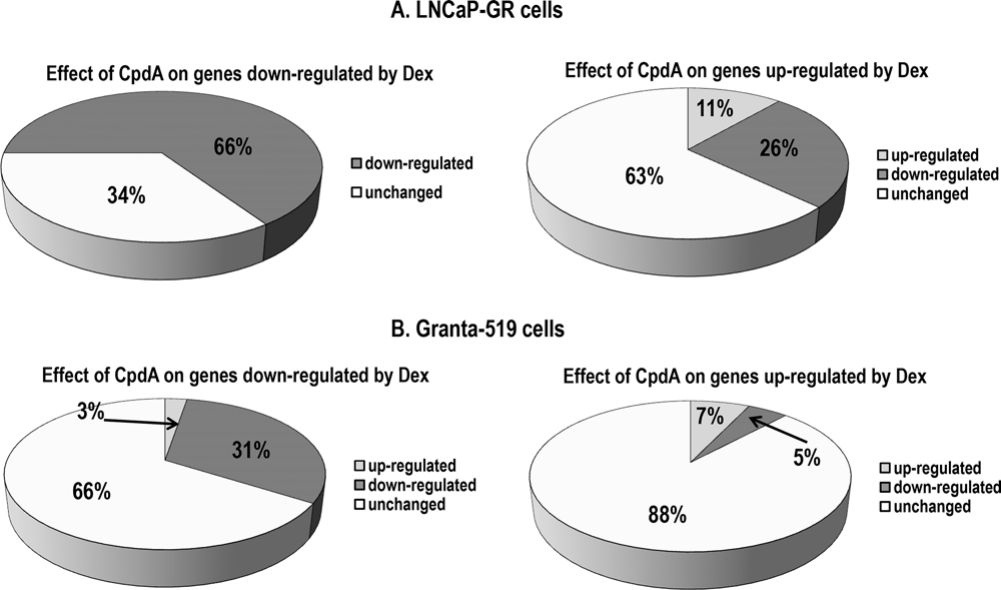
Global CpdA effect on the expression of glucocorticoid-responsive genes in epithelial and lymphoid cells **A.** LNCaP-GR cells were treated with vehicle (EtOH), glucocorticoid Dexamethasone (Dex, 1 uM) or CpdA (5 uM) for 8 h; **B.** Granta-519 cells expressing endogenous GR, were treated with vehicle, Dex (1 uM) or CpdA (1 uM) for 16 h. Total RNA was extracted, and global gene expression changes were evaluated by Illumina DNA arrays (three individual RNA samples/group). Expression changes with fold change > 1.5, and *p*-value < 0.05 were considered biologically and statistically significant. Effect of CpdA on Dex-regulated genes: down-regulation (black); up-regulation (grey), no change (white).

The global effect of CpdA and the glucocorticoids on gene expression was compared by DNA array analyses in A549 lung carcinoma and Granta-519 lymphoma cells with endogenous GR; and in prostate cancer LNCaP-GR cells stably expressing exogenous GR. In both, epithelial and lymphoid cells, molecular TA signatures of CpdA and the synthetic glucocorticoid, dexamethasone (Dex), were different. In fact, CpdA was able to activate only 7–11% of the genes induced by Dex in LNCaP-GR and Granta-519 cells (Fig. 2). However, their effects on the GR TR branch were much more similar − CpdA was able to inhibit the expression of 31% and 66% of genes negatively regulated by Dex in lymphoma and prostate cancer cells, respectively (Fig. 2), and suppressed 32% of the genes negatively regulated by Dex in A549 lung cells [41]. In summary, our validated microarray results (Supplementary Fig. 1, Supplementary Tables S1 and S2; NCBI arrays with access numbers GSE71102 (LNCaP-GR) and GSE71099 (Granta-519) confirmed at global level the unique CpdA capability to dissociate GR TA and TR functions, and to shift GR function towards TR.

In addition to its function as a SEGRA, CpdA acts as an antagonist of the androgen receptor (AR). Even though CpdA has a lower affinity for the AR compared to active androgens, CpdA induced considerable AR nuclear translocation [35, 42]. We and others showed that CpdA repressed the interaction between the NH(2)- and COOH-terminal domains of the AR critical for its function, and inhibited both constitutive and dihydrotestosterone (DHT)-induced AR-DNA binding and AR transcription activity in Luciferase reporter assays [35, 42]. These results suggest that the interaction of CpdA with the AR closely resembles the effects of well characterized anti-androgens, such as Casodex. CpdA did not significantly affect the activity of other steroid hormone receptors such as mineralcorticoid, progesterone, and estrogen receptors [36, 37].

It is important to note that CpdA is also characterized by its own molecular signature component, and regulated a subset of genes that were not affected by Gcs ([41], GEO submissions GSE71102 and GSE71099). Whether these genes were regulated via GR/AR remains to be investigated. Nevertheless, the therapeutic activity of CpdA was mostly mediated via GR or AR steroid receptors as discussed below.

## THE BIOLOGICAL ACTIVITY OF CPDA- POTENTIAL CLINICAL BENEFITS

Our findings have contributed to the understanding of the significance of GR TR mechanisms in the anti-inflammatory and anti-cancer effects of Gcs [43–46]. In contrast, GR TA is proposed to underlie many adverse metabolic effects of Gcs [46–50]. Even though some genes activated by Gcs (such as *DUSP1/MKP1, GILZ, IκBa*) are involved in anti-inflammatory responses, while other genes inhibited by Gcs/GR (adrenocorticotropic hormone (*ACTH*) or collagens) are involved in steroidal side effects, it is now well established in the field that GR ligands, which do not support GR dimerization and predominantly activate the TR branch of GR signaling, should have a better therapeutic index than classical Gcs [36, 50–52]. Indeed, it is believed that GR ligands that “dissociate” GR TR from GR TA will retain the therapeutic activity of classical steroids, but will have reduced adverse side effects regarding bone, glucose and lipid metabolism. Thus, based on its GR ligand profile, CpdA was expected to exert pronounced anti-inflammatory and anti-cancer effects, while inducing fewer adverse effects typical for the classical synthetic Gcs that often limit their therapeutic applications. As discussed below, we had also anticipated that, compared to Gcs, CpdA would be less likely to induce patient resistance to GR-targeted therapy [39, 106].

### Anti-inflammatory effects of compound A

Gcs remain a gold standard as anti-inflammatory drugs. They are effective against a wide range of inflammatory and autoimmune diseases characterized by the different types of CD4+ T helper (Th) lymphocytes. Th1 cells producing interferon (IFN)-α, interleukin (IL)-2, transcription factor T-bet, and tumor necrosis factor (TNF), as well as Th17 cells producing IL-17, are all implicated in the pathogenesis of autoimmune disorders including multiple sclerosis, rheumatoid arthritis, diabetes mellitus type 1; inflammatory bowel disease (Crohn's disease) and acute organ rejection [53–61]. Th2 cells that produce IL-4, IL-5, IL-6, IL-9, IL-10, and IL-13 are mostly responsible for atopic disorders (such as atopic dermatitis, rhinitis, asthma), and allergies [54–60]. It is well known that Gcs can block both Th1/Th17 and Th2 inflammatory responses as well as inflammatory factors, such as NF-κB, central for the production of cytokines and chemokines involved in the activation and differentiation of Th cells.

The therapeutic activity of CpdA was extensively tested in comparison with Gcs by us and other research groups in various Th1/Th17 and Th2 *in vivo* models of inflammation (Table 1). In the Th1/Th17 models, which included zymozan paw inflammation, collagen-induced arthritis (CIA), experimental autoimmune neuritis (EAN) and encephalomyelitis (EAE), type 1 diabetes and acute colitis, CpdA inhibited the development of clinical symptoms and morphological manifestations of disease, such as paw swelling, inflammation in joints, colon, pancreas, central nerve system and peripheral nerves [37, 38, 62–66]. CpdA also reduced neuronal damage and demyelination, as well as progression of neuropathic pain [65]. Furthermore, in the predominantly Th2-driven mouse asthma model, CpdA reduced inflammation and airway hyper-responsiveness [66]. In all the aforementioned models, CpdA decreased both the severity of localized clinical symptoms and the systemic signs of inflammation. However, CpdA doses in most studies were ~10-fold higher compared to Gcs, possibly due to a lower GR affinity and the non-steroidal nature of the compound [26, 33, 37].

**Table 1:** Anti-inflammatory effects of CpdA

Model description	Therapeutic effects of CpdA	Possible molecular mechanisms	References
***In vivo* models of inflammation**			
Zymozan-induced inflamed paw	Decreased swelling	Decreased NF-κB activity and inhibited expression of pro-inflammatory cytokines: *IL-8, IL-6*, and *E-selectin*	[37]
Collagen-induced arthritis	Decreased severity of disease; Strong anti-inflammatory response	Inhibited TNF-α-induced production of pro-inflammatory cytokines: IL-1β, IL-6	[70], [38] [62]
Experimental autoimmune encephalomyelitis	Ameliorated clinical symptoms and disease severity; Reduced leukocyte infiltration in the spinal cord; Reduced neuronal damage and demyelination	Impaired NF-κB activation; Inhibited pro-inflammatory cytokines: INF-α, IL-1β, TNF-α, IL-23, IL-17	[63]; [64]
Experimental autoimmune neuritis	Inhibited paraparesis; Decreased infiltration of sciatic nerves with lymphocytes and macrophages and the progression of neuropathic pain; Decreased demyelination; Modulated immune response: switch from Th1/Th17 towards anti-inflammatory T regulatory (Foxp3+/CD4+ Treg) response	Inhibited Th1/Th17 cytokines in microglia: *IL1β, IL-17, IL-12p35, IFN-γ, TNF-α*, and *iNOS*; Increased Th2 cytokine and *Foxp3* expression.	[65]
Acute trinitrobenzene sulfonic acid-induced colitis	Ameliorated acute colitis; Inhibited inflammatory cell infiltration into colon wall	Inhibited expression of pro-inflammatory genes: TNF-α, IL-1β, and COX-2	[69]
Streptozotocin model of type 1 diabetes	Protected against development of diabetes; Modulated peripheral immune response (switch from Th1/Th17 towards anti-inflammatory T regulatory/Treg response)	Inhibited pro-inflammatory cytokines: IL-1β, TNF-α, IL-6; Up-regulated expression of anti-inflammatory cytokines: IL-4 and IL-10	[73]
Ovalbumin-induced Th2-driven asthma model	Reduced inflammatory cell infiltration in lungs, cytokine production, mucus and Ig production; Reduced development of airway hyperresponsiveness (AHR)	Inhibited NF-κB activity and nuclear translocation; Inhibited STAT6 activity and nuclear translocation; Inhibited expression of Th2 cytokines: *IL-4, IL-5*, and *IL-13*	[66]
*mdx* (Dystrophin-deficient) mice, an animal model for Duchene muscular dystrophy	Reduced muscle inflammation; Improved strength and function of the limbs	Inhibited NF-κB signaling in muscle; Inhibited expression of IL-6, CCL2, IFN-γ, TNF-α, IL- 12p70	[67]
***In vitro* models of inflammation**			
Synovial fibroblasts from patients with rheumatoid arthritis		Inhibited NF-κB activity and IKK phosphorylation; Induced IκB-α accumulation; Inhibited *IL-1β* expression	[106]; [38] [70]
Co-cultivation of BMSC (bone marrow stem cells) with osteoclasts		Inhibited *IL-1β, TNF-α, IL-6* expression	[105]
Dengue virus (DENV) infection of HepG2 transformed hepatocyte cells	Reduced DENV production	Reduced the expression of DENV-induced cytokines: CXCL10 and TNF-α; Decreased leukocyte migration	[74]
Murine T-lymphocytes		Inhibited T-bet (T box expressed in T-cells) factor central for Th1 response; Decreased GR-dependent transrepression	[61]
Primary microglial cells		Inhibited NF-κB activation; Inhibited *TNF-α* and *IL-1β* cytokines	[63]
Immortalized murine macrophage cell line RAW 264.7		Attenuated expression of *TNF-α, iNOS*, and *IL-1*, but increased expression of anti-inflammatory *IL-10;* Induced macrophage differentiation towards M2 anti-inflammatory phenotype	[65]

CpdA has also been shown to ameliorate muscle pathology in *mdx* mice, an animal model for Duchene muscular dystrophy associated with abnormal muscle inflammation and activation of NF-κB. CpdA treatment for a period of 2 months, normalized limb strength and function and attenuated cathepsin-B enzyme activity (a surrogate marker for inflammation) [67].

#### Mechanisms of the anti-inflammatory effects of CpdA

At molecular level, the anti-inflammatory effects of CpdA were mediated mostly through blockage of inflammatory TFs including NF-κB, T-bet, STAT6, the latter being central to the Th2-driven asthma model [61, 66, 68], which resulted in strong inhibition of expression of numerous pro-inflammatory cytokines and chemokines characteristic for each specific model. For example, in the zymosan-induced paw inflammation model, the anti-inflammatory effect of CpdA was associated with the down-regulation of TNF-α-induced- and NF-κB-dependent pro-inflammatory genes such as *IL-6, E-selectine, IL-8* and others [37]. In the model of experimental colitis induced by trinitrobenzene sulfonic acid, CpdA reduced the production of colonic *TNF-α, IL-1*, and *COX-2* at mRNA level [69].

In models of autoimmune diseases such as arthritis, experimental autoimmune encephalomyelitis (EAE), autoimmune neuritis (EAN), and asthma, CpdA inhibited the expression of both Th1/Th17 and Th2 cytokines [38, 62–66, 70–72]. In mice with EAE, for instance, CpdA was able to penetrate the blood-brain barrier and attenuated EAE symptoms via the inhibition of NF-κB nuclear translocation and the expression of NF-κB-driven pro-inflammatory cytokines (*INF-γ; IL-1β; TNF-α; IL-23; IL-17* and others) in the central nerve system [63]. In the Th2 asthma model, CpdA's effects were mediated by the inhibition of TNF-α and Th2 inflammatory cytokines (*IL-4, IL-5, IL-6*) as well as blockage of NF-κB activity in lung [66]. In the model of Duchene muscular dystrophy, CpdA reduced expression of *IL-6, CCL2, IFNγ, TNF-α*, and *IL-12p70* cytokines in muscle [67]. Interestingly, in models of inflammatory type 1 diabetes and autoimmune neuritis, CpdA, in addition to its inhibitory effect on Th1/Th17 cytokines, was able to switch Th1/Th17 differentiation towards anti-inflammatory T regulatory (Treg) cell differentiation, an important mechanism for the resolving of inflammation [65, 73].

The molecular mechanisms of CpdA effects on NF-κB activation and the expression of inflammatory cytokines have been further corroborated *in vitro*, in cell lines and in primary cell cultures including mouse microglia and astrocytes, as well as synoviocytes from patients with rheumatoid arthritis [63, 70]. In most experiments, cells were activated by TNF-α or LPS to induce cytokine expression. Alternatively, HepG2 human liver cells were infected with Dengue virus to induce inflammatory cytokines and to model the “cytokine storm” that underlies the pathogenesis of Dengue hemorrhagic fever [74]. The aforementioned experiments demonstrated that CpdA inhibited NF-κB and the expression of pro-inflammatory cytokines in a GR-dependent fashion, and confirmed that CpdA acts as “dissociated” GR ligand, as it was incapable of activating GR target genes, such as *DUSP1/MKP1, GILZ, FKBP51* [38, 62, 65, 69, 70]. Interestingly, *in vitro* experiments confirmed that CpdA, in contrast to Gcs, can do both: inhibit expression of inflammatory cytokines and induce the expression of anti-inflammatory cytokines such as IL-10 [65].

### Anti-cancer effects of Compound A as a mono-therapy and in combination with proteasome inhibitors

Gcs usually promote cell differentiation and inhibit proliferation, and have proven anti-cancer activity [44, 45, 75, 76]. Synthetic Gcs have been extensively used clinically against leukemia, lymphomas, and multiple myeloma. The steroids are typically paired with traditional chemotherapies (cyclophosphamide, doxorubicin, vincristine, methotrexate), and newer (proteasome inhibitors) agents [77–80]. Gcs are also used in combination therapy of patients with epithelial cancers, mostly for palliative treatment of metastasis-related pain, decreasing nausea and other side effects of chemotherapy [81]. However, in some cases they have been used as anti-cancer drugs, and even as a mono-therapy, as in the case of elderly patients with prostate cancer [82].

**Figure 3:**
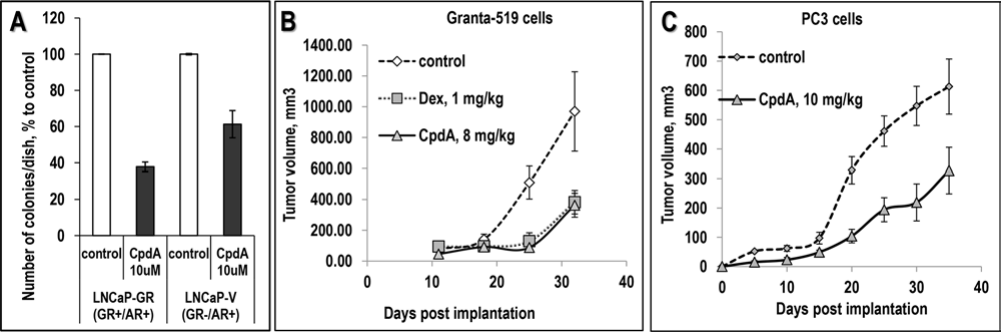
Anti-cancer effect of CpdA in colony forming assay and in xenograft models **A.** Inhibitory effect of CpdA on anchorage-independent cell growth. LNCaP-GR and LNCaP-V (empty virus) -infected cells on 1% soft agar were treated with vehicle or 5 uM CpdA as described ([35], Supplementary Table 3). **B, C.** Effect of CpdA on prostate cancer cells (PC3) and lymphoma cells (Granta-519) xenograft growth *in vivo*. Granta-519 cells (B, 10 million cells/site in BD Matrigel Matrix) and PC3 cells (C, 1.2 million cells/site) were injected s.c. into athymic nu/nu female mice. Mice with established tumors (>50 mm^3^) were treated by i.p. injections 3 times/wk for 35 days with: CpdA (solid line), Dex (dotted line) or vehicle (0.1% Etoh in saline, dashed line).

In contrast to anti-inflammatory effects, the anti-cancer effects of CpdA were uncovered more recently. Our findings showed that CpdA inhibited growth and viability of numerous cancer cells including prostate cancer, lymphoma, leukemia and multiple myeloma as well as melanoma cell lines (Table 2). The anti-cancer effect of CpdA was GR-dependent, and in cancer cells with GR knockdown, CpdA was significantly less toxic (Table 2) [35, 39, 83]. In prostate cancer cell models, the cytotoxic concentrations of CpdA were in the range of 10^−5^ to 10^−6^ M, in standard growth and apoptosis tests inmonolayer cultures as well as in colony-forming assays (Fig. 3A and [35, 83]). Moreover, using prostate cancer cells with different AR and GR status, we demonstrated clearly that CpdA could exert its cytostatic effect via both receptors which correlated with CpdA's dual GR/AR ligand properties shown in our earlier studies [35].

**Table 2:** Cytotoxic effect of CpdA in cancer cells *in vitro*

Cell line	Origin	Effect	CpdA concentration	References
RWPE-1	Non-transformed prostate cells	+/−	10 uM	[35]
LNCaP-GR	Prostate Cancer (PC)	++	10 uM	[35, 83]
PC3	PC	+++	10 uM	[35]
DU145	PC	+++	10 uM	[35]
Hematopoietic SCs		−	10 uM - 50 uM	Budunova et al., unpublished
Granta-519	Mantle cell lymphoma (MCL)	++	1 uM	[39]
NCEB-1	MCL	+++	1 uM	[39]
MM-1.S	Multiple myeloma	+++	1 uM	[39]
CEM	T cell leukemia	++	3 uM	[39]
A375	Melanoma	++	10 uM	Yemelyanov and Budunova, unpublished
C8161	Melanoma	++	10 uM	Yemelyanov and Budunova, unpublished

Cancer cells and non-transformed cells of the same origin were treated with CpdA or vehicle, and the cytotoxic effect was evaluated by cell counting as described [35, 39, 83]. The cytotoxic effect is depicted + (25%)/++ (30–50%)/+++ (>50%) cell number reduction in CpdA versus vehicle control group.

The leukemia and lymphoma cells appeared to be far more sensitive to CpdA than epithelial cancer cells. CpdA was equally or more potent than Gcs in the inhibition of growth and survival of transformed lymphoid cell lines, as well as in primary cell cultures from acute T cell leukemia patients [39].

We subsequently used human cancer cells xenografts to assess the anti-cancer activity of CpdA *in vivo* (Figs. 3B and 3C), and demonstrated that CpdA inhibited growth of PC3 prostate cancer cell xenografts by 40% and Granta-519 mantle cell lymphoma xenografts by 70% when used at doses ~ 8–10 μg/animal, which were lower than the anti-inflammatory doses of CpdA [37, 63].

As mentioned above, Gcs are mostly used in combination anti-cancer therapies, frequently with proteasome inhibitors such as Bortezomib (BZ) - a proteasome inhibitor approved by FDA in 2003 for the treatment of hematological malignancies [84, 85]. While the biological effects of GR ligands depend on GR levels, proteasome inhibitors are known to stabilize the GR and to prevent Gcs-induced GR degradation [86, 87]. We showed that BZ indeed induced GR accumulation in lymphoma, leukemia and prostate cancer cells, and enhanced CpdA's profile as a dissociated GR ligand further shifting GR signaling to TR [35, 39, 83]. Moreover, we showed that BZ sensitized lymphoma and prostate cancer cells to CpdA, and we revealed a pronounced GR-dependent cooperative CpdA and BZ anti-cancer effect [35, 39, 83]. Similar results were obtained in multiple myeloma cells MM.1S expressing endogenous functional GR, in which the anti-cancer effects of CpdA and BZ were also synergistic [39].

#### The mechanisms of anti-cancer effect of CpdA

There is growing evidence that, in parallel with inhibition of growth and survival, Gcs launch an anti-apoptotic program and, under certain conditions, can even protect cancer cells from chemotherapy-induced apoptosis [81, 88–91]. We and others have reported that Gcs can activate inhibitors of apoptosis IAPs, MAP kinase phosphatase DUSP1/MKP1 (resulting in AP1 inhibition), pro-survival serum and glucocorticoid-inducible protein kinase SGK1, as well as increasing the expression of anti-apoptotic genes from the Bcl-2 family (*Bcl-2* and *Bcl-xl*) [83, 88–90, 92]. In addition, we showed that Gcs prevent BZ-induced endoplasmic reticulum (ER) stress in prostate cancer cells [83].

CpdA on the other hand, in contrast to Gcs did not induce an anti-apoptotic self-defense program in cancer cells. Moreover, CpdA enhanced lethal BZ-induced ER stress [83]. Our findings highlighted the potential benefits of combining a SEGRA such as CpdA with proteasome inhibitors to trigger the maximal potential of anti-cancer GR signaling.

### Cell desensitization to activated GR signaling: CpdA versus glucocorticoids

Chronic treatment with Gcs frequently result in the development of resistance to steroids (tachyphylaxis) [93], which is in part mediated by the decreased GR levels in the cells. It is known that GR agonists induce the repression of GR transcription as well as the destabilization of the GR at mRNA and protein levels [94]. The inhibition of GR transcription by Gcs is mediated by the recruitment of activated GR to the negative GRE in exon 6 of *GR*, followed by the assembly of a GR-NCoR1-HDAC3 repression complex at the transcriptional start site of the *GR* gene [94]. Interestingly, CpdA did not induce GR loading in exon 6 (Haegeman and Cidlowski, unpublished data).

Although Gcs also induce GR degradation by proteasome [87], CpdA, in contrast, did not trigger significant GR degradation in lymphoid cells or in synoviocytes [38, 39, 70]. The differential effects of CpdA on crucial steps in GR degradation by the proteasome, such as GR phosphorylation and ubiquitination for example, remain to be investigated.

Another mechanism of cell desensitization common to Gcs is mediated by a negative feedback loop via the GR molecular chaperone FKBP51, also referred to as FK506-binding protein 5 [95]. FKBP51 sequesters the GR in the cytoplasm and prevents nuclear translocation [96, 97]. We showed that, in contrast to Gcs, CpdA did not activate *FKBP51* gene expression [39, 83].

In summary, our experiments demonstrated that CpdA failed to induce major mechanisms underlying the development of cell resistance to activated GR signaling.

### Adverse metabolic and atrophogenic effects: CpdA versus glucocorticoids

Together with their excellent therapeutic effects, Gcs are notorious for their multiple adverse side effects which develop in different organs and tissues during chronic treatment. The long list of undesirable effects includes changes in glucose and fat metabolism, Cushing's syndrome, diabetes, glaucoma, osteoporosis, muscle and skin atrophy, decreased wound healing, changes in mood and central nervous system functions, growth retardation in pediatric patients, and infertility. Many of these effects are severe and sometimes irreversible [98–100]. A considerable number of metabolic and atrophogenic effects of Gcs are due to their catabolic activity in peripheral organs/tissues, in which protein degradation and lipolysis are induced to stimulate glucocneogenesis in liver and to maintain high blood glucose levels [12, 46]. These effects involve activation of genes encoding gluconeogenetic enzymes, such as glucose-6-phosphatase, phosphoenolpyruvate carboxykinase (*PEPCK*), tyrosine aminotransferase as well as fatty acid synthase [12, 46, 64], linking many adverse Gcs effects to GR TA. In addition, we and others found that the induction of GR target gene *REDD1* (regulated in development and DNA damage response 1) is central to the atrophogenic effects of Gcs in skin, subcutaneous adipose and muscle [101, 102]. Importantly, REDD1 KO animals appeared to be protected against Gcs-induced skin atrophy and muscle waste [101, 103].

Although CpdA side effects during chronic *in vivo* treatment remain to be comprehensively analyzed, our results and reports by others, indicate that CpdA induces fewer adverse effects both in control animals and in animal models of diabetes, rheumatoid arthritis, osteoporosis, and Duchene muscle dystrophy when compared to Gcs [67, 73, 104, 105]. Indeed, in contrast to Gcs, CpdA did not induce key liver gluconeogenic enzymes, glucose-6-phosphatase and *PEPCK*, did not affect hepatic glycogen formation and, as a result did not induce hyperglycemia and hyperinsulinemia after systemic chronic animal treatment [64, 106]. It is important to note that although decreased β-cell insulin production and insulin resistance induced by Gcs may lead to the induction of type II diabetes or aggravation of pre-existing diabetes in patients [47], CpdA did not impair glucose metabolism, suggesting a reduced risk for induced type II diabetes by this GR ligand. Furthermore, CpdA was effective in the prevention and treatment of experimental inflammatory diabetes, which resembles type I diabetes in patients [73].

In contrast to steroids, CpdA did not induce muscle waste when it was used systemically in *mdx* mice [67]. CpdA also did not induce skin atrophy in rats and mice when applied topically [107, 108], which we have correlated with its inability to induce atrophogene *REDD1* in skin (Budunova, Haegeman, unpublished). In addition, the inhibitory effect of CpdA on collagen type I and III gene expression in skin, the key molecular change related to atrophy in dermis, was favorably diminished when compared to Gcs [107, 108].

Chronic administration of Gcs also leads to bone loss and osteoporosis due to reduced bone turnover, induction of osteoblast and osteocyte apoptosis, negative effects on *Collagen 1 and Runx2*, genes directly involved in bone formation, as well as activation of osteoclasts via activation of *RANKL* and *osteopontin* genes [12, 67]. The negative effects of CpdA on the bone metabolism were significantly weaker compared to Gcs in both newborn and adult mice [67, 104, 105].

Exogenous Gcs suppress the hypothalamic pituitary adrenal (HPA) axis, a major component of the neuroendocrine system, which controls stress adaptation and many body functions, including the immune response, mood and emotions, energy storage, while also regulating the biosynthesis of corticosteroids via ACTH in the adrenals [12]. ACTH is the key regulatory hormone in the HPA feedback loop, and its expression is negatively regulated by Gcs [12]. It is interestingly to note that even though GR TR by CpdA is overall very efficient, CpdA did not affect the HPA axis significantly as was assessed by the lack of changes in corticosterone and ACTH serum levels [63, 64].

It is however, important to state that CpdA has some limitations possibly due to its restricted chemical stability. CpdA has a relatively narrow therapeutic window and when used at high doses (~15 mg/kg) for longer periods it may gradually induce apoptosis via GR-independent metabolites [8, 64]. Although the anti-cancer effect of CpdA in the lymphoma xenograft model was achieved at lower doses (Fig. 3C), without inducing significant adverse effects during 4–6 wk animal treatment, the treatment of inflammatory diseases in animal models are reported to require higher doses of CpdA [62, 63, 65, 66].

Thus, in our ongoing work we have initiated synthesis of more stable SEGRA using CpdA as a prototype. To date, we and others have used a racemic Compound A for all *in vitro* and *in vivo* studies, even though it is known that enantiomers of chiral molecules including steroid receptor ligands often exhibit different chemical and biological properties [109, 110]. Thus, alternative approaches to improve CpdA ligand binding and pharmacological properties could involve its synthesis as an enantiopure compound.

## CONCLUSIONS

In this review, we summarized the results of 10 years of research collaboration between several academic organizations: Stellenbosch University (S. Africa), University of Ghent (Belgium), Northwestern University (Chicago, USA) and the Blokhin Cancer Center (Moscow, Russia), that led not only to the identification of CpdA as a unique SEGRA but also to the characterization of its ligand properties as well as its anti-inflammatory and anti-cancer activities. These studies unraveled the unusual biological properties of CpdA, but also provided a greater understanding of the mechanisms underlying the therapeutic and adverse effects of Gcs. To date no other SEGRA has been shown to exhibit the distinct profile of a dissociating GR ligand as has CpdA both *in vitro* and *in vivo* − preventing GR dimerization and subsequent TA, while switching GR activity towards TR. We also showed that CpdA is a dual GR/AR ligand and can act as an anti-inflammatory anti-androgen. The very beneficial pharmacological profile of CpdA can be attributed to its properties as a GR ligand retaining the therapeutic anti-inflammatory and anti-cancer potential of Gcs, while inducing fewer side effects. CpdA is furthermore less likely to induce patient resistance as it does not activate the major mechanisms of cell desensitization typical for Gcs. Overall, these properties make CpdA and its prospective derivatives very attractive candidates for clinical applications in the treatment of various inflammatory and autoimmune diseases such as rheumatoid arthritis, asthma and type I diabetes, neuropathy, muscle dystrophies as well as cancer, especially in combinational chemotherapy.

## SUPPLEMENTARY DATA FIGURE AND TABLES







## Abbreviations

AP-1: activator protein 1
AR: androgen receptor
BZ: Bortezomib
CIA: collagen-induced arthritis
CpdA: Compound A
DHT: dihydrotestosterone
EAE: experimental autoimmune encephalomyelitis
EAN: experimental autoimmune neuritis
FKBP51: FK506 binding protein 5
FRET: fluorescence resonance energy transfer
Gcs: glucocorticoids
GILZ: glucocorticoid-induced leucine zipper
GR: glucocorticoid receptor
GRE: glucocorticoid responsive element
IFN: interferon
IL: interleukin
NF-κB: nuclear factor kappa B
REDD1: regulated in development and DNA damage response 1
SEGRA: selective glucocorticoid receptor activator
STAT: signal transducer and activator of transcription
TA: transactivation
T-bet: T-box expressed in T cells
TF: transcription factor
Th: T helper
TNF: tumor necrosis factor
TR: transrepression
