# Supplementary material for: Discovery of Compound A – a selective activator of the glucocorticoid receptor with anti-inflammatory and anti-cancer activity
Source: Oncotarget. 2015 Oct 2;6(31):30730–44. doi: 10.18632/oncotarget.5078 (PMC4741564; doi:10.18632/oncotarget.5078)
Supplement: Supplementary file 1 [file oncotarget-06-30730-s001.pdf]

SUPPLEMENTARY DATA

SUPPLEMENTARY MATERIALS AND METHODS

Cell cultures and treatments

Prostate cancer (PC) LNCaP and PC3 cells and mantle cell lymphoma Granta-519 cells (American Type Culture Collection (ATCC)) were cultured in RPMI 1640 (Life Technologies) with 10% fetal bovine serum (referred thereafter as complete medium). LNCaP-GR cells and empty virus–expressing (LNCaP-V) control cells were generated by lentivirus infection. Cells were treated with CpdA (see below) or Dexamethasone (Dex; Sigma-Aldrich).

Synthesis of CpdA

The 2-(4-acetoxyphenyl)-2-chloro-N-methyl-ethylammonium chloride (CpdA) was synthesized from (±)-Synephrine and acetyl chloride as described [45].

Colony formation in soft agar

The colony-forming assay was performed as described previously [45]. Forty-eight hours after plating, cells were treated with CpdA, or vehicle (0.1% ethanol) for 2 wk. Complete medium with CpdA was changed twice weekly. Stably infected LNCaP-GR and LNCaP-V cells were cultured in the presence of 6 μg/μL blasticidin to maintain the selection. Images of entire wells were taken and colonies with diameter of > 50 μm were counted using AxioVision LE Rel. 4.5 software (Carl Zeiss MicroImaging, Inc.). Each experimental group consisted of six wells.

RNA extraction

RNA was isolated from Granta and LNCaP cells treated with Dex, CpdA, CpdA, or vehicle (0.1% ethanol and/or DMSO) for 8–16 h using TRIzol reagent according to the manufacturer's protocol. 1 ul aliquot was used for RNA fluorometric quantification (Qubit, Invitrogen) and remainder stored at −80°C until further analysis.

Gene profile analysis

The Illumina Human HT12 Expression BeadChips (DNA microarray) were utilized to profile gene expression in Granta-519 and LNCaP-GR cells. The Illumina BeadArray Reader and BeadScan software were used to scan and extract raw intensity values. The complete array results and experimental details were deposited to NCBI, the access number is GSM1815466 (Granta 519) and GSM1823672 (for LNCaP-GR).

Quantitative RT-PCR

First-strand cDNA was synthesized from 1 μg total RNA using random hexamer primers and 1 μL Superscript II Reverse Transcriptase (Invitrogen). Samples were stored at –20°C until analysis. All gene specific primers were designed using Oligo 7.0 and synthesized by Integrated DNA Technologies (Coralville, IA, USA) and are listed in Supplementary Table 3. Primers were optimized for appropriate primer concentration using a concentration gradient (150, 200, 250, 300, and 350 nM) and validated using a 10-log dilution curve. A working solution of cDNA was prepared by diluting samples 1:10 with DEPC-treated water. Five microliters of cDNA working solution was added to a 25 μL Power SYBR Green Master Mix (Applied Biosciences), 1 μg of cDNA and 500 nM of each specific forward and reverse primer (Supplementary Table 3). Quantitative real-time PCR analysis was carried out using Bio-Rad iQ5 detection system and software (Bio-Rad Laboratories, Hercules, CA, USA). Standard thermocycler conditions were as follows: 95°C for 10 min, followed by 40 cycles of 95°C for 15 sec, 60°C for 30 sec, and 72°C for 30 sec. Relative fold change in target mRNAs was quantified using the ΔΔCt method where ΔΔCt was determined by subtracting the average control ΔCt from the ΔCt of the sample. All reverse-transcribed cDNA samples were assayed in triplicate for each gene, and melt curve analyses were performed to ensure specificity of amplification. Melt curve analysis was carried out for 81 cycles with 0.5°C temperature increase from 55°C to 95°C. *RPL27* gene was used as the reference gene for the experiments.

Mouse studies

Animals were maintained under the protocols approved by the Northwestern University and Blokhin Cancer Research Center Institutional Animal Care and Use Committees (IACUC). Female athymic nude mice (Charles River, Wilmington, Massachusetts) at 6–8 weeks of age were injected subcutaneously with 1.2×10^6 PC3 or 1×10^7 Granta cells in 200 ul BD Matrigel in the right flank to generate solid human prostate cancer or lymphoma xenografts. When tumors reached 50 mm3, mice received CpdA, Dex or PBS intraperitoneally (i.p.) 3 times/week for 35 days. Tumor volume was determined twice per week by measuring tumor diameter with electronic calipers.

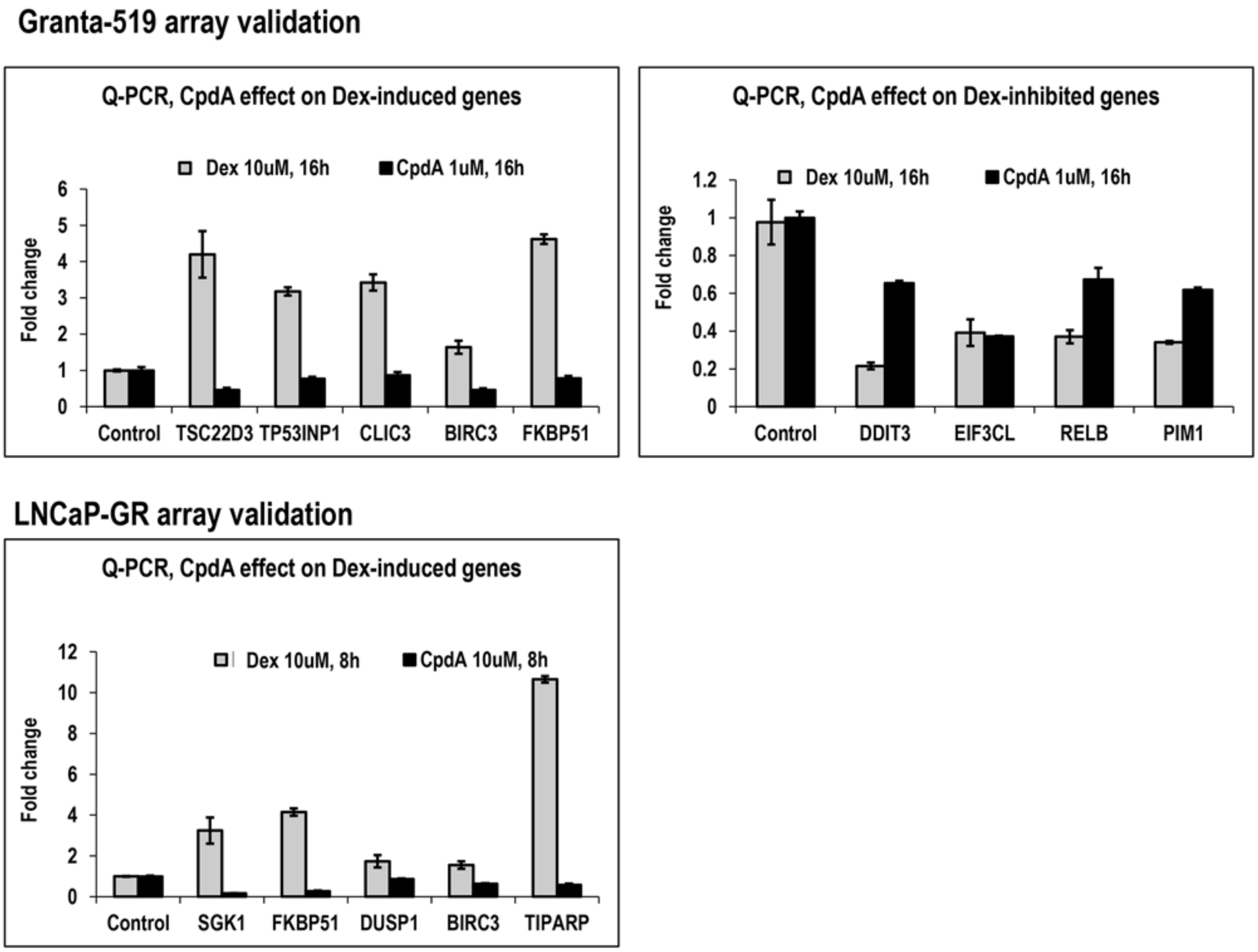

Supplementary Figure S1: Validation of DNA arrays results in LNCaP-GR (A) and Granta 519 (B) cells. Gene expression in LNCaP-GR **A.** and Granta 519 **B.** cells was analyzed by quantitative RT-PCR after LNCaP-GR cells treatment with 1 µM Dex or 5 µM CpdA for 8 h, and Granta-519 cells treatment with 1uM Dex or 1uM CpdA for 16 h.

Supplementary Table S1: Granta 519 microarray data. Granta-519 cells expressing endogenous GR, were treated with vehicle, Dex (1 μM) or CpdA (1 μM) for 16 h. Total RNA was extracted, and global gene expression changes were evaluated by Illumina DNA arrays (three individual RNA samples/group). The complete array results and experimental details were deposited to NCBI, the access number is GSE71099.

Supplementary Table S2: LNCaP-GR microarray data. LNCaP-GR cells stably infected with GR-expressing lentivirus, were treated with vehicle (EtOH), glucocorticoid Dexamethasone (Dex, 1 μM) or CpdA (5 μM) for 8h. Total RNA was extracted, and global gene expression changes were evaluated by Illumina DNA arrays (three individual RNA samples/group). The complete array results and experimental details were deposited to NCBI, the access number is GSE71102

Supplementary Table S3: Supplementary Table S3: Primer sequences and experimental methods

Gene	Forward primer	Reverse primer
SGK1	5′- catattatgtcggagcggaatgt -3′	5′- tgtcagcagtctggaaagaga -3′
BIRC3	5′- tttccgtggctcttattcaaact -3′	5′- gcacagtggtaggaacttctcat -3′
DDIT3	5′-cccttcagtgacttccatgac-3′	5′- tcaccatacagcagcctgag-3′
CLIC3	5′- cctcaagggcgtacctttcac -3′	5′- gtcgctgtcatagagcagga -3′
TP53INP1	5′-ttcctccaaccaagaaccaga-3′	5′-gctcagtaggtgactcttcact-3′
DUSP1	5′- accaccaccgtgttcaacttc -3′	5′- tgggagaggtcgtaatgggg-3′
TIPARP	5′- atcttttgcacccagtttcaagt -3′	5′- tcaaacacgaggtcaagggaa -3′
EIF3A	5′- agatgaggacagaggacctagac-3′	5′- tcagcattccgccaggatga-3′
RELB	5′- ccattgagcggaagattcaact -3′	5′- ctgctggtcccgatatgagg -3′
PIM1	5′- gagaaggaccggatttccgac -3′	5′- cagtccaggagcctaatgacg -3′
FKBP51	5′- gaatggtgaggaaacgccgat -3′	5′- tgccaagactaaagacaaatggt -3′
TSC22D3	5′- aacaccgaaatgtatcagaccc -3′	5′- tgtccagcttaacggaaacca -3′
RPL27	5′- accgctacccccgcaaagtg -3′	5′- cccgtcgggccttgcgttta -3′
