# Supplementary material for: Discovery of Compound A – a selective activator of the glucocorticoid receptor with anti-inflammatory and anti-cancer activity
Source: Oncotarget. 2015 Oct 2;6(31):30730–44. doi: 10.18632/oncotarget.5078 (PMC4741564; doi:10.18632/oncotarget.5078)
Supplement: Supplementary file 2 [file oncotarget-06-30730-s002.docx]

| **Symbol** | **Description** | **P.Value** | **FoldChange** | **Control- 1** | **Control- 2** | **Dex 1uM 16h - 1** | **Dex 1uM 16h - 2** |
| --- | --- | --- | --- | --- | --- | --- | --- |
| RN7SK | RNA. 7SK small nuclear | 3.09E-08 | 29.971563 | 40.31887 | 33.62700555 | 1134.241152 | 1073.766699 |
| TSC22D3 | TSC22 domain family. member 3 | 1.13E-07 | 9.852189559 | 252.5379406 | 228.0772885 | 2343.899033 | 2385.257633 |
| APOD | apolipoprotein D | 7.45E-07 | 9.096260828 | 16.03614395 | 16.77510306 | 157.4198163 | 141.3941858 |
| TSC22D3 | TSC22 domain family. member 3 | 3.86E-06 | 8.305361777 | 21.75939655 | 25.31213017 | 191.0029111 | 198.9081899 |
| RN7SK | RNA. 7SK small nuclear | 1.03E-07 | 8.219018525 | 276.6148939 | 272.1303042 | 2241.405811 | 2268.672948 |
| TSC22D3 | TSC22 domain family. member 3 | 4.62E-07 | 7.420074696 | 486.2449301 | 496.2798133 | 3389.539957 | 3919.742094 |
| TP53INP1 | tumor protein p53 inducible nuclear protein 1 | 4.91E-06 | 6.846445475 | 50.26280201 | 56.58129128 | 358.0037698 | 372.3593453 |
| CLIC3 | chloride intracellular channel 3 | 1.26E-06 | 6.413632313 | 16.63772999 | 13.44080093 | 91.88647129 | 100.1096089 |
| TP53INP1 | tumor protein p53 inducible nuclear protein 1 | 1.38E-06 | 5.964818421 | 126.4927789 | 117.0390539 | 672.7265835 | 782.9831239 |
| RN5S9 | RNA. 5S ribosomal 9 | 1.25E-06 | 5.494269626 | 457.001564 | 425.3696319 | 2298.614669 | 2552.924259 |
| SNORD3A | small nucleolar RNA. C/D box 3A | 2.44E-06 | 5.279434768 | 194.1684129 | 179.8070685 | 898.7196244 | 1082.769397 |
| SNORD3C | small nucleolar RNA. C/D box 3C | 1.08E-06 | 5.248139549 | 64.171929 | 62.8236787 | 339.9429459 | 326.6428627 |
| RNU4-2 | RNA. U4 small nuclear 2 | 5.50E-05 | 5.23797182 | 49.00565817 | 28.30697649 | 185.2757751 | 205.4222051 |
| LRRC16A | leucine rich repeat containing 16A | 4.71E-07 | 5.11964386 | 36.8831261 | 37.78781918 | 195.4861363 | 186.8715083 |
| IL1R2 | interleukin 1 receptor. type II | 5.20E-07 | 5.096848413 | 40.26834216 | 42.89705547 | 213.4174574 | 210.2639048 |
| SERPINA9 | serpin peptidase inhibitor. clade A (alpha-1 antiproteinase. antitrypsin). member 9 | 8.53E-06 | 5.081309902 | 19.86576572 | 27.62865681 | 121.6714708 | 116.4736509 |
| SNORD3D | small nucleolar RNA. C/D box 3D | 6.04E-07 | 4.948327226 | 210.3054394 | 200.565239 | 1022.804466 | 1009.7884 |
| SERPINA9 | serpin peptidase inhibitor. clade A (alpha-1 antiproteinase. antitrypsin). member 9 | 1.85E-06 | 4.857045271 | 15.14242326 | 13.20165408 | 69.93614656 | 67.43204549 |
| RNU1-3 | RNA. U1 small nuclear 3 | 1.01E-05 | 4.817434385 | 375.0944733 | 481.821182 | 1879.69101 | 2231.370596 |
| RNU1-9 | RNA. U1 small nuclear 9 | 7.82E-07 | 4.632712868 | 368.730266 | 352.4063425 | 1671.80723 | 1668.157573 |
| RNU1-5 | RNA. U1 small nuclear 5 | 1.40E-06 | 4.570676402 | 520.0185152 | 594.6882539 | 2527.775909 | 2555.821662 |
| ZFP36L2 | zinc finger protein 36. C3H type-like 2 | 0.00014168 | 4.478584693 | 46.12621428 | 53.84357626 | 215.4301308 | 231.2367452 |
| SCARNA8 | small Cajal body-specific RNA 8 | 0.00016705 | 4.388134164 | 32.87249594 | 21.49318512 | 119.8300716 | 113.5343912 |
| C3orf52 | chromosome 3 open reading frame 52 | 8.20E-06 | 4.207908826 | 31.78661358 | 31.56963054 | 130.7847421 | 135.8592848 |
| IL1R2 | interleukin 1 receptor. type II | 8.86E-06 | 4.15296177 | 106.01905 | 116.8482936 | 438.1012889 | 487.6942311 |
| FKBP5 | FK506 binding protein 5 | 1.91E-05 | 4.132902731 | 481.6181669 | 509.0255427 | 1718.908844 | 2436.127186 |
| SNORD13 | small nucleolar RNA. C/D box 13 | 0.0002481 | 4.069604837 | 139.7083272 | 84.03130475 | 395.1489652 | 492.0475031 |
| MYBPC1 | myosin binding protein C. slow type | 3.54E-05 | 3.958301863 | 29.72929105 | 22.59587698 | 117.7393114 | 89.3943528 |
| GLIPR2 | GLI pathogenesis-related 2 | 4.82E-06 | 3.891009802 | 35.79166429 | 43.09913638 | 149.6895904 | 156.021163 |
| RNASET2 | ribonuclease T2 | 5.82E-06 | 3.496685252 | 656.5199552 | 676.7993537 | 2262.820948 | 2400.88169 |
| FAM172A | family with sequence similarity 172. member A | 6.24E-05 | 3.493059279 | 60.24992027 | 46.50388341 | 160.4391916 | 213.0821864 |
| RGS1 | regulator of G-protein signaling 1 | 0.00019466 | 3.45202248 | 18.66504779 | 12.48486458 | 46.57517675 | 59.6218793 |
| SPATA13 | spermatogenesis associated 13 | 5.70E-06 | 3.427382583 | 16.74806816 | 18.30696529 | 61.20690437 | 58.84449412 |
| TMEM2 | transmembrane protein 2 | 1.41E-05 | 3.399841556 | 20.40942192 | 21.40853422 | 79.79004982 | 63.29745618 |
| SNORD46 | small nucleolar RNA. C/D box 46 | 0.00017077 | 3.300375502 | 33.1766818 | 22.26892591 | 82.23171608 | 97.86323636 |
| NAPSB | napsin B aspartic peptidase pseudogene | 1.17E-05 | 3.275137216 | 264.0460278 | 324.0991713 | 953.592607 | 962.6173472 |
| RNU1-1 | RNA. U1 small nuclear 1 | 5.66E-05 | 3.257939877 | 557.3632705 | 597.185829 | 1842.964867 | 1916.977052 |
| PLCH2 | phospholipase C. eta 2 | 1.03E-05 | 3.179881685 | 64.60263536 | 54.84232212 | 184.4590709 | 194.2173285 |
| ZNF831 | zinc finger protein 831 | 1.70E-05 | 3.173463355 | 40.48005718 | 33.39466536 | 117.6049439 | 115.7602909 |
| MYO3B | myosin IIIB | 0.00078573 | 3.126549658 | 38.79889377 | 30.83129898 | 126.1820974 | 92.67105034 |
| SESN1 | sestrin 1 | 4.24E-05 | 3.089143327 | 174.7816279 | 178.2778111 | 495.3907954 | 600.2349195 |
| FCER1G | Fc fragment of IgE. high affinity I. receptor for; gamma polypeptide | 6.59E-06 | 3.056902578 | 75.84145075 | 83.28215006 | 246.2785178 | 239.6598184 |
| RNU11 | RNA. U11 small nuclear | 3.35E-05 | 3.028492959 | 28.65100494 | 24.54332461 | 80.9343121 | 79.68814326 |
| TMEM100 | transmembrane protein 100 | 0.00012452 | 3.027561535 | 37.72697375 | 33.67836498 | 119.3898307 | 97.54872347 |
| SCARNA14 | small Cajal body-specific RNA 14 | 0.00065777 | 3.003471054 | 96.71050722 | 56.20666563 | 213.8244918 | 229.324754 |
| METTL7A | methyltransferase like 7A | 3.94E-05 | 2.994873358 | 59.94625023 | 45.56736547 | 155.8664828 | 157.1882691 |
| BMP7 | bone morphogenetic protein 7 | 0.00010176 | 2.99306739 | 262.9773454 | 277.2432548 | 681.7615952 | 958.0314874 |
| RNU1-8 | RNA. U1 small nuclear 8 | 1.35E-05 | 2.937977035 | 242.6721811 | 244.4269655 | 671.9779017 | 761.9226985 |
| PRDM1 | PR domain containing 1. with ZNF domain | 1.35E-05 | 2.925989381 | 21.80944915 | 19.07241501 | 60.22351625 | 59.13298021 |
| RN18S1 | RNA. 18S ribosomal 1 | 1.69E-05 | 2.920810051 | 1567.881878 | 1491.867339 | 4393.409778 | 4542.013945 |
| BMF | Bcl2 modifying factor | 6.04E-05 | 2.880077216 | 165.0185153 | 163.44367 | 469.5640692 | 476.4465495 |
| PNPLA7 | patatin-like phospholipase domain containing 7 | 1.68E-05 | 2.879371376 | 78.98640889 | 79.38265035 | 246.171892 | 211.1712868 |
| ESPNL | espin-like | 3.60E-05 | 2.829994976 | 48.97440722 | 49.02516179 | 137.6376535 | 139.7083272 |
| KCNH4 | potassium voltage-gated channel. subfamily H (eag-related). member 4 | 3.44E-05 | 2.829921754 | 18.32613419 | 15.52064744 | 49.35736065 | 46.15062889 |
| LOC338758 | hypothetical LOC338758 | 1.43E-05 | 2.820791969 | 66.54762799 | 64.87850741 | 181.9199839 | 188.8404985 |
| LPIN1 | lipin 1 | 1.20E-05 | 2.785779007 | 326.1659491 | 331.2180979 | 883.9028978 | 948.508973 |
| PIK3IP1 | phosphoinositide-3-kinase interacting protein 1 | 1.31E-05 | 2.7856812 | 250.2109049 | 271.2030621 | 701.7924876 | 750.334532 |
| LOC100133923 | hypothetical protein LOC100133923 | 1.93E-05 | 2.754924925 | 123.883056 | 111.3064213 | 340.1961642 | 307.6254444 |
| LRRC16A | leucine rich repeat containing 16A | 4.94E-05 | 2.75256843 | 24.06692916 | 27.0875245 | 77.29561822 | 63.90154677 |
| STMN3 | stathmin-like 3 | 1.44E-05 | 2.721252923 | 53.30072821 | 50.28855472 | 135.8754029 | 146.082862 |
| TXNIP | thioredoxin interacting protein | 6.81E-05 | 2.694070788 | 1291.642369 | 1091.538547 | 3224.041606 | 3173.940112 |
| NEK8 | NIMA (never in mitosis gene a)- related kinase 8 | 1.66E-05 | 2.691963123 | 29.08918489 | 26.21767402 | 75.69383061 | 73.01354581 |
| BMF | Bcl2 modifying factor | 1.52E-05 | 2.684169907 | 193.3616801 | 194.0451442 | 533.9247419 | 506.3060887 |
| ZNF160 | zinc finger protein 160 | 0.00013629 | 2.678519248 | 31.09751273 | 27.12077135 | 72.11714956 | 83.90323035 |
| MYBL1 | v-myb myeloblastosis viral oncogene homolog (avian)-like 1 | 0.00107736 | 2.672790334 | 42.2318946 | 53.26815647 | 102.0010556 | 157.5554208 |
| NLRP8 | NLR family. pyrin domain containing 8 | 2.21E-05 | 2.671582264 | 528.7856532 | 604.663825 | 1459.889145 | 1563.186784 |
| VTRNA1-1 | vault RNA 1-1 | 0.00172627 | 2.648603625 | 30.66486382 | 21.05608591 | 55.71682029 | 81.29546114 |
| LEP | leptin | 0.00016292 | 2.64133069 | 130.3468956 | 133.8097961 | 320.5483204 | 379.6126278 |
| GDPD5 | glycerophosphodiester phosphodiesterase domain containing 5 | 4.96E-05 | 2.629604481 | 76.116474 | 72.87040669 | 195.441934 | 196.2424539 |
| ANKRD36B | ankyrin repeat domain 36B | 0.00015549 | 2.629057741 | 43.73365891 | 42.82515771 | 102.1312643 | 126.7524391 |
| SORT1 | sortilin 1 | 5.87E-05 | 2.609251187 | 46.54384705 | 51.44632665 | 117.2086377 | 139.0877267 |
| RNU6-15 | RNA. U6 small nuclear 15 | 3.91E-05 | 2.591246483 | 388.8721339 | 425.674096 | 973.7490162 | 1141.44362 |
| SLC16A3 | solute carrier family 16. member 3 (monocarboxylic acid transporter 4) | 3.02E-05 | 2.589523896 | 131.0036266 | 124.7779229 | 309.0525412 | 354.6733832 |
| LOC100129322 | hypothetical protein LOC100129322 | 2.81E-05 | 2.584622258 | 28.34847249 | 29.6708948 | 77.8379527 | 72.18767628 |
| ZNF223 | zinc finger protein 223 | 3.18E-05 | 2.573438469 | 162.1934902 | 173.6770549 | 437.5075568 | 426.4006758 |
| DUSP2 | dual specificity phosphatase 2 | 0.00013939 | 2.560749402 | 120.6771455 | 149.9546316 | 342.9122726 | 346.0477617 |
| RASSF6 | Ras association (RalGDS/AF-6) domain family member 6 | 4.25E-05 | 2.555101718 | 24.15026614 | 28.06162751 | 62.79522603 | 70.45706317 |
| RNU11 | RNA. U11 small nuclear | 0.00117556 | 2.5543687 | 43.91313072 | 29.55479163 | 86.13672474 | 98.31077421 |
| TSHR | thyroid stimulating hormone receptor | 0.00299799 | 2.54707658 | 42.74644583 | 22.38967579 | 85.22390351 | 72.85685139 |
| BMPR2 | bone morphogenetic protein receptor. type II (serine/threonine kinase) | 0.00106598 | 2.546914521 | 35.04983038 | 28.07023531 | 78.79050735 | 81.00033519 |
| KLHL14 | kelch-like 14 (Drosophila) | 0.00185553 | 2.5393643 | 27.29674253 | 15.45950082 | 49.0736023 | 55.45087076 |
| IL23A | interleukin 23. alpha subunit p19 | 8.51E-05 | 2.52983222 | 141.5141606 | 177.2540793 | 420.6925705 | 381.6055963 |
| CXCR4 | chemokine (C-X-C motif) receptor 4 | 2.13E-05 | 2.526940657 | 477.8441902 | 502.7708879 | 1292.365174 | 1187.028845 |
| UMODL1 | uromodulin-like 1 | 6.29E-05 | 2.526784363 | 30.75271595 | 33.39171937 | 83.93537629 | 78.11124828 |
| GPR1 | G protein-coupled receptor 1 | 0.00052267 | 2.508647792 | 202.519827 | 236.505641 | 557.3632705 | 540.8166939 |
| CGN | cingulin | 8.93E-05 | 2.505894873 | 49.75414855 | 48.35943442 | 115.8331562 | 130.4377554 |
| TNFRSF13B | tumor necrosis factor receptor superfamily. member 13B | 0.00015271 | 2.499897836 | 128.1863714 | 139.891563 | 305.5461603 | 366.7761331 |
| LOC100132585 | similar to speedy homolog A | 3.96E-05 | 2.492843317 | 353.3293417 | 332.624503 | 811.156127 | 900.3667641 |
| TMEM106A | transmembrane protein 106A | 0.00057296 | 2.470394405 | 107.6818767 | 163.5098382 | 343.2306018 | 313.0639731 |
| FGD2 | FYVE. RhoGEF and PH domain containing 2 | 3.18E-05 | 2.460039708 | 395.0124945 | 381.5149543 | 981.0289498 | 929.6614219 |
| TSC22D1 | TSC22 domain family. member 1 | 0.00024229 | 2.459242412 | 19.11486301 | 22.11665346 | 58.69809402 | 43.55813571 |
| PODXL | podocalyxin-like | 0.00077572 | 2.451767748 | 43.87638565 | 60.14939394 | 145.8076907 | 108.8028633 |
| NOTCH2NL | notch 2 N-terminal like | 0.00168511 | 2.432425292 | 49.41900692 | 41.59280642 | 120.5965443 | 100.8454705 |
| C20orf94 | chromosome 20 open reading frame 94 | 0.00178059 | 2.422736116 | 21.63124242 | 14.68805004 | 50.96434318 | 36.59244283 |
| NBPF9 | neuroblastoma breakpoint family. member 9 | 0.00101145 | 2.422151965 | 54.63286474 | 82.15952386 | 157.1762416 | 167.5435689 |
| RAB11FIP4 | RAB11 family interacting protein 4 (class II) | 0.00010909 | 2.420946651 | 74.29394538 | 93.53021044 | 207.90353 | 195.8907409 |
| RNU6ATAC | RNA. U6atac small nuclear (U12-dependent splicing) | 0.0019853 | 2.417510843 | 24.58350537 | 15.96516287 | 55.95366438 | 40.99449024 |
| SCARNA11 | small Cajal body-specific RNA 11 | 0.00025709 | 2.414418728 | 31.34710043 | 23.43506793 | 60.8788882 | 70.34319041 |
| PCDHB9 | protocadherin beta 9 | 0.00227998 | 2.410284192 | 43.96639219 | 52.19515747 | 113.2118135 | 117.7594583 |
| FOXO3 | forkhead box O3 | 0.00011777 | 2.386971751 | 93.86651695 | 115.1522234 | 247.446911 | 248.8831832 |
| MYBPC1 | myosin binding protein C. slow type | 0.00023325 | 2.384205883 | 27.85862175 | 21.03635141 | 54.22641744 | 61.43369553 |
| C9orf140 | chromosome 9 open reading frame 140 | 8.97E-05 | 2.374139026 | 697.1635226 | 681.3617379 | 1553.697443 | 1723.289496 |
| ARHGEF37 | Rho guanine nucleotide exchange factor (GEF) 37 | 0.00022105 | 2.371108428 | 20.41105333 | 25.62806639 | 52.09493397 | 56.45320387 |
| CCDC24 | coiled-coil domain containing 24 | 0.00030599 | 2.35703379 | 74.17547584 | 60.16641994 | 154.9138869 | 160.0500366 |
| CXCR4 | chemokine (C-X-C motif) receptor 4 | 2.46E-05 | 2.353703291 | 1168.467768 | 1153.827885 | 2744.581166 | 2721.354493 |
| KIAA0513 | KIAA0513 | 0.00073721 | 2.348690501 | 44.3789457 | 38.3786908 | 99.1068534 | 94.80146118 |
| C9orf140 | chromosome 9 open reading frame 140 | 3.91E-05 | 2.345781347 | 309.396719 | 345.1560146 | 760.2231033 | 772.9744575 |
| CCBE1 | collagen and calcium binding EGF domains 1 | 0.00019429 | 2.343557468 | 509.164965 | 574.294399 | 1412.984338 | 1136.598192 |
| SLC5A8 | solute carrier family 5 (iodide transporter). member 8 | 0.00457443 | 2.338210073 | 39.00432219 | 51.81083654 | 129.7749046 | 85.13530095 |
| HOXA6 | homeobox A6 | 0.00064787 | 2.331055436 | 66.75925287 | 59.66974835 | 155.0066303 | 139.6434605 |
| ZNF669 | zinc finger protein 669 | 0.00492273 | 2.327566156 | 46.36414514 | 82.63140887 | 167.1482681 | 124.1736939 |
| CFLAR | CASP8 and FADD-like apoptosis regulator | 0.00150519 | 2.326863129 | 42.85743395 | 32.5344641 | 105.583533 | 71.50152551 |
| SERPINI1 | serpin peptidase inhibitor. clade I (neuroserpin). member 1 | 0.00013464 | 2.317961 | 49.7579194 | 40.96879289 | 109.3541424 | 100.159556 |
| GRIPAP1 | GRIP1 associated protein 1 | 0.00229283 | 2.315979636 | 399.7592237 | 596.7275426 | 1361.082975 | 940.0683857 |
| SERHL | serine hydrolase-like | 0.00025648 | 2.315479091 | 24.53792578 | 23.9064518 | 50.1055678 | 62.76950602 |
| BASP1 | brain abundant. membrane attached signal protein 1 | 3.51E-05 | 2.312403959 | 444.2602929 | 441.0107141 | 985.046998 | 1063.548001 |
| C8orf45 | chromosome 8 open reading frame 45 | 0.00012368 | 2.311932418 | 1250.037528 | 1449.247708 | 3368.870461 | 2874.296932 |
| DUSP19 | dual specificity phosphatase 19 | 0.00048611 | 2.311727993 | 620.0242974 | 740.5755388 | 1396.509111 | 1757.145653 |
| RUNDC2C | RUN domain containing 2C | 0.00010511 | 2.307295115 | 53.05507149 | 64.00922703 | 141.0372807 | 128.1863714 |
| HAUS2 | HAUS augmin-like complex. subunit 2 | 7.65E-05 | 2.306372312 | 201.6644169 | 204.1972344 | 490.7284307 | 446.3717922 |
| STXBP6 | syntaxin binding protein 6 (amisyn) | 8.54E-05 | 2.30375342 | 86.17850604 | 82.72957484 | 190.0130175 | 199.1353605 |
| TSPAN33 | tetraspanin 33 | 4.47E-05 | 2.297500953 | 174.3973118 | 176.5428795 | 400.2363942 | 406.054956 |
| RNF19A | ring finger protein 19A | 0.00120971 | 2.295495201 | 20.09106042 | 31.28749248 | 60.8021008 | 54.47632612 |
| SOCS1 | suppressor of cytokine signaling 1 | 0.00033671 | 2.29130979 | 53.42417464 | 57.24670604 | 111.1828829 | 144.4168988 |
| SNAP47 | synaptosomal-associated protein. 47kDa | 7.43E-05 | 2.287422091 | 144.0490526 | 128.6609059 | 293.4852179 | 330.4177742 |
| INF2 | inverted formin. FH2 and WH2 domain containing | 8.01E-05 | 2.280029981 | 28.45925208 | 32.28485857 | 73.0861479 | 65.35343417 |
| SHROOM4 | shroom family member 4 | 5.20E-05 | 2.279090165 | 236.4216512 | 231.0257148 | 553.3541543 | 512.7048294 |
| GNA12 | guanine nucleotide binding protein (G protein) alpha 12 | 6.84E-05 | 2.276670919 | 130.5695697 | 126.4927789 | 276.732795 | 309.3482028 |
| CD72 | CD72 molecule | 3.57E-05 | 2.274288394 | 349.7669981 | 336.6606285 | 766.8510378 | 794.239021 |
| LOC100188949 | hypothetical LOC100188949 | 5.32E-05 | 2.272072498 | 162.4904717 | 161.8201891 | 379.0311887 | 358.1211958 |
| SNORD56 | small nucleolar RNA. C/D box 56 | 0.00051267 | 2.267600775 | 36.78256869 | 38.92175536 | 78.97091052 | 93.21815892 |
| ZNF831 | zinc finger protein 831 | 0.00291748 | 2.261137531 | 43.52124736 | 45.35752069 | 118.2443122 | 85.3540906 |
| JSRP1 | junctional sarcoplasmic reticulum protein 1 | 5.32E-05 | 2.257947068 | 826.9484609 | 857.1337212 | 1797.051619 | 2010.916261 |
| MS4A7 | membrane-spanning 4-domains. subfamily A. member 7 | 0.00011773 | 2.246874746 | 19.16079548 | 20.5910939 | 41.08595045 | 48.47941182 |
| SERHL | serine hydrolase-like | 0.00404326 | 2.242993137 | 28.66418784 | 34.26616238 | 73.15559884 | 67.54814537 |
| TSC22D1 | TSC22 domain family. member 1 | 4.88E-05 | 2.230927323 | 66.36928861 | 63.35851423 | 145.7723204 | 143.5713965 |
| NBPF8 | neuroblastoma breakpoint family. member 8 | 0.00028416 | 2.230888921 | 29.65611605 | 37.39298259 | 78.49225628 | 70.312649 |
| EIF2AK4 | eukaryotic translation initiation factor 2 alpha kinase 4 | 0.00065889 | 2.225254334 | 147.9726546 | 160.5923416 | 322.4937058 | 364.8752149 |
| DDX51 | DEAD (Asp-Glu-Ala-Asp) box polypeptide 51 | 0.00021978 | 2.223882756 | 677.2437868 | 837.7889083 | 1732.223911 | 1619.941686 |
| SMAP2 | small ArfGAP2 | 4.38E-05 | 2.216360444 | 2460.545211 | 2350.377178 | 5436.35455 | 5225.669256 |
| POLD4 | polymerase (DNA-directed). delta 4 | 0.0001383 | 2.215856602 | 82.79396667 | 71.85149973 | 182.6437923 | 159.9237317 |
| ZNF652 | zinc finger protein 652 | 0.00098062 | 2.213217682 | 750.334532 | 940.5628808 | 1613.577236 | 2142.403523 |
| FAM40B | family with sequence similarity 40. member B | 9.02E-05 | 2.212093719 | 108.3521282 | 106.8250293 | 238.5877 | 237.3938487 |
| BLZF1 | basic leucine zipper nuclear factor 1 | 7.09E-05 | 2.203372867 | 831.7766575 | 912.3472482 | 1942.485439 | 1896.640924 |
| SMAD6 | SMAD family member 6 | 0.00041765 | 2.201495819 | 26.36877325 | 31.34710043 | 66.45646764 | 60.28173971 |
| LOC255167 | hypothetical LOC255167 | 0.0014205 | 2.199860516 | 642.5694205 | 953.592607 | 1910.990851 | 1551.724479 |
| BRI3BP | BRI3 binding protein | 7.12E-05 | 2.193562608 | 210.9613423 | 215.9897601 | 450.6101826 | 486.5585513 |
| DFFA | DNA fragmentation factor. 45kDa. alpha polypeptide | 0.00058751 | 2.18986387 | 99.24223873 | 133.9897443 | 264.478864 | 241.1078647 |
| MAGT1 | magnesium transporter 1 | 0.00021036 | 2.188415384 | 497.29762 | 622.8976034 | 1261.063207 | 1176.401864 |
| TNFSF14 | tumor necrosis factor (ligand) superfamily. member 14 | 0.00035424 | 2.186404395 | 191.7231083 | 253.9249031 | 503.3528231 | 462.3471994 |
| CARS2 | cysteinyl-tRNA synthetase 2. mitochondrial (putative) | 0.00010054 | 2.183823632 | 73.8227293 | 83.60552813 | 174.0859532 | 169.0816531 |
| RN28S1 | RNA. 28S ribosomal 1 | 0.00120429 | 2.180963712 | 5128.668613 | 3476.289423 | 8966.631318 | 9457.756556 |
| C9orf38 | chromosome 9 open reading frame 38 | 0.00016846 | 2.180494195 | 28.31814847 | 30.86531487 | 67.50124203 | 61.56496993 |
| BCL2L11 | BCL2-like 11 (apoptosis facilitator) | 0.00233567 | 2.180424624 | 29.89870906 | 31.78289535 | 69.780508 | 64.74316472 |
| MBTD1 | mbt domain containing 1 | 0.00084416 | 2.17644606 | 59.4081686 | 83.40541646 | 166.607152 | 140.8778113 |
| GAL3ST4 | galactose-3-O-sulfotransferase 4 | 0.01180793 | 2.175435691 | 48.1979517 | 34.06033126 | 106.1719028 | 73.17459844 |
| B3GALTL | beta 1.3-galactosyltransferase-like | 0.00480801 | 2.173450544 | 27.94112624 | 32.76807299 | 63.20515632 | 68.42925708 |
| LOC100134868 | hypothetical LOC100134868 | 0.0011318 | 2.166071803 | 157.1527866 | 186.1659644 | 396.3708625 | 346.3110483 |
| MCART1 | mitochondrial carrier triple repeat 1 | 0.00014218 | 2.165126839 | 2224.96464 | 2427.668134 | 4821.230028 | 5251.958256 |
| IDS | iduronate 2-sulfatase | 5.88E-05 | 2.164784528 | 140.8035056 | 149.76992 | 319.2457355 | 309.558199 |
| SLC35E1 | solute carrier family 35. member E1 | 0.00369712 | 2.162934589 | 483.5140876 | 668.8561862 | 993.5611998 | 1522.769006 |
| ARHGEF18 | Rho/Rac guanine nucleotide exchange factor (GEF) 18 | 7.80E-05 | 2.161792682 | 1123.295323 | 1043.283885 | 2436.127186 | 2248.146333 |
| ESR2 | estrogen receptor 2 (ER beta) | 0.00029708 | 2.161224122 | 25.070422 | 31.3318275 | 59.02101953 | 62.1641896 |
| MLKL | mixed lineage kinase domain-like | 0.00031228 | 2.160970991 | 77.66598399 | 59.99036531 | 149.1853246 | 145.8425085 |
| FAM102A | family with sequence similarity 102. member A | 8.53E-05 | 2.160667281 | 186.1659644 | 206.1804891 | 409.16457 | 437.9510988 |
| RECK | reversion-inducing-cysteine-rich protein with kazal motifs | 0.00266166 | 2.159920924 | 42.70051625 | 47.0919491 | 101.6861218 | 92.25582798 |
| LOC90586 | AOC3 pseudogene | 0.00048969 | 2.159234303 | 729.0010943 | 956.9773203 | 1873.194236 | 1736.387117 |
| IL28RA | interleukin 28 receptor. alpha (interferon. lambda receptor) | 0.00030756 | 2.157581296 | 31.66197782 | 28.90587218 | 66.96309655 | 63.62428679 |
| HSPC159 | galectin-related protein | 0.00367503 | 2.156173299 | 46.55627344 | 33.41708119 | 73.64609585 | 98.21194738 |
| LIX1L | Lix1 homolog (mouse)-like | 0.00031967 | 2.155865906 | 78.9424603 | 96.62690107 | 186.7084617 | 189.8839235 |
| BCYRN1 | brain cytoplasmic RNA 1 (non-protein coding) | 7.03E-05 | 2.154550906 | 198.3185432 | 219.5419225 | 457.001564 | 442.2589388 |
| SNORA79 | small nucleolar RNA. H/ACA box 79 | 0.00207024 | 2.152221363 | 26.28648529 | 21.29768372 | 46.93820504 | 55.247457 |
| VAMP1 | vesicle-associated membrane protein 1 (synaptobrevin 1) | 0.00038065 | 2.151657516 | 326.8045261 | 410.8438103 | 786.5351868 | 790.3017522 |
| RNU6-1 | RNA. U6 small nuclear 1 | 7.68E-05 | 2.150464029 | 418.4308549 | 417.4077738 | 854.2222383 | 945.5352547 |
| SERHL2 | serine hydrolase-like 2 | 0.00129453 | 2.148254809 | 17.73546182 | 18.17898802 | 42.69304346 | 34.85191731 |
| RHOBTB3 | Rho-related BTB domain containing 3 | 0.00031013 | 2.145748226 | 28.45707324 | 30.42539577 | 55.63298973 | 71.6558402 |
| TSHR | thyroid stimulating hormone receptor | 0.00446594 | 2.145403762 | 24.43388185 | 23.4600389 | 40.88033234 | 64.53938985 |
| EID2B | EP300 interacting inhibitor of differentiation 2B | 0.00139325 | 2.142169551 | 730.3157608 | 939.107438 | 1863.085142 | 1689.277256 |
| ERAP2 | endoplasmic reticulum aminopeptidase 2 | 0.00374206 | 2.141269964 | 1646.561659 | 1910.990851 | 3723.860454 | 3874.235877 |
| GNPTAB | N-acetylglucosamine-1-phosphate transferase. alpha and beta subunits | 0.00042446 | 2.140783742 | 78.70178739 | 76.79444721 | 145.154374 | 190.8226325 |
| FAM116B | family with sequence similarity 116. member B | 7.88E-05 | 2.139614444 | 774.905154 | 853.4009513 | 1698.33158 | 1782.584923 |
| NUBPL | nucleotide binding protein-like | 0.00014519 | 2.138204559 | 266.4662041 | 314.0450335 | 594.998847 | 643.0080989 |
| LOC100130276 | hypothetical protein LOC100130276 | 0.00198336 | 2.13790778 | 35.94272241 | 52.82593 | 96.68080627 | 89.76267616 |
| LOC391169 | hCG2040210 | 0.00012143 | 2.131145054 | 65.10801274 | 63.56549092 | 137.5351897 | 136.6683764 |
| TLE1 | transducin-like enhancer of split 1 (E(sp1) homolog. Drosophila) | 0.00056765 | 2.128413399 | 15.81347243 | 21.0273794 | 36.35237339 | 41.43731385 |
| BHLHE40 | basic helix-loop-helix family. member e40 | 0.00226823 | 2.126992065 | 90.04638216 | 75.80369269 | 166.100016 | 185.9168258 |
| ISG20 | interferon stimulated exonuclease gene 20kDa | 7.57E-05 | 2.124158253 | 3696.723918 | 4082.065306 | 8169.464112 | 8334.453635 |
| TRAF5 | TNF receptor-associated factor 5 | 0.00075784 | 2.12372037 | 109.6132143 | 120.5420947 | 219.6012507 | 271.3697931 |
| LOC401098 | hypothetical LOC401098 | 0.00072874 | 2.122743888 | 260.5862426 | 304.3293133 | 574.6268675 | 621.8770645 |
| GNA12 | guanine nucleotide binding protein (G protein) alpha 12 | 0.00041311 | 2.119299229 | 46.41748499 | 56.82899641 | 101.7772712 | 116.408676 |
| HCG2P7 | HLA complex group 2 pseudogene 7 | 0.00037916 | 2.114742411 | 1811.196689 | 2243.569493 | 4644.326183 | 3912.887649 |
| NAPSA | napsin A aspartic peptidase | 0.00017053 | 2.114000227 | 132.6204402 | 126.8230342 | 258.0861356 | 291.2419895 |
| HNRNPU | heterogeneous nuclear ribonucleoprotein U (scaffold attachment factor A) | 0.00020793 | 2.112590066 | 268.289594 | 286.230512 | 532.4968713 | 643.6253813 |
| CLN8 | ceroid-lipofuscinosis. neuronal 8 (epilepsy. progressive with mental retardation) | 0.00145421 | 2.112358052 | 21.43426375 | 23.27910555 | 56.99078108 | 39.06657338 |
| RECK | reversion-inducing-cysteine-rich protein with kazal motifs | 0.00045072 | 2.112345969 | 37.26905328 | 44.8447163 | 85.29923498 | 87.42680474 |
| LOC100129362 | hypothetical LOC100129362 | 0.00071356 | 2.101123398 | 1049.420811 | 1243.521307 | 2268.672948 | 2539.417624 |
| PRR5 | proline rich 5 (renal) | 0.00207412 | 2.0991762 | 61.95408484 | 63.83887334 | 130.0712726 | 133.9897443 |
| FCRL2 | Fc receptor-like 2 | 0.00033927 | 2.096551608 | 41.71258641 | 39.7307738 | 93.15937959 | 78.1949434 |
| ALPP | alkaline phosphatase. placental | 0.00052556 | 2.095823637 | 890.8612337 | 1189.79631 | 2109.030208 | 2207.543886 |
| CCDC85A | coiled-coil domain containing 85A | 0.00051999 | 2.083446331 | 125.0396565 | 112.90404 | 258.9840778 | 236.6185695 |
| HSD17B7 | hydroxysteroid (17-beta) dehydrogenase 7 | 0.00094939 | 2.082656668 | 877.4583549 | 1160.273254 | 2326.142152 | 1898.392047 |
| HIST1H4B | histone cluster 1. H4b | 0.00016787 | 2.08190001 | 19.63796379 | 16.41539381 | 37.36606701 | 37.39298259 |
| ZNF512B | zinc finger protein 512B | 0.00039872 | 2.081314656 | 29.21201738 | 33.8147826 | 59.90667362 | 71.42798999 |
| AFF3 | AF4/FMR2 family. member 3 | 0.00018493 | 2.075319106 | 204.1005419 | 189.721158 | 378.164322 | 441.0107141 |
| PNPT1 | polyribonucleotide nucleotidyltransferase 1 | 0.00037307 | 2.074536859 | 836.8852276 | 808.1349707 | 1774.620458 | 1640.161561 |
| GDF11 | growth differentiation factor 11 | 0.00082948 | 2.071431273 | 73.4972503 | 102.7130047 | 183.6505316 | 176.3783977 |
| DCLRE1C | DNA cross-link repair 1C | 9.63E-05 | 2.071083935 | 107.1653025 | 109.5114471 | 213.273618 | 236.0325918 |
| DEM1 | defects in morphology 1 homolog (S. cerevisiae) | 0.00030555 | 2.070980143 | 300.0175575 | 357.1494392 | 625.6003992 | 734.6009678 |
| SYAP1 | synapse associated protein 1 | 0.00017726 | 2.070763145 | 180.6221762 | 166.847023 | 332.3101169 | 388.8721339 |
| FAM175A | family with sequence similarity 175. member A | 0.00010903 | 2.070077437 | 442.8358975 | 462.3471994 | 884.504715 | 991.9369799 |
| LOC100133772 | similar to MCT | 0.00015044 | 2.068320761 | 1408.362722 | 1573.190855 | 3261.686948 | 2905.958721 |
| FKBP14 | FK506 binding protein 14. 22 kDa | 0.00052573 | 2.067797635 | 526.8661922 | 577.2944152 | 1130.27438 | 1150.61458 |
| RNY4 | RNA. Ro-associated Y4 | 0.00442833 | 2.0666422 | 33.45242433 | 26.09854081 | 52.72329538 | 70.72482193 |
| TCL1B | T-cell leukemia/lymphoma 1B | 0.00111387 | 2.065846696 | 42.01658381 | 38.89119218 | 90.78166353 | 76.81924564 |
| ZNF860 | zinc finger protein 860 | 0.00200462 | 2.063135658 | 27.30343759 | 17.84857624 | 47.51859888 | 43.65287499 |
| ST6GAL1 | ST6 beta-galactosamide alpha-2.6-sialyltranferase 1 | 0.00026353 | 2.061853542 | 183.2733728 | 150.500724 | 337.4080108 | 347.5347198 |
| XRCC2 | X-ray repair complementing defective repair in Chinese hamster cells 2 | 0.00021159 | 2.061428744 | 269.9552034 | 291.2419895 | 595.9219038 | 560.6515127 |
| RNU6ATAC | RNA. U6atac small nuclear (U12-dependent splicing) | 0.01676269 | 2.059953249 | 25.01629455 | 21.34018908 | 43.44849792 | 52.13882097 |
| PRKCB | protein kinase C. beta | 0.00338052 | 2.058306347 | 62.15012534 | 47.60829126 | 133.0066275 | 94.24782741 |
| VPS41 | vacuolar protein sorting 41 homolog (S. cerevisiae) | 0.0003579 | 2.05753421 | 198.6281295 | 191.5761376 | 414.0862954 | 389.0321008 |
| RBM9 | RNA binding motif protein 9 | 0.00015257 | 2.057236709 | 83.48761077 | 75.23152994 | 161.7189668 | 164.3726281 |
| CDKN2AIPNL | CDKN2A interacting protein N-terminal like | 0.00062042 | 2.053524201 | 2125.693881 | 2633.135241 | 4414.03554 | 5347.338943 |
| FAM73A | family with sequence similarity 73. member A | 0.00099881 | 2.05108781 | 213.5093241 | 272.2843816 | 508.2356688 | 481.2191949 |
| KLF2 | Kruppel-like factor 2 (lung) | 0.00831841 | 2.046996703 | 38.93237511 | 40.75519982 | 88.66980701 | 74.98121077 |
| FAM63A | family with sequence similarity 63. member A | 0.00024413 | 2.046886456 | 155.6596128 | 170.3003849 | 311.1308724 | 356.9734966 |
| FLJ35390 | hypothetical LOC255031 | 9.51E-05 | 2.045742982 | 761.4996731 | 750.2201901 | 1519.035822 | 1573.956022 |
| PPM1K | protein phosphatase. Mg2+/Mn2+ dependent. 1K | 0.00168357 | 2.042141125 | 74.46770159 | 71.02031172 | 177.4672671 | 124.2807213 |
| RBM38 | RNA binding motif protein 38 | 0.00019637 | 2.041858794 | 140.4627693 | 166.2842766 | 302.7127277 | 321.6867257 |
| DTWD2 | DTW domain containing 2 | 0.00080137 | 2.041523706 | 323.3686584 | 388.8721339 | 825.9093996 | 634.5724217 |
| USP49 | ubiquitin specific peptidase 49 | 0.00202334 | 2.040485684 | 363.9519084 | 510.0708363 | 991.4938678 | 779.5636238 |
| GSTTP2 | glutathione S-transferase theta pseudogene 2 | 0.00079357 | 2.039908957 | 49.0736023 | 64.45063326 | 105.6920514 | 124.5243748 |
| SORBS2 | sorbin and SH3 domain containing 2 | 0.00052265 | 2.039561902 | 24.86251744 | 19.95028584 | 43.96639219 | 46.92963494 |
| DAPP1 | dual adaptor of phosphotyrosine and 3-phosphoinositides | 0.00688286 | 2.039518874 | 40.06774264 | 46.64817403 | 78.92586937 | 98.506663 |
| LRRFIP1 | leucine rich repeat (in FLII) interacting protein 1 | 0.00021212 | 2.037556574 | 351.9094738 | 346.8745848 | 755.4081433 | 670.8742597 |
| ZBTB20 | zinc finger and BTB domain containing 20 | 0.00388279 | 2.037231028 | 30.32946785 | 33.00724944 | 54.81057113 | 75.80369269 |
| SLC2A5 | solute carrier family 2 (facilitated glucose/fructose transporter). member 5 | 8.46E-05 | 2.032770145 | 537.5178905 | 566.2742899 | 1116.108063 | 1126.912172 |
| ZNF394 | zinc finger protein 394 | 0.00130767 | 2.030932068 | 652.9588985 | 623.4427287 | 1447.740261 | 1159.798521 |
| SEMA4D | sema domain. immunoglobulin domain (Ig). transmembrane domain (TM) and short cytoplasmic domain. (semaphorin) 4D | 9.22E-05 | 2.026203055 | 421.2322346 | 424.1623929 | 847.5069782 | 865.5186039 |
| ARRDC3 | arrestin domain containing 3 | 0.02659807 | 2.026049979 | 32.63748991 | 15.54300863 | 53.58744859 | 38.85877163 |
| LOC728809 | hypothetical LOC728809 | 0.00077149 | 2.02555703 | 2058.467179 | 2313.937071 | 4406.723525 | 4434.744841 |
| LOC100129387 | hypothetical LOC100129387 | 0.000531 | 2.024653501 | 42.63116787 | 44.80063709 | 79.60360146 | 98.35130374 |
| PRICKLE1 | prickle homolog 1 (Drosophila) | 0.00229952 | 2.023215846 | 18.82117528 | 21.10685076 | 42.99979859 | 37.81709745 |
| ARHGAP24 | Rho GTPase activating protein 24 | 0.00017248 | 2.018122967 | 45.51837995 | 51.09251053 | 98.66072054 | 96.00525529 |
| MAP3K5 | mitogen-activated protein kinase kinase kinase 5 | 0.00017951 | 2.015103089 | 36.51213671 | 31.23067795 | 67.46754696 | 68.63067625 |
| LOC653506 | similar to meteorin. glial cell differentiation regulator-like | 0.00108001 | 2.014782299 | 78.86085826 | 58.16650898 | 138.9340185 | 134.0238701 |
| PSEN2 | presenilin 2 (Alzheimer disease 4) | 0.00851255 | 2.012656088 | 27.3514358 | 23.12038678 | 41.74460057 | 61.36405592 |
| TMEM8B | transmembrane protein 8B | 0.00014544 | 2.005311168 | 92.43263744 | 103.1169194 | 191.616218 | 200.0260488 |
| ZNF577 | zinc finger protein 577 | 0.00022189 | 2.004995348 | 141.282339 | 137.2557704 | 278.3782974 | 280.0334123 |
| RINL | Ras and Rab interactor-like | 0.00030941 | 2.00343566 | 85.19945767 | 72.95541046 | 165.7917622 | 150.4811798 |
| OPA3 | optic atrophy 3 (autosomal recessive. with chorea and spastic paraplegia) | 0.0001047 | 2.003379404 | 68.23623118 | 69.47281967 | 141.6236816 | 134.3446842 |
| IL10RA | interleukin 10 receptor. alpha | 0.00017173 | 2.002220723 | 63.43109881 | 59.15570095 | 115.727821 | 129.9825245 |
| PLEKHB2 | pleckstrin homology domain containing. family B (evectins) member 2 | 0.00117787 | 2.001276453 | 41.93218534 | 39.38230896 | 94.1515 | 70.24826447 |
| IVD | isovaleryl-CoA dehydrogenase | 0.00202616 | 1.999615135 | 43.83199363 | 46.01978716 | 85.77791079 | 94.02713344 |
| ZNF14 | zinc finger protein 14 | 0.00022996 | 1.998486563 | 1355.118715 | 1542.221681 | 2766.597816 | 3017.036628 |
| FAM58A | family with sequence similarity 58. member A | 0.00132261 | 1.99724032 | 160.2449645 | 145.5419976 | 271.1358745 | 343.1202809 |
| C13orf15 | chromosome 13 open reading frame 15 | 0.00019895 | 1.995611474 | 184.6843901 | 162.0476369 | 360.0766729 | 331.0014447 |
| SNORA12 | small nucleolar RNA. H/ACA box 12 | 0.00029274 | 1.993440785 | 327.2941519 | 291.6248328 | 579.8969028 | 654.0615779 |
| CSF2RA | colony stimulating factor 2 receptor. alpha. low-affinity (granulocyte-macrophage) | 0.00037058 | 1.993290687 | 689.2858774 | 821.2790549 | 1483.590484 | 1516.063402 |
| SYNJ2BP | synaptojanin 2 binding protein | 0.0002638 | 1.99263077 | 19.63651187 | 23.04869769 | 40.32096738 | 44.56905825 |
| TCEB3 | transcription elongation factor B (SIII). polypeptide 3 (110kDa. elongin A) | 0.00017164 | 1.991674333 | 296.3180458 | 286.6506118 | 542.702505 | 620.8487352 |
| HIATL2 | hippocampus abundant transcript-like 2 | 0.00041748 | 1.990860717 | 811.8496332 | 936.9208614 | 1660.314836 | 1815.807534 |
| AIRE | autoimmune regulator | 0.00012183 | 1.990342305 | 2102.487399 | 2116.207347 | 4087.605444 | 4311.994505 |
| ZNF682 | zinc finger protein 682 | 0.00137158 | 1.988535411 | 950.0778072 | 1216.705072 | 1939.143729 | 2357.225606 |
| STXBP6 | syntaxin binding protein 6 (amisyn) | 0.00044604 | 1.987063334 | 367.6092849 | 383.624121 | 770.5247047 | 722.6520347 |
| C14orf153 | chromosome 14 open reading frame 153 | 0.00625686 | 1.98588096 | 440.5959852 | 671.5774784 | 918.2882925 | 1270.761462 |
| DEF6 | differentially expressed in FDCP 6 homolog (mouse) | 0.00018673 | 1.981620513 | 178.9325767 | 179.0458795 | 357.2772994 | 352.1188722 |
| SNORA57 | small nucleolar RNA. H/ACA box 57 | 0.00084796 | 1.980309211 | 46.68379376 | 42.69304346 | 92.95670445 | 84.08306879 |
| MIDN | midnolin | 0.00017104 | 1.975419482 | 370.2326666 | 363.6351726 | 679.4568537 | 773.2098897 |
| HSPA1B | heat shock 70kDa protein 1B | 0.00044035 | 1.97450942 | 61.29234378 | 59.53441329 | 122.4575594 | 116.1735143 |
| PLA2G15 | phospholipase A2. group XV | 0.00013324 | 1.971096455 | 113.2118135 | 115.5651513 | 235.7774463 | 215.591748 |
| RGS13 | regulator of G-protein signaling 13 | 0.00068068 | 1.970920897 | 18.78126146 | 22.38128761 | 43.63362026 | 37.42199768 |
| CD1D | CD1d molecule | 0.00045983 | 1.970679287 | 37.84019708 | 41.02406094 | 86.36262846 | 69.80685792 |
| PFKFB4 | 6-phosphofructo-2-kinase/fructose-2.6-biphosphatase 4 | 0.00051299 | 1.970232335 | 48.03424137 | 56.65644003 | 96.65451651 | 109.2981851 |
| LFNG | LFNG O-fucosylpeptide 3-beta-N-acetylglucosaminyltransferase | 0.00018694 | 1.969237047 | 77.1633958 | 80.8710444 | 151.1012312 | 160.1520032 |
| PHF14 | PHD finger protein 14 | 0.00010477 | 1.969169054 | 120.207237 | 119.787265 | 233.5052983 | 239.1170416 |
| TMEM159 | transmembrane protein 159 | 0.00152379 | 1.9644751 | 60.08561302 | 69.80685792 | 147.5842521 | 109.678531 |
| C11orf63 | chromosome 11 open reading frame 63 | 0.00476752 | 1.963001893 | 36.13424493 | 54.62037005 | 86.78287977 | 87.6356882 |
| DENND5B | DENN/MADD domain containing 5B | 0.00444913 | 1.962203397 | 39.41132975 | 29.36001619 | 55.06323594 | 80.91027835 |
| CD24 | CD24 molecule | 0.00011846 | 1.961520575 | 146.6090915 | 149.3422112 | 282.2332588 | 298.483979 |
| HLA-DMA | major histocompatibility complex. class II. DM alpha | 0.00014861 | 1.961152321 | 670.8742597 | 721.6338263 | 1331.556574 | 1398.366595 |
| LOC730202 | hypothetical protein LOC730202 | 0.00147124 | 1.960593064 | 25.37262784 | 23.99703922 | 41.9921817 | 55.73520407 |
| OSM | oncostatin M | 0.00029076 | 1.960510883 | 22.05801927 | 22.42075891 | 47.69125162 | 39.85810207 |
| TDP1 | tyrosyl-DNA phosphodiesterase 1 | 0.00290329 | 1.959405941 | 387.1879587 | 549.2677829 | 882.2798804 | 925.440357 |
| TJP2 | tight junction protein 2 (zona occludens 2) | 0.00022299 | 1.957892783 | 84.39139186 | 81.17918201 | 150.4601037 | 174.5417294 |
| SERTAD1 | SERTA domain containing 1 | 0.00062258 | 1.957593732 | 62.32206101 | 66.87586969 | 117.8825764 | 135.4898501 |
| RHBDF1 | rhomboid 5 homolog 1 (Drosophila) | 0.00030153 | 1.954277531 | 55.02627173 | 50.77991436 | 111.1074995 | 96.04862599 |
| CHRNA5 | cholinergic receptor. nicotinic. alpha 5 | 0.00170149 | 1.950676722 | 323.2441533 | 394.5733913 | 604.0347273 | 803.4653788 |
| LOC100128288 | hypothetical protein LOC100128288 | 0.01021417 | 1.949412648 | 451.058212 | 730.3157608 | 940.5628808 | 1330.953831 |
| SLTM | SAFB-like. transcription modulator | 0.00139953 | 1.948933014 | 45.10572765 | 51.70353891 | 109.4147562 | 80.95988664 |
| KLHL28 | kelch-like 28 (Drosophila) | 0.00611274 | 1.948106596 | 69.31488868 | 110.8015817 | 154.7416024 | 188.3609334 |
| RNY1 | RNA. Ro-associated Y1 | 0.00037613 | 1.947901238 | 118.2888233 | 97.00786898 | 202.9008177 | 214.5856845 |
| PLA2G2D | phospholipase A2. group IID | 0.00061358 | 1.947692749 | 77.49848568 | 94.93354878 | 155.2657254 | 179.7538622 |
| ARL16 | ADP-ribosylation factor-like 16 | 0.0011067 | 1.94718952 | 1388.636575 | 1812.350825 | 3036.478613 | 3142.513049 |
| MAPK10 | mitogen-activated protein kinase 10 | 0.00275415 | 1.943562237 | 18.60798946 | 18.41341986 | 29.9093467 | 43.27368552 |
| C3orf34 | chromosome 3 open reading frame 34 | 0.00748198 | 1.942780309 | 693.308675 | 876.8756112 | 1393.586764 | 1646.561659 |
| SDR16C5 | short chain dehydrogenase/reductase family 16C. member 5 | 0.00275941 | 1.941857233 | 49.58916977 | 50.12741298 | 107.6519432 | 87.07126408 |
| TOB1 | transducer of ERBB2. 1 | 0.00145778 | 1.941331545 | 111.3307229 | 86.63709952 | 172.1862745 | 211.1153573 |
| POLR2J4 | polymerase (RNA) II (DNA directed) polypeptide J4. pseudogene | 0.0023677 | 1.938493326 | 33.87687039 | 42.6654046 | 64.2584925 | 84.52345448 |
| PPA2 | pyrophosphatase (inorganic) 2 | 0.00070508 | 1.938005792 | 1030.676263 | 1327.365984 | 2222.787206 | 2311.66667 |
| C6orf170 | chromosome 6 open reading frame 170 | 0.00443121 | 1.937758476 | 67.65087974 | 90.8299178 | 145.2573308 | 158.8413613 |
| AIDA | axin interactor. dorsalization associated | 0.00029473 | 1.937015458 | 682.3343423 | 624.5724922 | 1354.430373 | 1180.564098 |
| C14orf79 | chromosome 14 open reading frame 79 | 0.00157546 | 1.93698099 | 30.52497217 | 39.55534524 | 73.20856562 | 61.87985327 |
| OCIAD1 | OCIA domain containing 1 | 0.00019443 | 1.93612233 | 1375.05511 | 1471.284555 | 2726.781549 | 2781.198722 |
| ABHD15 | abhydrolase domain containing 15 | 0.00441006 | 1.934786292 | 70.20471049 | 43.94876248 | 105.8618829 | 109.1036532 |
| BMS1P5 | BMS1 pseudogene 5 | 0.00111043 | 1.934271209 | 792.2700722 | 1012.186434 | 1757.145653 | 1707.500092 |
| KLRC2 | killer cell lectin-like receptor subfamily C. member 2 | 0.00226033 | 1.933514721 | 82.288354 | 56.68946539 | 139.1220959 | 125.3544024 |
| SEMA3E | sema domain. immunoglobulin domain (Ig). short basic domain. secreted. (semaphorin) 3E | 0.00021228 | 1.933325868 | 287.3124334 | 299.6815571 | 572.8210101 | 561.8309011 |
| PLEKHA9 | pleckstrin homology domain containing. family A (phosphoinositide binding specific) member 9 | 0.00021731 | 1.93317069 | 201.7264924 | 204.3978875 | 385.5736455 | 399.6431764 |
| ATP13A2 | ATPase type 13A2 | 0.00428974 | 1.930266744 | 38.2101237 | 41.61297227 | 94.87554277 | 62.44354691 |
| CD69 | CD69 molecule | 0.00041233 | 1.9287222 | 185.3596082 | 189.8839235 | 374.846791 | 349.2921786 |
| MAP6D1 | MAP6 domain containing 1 | 0.00400944 | 1.927735829 | 60.00507685 | 38.11307126 | 94.503669 | 89.93076999 |
| PRRG4 | proline rich Gla (G-carboxyglutamic acid) 4 (transmembrane) | 0.00475224 | 1.927277227 | 50.56207483 | 58.65059368 | 131.5232819 | 83.74965791 |
| IL10 | interleukin 10 | 0.00136474 | 1.926445533 | 1308.338535 | 1814.079343 | 2955.127182 | 2980.668676 |
| PIK3CG | phosphoinositide-3-kinase. catalytic. gamma polypeptide | 0.0003508 | 1.925945902 | 80.21114746 | 84.16501569 | 155.3615618 | 161.1799169 |
| ZMAT3 | zinc finger. matrin type 3 | 0.00108213 | 1.92444261 | 1602.052037 | 1782.584923 | 2980.668676 | 3548.322398 |
| NDUFB10 | NADH dehydrogenase (ubiquinone) 1 beta subcomplex. 10. 22kDa | 0.00084328 | 1.921091803 | 1008.842112 | 879.4537402 | 1920.681392 | 1704.814427 |
| CXCR7 | chemokine (C-X-C motif) receptor 7 | 0.00012813 | 1.920407985 | 398.4831698 | 398.6341445 | 767.7476124 | 763.0500233 |
| TRIM13 | tripartite motif-containing 13 | 0.00079899 | 1.918225631 | 154.1722327 | 135.6434286 | 292.6078431 | 262.9773454 |
| CD24 | CD24 molecule | 0.00019151 | 1.917945704 | 1537.283183 | 1619.941686 | 2893.344397 | 3166.10813 |
| SELO | selenoprotein O | 0.0011152 | 1.916333281 | 96.40206636 | 101.3097319 | 180.1921996 | 199.0414867 |
| SNAPC1 | small nuclear RNA activating complex. polypeptide 1. 43kDa | 0.0009304 | 1.914569507 | 101.1282401 | 122.0372409 | 193.7882174 | 233.4423986 |
| RHOC | ras homolog gene family. member C | 0.00018171 | 1.913779931 | 285.6109936 | 271.3697931 | 535.4861919 | 530.1174975 |
| ITPK1AS | ITPK1 antisense RNA (non-protein coding) | 0.00027423 | 1.913537789 | 1522.769006 | 1698.929545 | 3244.104425 | 2920.039046 |
| CDKL1 | cyclin-dependent kinase-like 1 (CDC2-related kinase) | 0.02443974 | 1.912327192 | 24.18939553 | 21.05524956 | 35.37119939 | 52.65747421 |
| ZDHHC14 | zinc finger. DHHC-type containing 14 | 0.0037738 | 1.907476799 | 123.6824581 | 158.3142553 | 262.3609133 | 271.5485729 |
| EVI5 | ecotropic viral integration site 5 | 0.00028966 | 1.907220574 | 80.95988664 | 72.40067911 | 140.5908496 | 151.6552056 |
| UMODL1 | uromodulin-like 1 | 0.00051516 | 1.906951928 | 36.43773581 | 40.82791237 | 67.67350014 | 79.94096887 |
| PLEKHA1 | pleckstrin homology domain containing. family A (phosphoinositide binding specific) member 1 | 0.00275326 | 1.905400867 | 73.73979937 | 51.28095429 | 113.1434332 | 121.3392802 |
| PCYOX1 | prenylcysteine oxidase 1 | 0.01154693 | 1.903696616 | 26.09550402 | 26.1683692 | 42.11554748 | 58.76183819 |
| RASSF6 | Ras association (RalGDS/AF-6) domain family member 6 | 0.00070217 | 1.902089576 | 109.8100987 | 108.4816443 | 216.1206366 | 199.4179441 |
| C5orf28 | chromosome 5 open reading frame 28 | 0.0089252 | 1.902019659 | 738.0939027 | 1254.06292 | 1983.826799 | 1687.940736 |
| KIAA1751 | KIAA1751 | 0.00551861 | 1.899385116 | 395.1489652 | 451.4135394 | 878.3739694 | 732.6255052 |
| FUCA1 | fucosidase. alpha-L- 1. tissue | 0.00018554 | 1.897756371 | 224.5348336 | 238.3254814 | 447.1495818 | 431.0049835 |
| CCDC125 | coiled-coil domain containing 125 | 0.00025105 | 1.895445613 | 403.6133221 | 456.9248568 | 798.694796 | 829.5681649 |
| SUSD3 | sushi domain containing 3 | 0.00042975 | 1.894708955 | 897.6915754 | 967.6373307 | 1744.818211 | 1787.205919 |
| VPREB1 | pre-B lymphocyte 1 | 0.00092857 | 1.893635304 | 83.82214744 | 83.44852714 | 145.5419976 | 172.3383026 |
| RSPH3 | radial spoke 3 homolog (Chlamydomonas) | 0.00401024 | 1.893409873 | 20.09331717 | 29.60297006 | 43.03647894 | 49.54952191 |
| PCYOX1 | prenylcysteine oxidase 1 | 0.01272989 | 1.891212719 | 28.62124472 | 36.66424955 | 46.50576388 | 80.70590184 |
| ABTB1 | ankyrin repeat and BTB (POZ) domain containing 1 | 0.00021998 | 1.890177791 | 89.555133 | 100.0122278 | 179.7305897 | 178.0438173 |
| ZNF281 | zinc finger protein 281 | 0.0005835 | 1.889963779 | 120.0484218 | 106.01905 | 234.1883302 | 194.1252712 |
| SHCBP1 | SHC SH2-domain binding protein 1 | 0.00018626 | 1.88984453 | 3596.396416 | 3912.887649 | 7102.5499 | 7076.243953 |
| POFUT1 | protein O-fucosyltransferase 1 | 0.00098745 | 1.887018554 | 1900.585773 | 2385.257633 | 3754.91689 | 4299.072608 |
| FAM172A | family with sequence similarity 172. member A | 0.00118355 | 1.88490278 | 23.15896294 | 19.93527463 | 39.55534524 | 41.46809272 |
| TJP2 | tight junction protein 2 (zona occludens 2) | 0.00605547 | 1.882673929 | 36.66785466 | 28.30620334 | 56.79184959 | 64.7785657 |
| ZNF573 | zinc finger protein 573 | 0.00069566 | 1.88249266 | 74.2122681 | 58.68321856 | 121.2670833 | 127.2662599 |
| SCARNA13 | small Cajal body-specific RNA 13 | 0.00099265 | 1.882460328 | 110.0079575 | 90.28949561 | 205.7403028 | 171.077785 |
| PTPLAD2 | protein tyrosine phosphatase-like A domain containing 2 | 0.00111472 | 1.88125525 | 165.3113349 | 212.8532834 | 368.1205718 | 338.2893678 |
| AGPHD1 | aminoglycoside phosphotransferase domain containing 1 | 0.08105082 | 1.88021976 | 31.13494071 | 53.9073695 | 66.48265884 | 89.24934316 |
| PI16 | peptidase inhibitor 16 | 0.00714654 | 1.878681298 | 23.04298918 | 31.30784833 | 43.06659663 | 59.1231696 |
| MIR2116 | microRNA 2116 | 0.00041467 | 1.872222464 | 18.76723705 | 20.77649154 | 34.37095142 | 39.76453432 |
| ZNF483 | zinc finger protein 483 | 0.00286186 | 1.872127604 | 203.3704297 | 276.4025129 | 479.8910157 | 410.5424483 |
| ORAI2 | ORAI calcium release-activated calcium modulator 2 | 0.02463421 | 1.869152398 | 31.54184307 | 35.13812981 | 88.26097442 | 43.87189727 |
| ERAP2 | endoplasmic reticulum aminopeptidase 2 | 0.00190763 | 1.868209904 | 286.9418441 | 318.8294515 | 502.560324 | 635.3535465 |
| UNC119 | unc-119 homolog (C. elegans) | 0.01090281 | 1.868020551 | 317.1923607 | 200.0260488 | 503.7972318 | 439.4574166 |
| SPTLC1 | serine palmitoyltransferase. long chain base subunit 1 | 0.00149075 | 1.865573616 | 793.0271858 | 1040.759079 | 1615.699067 | 1777.880603 |
| C2CD2 | C2 calcium-dependent domain containing 2 | 0.00042459 | 1.863400832 | 127.2054523 | 139.6834741 | 230.5622541 | 267.5932261 |
| FGD3 | FYVE. RhoGEF and PH domain containing 3 | 0.00119303 | 1.863098262 | 665.400855 | 640.5717157 | 1236.448951 | 1196.592962 |
| AHR | aryl hydrocarbon receptor | 0.00032148 | 1.862829957 | 1145.855514 | 1301.615206 | 2333.594806 | 2217.856536 |
| CTSH | cathepsin H | 0.00054668 | 1.862560549 | 149.9546316 | 142.809337 | 294.4826426 | 252.2769578 |
| N4BP2 | NEDD4 binding protein 2 | 0.00806016 | 1.862253032 | 319.5018663 | 535.0175988 | 774.6971267 | 765.2210388 |
| UPF2 | UPF2 regulator of nonsense transcripts homolog (yeast) | 0.00214399 | 1.862112676 | 171.856801 | 212.4705145 | 385.0135229 | 328.8526322 |
| PHACTR1 | phosphatase and actin regulator 1 | 0.0006482 | 1.861915362 | 37.89763868 | 45.31528636 | 82.1665011 | 72.45726818 |
| MTMR10 | myotubularin related protein 10 | 0.00981624 | 1.861103906 | 33.67836498 | 45.47922805 | 70.54288562 | 75.2059331 |
| FAM119A | family with sequence similarity 119. member A | 0.00095375 | 1.859094626 | 1170.031749 | 1061.236015 | 1998.659772 | 2147.206169 |
| HSPA7 | heat shock 70kDa protein 7 (HSP70B) | 0.0019973 | 1.858720713 | 24.11851011 | 24.69924927 | 39.53012353 | 52.06361683 |
| SNORD43 | small nucleolar RNA. C/D box 43 | 0.0171382 | 1.858421104 | 18.23449709 | 19.85249976 | 31.59223505 | 39.57463288 |
| SP110 | SP110 nuclear body protein | 0.00037616 | 1.857277573 | 125.6368221 | 136.3783809 | 259.6839054 | 227.5993764 |
| C8orf37 | chromosome 8 open reading frame 37 | 0.00216561 | 1.856113671 | 1823.459766 | 2425.700845 | 4254.351333 | 3581.864989 |
| PPP2R3A | protein phosphatase 2. regulatory subunit B''. alpha | 0.0026257 | 1.855901926 | 33.41708119 | 30.54835389 | 55.7904085 | 63.02412584 |
| TNFAIP8L1 | tumor necrosis factor. alpha-induced protein 8-like 1 | 0.00030728 | 1.855182124 | 26.41797221 | 28.01459137 | 51.91554932 | 49.06360091 |
| SSPN | sarcospan (Kras oncogene-associated gene) | 0.0009758 | 1.854790912 | 17.15440824 | 17.00725137 | 28.07354873 | 35.75217571 |
| C14orf145 | chromosome 14 open reading frame 145 | 0.00060188 | 1.854331672 | 215.7373171 | 203.8850043 | 390.3287447 | 387.4849646 |
| ZNF549 | zinc finger protein 549 | 0.00126523 | 1.851026403 | 1573.956022 | 1931.190737 | 3398.548934 | 3064.428289 |
| ABHD12 | abhydrolase domain containing 12 | 0.00057081 | 1.847516241 | 17.75806655 | 16.19309077 | 33.15230821 | 29.60657623 |
| SCARNA16 | small Cajal body-specific RNA 16 | 0.00120213 | 1.847170655 | 40.73426041 | 44.83382941 | 80.05497146 | 77.8379527 |
| POLR2J4 | polymerase (RNA) II (DNA directed) polypeptide J4. pseudogene | 0.00621777 | 1.844467537 | 20.46881495 | 19.53270606 | 45.37354675 | 29.97743141 |
| ASXL2 | additional sex combs like 2 (Drosophila) | 0.00039579 | 1.843526805 | 190.0130175 | 213.3373601 | 358.5615293 | 384.2248884 |
| CREB1 | cAMP responsive element binding protein 1 | 0.00232453 | 1.843217818 | 461.8699817 | 527.7269646 | 943.1240373 | 878.0384384 |
| CATSPER2 | cation channel. sperm associated 2 | 0.00267264 | 1.842818558 | 783.8685765 | 932.7232861 | 1358.185677 | 1828.10898 |
| PRR5 | proline rich 5 (renal) | 0.00176219 | 1.840759521 | 32.68035316 | 43.28051067 | 64.87850741 | 73.87072794 |
| SDCCAG1 | serologically defined colon cancer antigen 1 | 0.00230259 | 1.840652811 | 62.40183585 | 55.28888341 | 112.958153 | 103.4811772 |
| C17orf79 | chromosome 17 open reading frame 79 | 0.00133091 | 1.839691169 | 397.1177293 | 345.3073216 | 764.1176985 | 607.3718385 |
| ABCC9 | ATP-binding cassette. sub-family C (CFTR/MRP). member 9 | 0.03302234 | 1.839127616 | 23.56571834 | 18.52372941 | 54.89210353 | 26.89818454 |
| ICA1L | islet cell autoantigen 1.69kDa-like | 0.04847778 | 1.839098248 | 30.83129898 | 38.18936116 | 68.90538785 | 57.79508357 |
| CCDC138 | coiled-coil domain containing 138 | 0.00067299 | 1.838706158 | 145.0572989 | 137.9461777 | 241.2314274 | 280.4400475 |
| EXTL2 | exostoses (multiple)-like 2 | 0.03613728 | 1.837694149 | 39.56157352 | 57.67623673 | 83.81037754 | 91.94309963 |
| HLA-DMB | major histocompatibility complex. class II. DM beta | 0.00021912 | 1.83756975 | 401.1913451 | 377.4267728 | 713.3913085 | 716.711068 |
| VPREB3 | pre-B lymphocyte 3 | 0.00138665 | 1.837277294 | 547.767756 | 531.7587403 | 926.5066411 | 1061.236015 |
| POLR1B | polymerase (RNA) I polypeptide B. 128kDa | 0.0028529 | 1.836315328 | 39.44807597 | 37.48426923 | 81.05407947 | 61.51691039 |
| C19orf51 | chromosome 19 open reading frame 51 | 0.00053381 | 1.834383928 | 130.2191867 | 119.3254139 | 234.9674431 | 222.5259738 |
| CNR1 | cannabinoid receptor 1 (brain) | 0.00330931 | 1.834200667 | 111.5143949 | 81.57328345 | 185.1595786 | 165.2823147 |
| ENO3 | enolase 3 (beta. muscle) | 0.00032106 | 1.829714924 | 89.28971992 | 86.41654173 | 162.9779556 | 158.5025809 |
| PAPD5 | PAP associated domain containing 5 | 0.00247873 | 1.82848117 | 132.0703506 | 147.9726546 | 275.9723335 | 236.7566762 |
| BLZF1 | basic leucine zipper nuclear factor 1 | 0.00779319 | 1.827480911 | 130.7419053 | 182.8324979 | 239.5219911 | 333.2947765 |
| CRCP | CGRP receptor component | 0.00375545 | 1.822156695 | 1245.164522 | 1805.550096 | 2583.41342 | 2889.44087 |
| TAGAP | T-cell activation RhoGTPase activating protein | 0.00049632 | 1.821359375 | 84.93846043 | 84.17072329 | 148.7663605 | 159.4233741 |
| PTPN12 | protein tyrosine phosphatase. non-receptor type 12 | 0.01741582 | 1.81933717 | 84.891634 | 59.19237775 | 163.3941027 | 101.7936498 |
| C7orf55 | chromosome 7 open reading frame 55 | 0.00403602 | 1.81816455 | 283.6310893 | 312.1469997 | 597.185829 | 490.0832681 |
| AK1 | adenylate kinase 1 | 0.00065483 | 1.817525331 | 49.06360091 | 59.61077086 | 97.00786898 | 99.59513796 |
| FBXW7 | F-box and WD repeat domain containing 7 | 0.00244549 | 1.816997274 | 65.62212883 | 83.95237405 | 138.5081841 | 131.3156274 |
| SLC27A4 | solute carrier family 27 (fatty acid transporter). member 4 | 0.03133582 | 1.814444379 | 19.00844101 | 17.97052778 | 23.97227706 | 46.91215271 |
| C21orf58 | chromosome 21 open reading frame 58 | 0.00127072 | 1.813475097 | 586.249014 | 685.0843245 | 1054.196397 | 1252.932922 |
| C7orf41 | chromosome 7 open reading frame 41 | 0.00107864 | 1.813340983 | 112.5038505 | 94.84652644 | 200.9076771 | 174.6430224 |
| TMEM19 | transmembrane protein 19 | 0.02010697 | 1.812279355 | 39.8032465 | 23.55303281 | 66.24082092 | 46.48254661 |
| TLR6 | toll-like receptor 6 | 0.01184128 | 1.81141 | 59.56633028 | 44.95915228 | 86.45668786 | 101.6374796 |
| TSPAN17 | tetraspanin 17 | 0.00076628 | 1.809391001 | 66.45646764 | 55.30884614 | 108.9334225 | 110.4677619 |
| EIF2C2 | eukaryotic translation initiation factor 2C. 2 | 0.00159608 | 1.807907942 | 145.1883029 | 180.0985733 | 299.353058 | 285.5030994 |
| SERPINA10 | serpin peptidase inhibitor. clade A (alpha-1 antiproteinase. antitrypsin). member 10 | 0.00587359 | 1.807411783 | 84.44443439 | 60.80840293 | 135.0159673 | 124.2406499 |
| RNPC3 | RNA-binding region (RNP1. RRM) containing 3 | 0.00086188 | 1.806378418 | 94.11468263 | 80.81609352 | 167.5435689 | 148.1306557 |
| CCR6 | chemokine (C-C motif) receptor 6 | 0.00428201 | 1.805108197 | 24.66752355 | 24.81910525 | 36.78808965 | 54.22641744 |
| PPIL3 | peptidylprolyl isomerase (cyclophilin)-like 3 | 0.00224347 | 1.802015394 | 16.79800803 | 22.97866404 | 34.7330109 | 36.08752692 |
| TMEM17 | transmembrane protein 17 | 0.00193599 | 1.801811603 | 981.6975028 | 1270.040952 | 2003.898342 | 2019.940067 |
| MARCH1 | membrane-associated ring finger (C3HC4) 1 | 0.0029262 | 1.801691905 | 31.51949892 | 44.23677446 | 65.66213711 | 68.93008252 |
| NDE1 | nudE nuclear distribution gene E homolog 1 (A. nidulans) | 0.00093973 | 1.8013719 | 277.4750158 | 230.256893 | 477.3990816 | 434.2718847 |
| LOC151162 | hypothetical LOC151162 | 0.00129569 | 1.79961937 | 708.8907928 | 781.9526215 | 1188.239564 | 1510.835166 |
| ADCK2 | aarF domain containing kinase 2 | 0.00114491 | 1.799521623 | 281.4400878 | 299.1010989 | 462.837929 | 588.9645612 |
| LOC100132774 | hypothetical LOC100132774 | 0.00783387 | 1.797731072 | 20.27816531 | 17.96915232 | 30.44237929 | 38.68361979 |
| SNORD11 | small nucleolar RNA. C/D box 11 | 0.00061916 | 1.796181509 | 23.30203473 | 23.15444848 | 38.5919777 | 45.10572765 |
| FKTN | fukutin | 0.00104286 | 1.795306529 | 3894.074699 | 4063.350786 | 6726.132284 | 7582.290346 |
| PTCRA | pre T-cell antigen receptor alpha | 0.00102278 | 1.795293456 | 54.65912584 | 47.39929954 | 98.31077421 | 84.93846043 |
| SNORA24 | small nucleolar RNA. H/ACA box 24 | 0.00389117 | 1.793710332 | 168.4382334 | 141.1687336 | 290.0109708 | 263.7967187 |
| SDHAP1 | succinate dehydrogenase complex. subunit A. flavoprotein pseudogene 1 | 0.00352444 | 1.792848656 | 1084.213864 | 1527.999467 | 2425.700845 | 2195.271197 |
| TDRD1 | tudor domain containing 1 | 0.00041119 | 1.792135071 | 69.01393771 | 66.24082092 | 121.6345758 | 120.7110274 |
| LOC100131096 | hypothetical LOC100131096 | 0.00102168 | 1.79087715 | 86.25431681 | 82.59652911 | 151.3412874 | 150.9790901 |
| IQCG | IQ motif containing G | 0.0298708 | 1.790738428 | 25.20036613 | 26.7868429 | 40.39518561 | 53.58744859 |
| LRRC37BP1 | leucine rich repeat containing 37B pseudogene 1 | 0.00085917 | 1.790053536 | 841.0572993 | 980.0620333 | 1666.265442 | 1585.137726 |
| BLK | B lymphoid tyrosine kinase | 0.00042736 | 1.789418252 | 1592.918838 | 1458.458874 | 2822.632561 | 2635.464764 |
| WDR74 | WD repeat domain 74 | 0.01133055 | 1.788375575 | 61.04046072 | 93.64917455 | 147.4858809 | 123.9620557 |
| RHOH | ras homolog gene family. member H | 0.00038252 | 1.787607436 | 217.2701302 | 202.0792834 | 358.4143678 | 391.4539788 |
| MARCH1 | membrane-associated ring finger (C3HC4) 1 | 0.0009851 | 1.786450922 | 87.93099217 | 88.7367771 | 144.8576293 | 171.9041774 |
| LRWD1 | leucine-rich repeats and WD repeat domain containing 1 | 0.00026979 | 1.786108432 | 229.6575867 | 220.8946377 | 398.283983 | 406.3392457 |
| DYRK2 | dual-specificity tyrosine-(Y)-phosphorylation regulated kinase 2 | 0.00096765 | 1.784520664 | 132.1686683 | 145.4736601 | 249.1976904 | 245.7038884 |
| SULT1A1 | sulfotransferase family. cytosolic. 1A. phenol-preferring. member 1 | 0.01882954 | 1.78289231 | 76.79444721 | 82.99757068 | 162.5688512 | 124.6258365 |
| TELO2 | TEL2. telomere maintenance 2. homolog (S. cerevisiae) | 0.06958729 | 1.782721693 | 36.83723562 | 74.55158837 | 98.43176395 | 88.66980701 |
| CD68 | CD68 molecule | 0.00154944 | 1.782647762 | 171.8274968 | 203.1461642 | 360.9402428 | 307.3244124 |
| PDE4B | phosphodiesterase 4B. cAMP-specific | 0.0043938 | 1.781449412 | 37.00457272 | 33.05220027 | 55.87117847 | 69.47281967 |
| PIGX | phosphatidylinositol glycan anchor biosynthesis. class X | 0.00042299 | 1.780800349 | 81.37824012 | 88.1058553 | 153.755307 | 147.8813527 |
| NRG4 | neuregulin 4 | 0.00333266 | 1.779705108 | 75.6669475 | 93.32280609 | 170.9557446 | 130.8297159 |
| CCRN4L | CCR4 carbon catabolite repression 4-like (S. cerevisiae) | 0.00159841 | 1.778399189 | 62.19300427 | 52.12989267 | 98.16640328 | 104.4537391 |
| SYTL2 | synaptotagmin-like 2 | 0.0064064 | 1.778110724 | 49.87071529 | 36.71147472 | 67.50959698 | 85.74316596 |
| PMEPA1 | prostate transmembrane protein. androgen induced 1 | 0.03629724 | 1.778078248 | 73.66818282 | 46.45447634 | 96.09806347 | 112.5886542 |
| DBNDD2 | dysbindin (dystrobrevin binding protein 1) domain containing 2 | 0.00110119 | 1.775771434 | 60.1109599 | 51.45882553 | 101.2205957 | 96.36487771 |
| LOC100129269 | hypothetical LOC100129269 | 0.00302325 | 1.775650808 | 169.257809 | 237.0933833 | 364.6557776 | 346.9765914 |
| CD83 | CD83 molecule | 0.00040697 | 1.774940554 | 675.2215424 | 649.9071989 | 1116.990675 | 1237.700922 |
| ZNF480 | zinc finger protein 480 | 0.00648955 | 1.774723454 | 51.28095429 | 33.82341761 | 74.44119686 | 73.38741947 |
| C9orf80 | chromosome 9 open reading frame 80 | 0.00504898 | 1.774215322 | 1918.618894 | 2699.853766 | 4143.290714 | 3935.466347 |
| CTSB | cathepsin B | 0.00268205 | 1.773711522 | 131.7735862 | 152.3967024 | 226.9800743 | 278.3441997 |
| FAM92B | family with sequence similarity 92. member B | 0.00848096 | 1.773399182 | 22.71481752 | 34.74331332 | 54.68472269 | 45.38658206 |
| TUFT1 | tuftelin 1 | 0.0008199 | 1.773399174 | 34.16957079 | 34.34011431 | 55.23659479 | 66.80783013 |
| FGR | Gardner-Rasheed feline sarcoma viral (v-fgr) oncogene homolog | 0.00052086 | 1.771982153 | 1190.570402 | 1121.540138 | 2026.455159 | 2068.957603 |
| DUT | deoxyuridine triphosphatase | 0.0015845 | 1.771839488 | 149.3422112 | 144.4659234 | 268.8120937 | 251.9694828 |
| CARD11 | caspase recruitment domain family. member 11 | 0.0013115 | 1.771431454 | 600.8601605 | 704.3804667 | 1047.829581 | 1267.473133 |
| SNORD12C | small nucleolar RNA. C/D box 12C | 0.00082188 | 1.769802619 | 28.22567096 | 29.98292047 | 53.98373256 | 49.1026538 |
| GUSBL1 | glucuronidase. beta-like 1 | 0.00193584 | 1.767847135 | 236.6185695 | 308.4334232 | 473.5061395 | 481.6971337 |
| NCF4 | neutrophil cytosolic factor 4. 40kDa | 0.00134122 | 1.767495098 | 142.4301348 | 159.0151583 | 292.2858547 | 242.0745032 |
| ACSS1 | acyl-CoA synthetase short-chain family member 1 | 0.00158624 | 1.766952316 | 236.0059112 | 280.2273754 | 432.3227536 | 477.611753 |
| C19orf39 | chromosome 19 open reading frame 39 | 0.01190263 | 1.766515057 | 21.22567595 | 29.89364064 | 41.55758856 | 47.64580697 |
| SMAP2 | small ArfGAP2 | 0.00771373 | 1.764561462 | 297.1143964 | 459.6155468 | 667.6313313 | 636.8765768 |
| PTGR2 | prostaglandin reductase 2 | 0.04798218 | 1.764198065 | 44.65022446 | 48.67541009 | 56.70517505 | 119.2903335 |
| KAT2A | K(lysine) acetyltransferase 2A | 0.00108543 | 1.762245749 | 416.1889203 | 388.8104573 | 648.1205614 | 775.3639321 |
| LOC728903 | hypothetical LOC728903 | 0.00428145 | 1.762102427 | 351.1132764 | 503.3528231 | 779.0669033 | 704.3804667 |
| NDUFB1 | NADH dehydrogenase (ubiquinone) 1 beta subcomplex. 1. 7kDa | 0.00619579 | 1.761075675 | 46.21005456 | 32.52715688 | 76.2802985 | 61.11196361 |
| STAG3L3 | stromal antigen 3-like 3 | 0.00069297 | 1.760007991 | 354.2285155 | 377.6237817 | 687.1660284 | 602.9904784 |
| QRFPR | pyroglutamylated RFamide peptide receptor | 0.00622822 | 1.760005616 | 542.1580019 | 627.2732123 | 1040.759079 | 1012.186434 |
| FAM170B | family with sequence similarity 170. member B | 0.0190643 | 1.759590606 | 14.22313083 | 19.60611951 | 35.71842046 | 24.17229437 |
| SLC2A3 | solute carrier family 2 (facilitated glucose transporter). member 3 | 0.0005175 | 1.758670551 | 137.7066565 | 147.5842521 | 240.2130915 | 261.678031 |
| RTBDN | retbindin | 0.0117281 | 1.75791186 | 23.05342059 | 20.30458822 | 30.84125681 | 46.9020348 |
| HES6 | hairy and enhancer of split 6 (Drosophila) | 0.00223117 | 1.757473441 | 107.4285181 | 91.12765612 | 172.4654361 | 175.3255684 |
| CD27 | CD27 molecule | 0.00041114 | 1.757325774 | 810.9257594 | 823.0754213 | 1499.012251 | 1375.05511 |
| C12orf35 | chromosome 12 open reading frame 35 | 0.0009529 | 1.757032848 | 179.2774647 | 147.8345939 | 296.0972985 | 276.3293979 |
| LRRC56 | leucine rich repeat containing 56 | 0.00527826 | 1.756800253 | 75.90615065 | 75.93427383 | 133.3371854 | 133.4161179 |
| GFOD2 | glucose-fructose oxidoreductase domain containing 2 | 0.00489066 | 1.754146924 | 70.86366136 | 94.76648335 | 145.3370391 | 142.178516 |
| ZNF69 | zinc finger protein 69 | 0.00875026 | 1.753829937 | 607.5485313 | 921.3010162 | 1242.333123 | 1385.860188 |
| FGF9 | fibroblast growth factor 9 (glia-activating factor) | 0.0059474 | 1.753334983 | 19.67407133 | 21.51359458 | 43.95819314 | 29.60037307 |
| TLE3 | transducin-like enhancer of split 3 (E(sp1) homolog. Drosophila) | 0.00328134 | 1.75271152 | 28.91063344 | 36.23538512 | 57.65153399 | 55.82137162 |
| C2orf56 | chromosome 2 open reading frame 56 | 0.01505246 | 1.752116611 | 101.6861218 | 147.6150724 | 218.9806308 | 210.4324441 |
| HRK | harakiri. BCL2 interacting protein (contains only BH3 domain) | 0.00424347 | 1.750143307 | 237.0933833 | 261.6385697 | 442.2589388 | 429.6272343 |
| DMC1 | DMC1 dosage suppressor of mck1 homolog. meiosis-specific homologous recombination (yeast) | 0.00086721 | 1.74933646 | 1477.029744 | 1615.699067 | 2494.094861 | 2928.083407 |
| LOC645381 | transducin-like enhancer of split 4 (E(sp1) homolog. Drosophila) pseudogene | 0.0006118 | 1.749295252 | 18.14221983 | 19.7872685 | 34.04964833 | 32.26189516 |
| RSAD1 | radical S-adenosyl methionine domain containing 1 | 0.00523015 | 1.746842352 | 59.42827219 | 43.97750885 | 86.84348182 | 91.83197547 |
| RAB26 | RAB26. member RAS oncogene family | 0.00047862 | 1.746609107 | 46.83895384 | 43.02635784 | 75.63432399 | 81.28572469 |
| PAFAH2 | platelet-activating factor acetylhydrolase 2. 40kDa | 0.00220705 | 1.745298552 | 29.114942 | 22.37326632 | 41.97672266 | 47.26898208 |
| PLEKHF1 | pleckstrin homology domain containing. family F (with FYVE domain) member 1 | 0.00176822 | 1.744045078 | 46.13488336 | 39.36884963 | 69.47281967 | 79.52114738 |
| POMT1 | protein-O-mannosyltransferase 1 | 0.00037575 | 1.742722779 | 76.75077975 | 75.19342928 | 128.3171575 | 136.5949279 |
| LOC729603 | calcium binding protein P22 pseudogene | 0.00223789 | 1.742302613 | 3948.265472 | 4746.722694 | 7381.120974 | 7707.704551 |
| CSAG1 | chondrosarcoma associated gene 1 | 0.107028 | 1.742265707 | 15.36254111 | 17.67869799 | 35.47926179 | 23.23633006 |
| FAM153B | family with sequence similarity 153. member B | 0.00059985 | 1.740295344 | 39.98081009 | 41.72220527 | 71.60976474 | 70.5492684 |
| LOC100133177 | hypothetical LOC100133177 | 0.00120622 | 1.740204412 | 4176.551415 | 4703.170552 | 7301.998177 | 8146.43076 |
| WASF3 | WAS protein family. member 3 | 0.0012273 | 1.738831075 | 19.97094436 | 18.31733511 | 30.07793525 | 36.77288118 |
| TP53TG1 | TP53 target 1 (non-protein coding) | 0.0079571 | 1.738219824 | 19.80065255 | 23.43342413 | 40.95855501 | 34.22788204 |
| PLEKHB2 | pleckstrin homology domain containing. family B (evectins) member 2 | 0.03689087 | 1.738103761 | 26.5654418 | 38.02774281 | 46.18563901 | 66.07878256 |
| NCRNA00259 | non-protein coding RNA 259 | 0.00045389 | 1.738018567 | 19.53551351 | 19.39863153 | 32.36346122 | 35.37119939 |
| BAG3 | BCL2-associated athanogene 3 | 0.02630921 | 1.737720039 | 68.02849357 | 97.03456116 | 146.7575381 | 135.8239952 |
| SNORA80 | small nucleolar RNA. H/ACA box 80 | 0.00054788 | 1.737707234 | 27.83078889 | 31.39863175 | 52.12989267 | 50.61772566 |
| FOXO3 | forkhead box O3 | 0.00064064 | 1.73610151 | 547.4385371 | 551.6883079 | 940.0683857 | 968.3222884 |
| C19orf66 | chromosome 19 open reading frame 66 | 0.00058302 | 1.735249166 | 60.8021008 | 58.76183819 | 107.6032402 | 99.97980777 |
| ZBP1 | Z-DNA binding protein 1 | 0.0030503 | 1.734919358 | 25.0479449 | 25.56865328 | 40.37634677 | 47.74319921 |
| SNX22 | sorting nexin 22 | 0.00476833 | 1.732150632 | 146.8692495 | 144.9708586 | 257.6684088 | 247.9258006 |
| SIPA1 | signal-induced proliferation-associated 1 | 0.00052132 | 1.731197388 | 499.9043378 | 459.1520841 | 823.4864995 | 835.3724684 |
| POGZ | pogo transposable element with ZNF domain | 0.00128979 | 1.730940763 | 34.74606755 | 36.99252295 | 62.25780953 | 61.85718986 |
| LPAR2 | lysophosphatidic acid receptor 2 | 0.00212379 | 1.729120024 | 120.6460186 | 125.3238917 | 187.1187324 | 241.5905156 |
| NRBP2 | nuclear receptor binding protein 2 | 0.01128953 | 1.729056083 | 46.59159959 | 34.53056557 | 71.2553879 | 67.50124203 |
| PRRT2 | proline-rich transmembrane protein 2 | 0.08944292 | 1.728645899 | 29.45733512 | 19.44040842 | 32.00341756 | 53.47053909 |
| GCLM | glutamate-cysteine ligase. modifier subunit | 0.06543545 | 1.727761323 | 27.68428381 | 17.11341677 | 35.86968556 | 39.4284719 |
| LOC100128510 | hypothetical protein LOC100128510 | 0.00551363 | 1.727514326 | 165.8945647 | 237.3279874 | 341.6493285 | 343.9092505 |
| FOXJ2 | forkhead box J2 | 0.00092384 | 1.72729919 | 151.4452624 | 150.1141968 | 242.2599912 | 279.9825087 |
| RN28S1 | RNA. 28S ribosomal 1 | 0.00356567 | 1.727101748 | 1681.958203 | 1419.43511 | 2697.130943 | 2640.368595 |
| FBXO32 | F-box protein 32 | 0.00148486 | 1.72680023 | 102.2942485 | 84.88077927 | 152.5461444 | 169.7241078 |
| SERTAD2 | SERTA domain containing 2 | 0.00044399 | 1.726272647 | 244.5685259 | 252.2769578 | 433.0559311 | 424.5735531 |
| TBC1D10C | TBC1 domain family. member 10C | 0.00175058 | 1.723883252 | 217.9629183 | 260.398764 | 441.5276657 | 382.014029 |
| IQSEC1 | IQ motif and Sec7 domain 1 | 0.00076628 | 1.72176786 | 193.1289751 | 178.5891622 | 319.0313257 | 320.4928907 |
| C20orf177 | chromosome 20 open reading frame 177 | 0.00041552 | 1.721140435 | 169.4746393 | 174.4950229 | 303.585056 | 288.5625638 |
| PLEKHA1 | pleckstrin homology domain containing. family A (phosphoinositide binding specific) member 1 | 0.00114952 | 1.720393745 | 31.11144915 | 26.86952342 | 47.46233313 | 52.12989267 |
| IFI6 | interferon. alpha-inducible protein 6 | 0.00083414 | 1.718398445 | 135.9622198 | 160.0500366 | 255.3686916 | 251.6251895 |
| C2orf69 | chromosome 2 open reading frame 69 | 0.00062633 | 1.71808047 | 1271.271775 | 1395.470375 | 2234.123249 | 2343.899033 |
| PLEKHO2 | pleckstrin homology domain containing. family O member 2 | 0.00314355 | 1.717262829 | 57.23482388 | 62.37246906 | 107.1268389 | 98.27171526 |
| MYO5A | myosin VA (heavy chain 12. myoxin) | 0.0033707 | 1.717210019 | 50.66988163 | 43.42225994 | 83.14866468 | 78.0286083 |
| MMP23B | matrix metallopeptidase 23B | 0.00123522 | 1.717065438 | 21.34463182 | 24.16231656 | 36.95129107 | 41.15013972 |
| CLOCK | clock homolog (mouse) | 0.00290909 | 1.717031688 | 50.5890661 | 49.62790728 | 74.83771711 | 98.90510614 |
| GLS | glutaminase | 0.0058568 | 1.716821928 | 488.7020156 | 475.1146834 | 694.0278709 | 986.0891375 |
| MAFF | v-maf musculoaponeurotic fibrosarcoma oncogene homolog F (avian) | 0.00521017 | 1.716671942 | 30.64744767 | 42.79732295 | 64.7785657 | 59.66974835 |
| CRIP1 | cysteine-rich protein 1 (intestinal) | 0.00547706 | 1.716332146 | 616.0035766 | 433.4805363 | 829.5681649 | 948.207599 |
| PDE7A | phosphodiesterase 7A | 0.00384463 | 1.715293487 | 111.401519 | 149.2752375 | 204.2727179 | 239.5219911 |
| APITD1 | apoptosis-inducing. TAF9-like domain 1 | 0.00470561 | 1.715289131 | 51.47003469 | 41.76214606 | 82.97263714 | 76.22142201 |
| SULT1A1 | sulfotransferase family. cytosolic. 1A. phenol-preferring. member 1 | 0.00120164 | 1.713649214 | 844.9606282 | 992.9155745 | 1475.769831 | 1669.45232 |
| TMEM107 | transmembrane protein 107 | 0.00351173 | 1.713309153 | 58.04158104 | 65.53644934 | 110.1245423 | 101.3933556 |
| KYNU | kynureninase (L-kynurenine hydrolase) | 0.0011063 | 1.71321222 | 251.436076 | 211.5173245 | 407.7731872 | 382.8046422 |
| PTP4A3 | protein tyrosine phosphatase type IVA. member 3 | 0.00147546 | 1.713191261 | 240.7680619 | 243.9875869 | 459.96471 | 374.846791 |
| FLJ40504 | keratin 18 pseudogene | 0.00673279 | 1.712675003 | 22.14413756 | 17.78633925 | 29.12017954 | 39.67356363 |
| RNF149 | ring finger protein 149 | 0.0112315 | 1.712621145 | 34.35838329 | 35.99157001 | 49.77427264 | 72.87040669 |
| HEATR5A | HEAT repeat containing 5A | 0.02171115 | 1.711904614 | 29.48078902 | 24.59357415 | 50.71414502 | 41.89775649 |
| GSDMB | gasdermin B | 0.00448046 | 1.711402478 | 60.97567938 | 55.11674653 | 110.3209261 | 89.22501623 |
| SPN | sialophorin | 0.00056054 | 1.709505294 | 151.7155192 | 142.2306861 | 249.2482581 | 253.0067344 |
| B3GNT1 | UDP-GlcNAc:betaGal beta-1.3-N-acetylglucosaminyltransferase 1 | 0.00287878 | 1.709468396 | 205.3814481 | 198.1182006 | 354.9989257 | 334.9505537 |
| ABLIM1 | actin binding LIM protein 1 | 0.00093936 | 1.708788178 | 310.4608342 | 302.1212702 | 491.2793257 | 557.4887343 |
| RNF170 | ring finger protein 170 | 0.00111795 | 1.708717963 | 58.92868754 | 62.11741217 | 98.88035538 | 108.0863558 |
| RPL7L1 | ribosomal protein L7-like 1 | 0.00180459 | 1.708603405 | 2594.737162 | 3293.815929 | 5121.597946 | 4871.579001 |
| LOC100130168 | hypothetical protein LOC100130168 | 0.00230178 | 1.708344922 | 3220.098323 | 3625.736227 | 6222.80597 | 5475.580916 |
| HLA-DPB1 | major histocompatibility complex. class II. DP beta 1 | 0.00114554 | 1.708220893 | 45.55952161 | 40.11392822 | 70.99457188 | 75.1168318 |
| TAGAP | T-cell activation RhoGTPase activating protein | 0.00241508 | 1.708033631 | 188.6934579 | 200.4137274 | 314.0450335 | 351.3057145 |
| PGBD4 | piggyBac transposable element derived 4 | 0.01876879 | 1.706083472 | 21.61999249 | 26.98323053 | 48.11255099 | 35.29324975 |
| CD59 | CD59 molecule. complement regulatory protein | 0.00748875 | 1.705802108 | 96.5705637 | 123.2350786 | 163.7436676 | 211.4812616 |
| SRGAP2 | SLIT-ROBO Rho GTPase activating protein 2 | 0.01682864 | 1.704121097 | 25.77803159 | 29.37112811 | 38.58039557 | 56.99078108 |
| SULT1A3 | sulfotransferase family. cytosolic. 1A. phenol-preferring. member 3 | 0.00386544 | 1.704013438 | 48.00327242 | 39.46429689 | 82.67334655 | 66.53585273 |
| FBXL18 | F-box and leucine-rich repeat protein 18 | 0.00147046 | 1.70306969 | 589.6268111 | 721.9092685 | 1141.894203 | 1081.182014 |
| PIK3R3 | phosphoinositide-3-kinase. regulatory subunit 3 (gamma) | 0.00965109 | 1.700725605 | 20.71907361 | 18.7657186 | 30.20329602 | 37.23485735 |
| KIAA0556 | KIAA0556 | 0.00244526 | 1.700496681 | 91.17105473 | 80.91027835 | 143.3862522 | 148.7663605 |
| FCRL2 | Fc receptor-like 2 | 0.00786529 | 1.699963521 | 14.61774031 | 17.93424542 | 32.84282664 | 23.06757955 |
| MFSD11 | major facilitator superfamily domain containing 11 | 0.00071161 | 1.699618506 | 160.3277033 | 179.1002553 | 287.9098477 | 288.1052409 |
| RHBDF2 | rhomboid 5 homolog 2 (Drosophila) | 0.00054303 | 1.699561926 | 445.2961909 | 431.3560788 | 771.0170926 | 719.6061788 |
| TMEM165 | transmembrane protein 165 | 0.00562493 | 1.697528565 | 121.6030652 | 92.39269203 | 201.7264924 | 160.4919998 |
| FBXO30 | F-box protein 30 | 0.010204 | 1.697505484 | 51.38648602 | 34.44225142 | 65.69865443 | 77.62584663 |
| MTMR9 | myotubularin related protein 9 | 0.00053864 | 1.693261657 | 91.97931152 | 97.76075495 | 164.1558947 | 157.0530471 |
| C14orf82 | chromosome 14 open reading frame 82 | 0.02177898 | 1.692661828 | 14.92261484 | 24.67452817 | 32.10678245 | 32.85771831 |
| LOC100129502 | hypothetical protein LOC100129502 | 0.00432629 | 1.692296949 | 136.5864555 | 167.2605118 | 225.9668153 | 289.5406429 |
| MCM8 | minichromosome maintenance complex component 8 | 0.0027038 | 1.692195893 | 2688.994622 | 3548.322398 | 5171.684919 | 5283.019596 |
| RHBDF2 | rhomboid 5 homolog 2 (Drosophila) | 0.00076756 | 1.692056121 | 900.3667641 | 908.6117394 | 1443.499548 | 1622.597017 |
| TYSND1 | trypsin domain containing 1 | 0.0009371 | 1.691940258 | 401.8233797 | 454.8789081 | 688.7226098 | 759.7255106 |
| LILRB1 | leukocyte immunoglobulin-like receptor. subfamily B (with TM and ITIM domains). member 1 | 0.00171567 | 1.691426861 | 2241.405811 | 2492.416955 | 3826.747481 | 4176.551415 |
| LMOD3 | leiomodin 3 (fetal) | 0.00949798 | 1.691036158 | 187.0058455 | 250.4272025 | 382.7028555 | 349.9296715 |
| TDRD1 | tudor domain containing 1 | 0.01291638 | 1.688658182 | 58.69809402 | 92.63657067 | 131.9506117 | 117.5110099 |
| ID3 | inhibitor of DNA binding 3. dominant negative helix-loop-helix protein | 0.00122199 | 1.688592157 | 1280.515276 | 1362.460948 | 2195.271197 | 2266.053624 |
| SERPINA5 | serpin peptidase inhibitor. clade A (alpha-1 antiproteinase. antitrypsin). member 5 | 0.04203065 | 1.687596845 | 25.59585523 | 18.42477558 | 27.79075844 | 48.3291172 |
| MBTD1 | mbt domain containing 1 | 0.00299514 | 1.687428646 | 34.08228524 | 43.68766721 | 67.13757646 | 63.14991017 |
| LOC100190938 | hypothetical LOC100190938 | 0.00398372 | 1.687287705 | 2990.615977 | 3557.550738 | 6033.202668 | 5020.443934 |
| WDR73 | WD repeat domain 73 | 0.03503883 | 1.686584702 | 48.7870924 | 67.85017447 | 104.4537391 | 90.14636669 |
| MARCH2 | membrane-associated ring finger (C3HC4) 2 | 0.00968202 | 1.686439354 | 21.28100745 | 23.15981058 | 44.45400203 | 31.53245452 |
| CSNK1D | casein kinase 1. delta | 0.00245373 | 1.686406112 | 274.0041131 | 307.7046079 | 435.7561628 | 550.264986 |
| RRBP1 | ribosome binding protein 1 homolog 180kDa (dog) | 0.00065062 | 1.686368877 | 274.3430735 | 275.3351605 | 440.7181137 | 487.4161677 |
| C10orf58 | chromosome 10 open reading frame 58 | 0.00150512 | 1.685298622 | 77.71428667 | 84.64886303 | 131.7735862 | 141.7905739 |
| TMOD2 | tropomodulin 2 (neuronal) | 0.00106262 | 1.68499276 | 50.97277081 | 49.06360091 | 91.26018894 | 77.80587226 |
| ABR | active BCR-related gene | 0.00104786 | 1.6844726 | 419.1147448 | 448.4832754 | 682.0663974 | 781.9526215 |
| DENR | density-regulated protein | 0.00056433 | 1.684277584 | 128.1438556 | 137.2780184 | 222.6253796 | 224.1565598 |
| HERPUD2 | HERPUD family member 2 | 0.00642823 | 1.684137472 | 43.26109597 | 50.27448533 | 90.1941869 | 68.39457908 |
| LOC100133182 | hypothetical protein LOC100133182 | 0.00730001 | 1.683730572 | 21.99492746 | 18.79797199 | 37.49118839 | 31.26435839 |
| ROM1 | retinal outer segment membrane protein 1 | 0.01432561 | 1.683273185 | 47.17044225 | 49.40863108 | 77.62584663 | 85.06984265 |
| VGLL4 | vestigial like 4 (Drosophila) | 0.00059593 | 1.681760339 | 404.2007823 | 423.8613161 | 702.8084789 | 689.4648856 |
| GPR65 | G protein-coupled receptor 65 | 0.00089746 | 1.681650241 | 57.62688168 | 57.9469116 | 96.89749386 | 97.45726438 |
| LOC151162 | hypothetical LOC151162 | 0.00098925 | 1.679439406 | 173.54267 | 161.472077 | 296.6130465 | 266.4662041 |
| NAAA | N-acylethanolamine acid amidase | 0.00116284 | 1.678966452 | 30.67100701 | 27.39676779 | 46.75997678 | 50.65672563 |
| NCF1C | neutrophil cytosolic factor 1C pseudogene | 0.00550412 | 1.678006929 | 619.7335307 | 748.9094452 | 1321.451492 | 988.9414419 |
| LOC100287081 | similar to hCG1999172 | 0.00148332 | 1.675591338 | 168.1817656 | 170.9557446 | 309.6087002 | 260.7267935 |
| CCDC6 | coiled-coil domain containing 6 | 0.00118273 | 1.675529366 | 842.7943911 | 888.0789017 | 1414.761715 | 1485.230907 |
| RAX2 | retina and anterior neural fold homeobox 2 | 0.00204947 | 1.675012876 | 112.394018 | 111.9229411 | 210.3054394 | 167.8216958 |
| CTSH | cathepsin H | 0.01047225 | 1.674922415 | 24.66192775 | 23.58620021 | 46.38160352 | 35.1826568 |
| IDS | iduronate 2-sulfatase | 0.0389963 | 1.674655601 | 35.85014754 | 29.67360251 | 43.8624939 | 68.01722574 |
| RAX2 | retina and anterior neural fold homeobox 2 | 0.00657185 | 1.673946247 | 16.68025687 | 18.72003309 | 25.40778081 | 34.4370252 |
| SCPEP1 | serine carboxypeptidase 1 | 0.00057832 | 1.673834836 | 801.4183568 | 752.2720611 | 1313.371172 | 1286.091723 |
| LOC100134159 | similar to Coiled-coil domain containing 144B | 0.00585144 | 1.673753669 | 1926.082033 | 1896.640924 | 3064.428289 | 3339.592818 |
| NPEPL1 | aminopeptidase-like 1 | 0.00299427 | 1.673743072 | 105.1671457 | 107.6032402 | 185.736688 | 170.681057 |
| TMEM44 | transmembrane protein 44 | 0.0979033 | 1.673422135 | 41.50581276 | 27.89828288 | 48.6333339 | 66.67505343 |
| PITPNC1 | phosphatidylinositol transfer protein. cytoplasmic 1 | 0.00106831 | 1.67262563 | 120.3306068 | 110.5875773 | 205.4222051 | 181.2310298 |
| STK36 | serine/threonine kinase 36 | 0.00069058 | 1.671896011 | 262.80835 | 239.4389487 | 416.7248361 | 422.0880876 |
| BRI3BP | BRI3 binding protein | 0.02652675 | 1.671667963 | 14.62709494 | 25.62948891 | 33.79072753 | 31.0027717 |
| MIR1323 | microRNA 1323 | 0.03001977 | 1.671270908 | 18.39075594 | 23.06650153 | 37.71321693 | 31.41821007 |
| MGEA5 | meningioma expressed antigen 5 (hyaluronidase) | 0.00079 | 1.67124193 | 1313.371172 | 1349.776282 | 2157.305208 | 2295.177757 |
| GM2A | GM2 ganglioside activator | 0.00485532 | 1.670484488 | 83.24280214 | 112.394018 | 162.6307526 | 160.5358791 |
| PRKCB | protein kinase C. beta | 0.00140644 | 1.669169386 | 174.5417294 | 183.3109624 | 310.6666603 | 286.9418441 |
| RASSF6 | Ras association (RalGDS/AF-6) domain family member 6 | 0.00312851 | 1.668650083 | 240.4824531 | 225.3415602 | 411.7323926 | 366.4714978 |
| ZNF608 | zinc finger protein 608 | 0.00161097 | 1.668581987 | 92.63657067 | 90.24549 | 150.0091078 | 155.1620291 |
| SLC29A1 | solute carrier family 29 (nucleoside transporters). member 1 | 0.00146275 | 1.668261398 | 178.7414394 | 207.7546936 | 316.7838405 | 326.2430563 |
| POU2F2 | POU class 2 homeobox 2 | 0.00128417 | 1.668221263 | 72.44162342 | 75.78299525 | 113.9708396 | 134.0520618 |
| ZC3HAV1 | zinc finger CCCH-type. antiviral 1 | 0.00067631 | 1.667762834 | 471.7109136 | 477.1905494 | 791.2581033 | 791.2581033 |
| DACT3 | dapper. antagonist of beta-catenin. homolog 3 (Xenopus laevis) | 0.00083127 | 1.666831802 | 40.90144919 | 43.38576136 | 68.36454411 | 72.11714956 |
| LOC644525 | chromosome 2 open reading frame 27A pseudogene | 0.00903204 | 1.666489306 | 25.87001022 | 29.20190886 | 55.49195585 | 37.80792752 |
| CDK11A | cyclin-dependent kinase 11A | 0.00563386 | 1.665349717 | 35.82664286 | 33.75081525 | 63.45812355 | 52.84623493 |
| SLC2A11 | solute carrier family 2 (facilitated glucose transporter). member 11 | 0.00094447 | 1.665121078 | 63.79913613 | 67.50124203 | 116.3022124 | 102.6668465 |
| FCRL3 | Fc receptor-like 3 | 0.00503596 | 1.664306638 | 23.57286098 | 23.30828244 | 37.79423054 | 40.26834216 |
| LOC441087 | hypothetical LOC441087 | 0.00937611 | 1.664259925 | 2231.370596 | 2781.198722 | 4077.668958 | 4215.354207 |
| EHBP1L1 | EH domain binding protein 1-like 1 | 0.00924649 | 1.663612088 | 281.7910089 | 288.8156461 | 508.6384238 | 442.8358975 |
| C3orf58 | chromosome 3 open reading frame 58 | 0.00139186 | 1.663565845 | 43.18238838 | 36.73304455 | 66.61773847 | 65.89518626 |
| NXF1 | nuclear RNA export factor 1 | 0.00196855 | 1.661466543 | 435.0151544 | 532.9788292 | 801.925756 | 798.1111599 |
| LOC100130906 | hypothetical LOC100130906 | 0.01808115 | 1.660333381 | 33.72394872 | 26.29729125 | 55.33397388 | 44.18228519 |
| MBD4 | methyl-CpG binding domain protein 4 | 0.00727944 | 1.659848462 | 364.1632144 | 519.4753654 | 687.5590099 | 758.0326893 |
| CRADD | CASP2 and RIPK1 domain containing adaptor with death domain | 0.00150059 | 1.659475316 | 147.1045683 | 147.2936549 | 243.7553431 | 244.792241 |
| IL18 | interleukin 18 (interferon-gamma-inducing factor) | 0.0013308 | 1.659350883 | 3669.998904 | 4019.823507 | 5987.047251 | 6784.794464 |
| RNU4ATAC | RNA. U4atac small nuclear (U12-dependent splicing) | 0.00220611 | 1.659285673 | 26.07565282 | 31.10291603 | 49.89048278 | 44.75699478 |
| NENF | neuron derived neurotrophic factor | 0.00064919 | 1.659190364 | 613.6363651 | 647.0144759 | 1039.471156 | 1051.489831 |
| TST | thiosulfate sulfurtransferase (rhodanese) | 0.00104187 | 1.658774802 | 432.1533021 | 452.0282781 | 729.0010943 | 737.3099907 |
| ARHGAP24 | Rho GTPase activating protein 24 | 0.00141977 | 1.658669301 | 40.57524898 | 38.89658005 | 62.22402472 | 69.780508 |
| PLEKHA2 | pleckstrin homology domain containing. family A (phosphoinositide binding specific) member 2 | 0.00112155 | 1.658610487 | 960.6280329 | 987.0994081 | 1723.289496 | 1513.724084 |
| HSCB | HscB iron-sulfur cluster co-chaperone homolog (E. coli) | 0.00352424 | 1.658350828 | 333.6513196 | 363.1191622 | 518.997942 | 641.9913889 |
| ITSN1 | intersectin 1 (SH3 domain protein) | 0.00417793 | 1.657799274 | 109.2237672 | 85.75283422 | 158.2802958 | 162.6307526 |
| RGPD1 | RANBP2-like and GRIP domain containing 1 | 0.00179855 | 1.657719552 | 20.97408283 | 18.89952529 | 31.30784833 | 34.79387295 |
| C14orf104 | chromosome 14 open reading frame 104 | 0.00235487 | 1.657110289 | 18.71183959 | 20.80166748 | 36.40361022 | 29.36114634 |
| TNFSF15 | tumor necrosis factor (ligand) superfamily. member 15 | 0.02135488 | 1.65690786 | 54.96488995 | 54.40241973 | 77.51328321 | 105.9068775 |
| ZNF318 | zinc finger protein 318 | 0.00064038 | 1.654870056 | 221.8159897 | 232.4230931 | 375.039019 | 376.4640129 |
| KCND1 | potassium voltage-gated channel. Shal-related subfamily. member 1 | 0.00650323 | 1.654572965 | 19.04479234 | 26.59014752 | 35.62413951 | 38.9156647 |
| LPAR5 | lysophosphatidic acid receptor 5 | 0.00139679 | 1.653118095 | 120.0369481 | 108.4664854 | 198.9545868 | 178.8398582 |
| SPNS2 | spinster homolog 2 (Drosophila) | 0.00139464 | 1.653038698 | 24.07012714 | 20.75161724 | 35.21238909 | 38.76152738 |
| C6orf192 | chromosome 6 open reading frame 192 | 0.00099653 | 1.651767692 | 74.83771711 | 79.86436693 | 133.2207951 | 122.4050956 |
| STAT5A | signal transducer and activator of transcription 5A | 0.00130146 | 1.651135724 | 114.9786439 | 118.3491903 | 177.4902462 | 209.0131109 |
| SBF1 | SET binding factor 1 | 0.00247205 | 1.650606381 | 642.2056818 | 775.5180547 | 1161.278429 | 1168.467768 |
| TICAM2 | toll-like receptor adaptor molecule 2 | 0.00125907 | 1.649571775 | 27.74639375 | 30.68472936 | 46.05010578 | 50.30841605 |
| RAD21L1 | RAD21-like 1 (S. pombe) | 0.11497227 | 1.648905797 | 21.03690952 | 15.75495948 | 31.19834785 | 28.88413214 |
| CREB1 | cAMP responsive element binding protein 1 | 0.00118289 | 1.648778724 | 4621.11491 | 5192.194334 | 7853.387026 | 8305.49374 |
| PHYH | phytanoyl-CoA 2-hydroxylase | 0.00078882 | 1.648671232 | 178.0438173 | 187.5481921 | 310.0110825 | 292.7727683 |
| LPHN1 | latrophilin 1 | 0.00061049 | 1.647735951 | 50.23374725 | 51.55910598 | 83.82214744 | 83.89139316 |
| SPDYE8P | speedy homolog E8 (Xenopus laevis). pseudogene | 0.00149788 | 1.6476779 | 3598.79032 | 4286.56157 | 6326.053597 | 6620.295514 |
| DBT | dihydrolipoamide branched chain transacylase E2 | 0.00201993 | 1.64719456 | 215.8873392 | 216.9171984 | 322.2496081 | 394.2925408 |
| ST6GAL1 | ST6 beta-galactosamide alpha-2.6-sialyltranferase 1 | 0.00668216 | 1.647131409 | 114.3158622 | 127.1767727 | 205.5993156 | 191.8444011 |
| KLF13 | Kruppel-like factor 13 | 0.00106157 | 1.646691228 | 1085.867591 | 1230.39019 | 1860.942194 | 1946.754522 |
| ISG20L2 | interferon stimulated exonuclease gene 20kDa-like 2 | 0.01848878 | 1.646601416 | 184.7186263 | 194.1252712 | 336.0744746 | 289.2905226 |
| TMEM170B | transmembrane protein 170B | 0.00170016 | 1.646587961 | 100.9727027 | 84.56794219 | 152.3775799 | 151.9352497 |
| ZNF324B | zinc finger protein 324B | 0.06420214 | 1.646471927 | 47.86285261 | 23.42722491 | 52.48496699 | 57.91527964 |
| CIAO1 | cytosolic iron-sulfur protein assembly 1 | 0.00108395 | 1.645223458 | 767.7476124 | 723.7121458 | 1305.197763 | 1152.279409 |
| POLR3GL | polymerase (RNA) III (DNA directed) polypeptide G (32kD)-like | 0.0010045 | 1.64474952 | 744.1292108 | 694.721998 | 1166.383833 | 1198.995213 |
| CUEDC1 | CUE domain containing 1 | 0.00166365 | 1.644343322 | 19.09812597 | 19.89050362 | 30.91928844 | 33.21941957 |
| ADAP1 | ArfGAP with dual PH domains 1 | 0.00079801 | 1.64396223 | 87.77775508 | 84.81698336 | 139.9896602 | 143.7325078 |
| UBTF | upstream binding transcription factor. RNA polymerase I | 0.0023343 | 1.642616897 | 45.39879064 | 40.77892166 | 71.76282023 | 69.60702871 |
| NFIA | nuclear factor I/A | 0.00626588 | 1.641852358 | 48.7328899 | 54.75848091 | 81.39275447 | 88.38041036 |
| IFIT3 | interferon-induced protein with tetratricopeptide repeats 3 | 0.00387344 | 1.640961406 | 31.16642794 | 36.93611334 | 58.82082125 | 52.69918199 |
| ITPKB | inositol 1.4.5-trisphosphate 3-kinase B | 0.00114309 | 1.640137757 | 58.11329556 | 62.7001442 | 97.98056899 | 100.03794 |
| DEGS1 | degenerative spermatocyte homolog 1. lipid desaturase (Drosophila) | 0.00240225 | 1.639911693 | 405.9112657 | 342.6523211 | 624.6528821 | 598.8071263 |
| SLCO4A1 | solute carrier organic anion transporter family. member 4A1 | 0.0039867 | 1.638710039 | 44.89906748 | 58.23547982 | 88.94438783 | 78.9424603 |
| MBP | myelin basic protein | 0.01622219 | 1.637854434 | 17.58896093 | 26.32215598 | 34.74852362 | 35.7417561 |
| CDK5R1 | cyclin-dependent kinase 5. regulatory subunit 1 (p35) | 0.00820227 | 1.637670975 | 117.7594583 | 114.6018827 | 159.4875272 | 226.9416098 |
| IL23A | interleukin 23. alpha subunit p19 | 0.01750757 | 1.636889936 | 915.9286761 | 878.0384384 | 1206.90406 | 1785.424105 |
| ZNF174 | zinc finger protein 174 | 0.01527054 | 1.636181611 | 35.00095847 | 23.21597106 | 50.61772566 | 42.97611745 |
| C20orf72 | chromosome 20 open reading frame 72 | 0.00109084 | 1.636173924 | 453.854901 | 492.1958528 | 804.6044635 | 743.2441098 |
| FAM21D | family with sequence similarity 21. member D | 0.0088433 | 1.636030232 | 28.23384157 | 40.39518561 | 51.81556897 | 58.91446788 |
| CXXC5 | CXXC finger 5 | 0.00273304 | 1.635987515 | 1343.159767 | 1506.321127 | 2211.392572 | 2448.721336 |
| CHMP7 | CHMP family. member 7 | 0.00073917 | 1.635829587 | 31.80271184 | 30.75594359 | 50.53040994 | 51.79841924 |
| TNFRSF1B | tumor necrosis factor receptor superfamily. member 1B | 0.00154647 | 1.635811707 | 30.91928844 | 30.34184287 | 52.92804797 | 47.4298982 |
| P2RY11 | purinergic receptor P2Y. G-protein coupled. 11 | 0.00329542 | 1.635749794 | 120.8449919 | 130.4826314 | 182.1677948 | 231.6026427 |
| EVI2A | ecotropic viral integration site 2A | 0.02619505 | 1.634834007 | 70.17476812 | 43.71519707 | 98.09864345 | 83.57911174 |
| FYTTD1 | forty-two-three domain containing 1 | 0.00263444 | 1.634587115 | 30.31198815 | 33.0955417 | 51.55295381 | 51.9931949 |
| LOC730060 | hypothetical LOC730060 | 0.01540018 | 1.634169436 | 39.0893503 | 32.5142537 | 56.8921589 | 59.65872879 |
| MS4A1 | membrane-spanning 4-domains. subfamily A. member 1 | 0.01296336 | 1.633183188 | 97.63236966 | 82.67744071 | 162.7893305 | 132.258843 |
| C2orf68 | chromosome 2 open reading frame 68 | 0.00159311 | 1.632799326 | 42.33436798 | 36.09706059 | 65.15126257 | 62.5327758 |
| PPP1R16A | protein phosphatase 1. regulatory (inhibitor) subunit 16A | 0.00733061 | 1.632362373 | 94.54343738 | 132.5337156 | 175.806172 | 189.9139186 |
| DNAJC28 | DnaJ (Hsp40) homolog. subfamily C. member 28 | 0.00137232 | 1.631959228 | 3900.915582 | 4478.382115 | 6590.195281 | 7060.054017 |
| LOC100129094 | similar to hCG2036765 | 0.00489359 | 1.631726294 | 41.73788821 | 50.24072622 | 72.37547492 | 77.14176602 |
| BRI3BP | BRI3 binding protein | 0.00392306 | 1.63134521 | 103.9337124 | 91.37760907 | 170.2009182 | 148.4998708 |
| TARSL2 | threonyl-tRNA synthetase-like 2 | 0.00624519 | 1.630869678 | 31.16267634 | 30.24631545 | 55.78084512 | 44.94285448 |
| FCAR | Fc fragment of IgA. receptor for | 0.01703479 | 1.630562749 | 161.7399048 | 183.2899931 | 263.9508309 | 298.6124683 |
| SSTR2 | somatostatin receptor 2 | 0.00888377 | 1.629778924 | 72.30417254 | 99.2895857 | 129.2468773 | 147.5381707 |
| ZBTB7B | zinc finger and BTB domain containing 7B | 0.00361309 | 1.629597719 | 25.16869222 | 25.96156882 | 47.44634857 | 36.57207516 |
| SNAP47 | synaptosomal-associated protein. 47kDa | 0.00653726 | 1.628718628 | 129.6208385 | 106.3496416 | 178.1747857 | 205.2375111 |
| LIME1 | Lck interacting transmembrane adaptor 1 | 0.00087205 | 1.628213947 | 412.4787232 | 405.8003111 | 671.4115228 | 660.9187574 |
| C8orf55 | chromosome 8 open reading frame 55 | 0.00073653 | 1.628046903 | 421.8619811 | 440.5259131 | 695.4230111 | 708.3152884 |
| C4orf22 | chromosome 4 open reading frame 22 | 0.00146505 | 1.627750355 | 97.92212862 | 103.7086879 | 152.1662118 | 176.828944 |
| LOC100130764 | p150-like | 0.0107647 | 1.627301062 | 41.39789694 | 43.70636868 | 79.63512186 | 60.16641994 |
| HSPA6 | heat shock 70kDa protein 6 (HSP70B') | 0.05699348 | 1.627173873 | 38.64546567 | 59.03510686 | 90.11401041 | 67.03235934 |
| SULT1A2 | sulfotransferase family. cytosolic. 1A. phenol-preferring. member 2 | 0.00273354 | 1.625923456 | 44.56905825 | 42.41445436 | 77.1633958 | 64.76437857 |
| LOC732360 | similar to G/T mismatch-specific thymine DNA glycosylase | 0.01113299 | 1.625516145 | 72.61602188 | 74.45305074 | 129.9201391 | 109.9565358 |
| CPEB3 | cytoplasmic polyadenylation element binding protein 3 | 0.00225804 | 1.624566748 | 95.98135443 | 113.9000351 | 163.3570786 | 176.6232587 |
| GRN | granulin | 0.01117141 | 1.624517223 | 361.0667742 | 345.1288064 | 626.5024717 | 524.921773 |
| HAVCR2 | hepatitis A virus cellular receptor 2 | 0.00151298 | 1.623981187 | 22.45960086 | 19.7893316 | 35.86249676 | 32.68546181 |
| PTPRCAP | protein tyrosine phosphatase. receptor type. C-associated protein | 0.00244915 | 1.623572807 | 342.1861523 | 373.0659369 | 585.606161 | 574.6268675 |
| TM7SF3 | transmembrane 7 superfamily member 3 | 0.00808054 | 1.623517955 | 65.80797385 | 75.43876239 | 129.0204615 | 101.4211838 |
| LY96 | lymphocyte antigen 96 | 0.00244076 | 1.622787505 | 272.5398735 | 267.4269344 | 429.1594792 | 447.2391327 |
| ARID2 | AT rich interactive domain 2 (ARID. RFX-like) | 0.00415618 | 1.621520739 | 122.9503801 | 94.47847739 | 175.0850248 | 174.4450998 |
| LHX3 | LIM homeobox 3 | 0.00864464 | 1.620609722 | 63.77403993 | 72.47574873 | 113.7746065 | 106.6960097 |
| HIBCH | 3-hydroxyisobutyryl-CoA hydrolase | 0.00589078 | 1.619285189 | 204.844755 | 274.0041131 | 384.5624845 | 382.7028555 |
| ST3GAL1 | ST3 beta-galactoside alpha-2.3-sialyltransferase 1 | 0.05947438 | 1.618595378 | 29.53952326 | 43.39998256 | 73.80734803 | 45.50614305 |
| SOD2 | superoxide dismutase 2. mitochondrial | 0.04183619 | 1.61858823 | 21.22845279 | 35.10509697 | 37.0076118 | 52.75580028 |
| ALB | albumin | 0.04444493 | 1.616645471 | 20.21424296 | 24.76796843 | 27.13603563 | 48.22042631 |
| LNPEP | leucyl/cystinyl aminopeptidase | 0.0018545 | 1.615616238 | 85.58019478 | 90.11401041 | 136.1220851 | 147.8813527 |
| TRIB1 | tribbles homolog 1 (Drosophila) | 0.00322194 | 1.614396267 | 99.01548909 | 89.74228122 | 136.1693745 | 170.0752387 |
| DOPEY2 | dopey family member 2 | 0.00398383 | 1.614245798 | 143.8698026 | 173.9564087 | 240.8844892 | 270.7326122 |
| ZNF557 | zinc finger protein 557 | 0.00108565 | 1.613895378 | 71.72152985 | 66.93723339 | 109.9351385 | 113.7447946 |
| SNF8 | SNF8. ESCRT-II complex subunit. homolog (S. cerevisiae) | 0.00210941 | 1.61332523 | 470.3142531 | 461.1195066 | 695.0363489 | 812.1532161 |
| LOC100130445 | similar to AML-associated zinc finger protein | 0.00148037 | 1.612972238 | 3181.884644 | 3596.396416 | 5314.77266 | 5601.715906 |
| DCAF11 | DDB1 and CUL4 associated factor 11 | 0.00155221 | 1.612615362 | 129.8711648 | 141.8161033 | 229.8781409 | 208.3541681 |
| LLPH | LLP homolog. long-term synaptic facilitation (Aplysia) | 0.00157681 | 1.612299982 | 332.1312704 | 379.4552449 | 582.6496444 | 562.2824642 |
| C4orf34 | chromosome 4 open reading frame 34 | 0.00164419 | 1.612235713 | 558.2802093 | 620.7703111 | 934.5427285 | 963.9202019 |
| RARA | retinoic acid receptor. alpha | 0.00183357 | 1.612070857 | 139.0057166 | 163.5634529 | 235.9422318 | 250.4272025 |
| KCNN4 | potassium intermediate/small conductance calcium-activated channel. subfamily N. member 4 | 0.03336176 | 1.612012813 | 88.56761567 | 65.4873589 | 97.58432475 | 154.4505099 |
| TSPAN17 | tetraspanin 17 | 0.01010316 | 1.611069373 | 322.4324092 | 292.6078431 | 545.916065 | 448.5669371 |
| SMC4 | structural maintenance of chromosomes 4 | 0.01216343 | 1.610760026 | 40.54023289 | 34.49770569 | 71.92864006 | 50.44710975 |
| SNORD55 | small nucleolar RNA. C/D box 55 | 0.01068307 | 1.610644542 | 45.83439633 | 48.83039521 | 67.48160433 | 86.03908165 |
| WDTC1 | WD and tetratricopeptide repeats 1 | 0.02449482 | 1.610430884 | 20.58235745 | 20.16826511 | 25.5916995 | 42.06769451 |
| PHAX | phosphorylated adaptor for RNA export | 0.0079894 | 1.608579653 | 42.41445436 | 31.28023862 | 56.65644003 | 60.59262971 |
| NCF1B | neutrophil cytosolic factor 1B pseudogene | 0.00163624 | 1.608363291 | 111.1074995 | 108.7836098 | 191.3979081 | 163.3570786 |
| HPS6 | Hermansky-Pudlak syndrome 6 | 0.00112274 | 1.60806052 | 470.949146 | 458.7580872 | 730.6810678 | 764.6006646 |
| PDPK1 | 3-phosphoinositide dependent protein kinase-1 | 0.03170073 | 1.6079467 | 35.1826568 | 59.78935142 | 69.57516507 | 78.17025454 |
| F12 | coagulation factor XII (Hageman factor) | 0.0083902 | 1.60793474 | 53.04234446 | 48.09403672 | 75.23152994 | 87.66997548 |
| TPCN1 | two pore segment channel 1 | 0.00079357 | 1.607715525 | 23.54270689 | 23.5722974 | 37.56749723 | 38.18250833 |
| LOC440704 | hypothetical LOC440704 | 0.00217304 | 1.607498015 | 370.4477928 | 363.9001841 | 547.4385371 | 636.3188827 |
| ZSCAN29 | zinc finger and SCAN domain containing 29 | 0.00260051 | 1.607089154 | 62.34403949 | 50.43659832 | 91.15668222 | 89.090656 |
| RCSD1 | RCSD domain containing 1 | 0.01461814 | 1.604891128 | 594.4264567 | 557.4887343 | 919.6819955 | 928.0848731 |
| PKIG | protein kinase (cAMP-dependent. catalytic) inhibitor gamma | 0.02020829 | 1.604751075 | 22.71125994 | 25.59585523 | 44.12557597 | 33.92624833 |
| GPATCH4 | G patch domain containing 4 | 0.00372296 | 1.604486236 | 46.92410964 | 41.24238261 | 68.76534957 | 72.45062301 |
| NFKBIZ | nuclear factor of kappa light polypeptide gene enhancer in B-cells inhibitor. zeta | 0.01383348 | 1.603601891 | 16.04451528 | 18.05731479 | 23.66641605 | 31.48041107 |
| OPRL1 | opiate receptor-like 1 | 0.00107984 | 1.601426931 | 378.3735854 | 376.4640129 | 577.2944152 | 632.7922871 |
| NFAM1 | NFAT activating protein with ITAM motif 1 | 0.05825616 | 1.601355122 | 15.34424383 | 20.85387679 | 38.48730155 | 21.32014423 |
| SLC26A6 | solute carrier family 26. member 6 | 0.00242496 | 1.600725865 | 180.3836385 | 185.736688 | 323.6072576 | 265.2836669 |
| RASA3 | RAS p21 protein activator 3 | 0.00492527 | 1.599223374 | 39.22883456 | 30.69794882 | 52.40032704 | 58.77586425 |
| ZNF791 | zinc finger protein 791 | 0.00484752 | 1.598795882 | 97.50199455 | 96.99357234 | 136.5779837 | 176.9953269 |
| CHKA | choline kinase alpha | 0.00120492 | 1.598729645 | 441.9231916 | 398.0010131 | 662.5216732 | 678.5485662 |
| TNFSF10 | tumor necrosis factor (ligand) superfamily. member 10 | 0.00604294 | 1.598383187 | 32.44034511 | 27.29988196 | 48.17293455 | 46.96831034 |
| LOC729513 | SMG1 homolog. phosphatidylinositol 3-kinase-related kinase (C. elegans) pseudogene | 0.00833449 | 1.597984229 | 85.20300537 | 75.53215011 | 144.8106926 | 113.4830785 |
| LOC100130298 | hCG1816373-like | 0.03675926 | 1.597319693 | 28.86743695 | 20.37941165 | 31.87790942 | 47.08620917 |
| ALS2 | amyotrophic lateral sclerosis 2 (juvenile) | 0.00155494 | 1.596774478 | 136.8305671 | 137.1175129 | 207.4025326 | 230.6477189 |
| ITIH1 | inter-alpha (globulin) inhibitor H1 | 0.05706307 | 1.596657767 | 18.1112896 | 15.9756861 | 37.6063977 | 19.61421064 |
| RHOBTB2 | Rho-related BTB domain containing 2 | 0.01023759 | 1.596493991 | 70.0279817 | 49.85824221 | 101.3493265 | 87.80561359 |
| YTHDC1 | YTH domain containing 1 | 0.00750046 | 1.596202279 | 26.6672438 | 21.76568852 | 43.3430163 | 34.11986176 |
| SLC16A12 | solute carrier family 16. member 12 (monocarboxylic acid transporter 12) | 0.00337619 | 1.59586096 | 3850.085255 | 4594.589987 | 7060.054017 | 6381.153411 |
| TSPAN14 | tetraspanin 14 | 0.0189642 | 1.594966268 | 28.09929269 | 24.42708999 | 36.27557801 | 48.13442455 |
| MAGED2 | melanoma antigen family D. 2 | 0.03218621 | 1.593418267 | 22.48578707 | 20.19754477 | 26.65769497 | 43.25573166 |
| CD83 | CD83 molecule | 0.00281627 | 1.593087738 | 1054.581143 | 975.6449372 | 1494.823867 | 1746.872319 |
| MS4A7 | membrane-spanning 4-domains. subfamily A. member 7 | 0.03802346 | 1.593020134 | 20.49575623 | 23.77189157 | 44.43761333 | 27.82399533 |
| LRP5L | low density lipoprotein receptor-related protein 5-like | 0.00179337 | 1.592995515 | 172.3730613 | 199.985633 | 295.0938941 | 296.4401843 |
| DISP1 | dispatched homolog 1 (Drosophila) | 0.00483224 | 1.592465634 | 24.05128932 | 23.0407273 | 36.48718695 | 38.51539021 |
| EGLN1 | egl nine homolog 1 (C. elegans) | 0.01257972 | 1.591498928 | 29.84530601 | 32.71799867 | 42.41445436 | 58.31248948 |
| PARD6A | par-6 partitioning defective 6 homolog alpha (C. elegans) | 0.0057012 | 1.590953624 | 129.6454738 | 154.2340663 | 208.9156676 | 242.2599912 |
| GJC1 | gap junction protein. gamma 1. 45kDa | 0.00299076 | 1.590867773 | 6326.053597 | 6367.957175 | 9890.7054 | 10307.98797 |
| TIMM8A | translocase of inner mitochondrial membrane 8 homolog A (yeast) | 0.02054877 | 1.590459663 | 120.245724 | 80.79412665 | 176.6740462 | 139.0983555 |
| CHST15 | carbohydrate (N-acetylgalactosamine 4-sulfate 6-O) sulfotransferase 15 | 0.00311501 | 1.590203665 | 375.933703 | 367.1165885 | 553.0743794 | 631.011437 |
| MTRF1 | mitochondrial translational release factor 1 | 0.00336827 | 1.590115558 | 68.30639636 | 57.77850907 | 97.86323636 | 101.9683765 |
| LOC202781 | hypothetical LOC202781 | 0.01438964 | 1.589551809 | 851.8136572 | 1001.145886 | 1531.758013 | 1406.699006 |
| GRK5 | G protein-coupled receptor kinase 5 | 0.01352151 | 1.589325467 | 128.3171575 | 110.9319211 | 157.8055447 | 227.847722 |
| TNFRSF21 | tumor necrosis factor receptor superfamily. member 21 | 0.00120974 | 1.588782607 | 191.182035 | 189.9494627 | 307.0639804 | 298.5281076 |
| TAF4B | TAF4b RNA polymerase II. TATA box binding protein (TBP)-associated factor. 105kDa | 0.01620516 | 1.587720814 | 34.41966166 | 40.1296141 | 71.42798999 | 48.74739667 |
| DUSP3 | dual specificity phosphatase 3 | 0.00264039 | 1.5873691 | 51.51800229 | 49.87071529 | 73.80021804 | 87.72084579 |
| CXorf21 | chromosome X open reading frame 21 | 0.01326501 | 1.586996931 | 16.50895016 | 23.11767458 | 33.86626704 | 28.38235629 |
| RHBDD1 | rhomboid domain containing 1 | 0.00274509 | 1.586055322 | 100.4555228 | 88.03446952 | 159.8814946 | 139.144176 |
| CYP2E1 | cytochrome P450. family 2. subfamily E. polypeptide 1 | 0.0629073 | 1.586042281 | 20.90565152 | 14.88954347 | 37.38192249 | 20.94657289 |
| SNRNP35 | small nuclear ribonucleoprotein 35kDa (U11/U12) | 0.00366822 | 1.584944615 | 47.57374061 | 45.75934948 | 83.04111195 | 65.85400127 |
| ABCG1 | ATP-binding cassette. sub-family G (WHITE). member 1 | 0.00115374 | 1.584701596 | 123.9456056 | 133.6625352 | 206.9957818 | 200.9899413 |
| PDE4C | phosphodiesterase 4C. cAMP-specific | 0.01576977 | 1.584665325 | 4286.56157 | 5396.411654 | 7444.113125 | 7803.263696 |
| CACNB3 | calcium channel. voltage-dependent. beta 3 subunit | 0.00766965 | 1.584441448 | 101.6304993 | 76.24738538 | 149.5170993 | 130.1099595 |
| PMPCA | peptidase (mitochondrial processing) alpha | 0.00329389 | 1.584245589 | 447.2391327 | 449.0202978 | 655.5833649 | 768.8167881 |
| EID2B | EP300 interacting inhibitor of differentiation 2B | 0.01107587 | 1.584209019 | 24.82251619 | 28.11671127 | 49.61500964 | 35.30386118 |
| NARF | nuclear prelamin A recognition factor | 0.00149273 | 1.583119956 | 394.7093965 | 368.730266 | 601.1679062 | 606.7616333 |
| CAPS | calcyphosine | 0.0024554 | 1.582470757 | 103.8313104 | 100.7150808 | 158.6485484 | 165.0661888 |
| SNORD83B | small nucleolar RNA. C/D box 83B | 0.01692989 | 1.58211281 | 50.55476885 | 60.55998517 | 77.87412441 | 98.4078189 |
| PLXNB2 | plexin B2 | 0.00421728 | 1.581750078 | 349.1569688 | 279.6858513 | 479.5255873 | 509.5128658 |
| UBE2D4 | ubiquitin-conjugating enzyme E2D 4 (putative) | 0.00293343 | 1.581111539 | 142.3699505 | 151.2781181 | 229.7137189 | 234.3864661 |
| CYTH4 | cytohesin 4 | 0.00166442 | 1.580423135 | 146.1592914 | 132.2187199 | 209.9405233 | 229.9163512 |
| SEZ6L2 | seizure related 6 homolog (mouse)-like 2 | 0.01183577 | 1.578836645 | 19.42085716 | 19.15539591 | 36.66049587 | 25.29505263 |
| LOC439949 | hypothetical LOC439949 | 0.010423 | 1.578188787 | 44.08013772 | 46.1646106 | 68.25373539 | 74.25806042 |
| DHDH | dihydrodiol dehydrogenase (dimeric) | 0.00744327 | 1.577930334 | 21.96305195 | 19.97771161 | 38.37264199 | 28.47032183 |
| ZNF689 | zinc finger protein 689 | 0.00240929 | 1.577436388 | 166.4528509 | 169.8053194 | 270.3587979 | 260.1391587 |
| C15orf63 | chromosome 15 open reading frame 63 | 0.03587387 | 1.576846266 | 913.3445621 | 1085.213561 | 1318.702247 | 1868.881742 |
| RHOF | ras homolog gene family. member F (in filopodia) | 0.04627832 | 1.576518221 | 41.9200637 | 37.07688733 | 64.22319905 | 60.14939394 |
| SERGEF | secretion regulating guanine nucleotide exchange factor | 0.00439867 | 1.576432123 | 118.608342 | 150.7308731 | 204.3213854 | 217.4473788 |
| SNORD14C | small nucleolar RNA. C/D box 14C | 0.0033038 | 1.576320647 | 27.88991843 | 25.11174977 | 44.51425089 | 39.09437532 |
| TLCD1 | TLC domain containing 1 | 0.01610929 | 1.576039965 | 25.34157501 | 30.35420623 | 36.90348853 | 51.77492917 |
| ANAPC11 | anaphase promoting complex subunit 11 | 0.00430879 | 1.5757334 | 101.8499132 | 126.4554527 | 172.7100149 | 185.1595786 |
| NBPF24 | neuroblastoma breakpoint family. member 24 | 0.02893853 | 1.575577812 | 35.02705185 | 39.17410483 | 60.901496 | 55.93123619 |
| LOC646936 | C-terminal binding protein 2 pseudogene | 0.0237626 | 1.574886063 | 107.440252 | 145.3806563 | 233.339042 | 166.0292142 |
| IQCG | IQ motif containing G | 0.01213356 | 1.574400107 | 25.43964143 | 24.86589546 | 34.26616238 | 45.75934948 |
| EIF1AX | eukaryotic translation initiation factor 1A. X-linked | 0.00658638 | 1.57385801 | 180.8077097 | 166.0739372 | 306.7890384 | 242.4430204 |
| KBTBD6 | kelch repeat and BTB (POZ) domain containing 6 | 0.03431245 | 1.573705709 | 27.64907155 | 31.61558774 | 37.0924688 | 58.36373981 |
| C7orf50 | chromosome 7 open reading frame 50 | 0.00190167 | 1.573701391 | 1754.233241 | 1815.807534 | 2805.991508 | 2811.353486 |
| IFI6 | interferon. alpha-inducible protein 6 | 0.04050099 | 1.573491822 | 46.72553902 | 58.69809402 | 96.02621791 | 70.71596554 |
| RAB11FIP5 | RAB11 family interacting protein 5 (class I) | 0.00483067 | 1.573231286 | 40.11056927 | 39.51393047 | 55.09880777 | 71.19541143 |
| LOC728975 | hypothetical protein LOC728975 | 0.00914134 | 1.573139641 | 35.41743387 | 46.85404475 | 61.99733526 | 66.24082092 |
| SNORA26 | small nucleolar RNA. H/ACA box 26 | 0.00182861 | 1.572790228 | 28.69602238 | 28.89175324 | 42.35692855 | 48.41865743 |
| ART1 | ADP-ribosyltransferase 1 | 0.06318149 | 1.572736018 | 34.71811349 | 24.08741899 | 59.93765964 | 34.51105772 |
| MRPL42P5 | mitochondrial ribosomal protein L42 pseudogene 5 | 0.04036305 | 1.572724139 | 25.78862262 | 23.21769542 | 45.0885913 | 32.84624251 |
| NSMCE2 | non-SMC element 2. MMS21 homolog (S. cerevisiae) | 0.00560769 | 1.572702905 | 140.1558021 | 161.7399048 | 212.4215673 | 263.9508309 |
| SLC25A23 | solute carrier family 25 (mitochondrial carrier; phosphate carrier). member 23 | 0.00269657 | 1.572415941 | 514.4702164 | 615.0265877 | 858.9723708 | 910.7722914 |
| ZNF775 | zinc finger protein 775 | 0.0166195 | 1.571940746 | 88.78853511 | 79.08371266 | 115.2761219 | 150.5140097 |
| LOC25845 | hypothetical LOC25845 | 0.00678053 | 1.571774266 | 91.07797283 | 84.3723941 | 159.0539318 | 119.357487 |
| GMPR2 | guanosine monophosphate reductase 2 | 0.01845442 | 1.571596618 | 26.24155205 | 18.42593659 | 30.56186159 | 39.07702173 |
| GCET2 | germinal center expressed transcript 2 | 0.00298983 | 1.570408089 | 153.9828397 | 139.9896602 | 221.079116 | 240.4615331 |
| CD40 | CD40 molecule. TNF receptor superfamily member 5 | 0.0067604 | 1.570248845 | 90.85748133 | 88.78853511 | 161.0687833 | 123.4932361 |
| EHD1 | EH-domain containing 1 | 0.00113658 | 1.56979281 | 1642.316644 | 1565.742313 | 2515.29097 | 2519.26366 |
| SCARNA12 | small Cajal body-specific RNA 12 | 0.00476939 | 1.568615745 | 38.45495838 | 38.87471916 | 64.3378436 | 57.17237706 |
| PLIN3 | perilipin 3 | 0.00264023 | 1.568465211 | 1117.952741 | 1291.642369 | 1976.764607 | 1797.051619 |
| SNORD17 | small nucleolar RNA. C/D box 17 | 0.02363324 | 1.567346104 | 24.511859 | 35.57975771 | 47.10814131 | 45.47922805 |
| ADD3 | adducin 3 (gamma) | 0.00207951 | 1.567157419 | 282.4865193 | 309.134464 | 468.1293139 | 458.1466988 |
| SNORD15B | small nucleolar RNA. C/D box 15B | 0.01700465 | 1.566921573 | 23.10673766 | 30.94124002 | 40.26003092 | 43.60103156 |
| MT1X | metallothionein 1X | 0.00223703 | 1.565833353 | 164.4562869 | 178.1747857 | 250.0131816 | 287.3590622 |
| LOC645895 | hypothetical LOC645895 | 0.00143823 | 1.56530754 | 7258.36957 | 7489.816302 | 11087.68733 | 12013.4744 |
| OR13A1 | olfactory receptor. family 13. subfamily A. member 1 | 0.01257526 | 1.564910065 | 46.34408885 | 45.15058083 | 59.65872879 | 85.89392763 |
| LOC729086 | similar to EGFR-coamplified and overexpressed protein | 0.002003 | 1.564790046 | 598.2704371 | 523.6600959 | 899.3394102 | 852.9735195 |
| CENPB | centromere protein B. 80kDa | 0.00391096 | 1.564666959 | 495.76432 | 576.8562115 | 789.9731766 | 886.2868772 |
| CCNY | cyclin Y | 0.00995126 | 1.564133282 | 282.7963064 | 365.2298892 | 504.3019063 | 501.0683241 |
| PPP1R12B | protein phosphatase 1. regulatory (inhibitor) subunit 12B | 0.0885891 | 1.563917918 | 36.743227 | 37.32110851 | 47.35304539 | 70.82911683 |
| MIF4GD | MIF4G domain containing | 0.00518408 | 1.563739467 | 217.5154025 | 206.6920922 | 376.5816325 | 291.9332334 |
| RGS2 | regulator of G-protein signaling 2. 24kDa | 0.01252405 | 1.563501746 | 108.8512449 | 135.083533 | 199.4787166 | 180.1921996 |
| ATP6V1H | ATPase. H+ transporting. lysosomal 50/57kDa. V1 subunit H | 0.02468782 | 1.563484211 | 30.20329602 | 19.94667713 | 40.50416753 | 36.35902143 |
| UBN2 | ubinuclein 2 | 0.0020754 | 1.563243772 | 31.88928185 | 33.47817939 | 54.04697061 | 48.27125934 |
| C3orf34 | chromosome 3 open reading frame 34 | 0.02854112 | 1.563225721 | 40.36652514 | 47.51859888 | 71.40005279 | 65.64926131 |
| TCEA2 | transcription elongation factor A (SII). 2 | 0.00131032 | 1.561942208 | 380.4305313 | 393.8783251 | 623.9081596 | 585.9313057 |
| LASS4 | LAG1 homolog. ceramide synthase 4 | 0.02053909 | 1.561769868 | 28.10114844 | 37.11997114 | 45.13986073 | 56.36448759 |
| CD82 | CD82 molecule | 0.00291118 | 1.561320318 | 121.0030305 | 122.3710765 | 174.7219345 | 206.5911062 |
| MYC | v-myc myelocytomatosis viral oncogene homolog (avian) | 0.00948544 | 1.560987274 | 778.2271097 | 573.4128385 | 1079.350382 | 1007.418782 |
| LEPROT | leptin receptor overlapping transcript | 0.00585448 | 1.560696536 | 91.37760907 | 80.63734846 | 131.4335496 | 136.5547228 |
| PCBD1 | pterin-4 alpha-carbinolamine dehydratase/dimerization cofactor of hepatocyte nuclear factor 1 alpha | 0.03126196 | 1.56023693 | 76.68989737 | 54.94266332 | 100.4555228 | 102.1069145 |
| LOC202781 | hypothetical LOC202781 | 0.00388417 | 1.55983938 | 60.71227872 | 53.81830215 | 97.90263884 | 81.20296537 |
| STAG3L2 | stromal antigen 3-like 2 | 0.00494535 | 1.559509808 | 352.1188722 | 409.516511 | 628.1808707 | 558.2802093 |
| PPP2R4 | protein phosphatase 2A activator. regulatory subunit 4 | 0.25126152 | 1.559464299 | 31.60893191 | 15.78755144 | 23.47227602 | 51.70353891 |
| PNPT1 | polyribonucleotide nucleotidyltransferase 1 | 0.00375554 | 1.558512792 | 4169.457624 | 4574.059644 | 6312.033428 | 7338.931597 |
| SAMD9 | sterile alpha motif domain containing 9 | 0.00955653 | 1.55849438 | 168.34488 | 125.0396565 | 229.9845059 | 222.3103012 |
| C19orf12 | chromosome 19 open reading frame 12 | 0.00205535 | 1.557402294 | 264.522208 | 249.5425184 | 408.2626311 | 392.1648663 |
| SP140 | SP140 nuclear body protein | 0.00212301 | 1.557377118 | 116.8043322 | 129.7749046 | 182.9720747 | 200.9335401 |
| DNAJC12 | DnaJ (Hsp40) homolog. subfamily C. member 12 | 0.00227275 | 1.556375064 | 443.598157 | 475.0437231 | 756.7903703 | 674.4911379 |
| IFIT2 | interferon-induced protein with tetratricopeptide repeats 2 | 0.00632705 | 1.555513579 | 69.38586824 | 74.66288433 | 120.6932374 | 103.8581225 |
| MT1F | metallothionein 1F | 0.00361106 | 1.555126498 | 149.6895904 | 133.0950352 | 235.0745007 | 204.9648437 |
| NPC2 | Niemann-Pick disease. type C2 | 0.00127162 | 1.554864391 | 923.8387541 | 943.7070114 | 1420.706457 | 1483.590484 |
| RNF213 | ring finger protein 213 | 0.03432054 | 1.55484085 | 38.03272326 | 51.38648602 | 63.71973242 | 74.14882691 |
| LOC728734 | similar to NPIP-like protein ENSP00000283050 | 0.0302112 | 1.554753169 | 27.66297119 | 30.52131533 | 57.56516424 | 35.4539986 |
| NACA2 | nascent polypeptide-associated complex alpha subunit 2 | 0.05628282 | 1.554282568 | 22.86345619 | 24.04956688 | 39.91163359 | 33.28201328 |
| LRIG1 | leucine-rich repeats and immunoglobulin-like domains 1 | 0.00362534 | 1.554108726 | 77.5711817 | 63.98287084 | 107.8905864 | 111.1074995 |
| RPPH1 | ribonuclease P RNA component H1 | 0.00826875 | 1.55369106 | 27.77819572 | 23.97914311 | 35.6957465 | 45.04541142 |
| PRKCB | protein kinase C. beta | 0.00208189 | 1.553536415 | 377.4977828 | 422.3088268 | 600.003896 | 641.258846 |
| SIAH1 | seven in absentia homolog 1 (Drosophila) | 0.05242665 | 1.55302788 | 28.16609497 | 50.98059424 | 56.78055732 | 60.99445938 |
| PDXK | pyridoxal (pyridoxine. vitamin B6) kinase | 0.00140676 | 1.552542516 | 432.7256233 | 428.3956225 | 680.8651786 | 656.2714586 |
| SLC25A37 | solute carrier family 25. member 37 | 0.01536543 | 1.551774432 | 131.7963757 | 149.877842 | 182.5855063 | 260.514429 |
| LOC646808 | similar to L antigen family. member 3 | 0.00500696 | 1.551616132 | 35.2051303 | 43.62094171 | 64.10842334 | 57.6706 |
| MT2A | metallothionein 2A | 0.03942606 | 1.551443411 | 48.47202446 | 42.37257354 | 77.26523983 | 63.98287084 |
| C1orf38 | chromosome 1 open reading frame 38 | 0.09898264 | 1.551423976 | 37.37224234 | 35.2051303 | 68.89093271 | 45.96783454 |
| ZNF121 | zinc finger protein 121 | 0.03485151 | 1.551179371 | 30.70826872 | 21.65019931 | 39.43566092 | 40.56506241 |
| PRKX | protein kinase. X-linked | 0.00490854 | 1.551154481 | 51.70971117 | 53.80874555 | 75.93427383 | 88.16520852 |
| C7orf27 | chromosome 7 open reading frame 27 | 0.00144822 | 1.550467658 | 830.7255173 | 807.8020147 | 1231.601946 | 1309.83786 |
| ABLIM1 | actin binding LIM protein 1 | 0.00643089 | 1.550268501 | 148.9147585 | 146.8191984 | 224.7013808 | 233.8453264 |
| ADI1 | acireductone dioxygenase 1 | 0.11071814 | 1.549871717 | 36.07122372 | 26.67079714 | 69.04497932 | 33.4700433 |
| C9orf72 | chromosome 9 open reading frame 72 | 0.01247111 | 1.549843351 | 23.87605375 | 32.05822127 | 45.73410567 | 40.20104925 |
| IL16 | interleukin 16 (lymphocyte chemoattractant factor) | 0.00342644 | 1.549041637 | 179.5340923 | 188.6523106 | 260.1391587 | 312.4132975 |
| FAM162A | family with sequence similarity 162. member A | 0.01176063 | 1.548967125 | 77.04273399 | 78.72948218 | 140.7032717 | 103.4306577 |
| TLR6 | toll-like receptor 6 | 0.0267109 | 1.548707421 | 30.2273959 | 20.39734761 | 34.14185809 | 43.3137751 |
| GOLGA8A | golgin A8 family. member A | 0.03526143 | 1.548669491 | 30.40419077 | 23.71712488 | 42.1033767 | 41.07674739 |
| SMPDL3B | sphingomyelin phosphodiesterase. acid-like 3B | 0.00437865 | 1.548011236 | 40.42937481 | 35.78038975 | 64.71970821 | 53.56162681 |
| EZR | ezrin | 0.00146708 | 1.547672785 | 3703.337363 | 3497.335641 | 5497.555445 | 5643.119721 |
| CDAN1 | congenital dyserythropoietic anemia. type I | 0.01107587 | 1.54758615 | 2809.149295 | 3460.321629 | 4693.508503 | 4960.247514 |
| NXF1 | nuclear RNA export factor 1 | 0.00187132 | 1.547291856 | 369.0793223 | 332.2491524 | 529.8728641 | 554.059488 |
| MED29 | mediator complex subunit 29 | 0.00621062 | 1.546547622 | 200.6161215 | 190.3304003 | 266.767396 | 342.3480327 |
| LOC100131096 | hypothetical LOC100131096 | 0.07570507 | 1.546186918 | 17.65656157 | 18.64833724 | 39.32459287 | 20.01732322 |
| ZNF786 | zinc finger protein 786 | 0.00145146 | 1.54616685 | 272.8419867 | 276.6148939 | 414.959842 | 434.8038787 |
| FAM120B | family with sequence similarity 120B | 0.00252145 | 1.545288945 | 301.1983035 | 346.2756905 | 507.3134449 | 490.9277124 |
| PHLDA1 | pleckstrin homology-like domain. family A. member 1 | 0.00303276 | 1.544858462 | 17.62666512 | 18.67971715 | 25.81985656 | 30.434349 |
| MYOM1 | myomesin 1. 185kDa | 0.00371025 | 1.544613188 | 19.58033649 | 20.52224654 | 28.69189998 | 33.41375009 |
| ZNF69 | zinc finger protein 69 | 0.00712602 | 1.54457112 | 162.5118787 | 139.4608831 | 228.699964 | 236.4216512 |
| NCF1 | neutrophil cytosolic factor 1 | 0.00585422 | 1.544430502 | 799.5575074 | 962.9082661 | 1474.839032 | 1245.164522 |
| OVGP1 | oviductal glycoprotein 1. 120kDa | 0.02600579 | 1.544035104 | 58.66715517 | 77.93235324 | 108.3715956 | 100.5800141 |
| PPP1R16B | protein phosphatase 1. regulatory (inhibitor) subunit 16B | 0.00873295 | 1.543991376 | 220.0822947 | 235.4367601 | 333.7748386 | 370.0799224 |
| C19orf44 | chromosome 19 open reading frame 44 | 0.00696011 | 1.54389171 | 48.19416595 | 45.84786118 | 70.34319041 | 74.87298674 |
| RANBP2 | RAN binding protein 2 | 0.03812379 | 1.543352366 | 29.31539918 | 39.88345065 | 42.96571629 | 64.8181551 |
| BLVRB | biliverdin reductase B (flavin reductase (NADPH)) | 0.00268942 | 1.54313805 | 75.13021946 | 68.89093271 | 118.3491903 | 104.1408196 |
| FCGR3A | Fc fragment of IgG. low affinity IIIa. receptor (CD16a) | 0.04724692 | 1.542999257 | 22.08072097 | 23.32035808 | 28.39051294 | 43.18238838 |
| SNORA5B | small nucleolar RNA. H/ACA box 5B | 0.00679895 | 1.542009834 | 19.55746987 | 24.9616893 | 36.22329265 | 32.04593927 |
| LOC730173 | similar to ubiquitin-conjugating enzyme E2C | 0.0104573 | 1.541958344 | 25.78012882 | 22.73589528 | 32.11390046 | 43.39596691 |
| CCDC21 | coiled-coil domain containing 21 | 0.00198111 | 1.54096115 | 161.5234533 | 178.4087709 | 261.2042509 | 261.9720334 |
| DPYD | dihydropyrimidine dehydrogenase | 0.01633091 | 1.540640509 | 18.49360135 | 19.89533275 | 34.36829753 | 25.41073956 |
| VHL | von Hippel-Lindau tumor suppressor | 0.01161629 | 1.540512802 | 156.5292971 | 120.3681128 | 229.5353486 | 194.7996326 |
| SLC2A6 | solute carrier family 2 (facilitated glucose transporter). member 6 | 0.00212016 | 1.540389749 | 450.6971631 | 488.0683833 | 689.6855737 | 756.7903703 |
| BIK | BCL2-interacting killer (apoptosis-inducing) | 0.00245269 | 1.54013941 | 181.7216507 | 179.5340923 | 261.678031 | 295.7375073 |
| BTG1 | B-cell translocation gene 1. anti-proliferative | 0.00311181 | 1.539743466 | 2487.637351 | 2133.32178 | 3566.170846 | 3528.076802 |
| MARCH2 | membrane-associated ring finger (C3HC4) 2 | 0.0031345 | 1.539726624 | 39.94560405 | 33.68190014 | 57.96204731 | 55.03121462 |
| APBB3 | amyloid beta (A4) precursor protein-binding. family B. member 3 | 0.00282283 | 1.539635682 | 260.1391587 | 250.8731263 | 426.177289 | 362.999065 |
| SNORD104 | small nucleolar RNA. C/D box 104 | 0.00322192 | 1.539325562 | 236.7566762 | 269.8495731 | 398.9554225 | 379.4552449 |
| IL25 | interleukin 25 | 0.01155212 | 1.539034161 | 103.1372696 | 126.7744038 | 178.5084877 | 173.4941578 |
| GPC2 | glypican 2 | 0.00375319 | 1.538971793 | 132.2932407 | 121.3392802 | 195.779062 | 194.193257 |
| CDKN2A | cyclin-dependent kinase inhibitor 2A (melanoma. p16. inhibits CDK4) | 0.00660829 | 1.538668866 | 187.867436 | 162.1934902 | 267.5286478 | 269.6528194 |
| VKORC1L1 | vitamin K epoxide reductase complex. subunit 1-like 1 | 0.00703022 | 1.538648994 | 150.5684666 | 133.633579 | 209.6456872 | 227.2180401 |
| PTP4A3 | protein tyrosine phosphatase type IVA. member 3 | 0.02027105 | 1.538390503 | 174.0859532 | 159.4233741 | 302.4235509 | 217.1867358 |
| PLEKHA4 | pleckstrin homology domain containing. family A (phosphoinositide binding specific) member 4 | 0.00148133 | 1.538219399 | 48.13442455 | 49.0736023 | 74.3046844 | 75.21840106 |
| PDK3 | pyruvate dehydrogenase kinase. isozyme 3 | 0.00237684 | 1.538075716 | 348.2095722 | 304.0431168 | 492.6556858 | 508.3792463 |
| P4HTM | prolyl 4-hydroxylase. transmembrane (endoplasmic reticulum) | 0.00347729 | 1.537480881 | 175.017961 | 146.7575381 | 250.1496259 | 242.7183594 |
| ANKLE1 | ankyrin repeat and LEM domain containing 1 | 0.00708691 | 1.537093918 | 145.945983 | 148.9416331 | 200.4137274 | 256.2604594 |
| RAB11FIP3 | RAB11 family interacting protein 3 (class II) | 0.0023412 | 1.536542457 | 315.0317145 | 336.2094567 | 514.8144523 | 485.7385828 |
| SLC29A1 | solute carrier family 29 (nucleoside transporters). member 1 | 0.00302166 | 1.536338017 | 310.7600866 | 290.7942425 | 476.2133737 | 447.9020025 |
| PARP4 | poly (ADP-ribose) polymerase family. member 4 | 0.0020122 | 1.536333435 | 776.0420009 | 851.8136572 | 1286.091723 | 1213.19007 |
| LUC7L | LUC7-like (S. cerevisiae) | 0.01406422 | 1.5357939 | 23.75263621 | 20.22660919 | 28.66636365 | 39.53012353 |
| GLMN | glomulin. FKBP associated protein | 0.00384932 | 1.535633839 | 242.7932375 | 233.0074684 | 402.5311833 | 331.4226953 |
| RPL23AP32 | ribosomal protein L23a pseudogene 32 | 0.00241808 | 1.535584293 | 25.04878277 | 23.30203473 | 35.52037195 | 38.74808904 |
| TGFBRAP1 | transforming growth factor. beta receptor associated protein 1 | 0.08477396 | 1.535428579 | 50.2227297 | 85.54438658 | 108.9608171 | 92.95670445 |
| SAP30L | SAP30-like | 0.0056272 | 1.535055063 | 352.8596072 | 335.7637832 | 586.249014 | 476.2133737 |
| IL11RA | interleukin 11 receptor. alpha | 0.00242867 | 1.534527489 | 129.0964911 | 119.0080157 | 199.1353605 | 181.6735126 |
| C19orf28 | chromosome 19 open reading frame 28 | 0.03820097 | 1.534314328 | 28.05042937 | 34.37570414 | 46.3339158 | 48.99150628 |
| DAPP1 | dual adaptor of phosphotyrosine and 3-phosphoinositides | 0.0048181 | 1.53411265 | 2278.494902 | 2819.132264 | 3890.171091 | 3886.058312 |
| GNRH1 | gonadotropin-releasing hormone 1 (luteinizing-releasing hormone) | 0.0148243 | 1.533680076 | 27.24986507 | 21.74224438 | 34.20096322 | 40.74740377 |
| TRIM69 | tripartite motif-containing 69 | 0.02671652 | 1.533284947 | 18.58794673 | 23.39008892 | 26.67695171 | 38.31535324 |
| CDC42EP4 | CDC42 effector protein (Rho GTPase binding) 4 | 0.01620625 | 1.53321393 | 25.91350966 | 24.71287695 | 44.50306484 | 33.82712867 |
| LOC100131076 | hypothetical LOC100131076 | 0.03425311 | 1.533045182 | 22.18052546 | 17.50315746 | 37.53024547 | 24.31177873 |
| PBX4 | pre-B-cell leukemia homeobox 4 | 0.09370344 | 1.532899523 | 26.64486585 | 26.32586971 | 45.98275824 | 35.84500335 |
| FBXO7 | F-box protein 7 | 0.04966979 | 1.532682025 | 50.29605727 | 61.72924003 | 96.04862599 | 75.93427383 |
| HCFC1R1 | host cell factor C1 regulator 1 (XPO1 dependent) | 0.0380773 | 1.531566239 | 41.95278759 | 52.08457778 | 56.74413407 | 90.32761993 |
| SERPINI1 | serpin peptidase inhibitor. clade I (neuroserpin). member 1 | 0.00215476 | 1.531491481 | 258.4606993 | 233.7655314 | 364.9175541 | 388.3375673 |
| PA2G4P4 | proliferation-associated 2G4 pseudogene 4 | 0.06383735 | 1.531488007 | 17.093506 | 18.51705672 | 33.58954062 | 22.10172861 |
| LOC728537 | hypothetical protein LOC728537 | 0.02628167 | 1.531233754 | 28.4833903 | 28.68981654 | 36.97239823 | 51.8232706 |
| MIB2 | mindbomb homolog 2 (Drosophila) | 0.00941404 | 1.531130398 | 109.4878947 | 136.8305671 | 175.3909915 | 200.2471325 |
| MLL5 | myeloid/lymphoid or mixed-lineage leukemia 5 (trithorax homolog. Drosophila) | 0.07164551 | 1.531113018 | 30.50294149 | 16.77319236 | 31.68995522 | 37.84864375 |
| C16orf93 | chromosome 16 open reading frame 93 | 0.00161748 | 1.530982628 | 352.9742637 | 368.7805719 | 543.6480504 | 561.2208082 |
| LOC100128505 | hypothetical LOC100128505 | 0.00525977 | 1.530915971 | 5069.331213 | 5987.047251 | 8146.43076 | 8731.697667 |
| TNFRSF13C | tumor necrosis factor receptor superfamily. member 13C | 0.01574811 | 1.530767127 | 70.77098979 | 74.7194384 | 119.0692491 | 104.0656757 |
| RHOC | ras homolog gene family. member C | 0.00157634 | 1.530466632 | 485.7385828 | 507.3134449 | 766.3418384 | 753.1893453 |
| CLN3 | ceroid-lipofuscinosis. neuronal 3 | 0.00422246 | 1.530257308 | 33.40614829 | 40.8344871 | 55.74856003 | 57.29922923 |
| KIAA1024 | KIAA1024 | 0.00612493 | 1.52996443 | 31.45124964 | 24.78423563 | 43.38576136 | 42.05608873 |
| ULK1 | unc-51-like kinase 1 (C. elegans) | 0.00158797 | 1.529958366 | 337.0724961 | 331.3648111 | 502.7708879 | 520.0185152 |
| GRK4 | G protein-coupled receptor kinase 4 | 0.00885873 | 1.529400295 | 43.13864728 | 40.30331111 | 59.3470004 | 68.52527953 |
| ZNF562 | zinc finger protein 562 | 0.0145954 | 1.529350102 | 59.53441329 | 65.54725256 | 98.5888597 | 92.57816326 |
| SPATA7 | spermatogenesis associated 7 | 0.02165148 | 1.529120751 | 74.88252941 | 60.06952744 | 99.94622593 | 105.232984 |
| QSOX1 | quiescin Q6 sulfhydryl oxidase 1 | 0.00229033 | 1.52903503 | 233.5975565 | 218.2058607 | 353.7534605 | 336.8750758 |
| MRPL10 | mitochondrial ribosomal protein L10 | 0.00915667 | 1.528611555 | 299.9662006 | 258.4606993 | 451.2981732 | 401.4186425 |
| PCDHB19P | protocadherin beta 19 pseudogene | 0.04654791 | 1.528551105 | 48.48935598 | 73.5350904 | 87.33761798 | 95.38929314 |
| PRRG2 | proline rich Gla (G-carboxyglutamic acid) 2 | 0.01198026 | 1.528475068 | 25.5939657 | 20.013844 | 35.82664286 | 33.40247884 |
| ZBTB45 | zinc finger and BTB domain containing 45 | 0.02341909 | 1.528273108 | 53.3770932 | 42.70051625 | 71.91145989 | 74.02729524 |
| ACCN3 | amiloride-sensitive cation channel 3 | 0.0026538 | 1.527310953 | 20.20697973 | 19.5627213 | 28.68555227 | 32.14566323 |
| RAGE | renal tumor antigen | 0.00926835 | 1.527142069 | 58.82082125 | 54.42032382 | 75.33903786 | 99.09027138 |
| MAPKAPK3 | mitogen-activated protein kinase-activated protein kinase 3 | 0.00541619 | 1.527028421 | 318.2665904 | 314.8028242 | 443.428477 | 526.8661922 |
| SNORD34 | small nucleolar RNA. C/D box 34 | 0.02013225 | 1.526592445 | 28.48215618 | 19.47748309 | 38.30950856 | 33.74778985 |
| KCTD6 | potassium channel tetramerisation domain containing 6 | 0.07849411 | 1.526406417 | 73.20856562 | 46.6588516 | 101.3933556 | 78.49225628 |
| CASZ1 | castor zinc finger 1 | 0.00290239 | 1.525909483 | 232.480109 | 248.7196256 | 355.4927972 | 378.7237967 |
| ZNF430 | zinc finger protein 430 | 0.01040985 | 1.525592316 | 4087.605444 | 4326.388185 | 5917.393473 | 6955.702357 |
| PLOD1 | procollagen-lysine 1. 2-oxoglutarate 5-dioxygenase 1 | 0.00335923 | 1.525050255 | 425.89008 | 469.1517344 | 668.2363618 | 695.4230111 |
| AAK1 | AP2 associated kinase 1 | 0.03199071 | 1.525020609 | 19.30600838 | 28.77556823 | 36.21648421 | 35.67479912 |
| CD53 | CD53 molecule | 0.00202196 | 1.524567148 | 924.3501895 | 931.1975412 | 1391.523785 | 1437.741589 |
| MAGED1 | melanoma antigen family D. 1 | 0.00193984 | 1.524516323 | 1235.255456 | 1352.130608 | 1970.242991 | 1970.242991 |
| SLAIN2 | SLAIN motif family. member 2 | 0.01393503 | 1.524385746 | 26.96980461 | 28.65592591 | 43.39797469 | 41.38210151 |
| GRAMD4 | GRAM domain containing 4 | 0.00182822 | 1.523985862 | 134.9721164 | 130.2883702 | 196.1916794 | 208.1761635 |
| CALN1 | calneuron 1 | 0.06555126 | 1.523281818 | 16.93678989 | 18.49296643 | 25.40234361 | 28.61043177 |
| STRC | stereocilin | 0.08226283 | 1.523217917 | 21.4640069 | 21.23310676 | 38.36172184 | 27.56451337 |
| LOC100133402 | similar to hCG2012269 | 0.03621508 | 1.522751607 | 16.75630943 | 24.69757777 | 35.94592824 | 26.69569078 |
| CERK | ceramide kinase | 0.00314613 | 1.522511942 | 483.8247838 | 432.4695491 | 720.6158273 | 673.0715967 |
| CDHR3 | cadherin-related family member 3 | 0.02359627 | 1.522509468 | 17.36433463 | 19.41716696 | 22.79795116 | 34.28216152 |
| ADHFE1 | alcohol dehydrogenase. iron containing. 1 | 0.0060349 | 1.522413995 | 25.25279714 | 26.51149807 | 35.64448572 | 43.532834 |
| RNF216 | ring finger protein 216 | 0.00308312 | 1.52233847 | 378.8337714 | 397.2788749 | 616.754678 | 565.5280517 |
| DUS2L | dihydrouridine synthase 2-like. SMM1 homolog (S. cerevisiae) | 0.00442637 | 1.522229444 | 182.6972384 | 172.750218 | 256.7623707 | 284.8258789 |
| UBA7 | ubiquitin-like modifier activating enzyme 7 | 0.04179102 | 1.522056423 | 53.56162681 | 43.91313072 | 72.95541046 | 74.6882285 |
| TSHZ1 | teashirt zinc finger homeobox 1 | 0.00782256 | 1.520828589 | 69.88578888 | 79.35525833 | 118.4844241 | 108.2589613 |
| ANKRD44 | ankyrin repeat domain 44 | 0.03188982 | 1.520197974 | 27.99334765 | 28.37314759 | 39.57463288 | 46.38160352 |
| ZNF91 | zinc finger protein 91 | 0.00394639 | 1.519891022 | 108.1049679 | 99.44879528 | 168.8992038 | 147.0423638 |
| ZNF346 | zinc finger protein 346 | 0.04468251 | 1.519411997 | 37.39878384 | 38.9156647 | 47.11803762 | 71.3092453 |
| TRIM4 | tripartite motif-containing 4 | 0.03142809 | 1.519389647 | 37.83646867 | 25.43051577 | 51.57472069 | 43.06923975 |
| GNB5 | guanine nucleotide binding protein (G protein). beta 5 | 0.05492874 | 1.51922877 | 98.45948709 | 73.00128631 | 134.7648132 | 123.0999624 |
| SMAD7 | SMAD family member 7 | 0.0020955 | 1.519179513 | 23.43447045 | 22.41425004 | 35.85014754 | 33.8147826 |
| CD84 | CD84 molecule | 0.00320146 | 1.519083272 | 75.02347589 | 65.92036254 | 109.1522226 | 104.5556119 |
| HEXIM2 | hexamthylene bis-acetamide inducible 2 | 0.03397189 | 1.518888432 | 31.47007716 | 25.66593884 | 51.85415825 | 35.93545294 |
| LOC678655 | hypothetical locus LOC678655 | 0.00907203 | 1.51808628 | 72.56889986 | 73.72509306 | 111.7017847 | 110.3821039 |
| FBXL15 | F-box and leucine-rich repeat protein 15 | 0.00908958 | 1.517908339 | 142.6427302 | 132.9103559 | 228.2943704 | 191.3393767 |
| EXT2 | exostosin 2 | 0.02601759 | 1.517848374 | 73.21981646 | 63.2561117 | 125.7125251 | 84.88077927 |
| CD79B | CD79b molecule. immunoglobulin-associated beta | 0.00582385 | 1.516508387 | 3234.14191 | 2822.632561 | 5009.555632 | 4190.866711 |
| IL23A | interleukin 23. alpha subunit p19 | 0.02145854 | 1.51639398 | 23.28288188 | 21.40396258 | 30.47608592 | 37.60069151 |
| IFT20 | intraflagellar transport 20 homolog (Chlamydomonas) | 0.00937511 | 1.515886424 | 127.0689968 | 141.0064743 | 200.8366685 | 205.0071337 |
| HEY1 | hairy/enhancer-of-split related with YRPW motif 1 | 0.02257611 | 1.51544502 | 27.63134372 | 22.92830732 | 33.09280193 | 43.96639219 |
| RRAS | related RAS viral (r-ras) oncogene homolog | 0.00863959 | 1.515434083 | 95.62881702 | 114.4885599 | 159.9237317 | 157.2215457 |
| REPS2 | RALBP1 associated Eps domain containing 2 | 0.07242804 | 1.514882084 | 16.48635863 | 20.32347124 | 20.96697881 | 36.67283061 |
| ALDOC | aldolase C. fructose-bisphosphate | 0.00232723 | 1.514268567 | 628.5091206 | 612.8944891 | 939.107438 | 940.5628808 |
| ZSCAN5A | zinc finger and SCAN domain containing 5A | 0.03567608 | 1.514058646 | 46.09805459 | 32.94868098 | 59.81085447 | 58.2138094 |
| DPEP2 | dipeptidase 2 | 0.00409662 | 1.513989849 | 21.11174797 | 20.19917253 | 31.36816894 | 31.16122555 |
| SYT15 | synaptotagmin XV | 0.00322853 | 1.513797963 | 39.00255439 | 41.07674739 | 56.44486205 | 65.04299272 |
| ZYG11B | zyg-11 homolog B (C. elegans) | 0.00363185 | 1.513738094 | 221.4139656 | 243.880709 | 338.8676947 | 365.1352582 |
| CDK6 | cyclin-dependent kinase 6 | 0.00182249 | 1.513536439 | 603.6339309 | 589.1419955 | 909.3257265 | 895.9007692 |
| LOC100130827 | similar to Putative uncharacterized serine/threonine-protein kinase SgK110 | 0.05251856 | 1.513254636 | 19.69703252 | 33.8485103 | 38.04054082 | 40.13448601 |
| CAMK4 | calcium/calmodulin-dependent protein kinase IV | 0.00460022 | 1.513113555 | 21.92961953 | 21.63765187 | 34.26295408 | 31.7073149 |
| ATP2A3 | ATPase. Ca++ transporting. ubiquitous | 0.00214962 | 1.512966216 | 103.4306577 | 109.0692388 | 157.2645254 | 164.2023094 |
| MRPL44 | mitochondrial ribosomal protein L44 | 0.00473748 | 1.512849007 | 264.8611868 | 233.9406623 | 406.9055828 | 348.5150656 |
| TNRC6B | trinucleotide repeat containing 6B | 0.05362607 | 1.512368301 | 25.48162601 | 32.69860629 | 49.99429249 | 38.11984138 |
| GPR183 | G protein-coupled receptor 183 | 0.00659249 | 1.512352335 | 61.14274019 | 60.8788882 | 82.42194486 | 103.2939096 |
| LOC727808 | hypothetical protein LOC727808 | 0.00490511 | 1.512194321 | 9077.899395 | 10414.87097 | 14703.72029 | 14703.72029 |
| GNL3L | guanine nucleotide binding protein-like 3 (nucleolar)-like | 0.00384724 | 1.512053138 | 105.583533 | 107.6818767 | 176.0170984 | 147.6787664 |
| SYAP1 | synapse associated protein 1 | 0.00703639 | 1.511990496 | 91.90388332 | 89.70484076 | 130.042422 | 144.9315094 |
| RBMS1 | RNA binding motif. single stranded interacting protein 1 | 0.00692293 | 1.51119959 | 81.00033519 | 75.50386955 | 106.4091412 | 131.2564764 |
| DAAM1 | dishevelled associated activator of morphogenesis 1 | 0.00343362 | 1.510507034 | 183.3109624 | 167.1201926 | 248.7196256 | 281.0300843 |
| CLEC16A | C-type lectin domain family 16. member A | 0.00196653 | 1.510476992 | 534.7935561 | 531.9925402 | 778.6082548 | 833.6829759 |
| C7orf68 | chromosome 7 open reading frame 68 | 0.00547188 | 1.50976699 | 87.86836994 | 107.9886153 | 143.2081002 | 151.0298565 |
| FSD1 | fibronectin type III and SPRY domain containing 1 | 0.00688171 | 1.50914668 | 140.512329 | 121.2670833 | 191.7231083 | 202.416452 |
| RBM38 | RNA binding motif protein 38 | 0.00380264 | 1.508815099 | 435.7561628 | 479.8910157 | 735.4067477 | 647.3372375 |
| LOC100128510 | hypothetical protein LOC100128510 | 0.00742142 | 1.508776575 | 73.42674399 | 77.49848568 | 107.2307123 | 120.8031229 |
| EXT2 | exostosin 2 | 0.0153949 | 1.508087626 | 42.58840254 | 41.39789694 | 68.99971195 | 58.11329556 |
| SPATS2 | spermatogenesis associated. serine-rich 2 | 0.00863967 | 1.507961952 | 92.93234326 | 98.8145545 | 132.3595988 | 157.7658967 |
| SIDT2 | SID1 transmembrane family. member 2 | 0.00383475 | 1.507803695 | 818.5431477 | 918.2882925 | 1226.242087 | 1393.586764 |
| CES2 | carboxylesterase 2 (intestine. liver) | 0.01044852 | 1.507387358 | 25.52278845 | 21.18241622 | 33.52443535 | 36.64307264 |
| RALGAPA1 | Ral GTPase activating protein. alpha subunit 1 (catalytic) | 0.07356902 | 1.50635805 | 57.71210806 | 31.03868795 | 62.11741217 | 65.43549077 |
| GDF11 | growth differentiation factor 11 | 0.03731096 | 1.506343367 | 62.79522603 | 65.10801274 | 95.98135443 | 96.65451651 |
| LOC100131850 | hypothetical LOC100131850 | 0.32201906 | 1.505786784 | 51.45882553 | 31.93564133 | 50.58043 | 73.66818282 |
| TP53I13 | tumor protein p53 inducible protein 13 | 0.0029944 | 1.5055291 | 365.3784847 | 408.7622476 | 591.3020692 | 572.5094488 |
| CNNM2 | cyclin M2 | 0.05169326 | 1.505331093 | 33.35062691 | 21.98931574 | 42.69601702 | 38.92175536 |
| EYS | eyes shut homolog (Drosophila) | 0.10674897 | 1.504686711 | 32.71799867 | 20.076854 | 40.28656282 | 36.91597527 |
| SNORA21 | small nucleolar RNA. H/ACA box 21 | 0.02551328 | 1.504653165 | 30.0830726 | 22.53360314 | 37.92858856 | 40.46308185 |
| KCNMB2 | potassium large conductance calcium-activated channel. subfamily M. beta member 2 | 0.0298366 | 1.503992697 | 23.39438858 | 22.9680625 | 37.38801511 | 32.50836337 |
| BIN1 | bridging integrator 1 | 0.03721939 | 1.503169048 | 114.0541218 | 115.9727168 | 212.4705145 | 140.6642696 |
| DIS3L | DIS3 mitotic control homolog (S. cerevisiae)-like | 0.00409141 | 1.502228382 | 456.3786747 | 469.1795465 | 651.3841452 | 741.8210561 |
| SERINC2 | serine incorporator 2 | 0.00801486 | 1.502213934 | 34.10719118 | 34.33013234 | 53.38659233 | 49.49402853 |
| HOXA3 | homeobox A3 | 0.00990293 | 1.50217602 | 31.69515738 | 25.77455231 | 43.15358179 | 42.71779669 |
| TPRG1L | tumor protein p63 regulated 1-like | 0.00287413 | 1.501830515 | 516.9668455 | 530.2943299 | 748.0915101 | 826.5455608 |
| PRO0628 | hypothetical LOC29053 | 0.01126831 | 1.501585811 | 20.2956053 | 21.60391222 | 29.65611605 | 33.33653455 |
| ABHD5 | abhydrolase domain containing 5 | 0.00590664 | 1.501397805 | 59.68171502 | 49.75414855 | 77.78711693 | 86.05071253 |
| ASIP | agouti signaling protein | 0.01031394 | 1.501356821 | 33.00457591 | 33.99593418 | 45.634268 | 55.42145086 |
| DEGS1 | degenerative spermatocyte homolog 1. lipid desaturase (Drosophila) | 0.042087 | 1.501226465 | 158.3586815 | 145.7066261 | 179.4962197 | 289.7065383 |
| LGMN | legumain | 0.01712479 | 1.501177548 | 286.4048042 | 366.7761331 | 490.2671928 | 482.8504655 |
| TAGAP | T-cell activation RhoGTPase activating protein | 0.05677771 | 1.500890252 | 135.5511348 | 208.2842251 | 211.8953314 | 300.1483929 |
| AMY2A | amylase. alpha 2A (pancreatic) | 0.01620293 | 1.500266529 | 81.06140021 | 60.29617038 | 96.28270295 | 114.2595207 |
| PRPF6 | PRP6 pre-mRNA processing factor 6 homolog (S. cerevisiae) | 0.03097358 | -1.500201004 | 49.22073766 | 76.60865172 | 40.46308185 | 41.40645745 |
| TCF12 | transcription factor 12 | 0.04161764 | -1.500210889 | 461.1195066 | 376.6657169 | 245.8053178 | 313.9595419 |
| XBP1 | X-box binding protein 1 | 0.00306817 | -1.500474432 | 3754.91689 | 3758.006932 | 2652.741151 | 2362.685043 |
| SCAMP1 | secretory carrier membrane protein 1 | 0.00442984 | -1.500629805 | 54.79153269 | 51.76571277 | 32.90613004 | 38.27645442 |
| EIF4H | eukaryotic translation initiation factor 4H | 0.00212081 | -1.501559655 | 3003.146386 | 2965.514448 | 1980.457432 | 1994.462395 |
| HMBS | hydroxymethylbilane synthase | 0.00849923 | -1.502653973 | 230.7827724 | 264.522208 | 180.0571637 | 150.1540852 |
| LRDD | leucine-rich repeats and death domain containing | 0.05919752 | -1.503007174 | 72.60890557 | 56.25952957 | 35.62907538 | 50.75272062 |
| CAPN10 | calpain 10 | 0.00840361 | -1.503188174 | 25.46646473 | 29.70804854 | 17.03085787 | 19.65982103 |
| DHODH | dihydroorotate dehydrogenase | 0.00370319 | -1.503701849 | 34.75769067 | 35.95306446 | 25.40565646 | 21.75369016 |
| C6orf89 | chromosome 6 open reading frame 89 | 0.05364779 | -1.50386746 | 46.73554692 | 38.6654265 | 22.73905263 | 35.13812981 |
| ATP5S | ATP synthase. H+ transporting. mitochondrial Fo complex. subunit s (factor B) | 0.00650151 | -1.504007015 | 82.06077805 | 71.593461 | 47.74633426 | 54.39628373 |
| C5orf44 | chromosome 5 open reading frame 44 | 0.00942565 | -1.504243757 | 161.3242442 | 149.9862109 | 90.48651344 | 118.1763801 |
| IFNAR1 | interferon (alpha. beta and omega) receptor 1 | 0.00585101 | -1.504274369 | 257.7158719 | 295.9327486 | 177.7516918 | 189.6122273 |
| UBXN8 | UBX domain protein 8 | 0.03509384 | -1.504315707 | 33.92305107 | 22.70402466 | 16.78965502 | 20.27113205 |
| FTH1 | ferritin. heavy polypeptide 1 | 0.00954998 | -1.504649741 | 224.2814558 | 197.4669062 | 137.8212996 | 141.9386002 |
| GALNT2 | UDP-N-acetyl-alpha-D-galactosamine:polypeptide N-acetylgalactosaminyltransferase 2 (GalNAc-T2) | 0.07813055 | -1.505087495 | 70.49419178 | 86.45668786 | 45.43476219 | 59.21615103 |
| PPP6R3 | protein phosphatase 6. regulatory subunit 3 | 0.00311046 | -1.505194155 | 392.3694844 | 423.4671952 | 263.2775009 | 278.5583639 |
| FAM76B | family with sequence similarity 76. member B | 0.03522598 | -1.505549565 | 267.0892104 | 233.3986359 | 132.4015459 | 207.7167347 |
| DIAPH3 | diaphanous homolog 3 (Drosophila) | 0.00509558 | -1.505983843 | 90.48651344 | 82.08885894 | 62.47176787 | 52.4256293 |
| PPIG | peptidylprolyl isomerase G (cyclophilin G) | 0.0091638 | -1.506258665 | 304.7799633 | 283.0312786 | 207.1480786 | 183.5442653 |
| TMEM8A | transmembrane protein 8A | 0.01045641 | -1.506408626 | 89.42791 | 71.3092453 | 50.75272062 | 55.36993401 |
| USP21 | ubiquitin specific peptidase 21 | 0.01373974 | -1.50724804 | 126.0359929 | 126.7524391 | 94.21546621 | 74.63787309 |
| BET1L | blocked early in transport 1 homolog (S. cerevisiae)-like | 0.00483815 | -1.507253444 | 83.3824413 | 87.45188908 | 55.11674653 | 58.23547982 |
| GNAI3 | guanine nucleotide binding protein (G protein). alpha inhibiting activity polypeptide 3 | 0.00458204 | -1.507574884 | 50.62707359 | 43.08748016 | 30.78404078 | 31.17816193 |
| GART | phosphoribosylglycinamide formyltransferase. phosphoribosylglycinamide synthetase. phosphoribosylaminoimidazole synthetase | 0.00991183 | -1.507687875 | 627.2732123 | 625.3424249 | 361.0667742 | 477.929925 |
| MCM2 | minichromosome maintenance complex component 2 | 0.0043382 | -1.507697083 | 325.7719086 | 309.8473212 | 214.065326 | 207.4373055 |
| DPM1 | dolichyl-phosphate mannosyltransferase polypeptide 1. catalytic subunit | 0.0040191 | -1.507723452 | 1089.362695 | 1199.917986 | 807.1772231 | 712.380107 |
| XRCC5 | X-ray repair complementing defective repair in Chinese hamster cells 5 (double-strand-break rejoining) | 0.00413642 | -1.507896738 | 410.243207 | 481.2191949 | 304.9438109 | 284.7221891 |
| DUT | deoxyuridine triphosphatase | 0.00320334 | -1.507998934 | 635.0401034 | 591.3020692 | 386.3327763 | 427.412212 |
| PRIM1 | primase. DNA. polypeptide 1 (49kDa) | 0.00936842 | -1.508047646 | 455.5450132 | 348.3395375 | 252.5673364 | 276.265566 |
| ICT1 | immature colon carcinoma transcript 1 | 0.00466296 | -1.508200199 | 254.1755469 | 306.0620726 | 183.414373 | 186.4626829 |
| RPL41 | ribosomal protein L41 | 0.0394597 | -1.508511766 | 57.35859508 | 51.95691009 | 42.4180872 | 30.87403105 |
| NUP50 | nucleoporin 50kDa | 0.02578979 | -1.508535751 | 32.90271095 | 25.64438889 | 18.83498302 | 19.68555164 |
| NIPSNAP3A | nipsnap homolog 3A (C. elegans) | 0.01511624 | -1.508621436 | 119.2287096 | 107.9963171 | 88.51549934 | 63.91604836 |
| CSNK1G3 | casein kinase 1. gamma 3 | 0.02934733 | -1.508805895 | 128.9760151 | 94.54343738 | 63.14343476 | 84.82920481 |
| ACOT2 | acyl-CoA thioesterase 2 | 0.00420623 | -1.508852109 | 107.9354079 | 92.68422077 | 69.38586824 | 63.32939868 |
| LOC100289596 | hypothetical protein LOC100289596 | 0.05032811 | -1.509085674 | 24.13737546 | 32.32742061 | 22.95770478 | 14.92467354 |
| TSEN15 | tRNA splicing endonuclease 15 homolog (S. cerevisiae) | 0.00639394 | -1.509117859 | 721.1434589 | 687.3230078 | 454.8789081 | 478.4542685 |
| ITPRIP | inositol 1.4.5-triphosphate receptor interacting protein | 0.00380771 | -1.509316363 | 336.342228 | 385.9301298 | 245.9326324 | 231.6932402 |
| LMO4 | LIM domain only 4 | 0.00338945 | -1.509544376 | 1080.248319 | 1206.00194 | 716.8975319 | 797.4854195 |
| MOXD1 | monooxygenase. DBH-like 1 | 0.00182287 | -1.509576254 | 88.28709754 | 92.08995943 | 59.66974835 | 59.79229228 |
| KLHL12 | kelch-like 12 (Drosophila) | 0.00292827 | -1.509851916 | 155.547726 | 176.7051007 | 108.3237843 | 111.3064213 |
| DET1 | de-etiolated homolog 1 (Arabidopsis) | 0.00531986 | -1.510172221 | 140.6140836 | 147.1045683 | 105.0861931 | 86.30905602 |
| SERF1A | small EDRK-rich factor 1A (telomeric) | 0.10342565 | -1.510280266 | 72.59422697 | 78.61782299 | 39.60499809 | 63.17685555 |
| CALCOCO2 | calcium binding and coiled-coil domain 2 | 0.00319018 | -1.510319931 | 121.9826781 | 126.7229606 | 76.58440679 | 88.4861328 |
| ACOX1 | acyl-CoA oxidase 1. palmitoyl | 0.00785419 | -1.510596407 | 51.1452269 | 41.0588777 | 28.21457693 | 32.61682974 |
| MZT1 | mitotic spindle organizing protein 1 | 0.00392234 | -1.511064487 | 1057.026379 | 895.9007692 | 665.8295683 | 622.8976034 |
| ISG15 | ISG15 ubiquitin-like modifier | 0.05137511 | -1.511107032 | 303.7872118 | 460.4120975 | 246.5728952 | 248.4164932 |
| BRD7P3 | bromodomain containing 7 pseudogene 3 | 0.01583195 | -1.511107198 | 86.56061292 | 101.066587 | 66.16209232 | 57.90661031 |
| FBXL17 | F-box and leucine-rich repeat protein 17 | 0.03112141 | -1.511255419 | 23.27361803 | 35.97046832 | 18.30412488 | 20.02558823 |
| MCM6 | minichromosome maintenance complex component 6 | 0.00334285 | -1.511866895 | 1529.571108 | 1331.556574 | 925.0119693 | 963.2850662 |
| PRMT1 | protein arginine methyltransferase 1 | 0.0022009 | -1.5123906 | 47.41297336 | 44.44684873 | 30.49003832 | 30.21706353 |
| FSHB | follicle stimulating hormone. beta polypeptide | 0.00587563 | -1.512484785 | 29.43071847 | 28.53394549 | 18.03344802 | 20.35644285 |
| LOC732360 | similar to G/T mismatch-specific thymine DNA glycosylase | 0.00533394 | -1.512548159 | 37.71632773 | 36.94137594 | 22.31070131 | 27.29674253 |
| LOC727803 | similar to RAN binding protein 1 | 0.00391273 | -1.512756681 | 931.5039575 | 1042.058171 | 697.8818077 | 607.7943114 |
| LOC644063 | heterogeneous nuclear ribonucleoprotein K pseudogene | 0.00885582 | -1.512785546 | 1299.986675 | 1149.25755 | 712.380107 | 916.4098532 |
| WDR1 | WD repeat domain 1 | 0.00220495 | -1.512929419 | 1378.744959 | 1302.261284 | 852.0672658 | 920.5997446 |
| REST | RE1-silencing transcription factor | 0.04571532 | -1.5130064 | 27.97534214 | 31.29203571 | 24.01908013 | 15.92103595 |
| LEF1 | lymphoid enhancer-binding factor 1 | 0.00259018 | -1.513107126 | 1048.961596 | 973.7490162 | 666.172546 | 669.7003661 |
| MED22 | mediator complex subunit 22 | 0.00736914 | -1.513230882 | 57.73878398 | 45.06228826 | 34.92422883 | 32.5344641 |
| TNKS2 | tankyrase. TRF1-interacting ankyrin-related ADP-ribose polymerase 2 | 0.06524963 | -1.513231781 | 20.93184271 | 29.73930818 | 19.94572571 | 13.62942625 |
| HNRNPA1 | heterogeneous nuclear ribonucleoprotein A1 | 0.00194329 | -1.514040532 | 141.7153065 | 136.0417412 | 92.19676955 | 91.22171538 |
| LDOC1L | leucine zipper. down-regulated in cancer 1-like | 0.0458808 | -1.51422145 | 47.62821014 | 47.22186513 | 40.74740377 | 24.07291413 |
| C1orf204 | chromosome 1 open reading frame 204 | 0.10098259 | -1.514279322 | 22.71064815 | 26.8408876 | 12.69621423 | 20.93824015 |
| SNORD31 | small nucleolar RNA. C/D box 31 | 0.00275251 | -1.514394114 | 173.0609963 | 165.3470121 | 106.8667847 | 116.7548639 |
| INPP5E | inositol polyphosphate-5-phosphatase. 72 kDa | 0.004623 | -1.51458754 | 179.2407742 | 173.0202057 | 116.0968753 | 116.4459382 |
| FANCI | Fanconi anemia. complementation group I | 0.0189176 | -1.514769384 | 260.398764 | 188.951957 | 161.1526937 | 133.0637339 |
| GDI1 | GDP dissociation inhibitor 1 | 0.00885736 | -1.515146934 | 174.7624344 | 175.2447716 | 120.1684342 | 111.0179619 |
| WAC | WW domain containing adaptor with coiled-coil | 0.00278501 | -1.515375683 | 751.2354395 | 744.51307 | 474.1569239 | 513.6717348 |
| RNASEH2A | ribonuclease H2. subunit A | 0.0047094 | -1.515425064 | 200.5094717 | 206.1152836 | 122.819417 | 146.5241215 |
| LIN52 | lin-52 homolog (C. elegans) | 0.03385537 | -1.516089689 | 72.63263295 | 63.08791005 | 39.48899323 | 50.48380475 |
| ERO1LB | ERO1-like beta (S. cerevisiae) | 0.0239653 | -1.517441791 | 33.37714968 | 29.20379564 | 18.22334436 | 23.22931395 |
| EIF2B2 | eukaryotic translation initiation factor 2B. subunit 2 beta. 39kDa | 0.00717413 | -1.517526578 | 214.9264583 | 263.0132393 | 154.4301341 | 158.9508416 |
| WRN | Werner syndrome. RecQ helicase-like | 0.00234532 | -1.517714877 | 126.7229606 | 126.3244498 | 78.76825712 | 88.2289493 |
| HMGB2 | high-mobility group box 2 | 0.00224944 | -1.517738585 | 1633.083498 | 1497.137357 | 1060.632271 | 1000.716937 |
| HERC3 | hect domain and RLD 3 | 0.00597376 | -1.51818884 | 73.3124202 | 89.35958895 | 53.17805661 | 53.44839492 |
| REEP4 | receptor accessory protein 4 | 0.00321807 | -1.518455597 | 52.78581134 | 53.33969646 | 35.79166429 | 34.11784993 |
| SAP18 | Sin3A-associated protein. 18kDa | 0.01088484 | -1.518614259 | 73.47555401 | 70.80771207 | 54.13763164 | 41.67054988 |
| HLA-G | major histocompatibility complex. class I. G | 0.04085565 | -1.51882272 | 71.50152551 | 65.28048856 | 38.60654957 | 52.41113261 |
| COG3 | component of oligomeric golgi complex 3 | 0.01200974 | -1.51907536 | 174.3082037 | 177.6509857 | 98.76111065 | 135.8754029 |
| ACTR1B | ARP1 actin-related protein 1 homolog B. centractin beta (yeast) | 0.00364064 | -1.520477457 | 260.7910515 | 232.1949896 | 154.2752841 | 169.7810455 |
| DKC1 | dyskeratosis congenita 1. dyskerin | 0.00163861 | -1.521006534 | 2228.332415 | 2200.028151 | 1431.545852 | 1480.26951 |
| FAM24B | family with sequence similarity 24. member B | 0.01645788 | -1.521276521 | 245.9326324 | 215.4593478 | 142.809337 | 160.3277033 |
| ITPRIP | inositol 1.4.5-triphosphate receptor interacting protein | 0.00259168 | -1.52166928 | 466.7765611 | 444.3572103 | 283.0665648 | 316.4551377 |
| ZNF93 | zinc finger protein 93 | 0.0034087 | -1.521857102 | 42.68382111 | 37.29592268 | 27.63740866 | 24.87021709 |
| TCF19 | transcription factor 19 | 0.0021091 | -1.522528611 | 39.92307039 | 36.5825138 | 25.79337782 | 24.42633643 |
| SERPINA11 | serpin peptidase inhibitor. clade A (alpha-1 antiproteinase. antitrypsin). member 11 | 0.07497707 | -1.522820787 | 21.15209264 | 37.0076118 | 17.41580902 | 19.38220367 |
| CBLB | Cas-Br-M (murine) ecotropic retroviral transforming sequence b | 0.00321641 | -1.523591103 | 572.5094488 | 592.2310237 | 381.0055035 | 383.359039 |
| SRP54 | signal recognition particle 54kDa | 0.00212721 | -1.523592178 | 573.809078 | 630.607964 | 392.8803569 | 396.7610961 |
| LOC729342 | nucleophosmin (nucleolar phosphoprotein B23. numatrin) pseudogene | 0.00515778 | -1.524321631 | 103.1637027 | 84.24451644 | 59.03510686 | 63.35851423 |
| CDCA5 | cell division cycle associated 5 | 0.00302007 | -1.524630578 | 1161.278429 | 1268.956246 | 849.5607842 | 746.2069638 |
| HYOU1 | hypoxia up-regulated 1 | 0.01531574 | -1.524655606 | 340.4965409 | 316.9303888 | 250.3730096 | 185.4153 |
| ORC5L | origin recognition complex. subunit 5-like (yeast) | 0.01329212 | -1.524963395 | 85.29923498 | 71.41988671 | 58.44985665 | 44.81898374 |
| CCL4L2 | chemokine (C-C motif) ligand 4-like 2 | 0.0069741 | -1.525372901 | 1998.659772 | 2234.123249 | 1340.56749 | 1431.545852 |
| HPS4 | Hermansky-Pudlak syndrome 4 | 0.01765389 | -1.525458292 | 114.9140202 | 140.0831613 | 89.53114115 | 77.26523983 |
| PPIL5 | peptidylprolyl isomerase (cyclophilin)-like 5 | 0.00176059 | -1.525734575 | 237.5626409 | 222.3786774 | 152.3967024 | 148.9147585 |
| FOXM1 | forkhead box M1 | 0.00673928 | -1.52593781 | 215.3142131 | 224.9765299 | 144.6983714 | 143.771433 |
| MAGED2 | melanoma antigen family D. 2 | 0.01675592 | -1.526035319 | 30.34011562 | 33.2766671 | 20.85724164 | 20.7860042 |
| C6orf114 | chromosome 6 open reading frame 114 | 0.10465227 | -1.526501733 | 31.93325144 | 60.61361698 | 33.2325556 | 24.99510684 |
| ECSIT | ECSIT homolog (Drosophila) | 0.0190207 | -1.526619361 | 75.36595072 | 59.52937579 | 39.00255439 | 49.35736065 |
| GABBR1 | gamma-aminobutyric acid (GABA) B receptor. 1 | 0.03073852 | -1.52661964 | 26.3815205 | 31.19834785 | 20.44802544 | 17.27103447 |
| HIST1H2BD | histone cluster 1. H2bd | 0.02907087 | -1.526894144 | 97.29052053 | 90.64854184 | 61.80350077 | 61.20690437 |
| SMARCD1 | SWI/SNF related. matrix associated. actin dependent regulator of chromatin. subfamily d. member 1 | 0.01011626 | -1.527306683 | 1203.880534 | 1097.832318 | 649.2756266 | 872.6453914 |
| SNRNP40 | small nuclear ribonucleoprotein 40kDa (U5) | 0.00169663 | -1.527658754 | 490.6106711 | 501.7357204 | 334.9505537 | 314.9043102 |
| RAD51 | RAD51 homolog (RecA homolog. E. coli) (S. cerevisiae) | 0.00301193 | -1.52784072 | 29.92454883 | 32.18462252 | 21.61303303 | 19.08992195 |
| ZNF589 | zinc finger protein 589 | 0.01212285 | -1.528082341 | 136.6358494 | 109.678531 | 81.81622779 | 78.44284197 |
| C16orf87 | chromosome 16 open reading frame 87 | 0.00210152 | -1.52823791 | 296.7743111 | 288.5625638 | 185.0802091 | 198.1182006 |
| GADD45A | growth arrest and DNA-damage-inducible. alpha | 0.00521948 | -1.528243547 | 534.4642596 | 508.2356688 | 336.2094567 | 345.9304115 |
| CDT1 | chromatin licensing and DNA replication factor 1 | 0.01081473 | -1.528542545 | 271.425027 | 275.2749439 | 209.355082 | 152.7488687 |
| SUPT7L | suppressor of Ty 7 (S. cerevisiae)-like | 0.02830117 | -1.528803172 | 44.98609347 | 30.37140875 | 21.61224208 | 27.04831681 |
| AKT1S1 | AKT1 substrate 1 (proline-rich) | 0.02094955 | -1.528812835 | 27.6579377 | 29.65611605 | 22.41604211 | 15.65548377 |
| TCF12 | transcription factor 12 | 0.00455573 | -1.529130149 | 415.8144525 | 402.5311833 | 254.4113618 | 281.367353 |
| EIF4G2 | eukaryotic translation initiation factor 4 gamma. 2 | 0.00155494 | -1.529346901 | 463.7059654 | 466.5307497 | 297.9230613 | 310.4608342 |
| SCMH1 | sex comb on midleg homolog 1 (Drosophila) | 0.00482922 | -1.529505285 | 270.4220088 | 304.9438109 | 183.6089839 | 191.9844114 |
| TAF9 | TAF9 RNA polymerase II. TATA box binding protein (TBP)-associated factor. 32kDa | 0.02418428 | -1.529518566 | 212.5055206 | 194.3401952 | 162.3406262 | 108.7417026 |
| GNL3 | guanine nucleotide binding protein-like 3 (nucleolar) | 0.00177329 | -1.529521579 | 656.7288381 | 711.8951404 | 444.8196519 | 449.2694963 |
| BMP3 | bone morphogenetic protein 3 | 0.00740721 | -1.529570037 | 151.7435487 | 149.1578215 | 101.1646422 | 95.62881702 |
| GTF2IP1 | general transcription factor IIi. pseudogene 1 | 0.03173099 | -1.529687865 | 162.8679573 | 202.416452 | 107.440252 | 131.1320336 |
| SMC6 | structural maintenance of chromosomes 6 | 0.01384869 | -1.529837589 | 98.86101318 | 82.35367997 | 67.80439741 | 51.30498345 |
| UBASH3B | ubiquitin associated and SH3 domain containing B | 0.00817506 | -1.529946341 | 44.85562766 | 42.70689962 | 25.49608984 | 32.09881523 |
| TMEM48 | transmembrane protein 48 | 0.01905554 | -1.530148139 | 163.7436676 | 193.2686042 | 97.1544628 | 139.1220959 |
| WAPAL | wings apart-like homolog (Drosophila) | 0.00339326 | -1.530473351 | 193.2686042 | 207.7167347 | 130.7419053 | 131.0890582 |
| CDC25C | cell division cycle 25 homolog C (S. pombe) | 0.0072353 | -1.530908654 | 106.3496416 | 139.144176 | 77.89823719 | 81.05407947 |
| TK1 | thymidine kinase 1. soluble | 0.00295713 | -1.531020932 | 245.0659113 | 276.3293979 | 161.1799169 | 179.2407742 |
| SLC29A2 | solute carrier family 29 (nucleoside transporters). member 2 | 0.00305057 | -1.531081693 | 29.82629146 | 29.00087503 | 20.26110811 | 18.21169791 |
| GCH1 | GTP cyclohydrolase 1 | 0.01969497 | -1.532334938 | 110.8015817 | 86.32897456 | 74.78471028 | 54.4731286 |
| TSC1 | tuberous sclerosis 1 | 0.00610601 | -1.532417789 | 685.651954 | 718.1201328 | 464.1714357 | 451.7186686 |
| GYPC | glycophorin C (Gerbich blood group) | 0.00155542 | -1.532978086 | 907.7411016 | 930.1261485 | 615.0265877 | 584.1684131 |
| LAT2 | linker for activation of T cells family. member 2 | 0.03370726 | -1.533027731 | 70.82911683 | 70.24826447 | 41.28138681 | 51.28532658 |
| KCNN3 | potassium intermediate/small conductance calcium-activated channel. subfamily N. member 3 | 0.02690844 | -1.533035973 | 84.76238213 | 97.1544628 | 49.71892523 | 70.47571399 |
| CCL3 | chemokine (C-C motif) ligand 3 | 0.00143435 | -1.533595585 | 3451.935117 | 3368.870461 | 2205.996115 | 2241.405811 |
| MRPL20 | mitochondrial ribosomal protein L20 | 0.0047781 | -1.533928929 | 620.9995974 | 628.8624044 | 451.058212 | 367.9627706 |
| RAD17 | RAD17 homolog (S. pombe) | 0.00267873 | -1.534073461 | 205.9380684 | 231.264427 | 143.2228416 | 141.2995798 |
| RAD17 | RAD17 homolog (S. pombe) | 0.00410145 | -1.534174861 | 161.6511509 | 155.7229725 | 93.4437467 | 114.4541431 |
| MGC72080 | MGC72080 pseudogene | 0.005904 | -1.534530461 | 203.9361306 | 200.7802652 | 148.8133167 | 116.8482936 |
| CCPG1 | cell cycle progression 1 | 0.00218982 | -1.534865263 | 650.3453375 | 596.2340215 | 409.0175995 | 402.4188119 |
| RPS24 | ribosomal protein S24 | 0.00498923 | -1.534938543 | 688.5287979 | 728.5440269 | 458.9926348 | 463.8635114 |
| PRKAR1A | protein kinase. cAMP-dependent. regulatory. type I. alpha (tissue specific extinguisher 1) | 0.00147224 | -1.535279019 | 1619.941686 | 1600.70979 | 1029.206385 | 1068.894594 |
| CCDC132 | coiled-coil domain containing 132 | 0.01439864 | -1.5354482 | 29.11080856 | 32.71296844 | 17.73580896 | 22.77471136 |
| P4HA2 | prolyl 4-hydroxylase. alpha polypeptide II | 0.15133528 | -1.535535475 | 50.44185377 | 51.77492917 | 51.7399646 | 21.40743905 |
| GOLT1B | golgi transport 1B | 0.00645879 | -1.535801496 | 163.9044942 | 144.3301288 | 109.2652808 | 91.79002805 |
| C19orf6 | chromosome 19 open reading frame 6 | 0.0071379 | -1.535970056 | 443.2699901 | 346.4250829 | 261.5756941 | 248.8368957 |
| CENPM | centromere protein M | 0.00390884 | -1.535980877 | 163.8263423 | 165.7264912 | 101.7936498 | 113.053332 |
| BNIP2 | BCL2/adenovirus E1B 19kDa interacting protein 2 | 0.00929642 | -1.536447751 | 757.4857437 | 631.011437 | 484.5809825 | 417.8397339 |
| MCRS1 | microspherule protein 1 | 0.05441321 | -1.536606859 | 52.50992392 | 74.25806042 | 32.42385727 | 50.93247451 |
| SDHAF1 | succinate dehydrogenase complex assembly factor 1 | 0.12720691 | -1.536683823 | 96.23435596 | 103.5237128 | 61.74018649 | 68.33339646 |
| FNDC3B | fibronectin type III domain containing 3B | 0.00438097 | -1.536915642 | 365.0422322 | 312.5089916 | 217.1022327 | 222.4545422 |
| UBXN8 | UBX domain protein 8 | 0.03125096 | -1.537189136 | 37.0953546 | 24.29009929 | 18.89234482 | 20.18405126 |
| HMGB1 | high-mobility group box 1 | 0.03939965 | -1.537293436 | 32.67857721 | 31.55982734 | 18.82839681 | 23.17772842 |
| SNRPA | small nuclear ribonucleoprotein polypeptide A | 0.00649408 | -1.537295028 | 302.2015712 | 279.3936898 | 204.6024415 | 174.6176159 |
| PNMA1 | paraneoplastic antigen MA1 | 0.15844249 | -1.537447615 | 61.02382195 | 38.12946615 | 29.73465083 | 33.10523169 |
| BCCIP | BRCA2 and CDKN1A interacting protein | 0.00465159 | -1.538368211 | 486.4350507 | 407.7731872 | 286.0083496 | 293.0516836 |
| C6orf162 | chromosome 6 open reading frame 162 | 0.01453506 | -1.538562702 | 49.54515537 | 44.55759096 | 27.18459877 | 34.30595942 |
| UFM1 | ubiquitin-fold modifier 1 | 0.00219983 | -1.538581983 | 1176.401864 | 1038.594898 | 730.9199172 | 706.1399904 |
| SECISBP2 | SECIS binding protein 2 | 0.00154703 | -1.538782007 | 468.1293139 | 447.3337736 | 298.9719092 | 295.8100617 |
| SEPHS1 | selenophosphate synthetase 1 | 0.02159891 | -1.538930493 | 368.1205718 | 318.3581488 | 234.2883801 | 211.2113727 |
| YRDC | yrdC domain containing (E. coli) | 0.00257919 | -1.539230277 | 1012.886305 | 898.4192679 | 591.1389756 | 649.7450313 |
| GMNN | geminin. DNA replication inhibitor | 0.0099242 | -1.539633666 | 284.8258789 | 223.7752366 | 151.4452624 | 177.5419483 |
| PDCL3 | phosducin-like 3 | 0.04318404 | -1.539726748 | 197.7594535 | 135.3317719 | 116.8292695 | 96.62690107 |
| DDX42 | DEAD (Asp-Glu-Ala-Asp) box polypeptide 42 | 0.00147409 | -1.540068147 | 903.4556111 | 873.9718797 | 563.6420679 | 590.6374799 |
| PSMC4 | proteasome (prosome. macropain) 26S subunit. ATPase. 4 | 0.00164669 | -1.540207678 | 438.3274972 | 444.2602929 | 298.3880302 | 275.1035842 |
| CENPK | centromere protein K | 0.00522506 | -1.540394967 | 269.5885311 | 244.6602162 | 164.302805 | 169.1825661 |
| ZNF705G | zinc finger protein 705G | 0.01019074 | -1.540669353 | 39.52667819 | 49.72504017 | 32.19502601 | 25.71921997 |
| VRK1 | vaccinia related kinase 1 | 0.01343528 | -1.540734785 | 326.035177 | 309.396719 | 228.7845491 | 185.736688 |
| GART | phosphoribosylglycinamide formyltransferase. phosphoribosylglycinamide synthetase. phosphoribosylaminoimidazole synthetase | 0.00141649 | -1.541035754 | 1707.500092 | 1730.026604 | 1123.899932 | 1106.777809 |
| ORC1L | origin recognition complex. subunit 1-like (S. cerevisiae) | 0.00406013 | -1.54109625 | 80.47154572 | 90.55012457 | 59.15570095 | 51.86510526 |
| FNDC3A | fibronectin type III domain containing 3A | 0.00494147 | -1.541227642 | 227.1671148 | 213.6465784 | 143.0102792 | 142.8698439 |
| RECQL4 | RecQ protein-like 4 | 0.031914 | -1.541672044 | 77.93235324 | 49.88243208 | 38.64062649 | 42.32892674 |
| CDKN2AIP | CDKN2A interacting protein | 0.00903095 | -1.541872005 | 228.7845491 | 271.9512672 | 143.0330235 | 182.9720747 |
| SEH1L | SEH1-like (S. cerevisiae) | 0.00182204 | -1.542482338 | 205.8297088 | 209.355082 | 140.8778113 | 128.5608016 |
| TMPO | thymopoietin | 0.00255344 | -1.54298281 | 188.607526 | 188.3609334 | 121.1907691 | 123.1283714 |
| KLRA1 | killer cell lectin-like receptor subfamily A. member 1. pseudogene | 0.05819254 | -1.543045553 | 29.94922927 | 23.40288569 | 15.63876151 | 18.82327601 |
| PRPH | peripherin | 0.00275187 | -1.543139663 | 148.3022867 | 151.0298565 | 89.573226 | 105.0079001 |
| NFKBIB | nuclear factor of kappa light polypeptide gene enhancer in B-cells inhibitor. beta | 0.01163978 | -1.543435521 | 40.87775331 | 50.8896144 | 30.79426753 | 28.35758994 |
| ZFAND3 | zinc finger. AN1-type domain 3 | 0.01760528 | -1.543548778 | 121.3675894 | 107.3233183 | 85.89392763 | 63.64930116 |
| PPP2R3B | protein phosphatase 2. regulatory subunit B''. beta | 0.06760283 | -1.543772318 | 63.73825891 | 57.79508357 | 54.00526191 | 28.62124472 |
| SNRNP27 | small nuclear ribonucleoprotein 27kDa (U4/U6.U5) | 0.00431907 | -1.543994095 | 362.2807175 | 325.2752458 | 221.8819625 | 222.78347 |
| MRPL21 | mitochondrial ribosomal protein L21 | 0.00680795 | -1.544387667 | 713.3913085 | 654.796856 | 418.365547 | 468.1293139 |
| PIGF | phosphatidylinositol glycan anchor biosynthesis. class F | 0.05008304 | -1.544540413 | 52.93725914 | 43.37732463 | 39.67356363 | 24.26187996 |
| LPP | LIM domain containing preferred translocation partner in lipoma | 0.00421094 | -1.544611262 | 1671.80723 | 1819.538209 | 1097.115919 | 1162.134697 |
| SPCS3 | signal peptidase complex subunit 3 homolog (S. cerevisiae) | 0.01405611 | -1.544723858 | 139.8202282 | 152.1662118 | 86.30905602 | 103.3070976 |
| CCNL2 | cyclin L2 | 0.00904177 | -1.54488258 | 121.5016599 | 116.0968753 | 81.62448777 | 72.40879514 |
| ABCE1 | ATP-binding cassette. sub-family E (OABP). member 1 | 0.00314429 | -1.544918448 | 333.7748386 | 298.5281076 | 219.1840586 | 190.4665607 |
| SLC38A2 | solute carrier family 38. member 2 | 0.00926438 | -1.54504934 | 906.1019162 | 665.9337533 | 511.2715901 | 494.3923135 |
| ELAVL1 | ELAV (embryonic lethal. abnormal vision. Drosophila)-like 1 (Hu antigen R) | 0.00183013 | -1.545667404 | 194.3401952 | 197.7594535 | 121.2990295 | 132.6204402 |
| HAUS8 | HAUS augmin-like complex. subunit 8 | 0.00442633 | -1.545772478 | 128.2299626 | 128.7353476 | 85.68766902 | 80.62650891 |
| ISL2 | ISL LIM homeobox 2 | 0.04210431 | -1.546027952 | 46.81003896 | 64.70692033 | 42.55961451 | 29.77538352 |
| ZDHHC13 | zinc finger. DHHC-type containing 13 | 0.0047845 | -1.5461527 | 33.44198944 | 28.98060487 | 18.74135684 | 21.63185458 |
| ADM2 | adrenomedullin 2 | 0.002637 | -1.546656695 | 239.4389487 | 267.1330802 | 160.8426724 | 166.2391603 |
| LOC100128760 | hypothetical LOC100128760 | 0.01671579 | -1.546662493 | 41.9885442 | 44.94101738 | 23.97457553 | 32.90271095 |
| TUBA1C | tubulin. alpha 1c | 0.00416639 | -1.547172451 | 4236.399336 | 4201.271171 | 2974.656857 | 2499.55837 |
| ME2 | malic enzyme 2. NAD(+)-dependent. mitochondrial | 0.00308193 | -1.547177171 | 1292.365174 | 1249.340779 | 808.6676908 | 834.095874 |
| DAD1L | defender against cell death 1-like | 0.00769594 | -1.547197752 | 38.30563534 | 47.49078889 | 29.00087503 | 26.20410315 |
| PAICS | phosphoribosylaminoimidazole carboxylase. phosphoribosylaminoimidazole succinocarboxamide synthetase | 0.00282125 | -1.548211523 | 2854.126762 | 3110.833208 | 1825.870362 | 2028.707591 |
| ULBP2 | UL16 binding protein 2 | 0.06900399 | -1.548297513 | 40.40456139 | 51.7399646 | 27.37977203 | 31.85061803 |
| CDK4 | cyclin-dependent kinase 4 | 0.00375708 | -1.548320326 | 1819.538209 | 1527.115503 | 1020.569909 | 1135.713208 |
| ACSF3 | acyl-CoA synthetase family member 3 | 0.0013545 | -1.548320357 | 88.2289493 | 90.39799026 | 58.58173391 | 56.79184959 |
| RPS6KA3 | ribosomal protein S6 kinase. 90kDa. polypeptide 3 | 0.00866215 | -1.548542218 | 110.8343973 | 109.1766668 | 61.6886065 | 81.8000059 |
| TSTD2 | thiosulfate sulfurtransferase (rhodanese)-like domain containing 2 | 0.01946301 | -1.54892055 | 45.35752069 | 48.35372279 | 24.60513909 | 37.15310506 |
| CABLES1 | Cdk5 and Abl enzyme substrate 1 | 0.0023074 | -1.549390896 | 71.44046841 | 74.15630803 | 43.72267814 | 50.47352515 |
| LARP1B | La ribonucleoprotein domain family. member 1B | 0.07575461 | -1.549794608 | 28.03805884 | 53.17805661 | 26.04051019 | 23.83870312 |
| PYHIN1 | pyrin and HIN domain family. member 1 | 0.01757769 | -1.549837515 | 232.5210641 | 262.6738614 | 136.2233382 | 186.6617371 |
| ELP4 | elongation protein 4 homolog (S. cerevisiae) | 0.01391679 | -1.54988772 | 99.76718146 | 93.87930812 | 53.03113521 | 73.52350197 |
| PBX3 | pre-B-cell leukemia homeobox 3 | 0.00133593 | -1.550021482 | 471.629128 | 477.8441902 | 313.7481041 | 298.9719092 |
| DOCK3 | dedicator of cytokinesis 3 | 0.05946125 | -1.550033497 | 31.1758999 | 19.57477226 | 19.58012908 | 12.97233032 |
| VPS52 | vacuolar protein sorting 52 homolog (S. cerevisiae) | 0.00151947 | -1.550584404 | 169.9331743 | 180.6221762 | 109.7666579 | 116.3022124 |
| ATF4 | activating transcription factor 4 (tax-responsive enhancer element B67) | 0.02172366 | -1.550615277 | 804.753671 | 980.2043234 | 684.5144714 | 479.2794735 |
| UQCR10 | ubiquinol-cytochrome c reductase. complex III subunit X | 0.02716347 | -1.551493173 | 102.7393958 | 90.89585642 | 54.12954714 | 71.67157723 |
| PPP1R15B | protein phosphatase 1. regulatory (inhibitor) subunit 15B | 0.00272734 | -1.552012069 | 30.291601 | 29.92272249 | 18.01274512 | 20.89071193 |
| ATAD2 | ATPase family. AAA domain containing 2 | 0.0031585 | -1.552363171 | 213.9093873 | 200.6843715 | 122.0919352 | 145.9048597 |
| ZNF681 | zinc finger protein 681 | 0.00853996 | -1.552416323 | 61.94652002 | 53.54600166 | 37.39298259 | 36.80759596 |
| CTDSP1 | CTD (carboxy-terminal domain. RNA polymerase II. polypeptide A) small phosphatase 1 | 0.00240247 | -1.552418009 | 563.2521086 | 588.4684254 | 372.9922951 | 368.730266 |
| GSDMA | gasdermin A | 0.00274349 | -1.552694283 | 55.33397388 | 49.54515537 | 34.50269057 | 32.95855664 |
| GOLPH3L | golgi phosphoprotein 3-like | 0.01328877 | -1.552792626 | 232.6703244 | 210.9613423 | 151.582743 | 134.2972728 |
| BGLAP | bone gamma-carboxyglutamate (gla) protein | 0.00885769 | -1.553468739 | 29.25798811 | 39.0893503 | 21.02997665 | 22.53507895 |
| POLE | polymerase (DNA directed). epsilon | 0.01156957 | -1.553532322 | 138.1682315 | 105.1293155 | 69.68921896 | 86.36262846 |
| DERL1 | Der1-like domain family. member 1 | 0.00328915 | -1.553611087 | 745.6495963 | 673.0715967 | 455.3818613 | 456.5997815 |
| YME1L1 | YME1-like 1 (S. cerevisiae) | 0.00913508 | -1.553846364 | 410.1827221 | 381.0055035 | 222.9174969 | 290.3676984 |
| UQCC | ubiquinol-cytochrome c reductase complex chaperone | 0.01033994 | -1.554117345 | 53.39563805 | 65.41605373 | 37.80792752 | 38.25072181 |
| TMEM168 | transmembrane protein 168 | 0.00416617 | -1.554270008 | 123.0019661 | 119.0872568 | 86.97528202 | 69.71537457 |
| USP48 | ubiquitin specific peptidase 48 | 0.00500478 | -1.554471513 | 48.90076284 | 41.86441383 | 28.77556823 | 29.44226417 |
| LOC441228 | exportin. tRNA (nuclear export receptor for tRNAs) pseudogene | 0.00217322 | -1.555010445 | 1327.365984 | 1335.72443 | 836.3966914 | 876.6548224 |
| NASP | nuclear autoantigenic sperm protein (histone-binding) | 0.00167114 | -1.555285475 | 161.7712738 | 178.9998528 | 111.2306324 | 107.6240771 |
| TRIM11 | tripartite motif-containing 11 | 0.00210921 | -1.555452916 | 285.2293647 | 284.1242418 | 181.6735126 | 184.3729362 |
| IQCB1 | IQ motif containing B1 | 0.00192575 | -1.555471083 | 741.2280667 | 792.6291154 | 472.4450726 | 513.980532 |
| PYROXD1 | pyridine nucleotide-disulphide oxidoreductase domain 1 | 0.0020798 | -1.555791451 | 39.92934138 | 43.87189727 | 28.2400089 | 25.62777911 |
| CDCA4 | cell division cycle associated 4 | 0.00140541 | -1.555839343 | 383.359039 | 389.7694125 | 241.1595292 | 255.9643818 |
| UTP18 | UTP18. small subunit (SSU) processome component. homolog (yeast) | 0.00779248 | -1.556641982 | 217.4473788 | 241.7949184 | 135.7394137 | 159.8519355 |
| METTL9 | methyltransferase like 9 | 0.02520652 | -1.556730342 | 33.05220027 | 35.46065243 | 22.67956368 | 21.32483216 |
| NET1 | neuroepithelial cell transforming 1 | 0.10498429 | -1.557387797 | 38.02774281 | 25.66959402 | 26.10643715 | 15.41623802 |
| CDCA2 | cell division cycle associated 2 | 0.03094655 | -1.557653504 | 47.93511534 | 55.27603178 | 25.99526029 | 42.01020024 |
| ZBTB8OS | zinc finger and BTB domain containing 8 opposite strand | 0.00122836 | -1.558212203 | 443.7341931 | 435.3253095 | 276.3293979 | 287.9098477 |
| CBX5 | chromobox homolog 5 | 0.00156855 | -1.558225841 | 251.3571245 | 260.2560994 | 156.9731003 | 171.6350875 |
| TSGA14 | testis specific. 14 | 0.00132394 | -1.558262775 | 99.22016757 | 105.8434911 | 66.41135287 | 65.1238988 |
| ANKRD32 | ankyrin repeat domain 32 | 0.02752883 | -1.558754386 | 86.59764211 | 54.51371152 | 42.01658381 | 46.24190348 |
| DERL3 | Der1-like domain family. member 3 | 0.03594571 | -1.559118198 | 40.59610038 | 24.08832492 | 20.93396623 | 19.21681094 |
| LYPLA2P1 | lysophospholipase II pseudogene 1 | 0.08082331 | -1.559272131 | 52.54915018 | 71.6558402 | 50.37091214 | 30.74635525 |
| RANBP1 | RAN binding protein 1 | 0.00400833 | -1.560226039 | 1042.708975 | 941.3392302 | 647.763981 | 622.4685748 |
| TPM2 | tropomyosin 2 (beta) | 0.00570348 | -1.560353691 | 38.81009725 | 39.72287608 | 22.28481874 | 28.41385796 |
| FAM54A | family with sequence similarity 54. member A | 0.00207612 | -1.561664154 | 127.1767727 | 146.3576443 | 85.99497175 | 88.75132981 |
| FBXO7 | F-box protein 7 | 0.01252601 | -1.561720419 | 147.407146 | 128.9760151 | 92.04055167 | 84.69186602 |
| SLAMF7 | SLAM family member 7 | 0.01856586 | -1.56198085 | 75.31839667 | 107.3587556 | 51.4007244 | 64.47893939 |
| NEDD1 | neural precursor cell expressed. developmentally down-regulated 1 | 0.00552927 | -1.562445967 | 72.85685139 | 64.27021558 | 41.00510481 | 46.77698377 |
| NSUN3 | NOP2/Sun domain family. member 3 | 0.0014647 | -1.563159916 | 74.73589829 | 72.66512776 | 45.60645487 | 48.7328899 |
| SESTD1 | SEC14 and spectrin domains 1 | 0.00445079 | -1.563500211 | 78.37565772 | 67.2438836 | 43.04126188 | 50.0902388 |
| SREBF1 | sterol regulatory element binding transcription factor 1 | 0.02720359 | -1.564805812 | 107.2059188 | 106.9081355 | 60.52780355 | 77.3310138 |
| MRPS22 | mitochondrial ribosomal protein S22 | 0.00149536 | -1.5648778 | 1141.44362 | 1082.769397 | 682.3343423 | 739.6603671 |
| RNF219 | ring finger protein 219 | 0.00306847 | -1.56547228 | 772.8263179 | 641.258846 | 458.5695038 | 440.9804529 |
| RFX3 | regulatory factor X. 3 (influences HLA class II expression) | 0.02819818 | -1.565993268 | 26.55851866 | 41.05406272 | 21.87586156 | 20.32427401 |
| HLTF | helicase-like transcription factor | 0.00457105 | -1.566580239 | 141.0372807 | 111.2488992 | 82.78549465 | 77.22716848 |
| HMGXB4 | HMG box domain containing 4 | 0.00151949 | -1.567155683 | 181.6326001 | 169.0572417 | 111.8287657 | 111.8020492 |
| ADH5 | alcohol dehydrogenase 5 (class III). chi polypeptide | 0.02218528 | -1.567454537 | 71.08531892 | 47.28425522 | 33.52737329 | 40.80440343 |
| LOC642590 | spermine synthase pseudogene | 0.00770614 | -1.567519062 | 549.8504438 | 525.1034929 | 370.2326666 | 317.3869677 |
| SLC30A6 | solute carrier family 30 (zinc transporter). member 6 | 0.02442707 | -1.567794335 | 31.71016197 | 32.7367229 | 19.06024201 | 22.15782206 |
| ZFX | zinc finger protein. X-linked | 0.01159481 | -1.568174666 | 77.00433662 | 59.46103361 | 39.30360204 | 47.37251229 |
| MASTL | microtubule associated serine/threonine kinase-like | 0.00449492 | -1.569685833 | 150.4072779 | 129.2468773 | 81.84216139 | 96.40206636 |
| SENP2 | SUMO1/sentrin/SMT3 specific peptidase 2 | 0.00404341 | -1.569693025 | 665.9337533 | 600.6294696 | 369.5761406 | 439.2424265 |
| LCLAT1 | lysocardiolipin acyltransferase 1 | 0.00465008 | -1.569729207 | 94.45354871 | 97.19566323 | 53.86143721 | 69.17318162 |
| KLHL28 | kelch-like 28 (Drosophila) | 0.0024035 | -1.569891813 | 72.09083227 | 77.37836495 | 44.82407283 | 50.49505199 |
| HNRPDL | heterogeneous nuclear ribonucleoprotein D-like | 0.00139764 | -1.569939028 | 1674.518831 | 1736.387117 | 1131.109618 | 1042.956809 |
| RRN3P2 | RNA polymerase I transcription factor homolog (S. cerevisiae) pseudogene 2 | 0.01077238 | -1.569982077 | 67.45377362 | 58.40789748 | 33.99593418 | 47.01770809 |
| CDC5L | CDC5 cell division cycle 5-like (S. pombe) | 0.01575902 | -1.570512944 | 161.9266822 | 122.1483061 | 78.22913296 | 102.5071854 |
| GCH1 | GTP cyclohydrolase 1 | 0.00510333 | -1.571130786 | 37.48426923 | 29.72395745 | 22.62044756 | 19.9539927 |
| XPO7 | exportin 7 | 0.00222201 | -1.571261125 | 142.2731226 | 163.2966362 | 93.39968183 | 100.7529971 |
| FXR2 | fragile X mental retardation. autosomal homolog 2 | 0.00327368 | -1.571288168 | 70.11107703 | 69.20237834 | 49.23755456 | 39.91163359 |
| SELK | selenoprotein K | 0.00137884 | -1.571680925 | 362.7356741 | 336.5513674 | 216.5385383 | 228.2325924 |
| FEN1 | flap structure-specific endonuclease 1 | 0.00156677 | -1.572396084 | 1173.652797 | 1211.4708 | 762.8358419 | 753.8719619 |
| PINX1 | PIN2/TERF1 interacting. telomerase inhibitor 1 | 0.0043994 | -1.572657991 | 106.1092157 | 84.08306879 | 58.15165632 | 62.03417301 |
| STK40 | serine/threonine kinase 40 | 0.0019343 | -1.573101735 | 1485.230907 | 1589.477702 | 917.7456926 | 1039.471156 |
| PDZD7 | PDZ domain containing 7 | 0.00380543 | -1.573306477 | 26.42108951 | 30.31484439 | 18.2691807 | 17.71170349 |
| LOC100128086 | APAF1 interacting protein pseudogene | 0.00192276 | -1.573306963 | 580.085728 | 672.4654156 | 395.0124945 | 398.9554225 |
| ZNF322B | zinc finger protein 322B | 0.08971201 | -1.573437583 | 32.81996046 | 23.74443822 | 14.2931455 | 22.02282859 |
| BAX | BCL2-associated X protein | 0.02054936 | -1.574025598 | 50.54156043 | 59.94625023 | 35.45095888 | 34.49523439 |
| TUBG1 | tubulin. gamma 1 | 0.03562225 | -1.57495635 | 335.4423127 | 442.2589388 | 306.0620726 | 195.4105039 |
| VBP1 | von Hippel-Lindau binding protein 1 | 0.00406695 | -1.575109472 | 674.4911379 | 556.8033669 | 390.8430389 | 387.3060069 |
| SCML2 | sex comb on midleg-like 2 (Drosophila) | 0.00138381 | -1.575208044 | 85.86889732 | 77.58136524 | 51.86510526 | 51.76571277 |
| DNMT3B | DNA (cytosine-5-)-methyltransferase 3 beta | 0.02669092 | -1.575404861 | 41.17726463 | 49.60657713 | 34.90798492 | 23.57694949 |
| DAZAP1 | DAZ associated protein 1 | 0.0082453 | -1.575532159 | 838.1144134 | 836.3966914 | 585.1949906 | 482.5701688 |
| IWS1 | IWS1 homolog (S. cerevisiae) | 0.00235269 | -1.575794434 | 478.4542685 | 427.5750564 | 269.6909799 | 305.4831427 |
| ANAPC7 | anaphase promoting complex subunit 7 | 0.00296853 | -1.57591493 | 121.1416166 | 120.7747676 | 69.50257295 | 84.76238213 |
| STX5 | syntaxin 5 | 0.00203727 | -1.576124494 | 94.47847739 | 105.8618829 | 60.04997195 | 67.04689257 |
| NFE2L2 | nuclear factor (erythroid-derived 2)-like 2 | 0.00181626 | -1.576145877 | 366.6309345 | 341.7954303 | 214.1649305 | 235.5343108 |
| NDUFA4L2 | NADH dehydrogenase (ubiquinone) 1 alpha subcomplex. 4-like 2 | 0.0094315 | -1.576927789 | 48.28324044 | 49.34803588 | 27.40140002 | 34.96792929 |
| PLCXD1 | phosphatidylinositol-specific phospholipase C. X domain containing 1 | 0.0014366 | -1.577184334 | 401.300877 | 430.8862311 | 256.1049227 | 271.425027 |
| HNRPDL | heterogeneous nuclear ribonucleoprotein D-like | 0.00192466 | -1.577271525 | 140.9605906 | 126.5104917 | 83.90323035 | 85.43437494 |
| SON | SON DNA binding protein | 0.0086672 | -1.577731499 | 92.28959617 | 100.7615336 | 64.89654473 | 57.56516424 |
| MED1 | mediator complex subunit 1 | 0.0012666 | -1.578087682 | 93.67386521 | 86.05071253 | 57.73878398 | 56.05869952 |
| HMGXB4 | HMG box domain containing 4 | 0.00443166 | -1.578135263 | 68.65516215 | 57.55572154 | 38.7856727 | 40.90740435 |
| LOC643438 | chromosome 15 open reading frame 63 pseudogene | 0.00193023 | -1.578505621 | 301.9874201 | 290.4102967 | 193.3169389 | 182.0701237 |
| DOCK8 | dedicator of cytokinesis 8 | 0.0019065 | -1.578671744 | 305.7425424 | 303.6931228 | 207.0406869 | 179.9498373 |
| THSD1P1 | thrombospondin. type I. domain containing 1 pseudogene 1 | 0.0299367 | -1.579936135 | 36.67283061 | 60.63801636 | 29.99254611 | 29.70277458 |
| MTO1 | mitochondrial translation optimization 1 homolog (S. cerevisiae) | 0.00183045 | -1.580081392 | 669.3479565 | 599.7581314 | 381.7223349 | 421.2322346 |
| MTHFD1 | methylenetetrahydrofolate dehydrogenase (NADP+ dependent) 1. methenyltetrahydrofolate cyclohydrolase. formyltetrahydrofolate synthetase | 0.02675867 | -1.580816861 | 75.50386955 | 77.46837694 | 38.02774281 | 61.55027529 |
| ARL6IP6 | ADP-ribosylation-like factor 6 interacting protein 6 | 0.00886095 | -1.580952521 | 609.1231 | 476.2755583 | 305.2634153 | 380.2340019 |
| GMPPB | GDP-mannose pyrophosphorylase B | 0.01447052 | -1.581377944 | 90.16557475 | 106.7747078 | 73.88129611 | 52.10786592 |
| CSNK2A1P | casein kinase 2. alpha 1 polypeptide pseudogene | 0.00252237 | -1.581455105 | 140.7032717 | 144.720748 | 94.3591755 | 86.28536129 |
| C6orf125 | chromosome 6 open reading frame 125 | 0.00217923 | -1.582081761 | 510.9858473 | 563.7866181 | 328.2297455 | 350.6613102 |
| DEPDC1B | DEP domain containing 1B | 0.01295044 | -1.58215272 | 69.4147217 | 53.92542565 | 33.4700433 | 44.67783191 |
| IRF2BP2 | interferon regulatory factor 2 binding protein 2 | 0.00141533 | -1.582246637 | 277.0800696 | 258.8086357 | 175.4785175 | 163.2344168 |
| SLC38A1 | solute carrier family 38. member 1 | 0.00168821 | -1.582640837 | 119.0080157 | 128.8850546 | 82.86532784 | 73.89944652 |
| HNRNPK | heterogeneous nuclear ribonucleoprotein K | 0.04297513 | -1.582683356 | 372.6099795 | 397.2051607 | 260.834883 | 226.5247307 |
| SLC39A14 | solute carrier family 39 (zinc transporter). member 14 | 0.00254026 | -1.582795763 | 132.7434128 | 115.3658915 | 81.99431586 | 74.55158837 |
| THAP1 | THAP domain containing. apoptosis associated protein 1 | 0.00857518 | -1.582911839 | 32.95855664 | 30.2962628 | 23.49048911 | 16.96491662 |
| HNRNPK | heterogeneous nuclear ribonucleoprotein K | 0.00170884 | -1.583382015 | 2111.37904 | 1986.756033 | 1220.052674 | 1371.389279 |
| PKM2 | pyruvate kinase. muscle | 0.00249174 | -1.583714309 | 165.826073 | 172.4654361 | 103.9337124 | 109.7096339 |
| LOC100129958 | keratin 8 pseudogene | 0.04233732 | -1.583806464 | 36.87426233 | 50.60923806 | 24.62339754 | 30.21349325 |
| MCM7 | minichromosome maintenance complex component 7 | 0.00259938 | -1.584127611 | 3265.437067 | 3422.164156 | 2159.250898 | 2062.332662 |
| ASAP1 | ArfGAP with SH3 domain. ankyrin repeat and PH domain 1 | 0.00152106 | -1.58423033 | 126.8534392 | 114.0140757 | 73.92007827 | 77.95817283 |
| C16orf75 | chromosome 16 open reading frame 75 | 0.00129578 | -1.584900738 | 735.7653221 | 763.450415 | 455.8049369 | 490.6106711 |
| VNN2 | vanin 2 | 0.00220315 | -1.585625699 | 79.85459515 | 79.33888989 | 46.70511254 | 53.95357877 |
| NFKBIB | nuclear factor of kappa light polypeptide gene enhancer in B-cells inhibitor. beta | 0.01714902 | -1.585673496 | 89.06018212 | 85.29923498 | 67.14971786 | 44.99424706 |
| C3orf75 | chromosome 3 open reading frame 75 | 0.00288196 | -1.585771298 | 134.6932864 | 149.7353778 | 89.24934316 | 89.86368786 |
| ZNF273 | zinc finger protein 273 | 0.02117928 | -1.586036808 | 64.49881654 | 46.09805459 | 29.92637528 | 39.49605347 |
| RAE1 | RAE1 RNA export 1 homolog (S. pombe) | 0.01896734 | -1.586133252 | 102.1312643 | 127.3703959 | 61.38781695 | 84.2297822 |
| DCP1A | DCP1 decapping enzyme homolog A (S. cerevisiae) | 0.0048582 | -1.586598215 | 127.8855907 | 142.4659777 | 87.42680474 | 82.78549465 |
| CKS1B | CDC28 protein kinase regulatory subunit 1B | 0.00177314 | -1.587288746 | 3091.14403 | 3543.258039 | 2043.684844 | 2127.140731 |
| FAS | Fas (TNF receptor superfamily. member 6) | 0.04299103 | -1.587517225 | 35.9143985 | 33.10523169 | 25.71768879 | 18.3440996 |
| HMGA2 | high mobility group AT-hook 2 | 0.02470102 | -1.588452333 | 20.72822719 | 30.34011562 | 14.26659185 | 17.4707191 |
| ING3 | inhibitor of growth family. member 3 | 0.00400574 | -1.589252632 | 122.0919352 | 128.2299626 | 82.50418093 | 75.13021946 |
| MOBKL3 | MOB1. Mps One Binder kinase activator-like 3 (yeast) | 0.0016519 | -1.589351574 | 744.51307 | 682.3343423 | 428.1185019 | 469.7487213 |
| GMPS | guanine monphosphate synthetase | 0.00135669 | -1.590002692 | 848.8486782 | 810.9257594 | 515.4736528 | 528.2131166 |
| HAUS8 | HAUS augmin-like complex. subunit 8 | 0.00541755 | -1.590656654 | 193.7149261 | 148.2280627 | 112.117069 | 101.2205957 |
| FOLR2 | folate receptor 2 (fetal) | 0.0394895 | -1.590719251 | 48.96482715 | 84.14973406 | 39.88345065 | 40.82791237 |
| NUDT15 | nudix (nucleoside diphosphate linked moiety X)-type motif 15 | 0.02390433 | -1.591985709 | 81.64092478 | 73.37577627 | 55.47923841 | 42.60414005 |
| RETSAT | retinol saturase (all-trans-retinol 13.14-reductase) | 0.19775739 | -1.592050158 | 38.48730155 | 70.77098979 | 44.8447163 | 23.96337362 |
| TMEM33 | transmembrane protein 33 | 0.00730662 | -1.592150587 | 75.96959567 | 67.65087974 | 39.56157352 | 51.2473513 |
| SDHAP1 | succinate dehydrogenase complex. subunit A. flavoprotein pseudogene 1 | 0.00397708 | -1.593318832 | 73.48365269 | 70.58359044 | 43.16202191 | 47.33553856 |
| NACC2 | NACC family member 2. BEN and BTB (POZ) domain containing | 0.06183733 | -1.593669155 | 29.77720978 | 17.29869493 | 12.17231303 | 16.66202929 |
| NARS | asparaginyl-tRNA synthetase | 0.00168159 | -1.593742941 | 2721.354493 | 2543.624385 | 1755.357782 | 1552.515342 |
| DOHH | deoxyhypusine hydroxylase/monooxygenase | 0.04695076 | -1.594617813 | 54.47632612 | 48.37409126 | 31.60893191 | 32.78669096 |
| SPC25 | SPC25. NDC80 kinetochore complex component. homolog (S. cerevisiae) | 0.00704256 | -1.594665515 | 108.227675 | 115.1926079 | 65.41605373 | 74.94443546 |
| STRADA | STE20-related kinase adaptor alpha | 0.0040966 | -1.5948063 | 66.40325137 | 78.83630061 | 47.64580697 | 43.19913086 |
| CSGALNACT2 | chondroitin sulfate N-acetylgalactosaminyltransferase 2 | 0.00456242 | -1.594827914 | 54.82801937 | 62.5109969 | 38.31918448 | 35.16536001 |
| RALGPS2 | Ral GEF with PH domain and SH3 binding motif 2 | 0.02197682 | -1.595210438 | 92.16718913 | 92.80605936 | 59.55144359 | 56.44486205 |
| ZNF679 | zinc finger protein 679 | 0.01595648 | -1.595744942 | 101.7166114 | 71.24428724 | 58.04916018 | 49.02516179 |
| RPS15 | ribosomal protein S15 | 0.00248962 | -1.59577193 | 149.4528307 | 145.0572989 | 99.56976788 | 85.50166931 |
| FYN | FYN oncogene related to SRC. FGR. YES | 0.00898526 | -1.595776037 | 105.7614499 | 124.1736939 | 62.58206438 | 82.40683476 |
| SRSF10 | serine/arginine-rich splicing factor 10 | 0.00220071 | -1.596211312 | 163.2344168 | 181.1776189 | 116.293297 | 99.81155643 |
| PAICS | phosphoribosylaminoimidazole carboxylase. phosphoribosylaminoimidazole succinocarboxamide synthetase | 0.00106682 | -1.596485413 | 2831.142247 | 2848.673172 | 1768.222267 | 1789.524099 |
| CDK1 | cyclin-dependent kinase 1 | 0.01842394 | -1.597293482 | 218.4782168 | 149.0939481 | 108.5608262 | 117.6049439 |
| HUWE1 | HECT. UBA and WWE domain containing 1 | 0.00425207 | -1.59891732 | 118.9269111 | 129.7891599 | 86.23571713 | 70.01310958 |
| EPN2 | epsin 2 | 0.00610695 | -1.599160744 | 47.81623857 | 42.73015156 | 28.31252997 | 28.21934464 |
| YEATS4 | YEATS domain containing 4 | 0.00114497 | -1.599822965 | 532.8001487 | 515.1269277 | 329.892144 | 325.0594254 |
| KDM2B | lysine (K)-specific demethylase 2B | 0.0186006 | -1.599953687 | 29.50894636 | 43.01256281 | 22.24126728 | 22.29331594 |
| PGK1 | phosphoglycerate kinase 1 | 0.00517427 | -1.600170983 | 478.6489234 | 386.3327763 | 247.7918806 | 291.4464623 |
| APIP | APAF1 interacting protein | 0.01205455 | -1.601292847 | 189.5118191 | 197.8777151 | 132.1045381 | 110.7067044 |
| CKS1B | CDC28 protein kinase regulatory subunit 1B | 0.00103713 | -1.601296106 | 2306.808659 | 2231.370596 | 1375.05511 | 1459.889145 |
| ITFG2 | integrin alpha FG-GAP repeat containing 2 | 0.00126728 | -1.601722792 | 240.1199937 | 254.9625968 | 161.9266822 | 147.3708772 |
| IGLL1 | immunoglobulin lambda-like polypeptide 1 | 0.01940427 | -1.602075849 | 40.23434359 | 37.53994329 | 19.43959415 | 30.27172655 |
| CCPG1 | cell cycle progression 1 | 0.03236938 | -1.604010231 | 58.77586425 | 41.17726463 | 26.70424423 | 35.22588866 |
| GRB2 | growth factor receptor-bound protein 2 | 0.00951852 | -1.604213312 | 340.0193812 | 425.89008 | 260.7267935 | 215.8198078 |
| RPL32 | ribosomal protein L32 | 0.00730633 | -1.604537999 | 56.1471154 | 59.20832399 | 39.25650741 | 32.89262583 |
| RB1 | retinoblastoma 1 | 0.0066774 | -1.604578013 | 150.358427 | 134.5375197 | 77.27947886 | 101.6682462 |
| TXLNG | taxilin gamma | 0.00140997 | -1.605494511 | 55.71682029 | 55.03121462 | 32.36920931 | 36.74901316 |
| PANK1 | pantothenate kinase 1 | 0.00141206 | -1.605754499 | 36.36733962 | 36.98931215 | 24.33078417 | 21.44239965 |
| ARFGAP3 | ADP-ribosylation factor GTPase activating protein 3 | 0.00150623 | -1.605890038 | 670.4314682 | 680.7196495 | 393.994317 | 449.1600401 |
| KLHL38 | kelch-like 38 (Drosophila) | 0.04182936 | -1.606156648 | 36.28513786 | 26.21213185 | 14.94521762 | 24.66908714 |
| CBS | cystathionine-beta-synthase | 0.00137542 | -1.606179594 | 1136.598192 | 1272.789485 | 721.9092685 | 776.7704821 |
| HPS4 | Hermansky-Pudlak syndrome 4 | 0.01313975 | -1.606537943 | 42.63746727 | 49.50698991 | 25.67336477 | 31.85614028 |
| WDR33 | WD repeat domain 33 | 0.00791766 | -1.606792994 | 141.3941858 | 130.5695697 | 72.88991991 | 98.10388196 |
| FBXO38 | F-box protein 38 | 0.00531619 | -1.606836576 | 50.53040994 | 46.36414514 | 28.85926064 | 31.44173189 |
| DHX40 | DEAH (Asp-Glu-Ala-His) box polypeptide 40 | 0.00352467 | -1.607048612 | 88.43639296 | 102.5321757 | 56.46092923 | 62.18492919 |
| CAMP | cathelicidin antimicrobial peptide | 0.03293749 | -1.607172815 | 161.2664525 | 150.2971919 | 123.4445718 | 76.01472805 |
| DHX36 | DEAH (Asp-Glu-Ala-His) box polypeptide 36 | 0.00169182 | -1.607294528 | 287.0177108 | 283.3838852 | 191.899187 | 164.0664493 |
| PRPSAP2 | phosphoribosyl pyrophosphate synthetase-associated protein 2 | 0.005775 | -1.607665148 | 428.4543849 | 371.6403637 | 226.2630014 | 272.2843816 |
| OR2A1 | olfactory receptor. family 2. subfamily A. member 1 | 0.00342615 | -1.608381564 | 32.81063994 | 27.05650059 | 17.40089848 | 19.72134272 |
| ARF1 | ADP-ribosylation factor 1 | 0.00546093 | -1.608948853 | 277.3731053 | 235.8400844 | 142.6210792 | 177.1796236 |
| RNF146 | ring finger protein 146 | 0.0011472 | -1.6089904 | 142.2306861 | 129.0204615 | 85.91500766 | 82.50418093 |
| SEC63 | SEC63 homolog (S. cerevisiae) | 0.00121011 | -1.609045749 | 58.28978771 | 52.32423729 | 35.16536001 | 33.4999013 |
| METTL4 | methyltransferase like 4 | 0.00240219 | -1.609103174 | 117.124262 | 137.6376535 | 83.43041806 | 74.62630249 |
| SFRS12 | splicing factor. arginine/serine-rich 12 | 0.01037377 | -1.609170996 | 140.037192 | 107.4285181 | 68.93008252 | 84.28490234 |
| DUT | deoxyuridine triphosphatase | 0.00202024 | -1.609181815 | 490.2671928 | 449.2694963 | 282.3213283 | 301.2907817 |
| HAUS8 | HAUS augmin-like complex. subunit 8 | 0.01357211 | -1.609916249 | 115.8890495 | 111.2658456 | 82.74277953 | 60.1267321 |
| PRKDC | protein kinase. DNA-activated. catalytic polypeptide | 0.00576116 | -1.610159806 | 80.8710444 | 104.1200469 | 52.59495271 | 61.75119631 |
| SLC10A7 | solute carrier family 10 (sodium/bile acid cotransporter family). member 7 | 0.00210587 | -1.610287298 | 39.83348782 | 36.5575288 | 25.09020035 | 22.38279422 |
| LRMP | lymphoid-restricted membrane protein | 0.00120223 | -1.610303527 | 3123.891048 | 2831.142247 | 1907.550408 | 1787.994002 |
| MZT1 | mitotic spindle organizing protein 1 | 0.00393105 | -1.613252265 | 181.9199839 | 175.9362165 | 123.9456056 | 99.22016757 |
| TIPIN | TIMELESS interacting protein | 0.00673674 | -1.613317498 | 114.1067198 | 85.83646809 | 63.67320719 | 59.09988643 |
| LOC730183 | hypothetical protein LOC730183 | 0.0138975 | -1.614398525 | 36.59244283 | 38.6287329 | 25.7850265 | 21.03355973 |
| HCK | hemopoietic cell kinase | 0.00536208 | -1.614504778 | 48.01964816 | 61.80350077 | 31.29203571 | 36.38476406 |
| EIF5A | eukaryotic translation initiation factor 5A | 0.00115533 | -1.615155755 | 1607.005098 | 1677.189144 | 977.8451839 | 1056.575115 |
| TBC1D1 | TBC1 (tre-2/USP6. BUB2. cdc16) domain family. member 1 | 0.06097735 | -1.615223666 | 42.06156581 | 31.41821007 | 20.29751539 | 24.95506224 |
| U2AF2 | U2 small nuclear RNA auxiliary factor 2 | 0.00380879 | -1.615292274 | 58.18559902 | 46.44279023 | 32.30425475 | 32.06061547 |
| IDUA | iduronidase. alpha-L- | 0.00352214 | -1.615327035 | 36.8485887 | 35.33215999 | 25.2887238 | 19.7307326 |
| ATP2A2 | ATPase. Ca++ transporting. cardiac muscle. slow twitch 2 | 0.00142285 | -1.61575754 | 461.6379615 | 490.9277124 | 279.6858513 | 310.3817541 |
| TP53RK | TP53 regulating kinase | 0.00105019 | -1.616081134 | 313.461299 | 294.1917057 | 186.7902069 | 189.0313908 |
| PYHIN1 | pyrin and HIN domain family. member 1 | 0.00344056 | -1.616120332 | 337.9145383 | 405.4520505 | 219.080737 | 239.4389487 |
| IDH1 | isocitrate dehydrogenase 1 (NADP+). soluble | 0.00189372 | -1.616144375 | 208.5165747 | 207.6077199 | 121.0546534 | 136.9122419 |
| CACYBP | calcyclin binding protein | 0.03882471 | -1.616423752 | 37.18999376 | 40.44270695 | 22.75296904 | 25.29984143 |
| NEIL3 | nei endonuclease VIII-like 3 (E. coli) | 0.01232463 | -1.616590446 | 181.2964586 | 175.1810801 | 115.1013228 | 105.583533 |
| SNRNP40 | small nuclear ribonucleoprotein 40kDa (U5) | 0.00140705 | -1.616630634 | 737.4983755 | 732.6255052 | 427.5750564 | 483.5140876 |
| LEO1 | Leo1. Paf1/RNA polymerase II complex component. homolog (S. cerevisiae) | 0.00607108 | -1.616933708 | 368.4941005 | 432.0459453 | 222.5259738 | 273.6499248 |
| TGM2 | transglutaminase 2 (C polypeptide. protein-glutamine-gamma-glutamyltransferase) | 0.02021609 | -1.617663788 | 29.79681409 | 35.71842046 | 16.41431259 | 24.77780606 |
| CEP110 | centrosomal protein 110kDa | 0.03140242 | -1.617837306 | 249.2482581 | 278.3441997 | 154.3367159 | 171.7415433 |
| TMTC4 | transmembrane and tetratricopeptide repeat containing 4 | 0.01251994 | -1.617952477 | 29.06890756 | 33.36650214 | 23.39008892 | 15.84075937 |
| LMO2 | LIM domain only 2 (rhombotin-like 1) | 0.00305461 | -1.619755302 | 177.1446237 | 216.1158799 | 118.8319869 | 122.7955069 |
| CCR9 | chemokine (C-C motif) receptor 9 | 0.0011899 | -1.619983288 | 31.98132372 | 36.17797431 | 20.90565152 | 21.08899608 |
| CNOT3 | CCR4-NOT transcription complex. subunit 3 | 0.00632414 | -1.620135553 | 117.4786027 | 121.4564749 | 68.80343284 | 79.00719311 |
| ASB7 | ankyrin repeat and SOCS box-containing 7 | 0.00232793 | -1.620171638 | 65.26034763 | 55.81619147 | 37.04734658 | 37.45677858 |
| LIG3 | ligase III. DNA. ATP-dependent | 0.00117824 | -1.62051297 | 138.2819871 | 131.4796963 | 84.95517254 | 81.49471679 |
| GNAZ | guanine nucleotide binding protein (G protein). alpha z polypeptide | 0.00871353 | -1.62099182 | 54.38308766 | 41.38210151 | 29.57201244 | 28.96235525 |
| ZNF473 | zinc finger protein 473 | 0.00608699 | -1.621034963 | 62.58206438 | 61.51691039 | 32.57483223 | 44.97565004 |
| DCAF15 | DDB1 and CUL4 associated factor 15 | 0.01274851 | -1.621542459 | 38.87471916 | 53.62989102 | 29.02607051 | 27.31676606 |
| SORL1 | sortilin-related receptor. L(DLR class) A repeats-containing | 0.00664164 | -1.621926318 | 265.6585126 | 295.8100617 | 157.6297318 | 189.5118191 |
| APBB2 | amyloid beta (A4) precursor protein-binding. family B. member 2 | 0.00360958 | -1.62266852 | 33.81993808 | 29.51531004 | 19.0041513 | 19.94858322 |
| NFRKB | nuclear factor related to kappaB binding protein | 0.02429565 | -1.622721491 | 50.8425839 | 58.11329556 | 26.13100402 | 42.93970395 |
| OR4S1 | olfactory receptor. family 4. subfamily S. member 1 | 0.00278321 | -1.623990481 | 31.69190403 | 27.22399328 | 16.74962771 | 19.53116027 |
| YWHAZ | tyrosine 3-monooxygenase/tryptophan 5-monooxygenase activation protein. zeta polypeptide | 0.00083112 | -1.624201404 | 297.9230613 | 289.6106403 | 174.8975359 | 187.0058455 |
| WDR33 | WD repeat domain 33 | 0.00193392 | -1.624256041 | 152.7125401 | 156.913063 | 94.33580198 | 96.28270295 |
| TMED10 | transmembrane emp24-like trafficking protein 10 (yeast) | 0.0050617 | -1.624338363 | 254.6014917 | 271.1640404 | 140.0831613 | 186.7902069 |
| PPM1A | protein phosphatase. Mg2+/Mn2+ dependent. 1A | 0.00222198 | -1.624548292 | 68.46959763 | 62.56641454 | 43.23945181 | 37.53994329 |
| CD74 | CD74 molecule. major histocompatibility complex. class II invariant chain | 0.00259805 | -1.624848895 | 97.26964185 | 106.1170908 | 69.17318162 | 56.51958204 |
| EIF2B3 | eukaryotic translation initiation factor 2B. subunit 3 gamma. 58kDa | 0.00707673 | -1.625111057 | 308.1344617 | 270.8396703 | 207.856434 | 152.0277914 |
| ACTB | actin. beta | 0.00164815 | -1.626016199 | 7060.054017 | 7953.031849 | 4552.172531 | 4665.221794 |
| NET1 | neuroepithelial cell transforming 1 | 0.00080347 | -1.626812505 | 110.9107848 | 112.2264257 | 66.31762526 | 70.91938142 |
| TAP2 | transporter 2. ATP-binding cassette. sub-family B (MDR/TAP) | 0.07023769 | -1.626885059 | 36.32224421 | 23.87574574 | 13.82372064 | 23.70232392 |
| CHRNA5 | cholinergic receptor. nicotinic. alpha 5 | 0.0022585 | -1.626962788 | 56.03597408 | 57.45268021 | 32.98364432 | 36.87426233 |
| C1orf55 | chromosome 1 open reading frame 55 | 0.00105129 | -1.626999831 | 521.7668748 | 482.8504655 | 317.0375512 | 300.1949883 |
| CCDC59 | coiled-coil domain containing 59 | 0.00205318 | -1.627305064 | 1003.761085 | 1003.761085 | 643.6253813 | 591.1389756 |
| PPM1A | protein phosphatase. Mg2+/Mn2+ dependent. 1A | 0.00298017 | -1.627419735 | 88.75132981 | 84.76238213 | 47.79536276 | 59.42827219 |
| AURKB | aurora kinase B | 0.00172371 | -1.627420543 | 355.3089302 | 385.0135229 | 212.0719151 | 243.556296 |
| HPS4 | Hermansky-Pudlak syndrome 4 | 0.00263443 | -1.62744241 | 550.9324324 | 685.651954 | 374.1947296 | 381.14755 |
| ABCF2 | ATP-binding cassette. sub-family F (GCN20). member 2 | 0.00328909 | -1.627670213 | 257.5331051 | 239.5219911 | 135.520403 | 171.8070344 |
| C6orf48 | chromosome 6 open reading frame 48 | 0.05943421 | -1.628071271 | 61.32209973 | 58.61404591 | 34.77766511 | 38.99163718 |
| ARHGAP11B | Rho GTPase activating protein 11B | 0.01117796 | -1.628242907 | 54.71535273 | 75.99392658 | 36.75303057 | 42.67333081 |
| PQLC2 | PQ loop repeat containing 2 | 0.01090677 | -1.628388983 | 26.25858691 | 37.23485735 | 18.14067256 | 20.32597328 |
| SMC3 | structural maintenance of chromosomes 3 | 0.00311931 | -1.62842599 | 41.13160302 | 40.15117769 | 28.14866228 | 22.12483868 |
| TMTC4 | transmembrane and tetratricopeptide repeat containing 4 | 0.00434593 | -1.628901007 | 77.89823719 | 76.22142201 | 47.17044225 | 47.4400726 |
| SNX16 | sorting nexin 16 | 0.00532482 | -1.631373539 | 50.71414502 | 45.06556572 | 26.76933242 | 32.079646 |
| ADSS | adenylosuccinate synthase | 0.01565584 | -1.632288262 | 121.3392802 | 104.1745431 | 55.90461777 | 84.86363327 |
| MBTPS2 | membrane-bound transcription factor peptidase. site 2 | 0.03381525 | -1.632389726 | 110.9319211 | 64.13087762 | 48.53572768 | 55.00655757 |
| C16orf72 | chromosome 16 open reading frame 72 | 0.00287966 | -1.632629946 | 561.2208082 | 513.5179574 | 368.0082329 | 293.8030833 |
| LOC732146 | hypothetical protein LOC732146 | 0.06866044 | -1.632710761 | 30.86222108 | 38.8242424 | 16.06811241 | 27.97349925 |
| MCM10 | minichromosome maintenance complex component 10 | 0.00241173 | -1.632774382 | 38.09339023 | 35.04528728 | 22.04883596 | 22.71125994 |
| TRIB2 | tribbles homolog 2 (Drosophila) | 0.00853816 | -1.632942607 | 121.7374589 | 162.4086498 | 80.34128999 | 92.28959617 |
| PINX1 | PIN2/TERF1 interacting. telomerase inhibitor 1 | 0.00405723 | -1.633542766 | 182.3147153 | 153.5083098 | 114.5329177 | 91.5719097 |
| LIG4 | ligase IV. DNA. ATP-dependent | 0.0058649 | -1.633731698 | 55.12379954 | 45.40824731 | 28.42266076 | 32.9949657 |
| LOC100130131 | hypothetical LOC100130131 | 0.00125008 | -1.634604612 | 54.51880924 | 52.13882097 | 33.83017428 | 31.44690386 |
| YIPF1 | Yip1 domain family. member 1 | 0.00106869 | -1.635120133 | 2285.786833 | 2228.332415 | 1468.131603 | 1297.631517 |
| CWC27 | CWC27 spliceosome-associated protein homolog (S. cerevisiae) | 0.00154917 | -1.635393762 | 709.103153 | 686.2574453 | 423.2827657 | 429.8542505 |
| H2AFY | H2A histone family. member Y | 0.00078799 | -1.635628128 | 429.3936574 | 447.7537231 | 276.1936671 | 260.2028047 |
| SRI | sorcin | 0.00553268 | -1.635672946 | 138.4350905 | 158.5550153 | 78.98640889 | 103.8677832 |
| CMAH | cytidine monophosphate-N-acetylneuraminic acid hydroxylase (CMP-N-acetylneuraminate monooxygenase) pseudogene | 0.00238104 | -1.635983854 | 49.1381232 | 61.53054013 | 33.24405707 | 33.9810854 |
| NAMPT | nicotinamide phosphoribosyltransferase | 0.00607127 | -1.636185499 | 142.6900222 | 139.8202282 | 73.2886715 | 101.6861218 |
| CKAP5 | cytoskeleton associated protein 5 | 0.0026205 | -1.636409032 | 69.39562042 | 80.10110401 | 48.97440722 | 42.38553225 |
| ACTL6A | actin-like 6A | 0.00070177 | -1.636425455 | 1254.712268 | 1202.384606 | 761.9226985 | 739.4080717 |
| SH3BP2 | SH3-domain binding protein 2 | 0.04033357 | -1.636475202 | 57.56516424 | 34.7636013 | 27.89966917 | 26.78345489 |
| PSPH | phosphoserine phosphatase | 0.0015778 | -1.636719118 | 243.3625749 | 204.9451552 | 134.5669896 | 138.3581035 |
| COPZ1 | coatomer protein complex. subunit zeta 1 | 0.00074787 | -1.63823511 | 1105.18718 | 1113.335986 | 697.1635226 | 657.6199366 |
| SLC43A3 | solute carrier family 43. member 3 | 0.0098035 | -1.638741863 | 230.9709505 | 224.0042041 | 157.8701618 | 122.0372409 |
| ZWINT | ZW10 interactor | 0.00714959 | -1.638869752 | 63.06110694 | 69.93614656 | 42.427136 | 38.70175806 |
| ANKRD28 | ankyrin repeat domain 28 | 0.00327089 | -1.640302347 | 107.6519432 | 96.59355356 | 65.31870745 | 59.16769113 |
| MND1 | meiotic nuclear divisions 1 homolog (S. cerevisiae) | 0.01321652 | -1.640982569 | 442.0482694 | 296.5259386 | 225.8333921 | 215.5441748 |
| MDM1 | Mdm1 nuclear protein homolog (mouse) | 0.01918173 | -1.641437326 | 47.49078889 | 46.3175043 | 23.98663065 | 34.03585594 |
| DNAJC19 | DnaJ (Hsp40) homolog. subfamily C. member 19 | 0.00621527 | -1.642180085 | 49.98157905 | 42.11554748 | 28.98060487 | 26.93415873 |
| MIR1228 | microRNA 1228 | 0.00850775 | -1.642206563 | 89.76267616 | 79.57304018 | 59.31745774 | 44.65022446 |
| CBLL1 | Cas-Br-M (murine) ecotropic retroviral transforming sequence-like 1 | 0.06494674 | -1.642561486 | 113.5067868 | 65.93482698 | 41.26884184 | 67.21576545 |
| ANP32C | acidic (leucine-rich) nuclear phosphoprotein 32 family. member C | 0.00506836 | -1.642665364 | 113.3886911 | 124.2807213 | 71.69528823 | 72.84247474 |
| PAG1 | phosphoprotein associated with glycosphingolipid microdomains 1 | 0.00149503 | -1.642759575 | 41.56265718 | 48.69478696 | 26.58528039 | 28.2095732 |
| PSMA4 | proteasome (prosome. macropain) subunit. alpha type. 4 | 0.00463475 | -1.643033096 | 1785.424105 | 2017.497551 | 1191.092907 | 1120.253084 |
| FAM18B1 | family with sequence similarity 18. member B1 | 0.00262459 | -1.643142349 | 233.8453264 | 187.2365828 | 121.9050423 | 133.0293146 |
| MRPL42 | mitochondrial ribosomal protein L42 | 0.00289133 | -1.643877055 | 206.8445328 | 164.992844 | 118.6987552 | 106.3957604 |
| BCAT1 | branched chain amino-acid transaminase 1. cytosolic | 0.00099085 | -1.644387761 | 2805.991508 | 2660.693597 | 1642.316644 | 1681.186335 |
| ALKBH8 | alkB. alkylation repair homolog 8 (E. coli) | 0.00174927 | -1.644405408 | 38.00913008 | 31.77218128 | 21.03478463 | 21.2314228 |
| RAP1GDS1 | RAP1. GTP-GDP dissociation stimulator 1 | 0.0016799 | -1.645091443 | 192.9339153 | 183.1097051 | 117.8373778 | 110.7788327 |
| CNBP | CCHC-type zinc finger. nucleic acid binding protein | 0.01690718 | -1.645139001 | 134.7295029 | 103.4306577 | 58.91446788 | 87.39464805 |
| USP4 | ubiquitin specific peptidase 4 (proto-oncogene) | 0.01268883 | -1.645181353 | 70.01310958 | 49.35736065 | 37.61617117 | 33.94135296 |
| ARMCX3 | armadillo repeat containing. X-linked 3 | 0.00106578 | -1.64527823 | 263.2775009 | 232.6703244 | 149.1578215 | 151.7155192 |
| CYBB | cytochrome b-245. beta polypeptide | 0.00601452 | -1.645348976 | 297.5412096 | 264.0920825 | 147.6578505 | 196.5757371 |
| PPAP2A | phosphatidic acid phosphatase type 2A | 0.00094994 | -1.645528836 | 47.64580697 | 48.75582427 | 30.30301333 | 28.31094582 |
| PIM3 | pim-3 oncogene | 0.12321211 | -1.645855457 | 17.3269386 | 38.4838829 | 20.593403 | 11.95333853 |
| POLR1D | polymerase (RNA) I polypeptide D. 16kDa | 0.00202901 | -1.645907592 | 394.4560687 | 423.379883 | 239.2526671 | 257.6684088 |
| EIF2S2 | eukaryotic translation initiation factor 2. subunit 2 beta. 38kDa | 0.0075185 | -1.646199846 | 132.8761349 | 95.08344075 | 63.82206359 | 73.04927671 |
| DOM3Z | dom-3 homolog Z (C. elegans) | 0.0050001 | -1.646578884 | 148.1951105 | 158.6485484 | 108.0427757 | 80.26184923 |
| STX5 | syntaxin 5 | 0.00318772 | -1.646645612 | 292.9166205 | 368.0082329 | 186.1182821 | 213.605402 |
| ARL5B | ADP-ribosylation factor-like 5B | 0.02801507 | -1.647637023 | 51.72102093 | 41.6864154 | 27.08503627 | 29.32304959 |
| LMAN1 | lectin. mannose-binding. 1 | 0.01445848 | -1.647700005 | 173.0993034 | 192.8318724 | 88.88426582 | 138.3225424 |
| AGA | aspartylglucosaminidase | 0.0007364 | -1.648490572 | 118.3491903 | 120.245724 | 70.05363968 | 74.75345903 |
| LOC652826 | similar to 26S protease regulatory subunit 6B (MIP224) (MB67-interacting protein) (TAT-binding protein 7) (TBP-7) | 0.00167828 | -1.648785545 | 273.8332856 | 230.7827724 | 152.6558061 | 152.2817833 |
| HSPA13 | heat shock protein 70kDa family. member 13 | 0.01184904 | -1.649648301 | 61.22277292 | 63.36715352 | 45.49972167 | 31.3318275 |
| RAD21 | RAD21 homolog (S. pombe) | 0.00127966 | -1.650217224 | 270.2391275 | 303.9610175 | 164.0992993 | 183.8133972 |
| MKI67 | antigen identified by monoclonal antibody Ki-67 | 0.00264311 | -1.650473918 | 70.99457188 | 68.19075846 | 47.60829126 | 37.32937683 |
| SH3BP1 | SH3-domain binding protein 1 | 0.00231079 | -1.650655356 | 63.50144217 | 60.75895829 | 37.40483841 | 37.85762056 |
| SSRP1 | structure specific recognition protein 1 | 0.01124675 | -1.651069122 | 315.5622218 | 362.1182975 | 187.6023047 | 223.4430213 |
| C1orf189 | chromosome 1 open reading frame 189 | 0.00510171 | -1.651085149 | 27.91508189 | 30.10010516 | 17.87860603 | 17.23988333 |
| PDCD4 | programmed cell death 4 (neoplastic transformation inhibitor) | 0.00074766 | -1.652636847 | 317.5153919 | 336.9486114 | 194.6867979 | 201.2042217 |
| CLDN20 | claudin 20 | 0.01437358 | -1.652884073 | 42.53832841 | 29.46867333 | 20.94317807 | 21.90854971 |
| PPPDE2 | PPPDE peptidase domain containing 2 | 0.00249923 | -1.653247562 | 1097.832318 | 1008.071104 | 710.4221937 | 569.9475292 |
| PPM1A | protein phosphatase. Mg2+/Mn2+ dependent. 1A | 0.00331553 | -1.653322838 | 40.28656282 | 47.73021694 | 23.83367126 | 29.51531004 |
| IP6K2 | inositol hexakisphosphate kinase 2 | 0.02473349 | -1.653393098 | 45.53727172 | 53.6706617 | 23.33700586 | 38.30950856 |
| FKBP1A | FK506 binding protein 1A. 12kDa | 0.01216617 | -1.653779287 | 991.4938678 | 1294.256119 | 576.8562115 | 813.3689502 |
| TMEM18 | transmembrane protein 18 | 0.0010662 | -1.654144386 | 66.27678059 | 59.22778591 | 36.35902143 | 39.45734739 |
| FAM60A | family with sequence similarity 60. member A | 0.00239732 | -1.654309415 | 225.9668153 | 204.5005391 | 129.3643159 | 130.5242311 |
| ZNHIT2 | zinc finger. HIT type 2 | 0.00445412 | -1.654311814 | 55.40917 | 66.21048712 | 35.25777294 | 38.02058623 |
| KIAA2026 | KIAA2026 | 0.04213768 | -1.655816598 | 58.62805285 | 111.1338 | 49.67494899 | 47.83984259 |
| COMT | catechol-O-methyltransferase | 0.01243509 | -1.656049682 | 124.2096972 | 171.4744229 | 99.72750475 | 77.87412441 |
| PPID | peptidylprolyl isomerase D | 0.00268647 | -1.656334413 | 69.84004185 | 56.23647354 | 35.67479912 | 40.1296141 |
| CSNK2A1 | casein kinase 2. alpha 1 polypeptide | 0.00107153 | -1.656386671 | 45.15058083 | 46.0388102 | 29.01098119 | 26.11569342 |
| SGOL1 | shugoshin-like 1 (S. pombe) | 0.00711939 | -1.657028512 | 132.6906228 | 132.0703506 | 83.6230393 | 76.32374156 |
| C16orf59 | chromosome 16 open reading frame 59 | 0.00318966 | -1.657832337 | 237.3938487 | 239.6598184 | 163.8687191 | 126.3244498 |
| PDCD10 | programmed cell death 10 | 0.00593775 | -1.657972598 | 75.30493507 | 78.21840797 | 51.19319822 | 41.85674492 |
| FIGNL1 | fidgetin-like 1 | 0.00151216 | -1.658794608 | 168.9941532 | 158.2289609 | 90.2110939 | 107.7240012 |
| SETD3 | SET domain containing 3 | 0.02160462 | -1.658969605 | 59.77678591 | 60.26831759 | 29.28848832 | 44.69385476 |
| CDK2 | cyclin-dependent kinase 2 | 0.00337072 | -1.659074038 | 87.48427756 | 86.39600742 | 47.12297983 | 58.27193004 |
| SLC5A12 | solute carrier family 5 (sodium/glucose cotransporter). member 12 | 0.01232672 | -1.659829878 | 44.4236337 | 28.95370287 | 20.75578039 | 22.49323758 |
| SLC1A4 | solute carrier family 1 (glutamate/neutral amino acid transporter). member 4 | 0.0007211 | -1.659876319 | 136.8085804 | 129.477906 | 83.19425958 | 77.27947886 |
| ORC6L | origin recognition complex. subunit 6 like (yeast) | 0.00151201 | -1.660051619 | 136.9487118 | 126.5969341 | 73.01354581 | 86.16571867 |
| RNF214 | ring finger protein 214 | 0.00128507 | -1.660260957 | 66.91147601 | 65.74847676 | 40.63469861 | 39.27684118 |
| FNDC3A | fibronectin type III domain containing 3A | 0.00480466 | -1.660611719 | 231.6932402 | 231.9272334 | 160.2449645 | 121.6030652 |
| THOC3 | THO complex 3 | 0.00152386 | -1.66091093 | 346.1206527 | 352.3419417 | 225.0842012 | 196.405732 |
| RFC3 | replication factor C (activator 1) 3. 38kDa | 0.01621156 | -1.661097905 | 205.5993156 | 161.5234533 | 99.59513796 | 120.8449919 |
| CNOT4 | CCR4-NOT transcription complex. subunit 4 | 0.00059733 | -1.661954565 | 35.24856717 | 35.44357436 | 21.79842319 | 20.74988086 |
| LOC643668 | proteasome (prosome. macropain) 26S subunit. ATPase. 1 pseudogene | 0.0035282 | -1.662215127 | 189.455891 | 167.3384534 | 112.9924774 | 101.5499873 |
| HSPB11 | heat shock protein family B (small). member 11 | 0.00101226 | -1.662492414 | 618.5162446 | 635.0401034 | 383.624121 | 370.4477928 |
| FGF12 | fibroblast growth factor 12 | 0.00077697 | -1.663063792 | 120.1572846 | 111.5899239 | 72.42516814 | 66.93723339 |
| RAD51C | RAD51 homolog C (S. cerevisiae) | 0.00178568 | -1.664419774 | 315.1722297 | 375.0944733 | 218.3459418 | 195.441934 |
| DENND1A | DENN/MADD domain containing 1A | 0.00553434 | -1.664456883 | 114.5329177 | 90.85748133 | 54.1569491 | 69.35722823 |
| SASS6 | spindle assembly 6 homolog (C. elegans) | 0.0062671 | -1.665082115 | 102.6299338 | 83.70256499 | 48.28324044 | 64.171929 |
| LOC727848 | similar to actin-like protein | 0.00641502 | -1.665139559 | 42.31452271 | 34.64167519 | 26.71973423 | 19.7858595 |
| HMGN2 | high-mobility group nucleosomal binding domain 2 | 0.00156764 | -1.665355139 | 110.952827 | 132.0299073 | 71.52392733 | 73.84916925 |
| PRKDC | protein kinase. DNA-activated. catalytic polypeptide | 0.00114106 | -1.665479714 | 76.76985383 | 88.09713307 | 48.09403672 | 50.69703542 |
| THRAP3 | thyroid hormone receptor associated protein 3 | 0.00644729 | -1.666384412 | 133.5787716 | 143.3862522 | 80.79412665 | 85.37186697 |
| ZNF143 | zinc finger protein 143 | 0.00539182 | -1.667382831 | 190.9469303 | 216.3404806 | 104.5812302 | 142.0777512 |
| KIAA0101 | KIAA0101 | 0.00076227 | -1.668014532 | 2564.437738 | 2328.606974 | 1463.914219 | 1466.131971 |
| LYAR | Ly1 antibody reactive homolog (mouse) | 0.00093301 | -1.668371509 | 438.5661277 | 430.6493066 | 273.8332856 | 247.7918806 |
| METTL9 | methyltransferase like 9 | 0.00070181 | -1.668943462 | 92.88388252 | 96.5705637 | 54.5722464 | 59.01060219 |
| OSGIN2 | oxidative stress induced growth inhibitor family member 2 | 0.00122888 | -1.669161445 | 110.8590825 | 116.7059941 | 73.59127068 | 63.10175689 |
| HIF1A | hypoxia inducible factor 1. alpha subunit (basic helix-loop-helix transcription factor) | 0.01058612 | -1.670506219 | 79.31278337 | 63.74547996 | 42.30555745 | 42.82515771 |
| ARF4 | ADP-ribosylation factor 4 | 0.0009214 | -1.671388638 | 301.2907817 | 301.8520391 | 178.3030479 | 182.5855063 |
| KCNK3 | potassium channel. subfamily K. member 3 | 0.00809625 | -1.672538184 | 27.28220897 | 31.36592258 | 20.53257819 | 14.89848229 |
| PVT1 | Pvt1 oncogene (non-protein coding) | 0.01389547 | -1.673240465 | 44.11273836 | 46.34408885 | 28.08972441 | 25.99526029 |
| SELT | selenoprotein T | 0.00314088 | -1.674011673 | 420.5930786 | 390.8430389 | 255.0036897 | 230.03879 |
| SNTB1 | syntrophin. beta 1 (dystrophin-associated protein A1. 59kDa. basic component 1) | 0.00100239 | -1.674147787 | 266.8280576 | 277.0800696 | 167.9255532 | 157.0841654 |
| MAT2B | methionine adenosyltransferase II. beta | 0.00051823 | -1.674567324 | 186.835824 | 184.5877865 | 110.5875773 | 111.2119728 |
| APOE | apolipoprotein E | 0.02624724 | -1.674922428 | 36.70523956 | 32.8755032 | 24.67766366 | 17.43038722 |
| PRIM1 | primase. DNA. polypeptide 1 (49kDa) | 0.00098549 | -1.675548279 | 357.6061908 | 355.2267651 | 200.5094717 | 225.6638143 |
| LOC643668 | proteasome (prosome. macropain) 26S subunit. ATPase. 1 pseudogene | 0.00341564 | -1.675819519 | 486.7993346 | 409.4541038 | 243.6039666 | 291.350897 |
| C22orf32 | chromosome 22 open reading frame 32 | 0.0057817 | -1.676257946 | 87.64916906 | 77.53721358 | 41.72848112 | 57.96204731 |
| KLHL29 | kelch-like 29 (Drosophila) | 0.00545345 | -1.677219753 | 48.77519137 | 55.32544015 | 27.06308422 | 35.44595108 |
| RMND5A | required for meiotic nuclear division 5 homolog A (S. cerevisiae) | 0.00544674 | -1.678244974 | 33.0554162 | 25.94924897 | 15.93548468 | 19.11135304 |
| LOC121456 | solute carrier family 9 (sodium/hydrogen exchanger). member 7 pseudogene | 0.0012245 | -1.678660302 | 28.5452149 | 29.91538225 | 18.20500522 | 16.64605883 |
| DUS3L | dihydrouridine synthase 3-like (S. cerevisiae) | 0.00064074 | -1.679928994 | 156.7376571 | 146.1592914 | 88.79926282 | 91.41312747 |
| LOC100287833 | similar to hCG1644658 | 0.0005911 | -1.6806429 | 1704.814427 | 1755.357782 | 999.5828811 | 1059.919936 |
| DEPDC6 | DEP domain containing 6 | 0.00253662 | -1.680729159 | 443.126893 | 402.1615246 | 250.591474 | 251.7484852 |
| TMEM183A | transmembrane protein 183A | 0.00580411 | -1.681444928 | 45.60147371 | 36.77562658 | 27.38868935 | 21.65720532 |
| EID1 | EP300 interacting inhibitor of differentiation 1 | 0.01199828 | -1.682371544 | 84.35975661 | 63.32279772 | 47.47902524 | 39.75115853 |
| CLK1 | CDC-like kinase 1 | 0.00089721 | -1.683260392 | 948.9252789 | 829.5681649 | 525.5629642 | 528.6359953 |
| FAM3C | family with sequence similarity 3. member C | 0.00180363 | -1.68366091 | 465.1373259 | 418.0104635 | 269.9715238 | 254.0628288 |
| LEF1 | lymphoid enhancer-binding factor 1 | 0.00061279 | -1.685292933 | 1051.489831 | 1049.420811 | 595.3779355 | 652.5461237 |
| NCAPG | non-SMC condensin I complex. subunit G | 0.0010069 | -1.685779625 | 729.408817 | 626.5024717 | 398.1510401 | 403.8725551 |
| PANK1 | pantothenate kinase 1 | 0.00171264 | -1.686111246 | 92.95670445 | 108.6993224 | 64.60263536 | 55.0154754 |
| GORAB | golgin. RAB6-interacting | 0.04249981 | -1.686707518 | 71.93856028 | 45.68922904 | 39.29679728 | 29.39942619 |
| RPRD1B | regulation of nuclear pre-mRNA domain containing 1B | 0.01056873 | -1.687513114 | 123.9083321 | 100.1805299 | 54.39628373 | 80.13464041 |
| RPS7 | ribosomal protein S7 | 0.00851847 | -1.688445434 | 591.8881741 | 554.3875298 | 335.9569478 | 342.6063661 |
| SAP30L | SAP30-like | 0.0085338 | -1.689246452 | 105.8261491 | 115.0685193 | 57.6706 | 73.99613168 |
| CTNNAL1 | catenin (cadherin-associated protein). alpha-like 1 | 0.00139301 | -1.689593272 | 403.1498165 | 370.5318617 | 208.5165747 | 250.9499423 |
| BAT1 | HLA-B associated transcript 1 | 0.00133846 | -1.69155607 | 1387.309472 | 1473.596606 | 892.6553134 | 800.3784263 |
| CSAD | cysteine sulfinic acid decarboxylase | 0.00848107 | -1.691798627 | 26.02225794 | 33.19295971 | 17.45520367 | 17.28895322 |
| LOC728115 | similar to family with sequence similarity 60. member A | 0.01423215 | -1.69188969 | 277.2432548 | 229.7998264 | 185.2322029 | 120.1572846 |
| ACTL6A | actin-like 6A | 0.00176036 | -1.691994133 | 601.1679062 | 606.2998851 | 380.2845185 | 334.7934267 |
| PPM1B | protein phosphatase. Mg2+/Mn2+ dependent. 1B | 0.00201272 | -1.693213026 | 47.08620917 | 42.21941748 | 28.82254954 | 24.05749301 |
| TRIP13 | thyroid hormone receptor interactor 13 | 0.00166796 | -1.694402593 | 330.5861051 | 303.8483456 | 189.2021431 | 184.9192849 |
| TFAP4 | transcription factor AP-4 (activating enhancer binding protein 4) | 0.0074892 | -1.695121642 | 35.07307301 | 46.74968183 | 20.76940735 | 27.47430006 |
| TRIM35 | tripartite motif-containing 35 | 0.06925958 | -1.695127621 | 23.77308879 | 28.78152952 | 15.91383254 | 14.96304209 |
| MX1 | myxovirus (influenza virus) resistance 1. interferon-inducible protein p78 (mouse) | 0.03081548 | -1.695354388 | 867.7605257 | 1334.725455 | 620.4448426 | 649.482816 |
| TRIM39 | tripartite motif-containing 39 | 0.00080139 | -1.69584525 | 64.74316472 | 60.02403362 | 39.03475278 | 34.61746241 |
| C11orf51 | chromosome 11 open reading frame 51 | 0.00071154 | -1.695948861 | 150.6730581 | 151.0819415 | 89.27244387 | 88.65540768 |
| ACSL5 | acyl-CoA synthetase long-chain family member 5 | 0.00178034 | -1.696636222 | 98.24079322 | 105.583533 | 67.05793882 | 53.73538701 |
| TMED2 | transmembrane emp24 domain trafficking protein 2 | 0.00069627 | -1.697737098 | 1894.603681 | 1768.222267 | 1025.64582 | 1133.22707 |
| MED8 | mediator complex subunit 8 | 0.0031967 | -1.698074714 | 98.4078189 | 101.6861218 | 62.55286556 | 55.47923841 |
| MCM3 | minichromosome maintenance complex component 3 | 0.0005799 | -1.698455497 | 568.6585987 | 575.5154063 | 351.9094738 | 322.3808692 |
| CEP57 | centrosomal protein 57kDa | 0.00108272 | -1.698466114 | 82.40683476 | 70.52296424 | 44.70609911 | 45.06228826 |
| C3orf26 | chromosome 3 open reading frame 26 | 0.00235683 | -1.699554457 | 481.4056759 | 432.5922767 | 279.0422598 | 258.3746003 |
| E2F2 | E2F transcription factor 2 | 0.00069299 | -1.699746336 | 1896.640924 | 2064.897408 | 1218.757815 | 1112.238581 |
| DNAJC10 | DnaJ (Hsp40) homolog. subfamily C. member 10 | 0.00725274 | -1.699997049 | 1370.836274 | 938.239519 | 709.4899254 | 627.2732123 |
| MASTL | microtubule associated serine/threonine kinase-like | 0.00246174 | -1.700221919 | 29.02607051 | 34.45955353 | 20.27750676 | 17.06365713 |
| WHSC1L1 | Wolf-Hirschhorn syndrome candidate 1-like 1 | 0.00047017 | -1.700476714 | 46.96831034 | 49.77082615 | 28.52793417 | 28.33792748 |
| RAP1GDS1 | RAP1. GTP-GDP dissociation stimulator 1 | 0.00076168 | -1.700720807 | 113.6925436 | 123.4932361 | 68.9583468 | 70.39183117 |
| CHMP2A | chromatin modifying protein 2A | 0.02155729 | -1.701072687 | 79.9059702 | 71.34348884 | 34.09741837 | 57.77850907 |
| PIGA | phosphatidylinositol glycan anchor biosynthesis. class A | 0.0103657 | -1.702822706 | 38.08728306 | 32.89632595 | 16.83570742 | 25.66593884 |
| MCM3 | minichromosome maintenance complex component 3 | 0.0007308 | -1.703408609 | 3142.513049 | 3261.686948 | 2006.200249 | 1760.78938 |
| MIER2 | mesoderm induction early response 1. family member 2 | 0.01401502 | -1.703709369 | 55.9758992 | 57.03696507 | 41.99797659 | 26.19015673 |
| IQCK | IQ motif containing K | 0.00110526 | -1.705228756 | 37.49118839 | 40.04181889 | 21.87724922 | 23.59853237 |
| ANGPTL6 | angiopoietin-like 6 | 0.00206582 | -1.70582509 | 89.70484076 | 103.1815036 | 57.98698773 | 54.85524858 |
| PLK4 | polo-like kinase 4 | 0.00055884 | -1.705987009 | 352.6818644 | 372.5165625 | 211.1153573 | 213.8244918 |
| RAB1B | RAB1B. member RAS oncogene family | 0.01584797 | -1.708290583 | 47.17630305 | 69.10306739 | 32.99206888 | 33.86009607 |
| ASS1 | argininosuccinate synthase 1 | 0.0007905 | -1.70853243 | 3107.965433 | 3151.533955 | 1966.204336 | 1706.565915 |
| UBE2I | ubiquitin-conjugating enzyme E2I (UBC9 homolog. yeast) | 0.00327696 | -1.708794537 | 52.1632217 | 58.89007874 | 33.44198944 | 31.45826324 |
| RFC5 | replication factor C (activator 1) 5. 36.5kDa | 0.0026088 | -1.709184777 | 548.4353907 | 570.8359437 | 285.7051613 | 375.0944733 |
| RAD54B | RAD54 homolog B (S. cerevisiae) | 0.01163214 | -1.710017779 | 60.45777381 | 63.48676762 | 28.80581691 | 45.56736547 |
| LOC344593 | protein tyrosine phosphatase. non-receptor type 11 pseudogene | 0.05277519 | -1.710049106 | 28.29622011 | 38.06404263 | 19.11546816 | 19.26820391 |
| FASTKD2 | FAST kinase domains 2 | 0.0030358 | -1.710983508 | 84.66689491 | 77.01718973 | 51.28532658 | 43.43266996 |
| ARHGEF3 | Rho guanine nucleotide exchange factor (GEF) 3 | 0.00262679 | -1.712124266 | 31.45304241 | 39.75115853 | 21.52864383 | 19.81185829 |
| SEC24D | SEC24 family. member D (S. cerevisiae) | 0.00767513 | -1.712267558 | 178.472082 | 147.5381707 | 111.3307229 | 80.67080652 |
| BYSL | bystin-like | 0.0150817 | -1.713259849 | 373.5350572 | 357.6908194 | 222.212169 | 204.844755 |
| GMEB2 | glucocorticoid modulatory element binding protein 2 | 0.00212899 | -1.71376586 | 308.8417976 | 296.6950088 | 163.9482149 | 190.2990612 |
| ZEB2 | zinc finger E-box binding homeobox 2 | 0.00327715 | -1.714432528 | 90.8803057 | 88.62047293 | 46.22286058 | 59.27973148 |
| RAD51C | RAD51 homolog C (S. cerevisiae) | 0.00044651 | -1.714560856 | 387.3060069 | 393.6844427 | 235.007502 | 220.7064121 |
| RAD51C | RAD51 homolog C (S. cerevisiae) | 0.00150305 | -1.714800573 | 392.103679 | 411.8485983 | 250.2734437 | 219.4302511 |
| LRRC40 | leucine rich repeat containing 40 | 0.00230994 | -1.715201604 | 79.46729763 | 64.99673779 | 38.9156647 | 45.11544062 |
| RAD54L | RAD54-like (S. cerevisiae) | 0.00052496 | -1.715895154 | 138.7028527 | 140.8276384 | 84.81698336 | 78.21840797 |
| MAZ | MYC-associated zinc finger protein (purine-binding transcription factor) | 0.00054725 | -1.716101451 | 60.94616287 | 63.87247524 | 38.06404263 | 34.72636186 |
| PSCA | prostate stem cell antigen | 0.02567344 | -1.717427828 | 33.15722109 | 29.76389592 | 18.82969225 | 17.76919238 |
| TBC1D17 | TBC1 domain family. member 17 | 0.03672579 | -1.717508326 | 41.49105086 | 79.06482953 | 33.27247048 | 33.42373834 |
| EZH2 | enhancer of zeste homolog 2 (Drosophila) | 0.00070633 | -1.717542956 | 385.3014641 | 384.5624845 | 216.2638555 | 232.2567893 |
| SLMO2 | slowmo homolog 2 (Drosophila) | 0.00602948 | -1.71785587 | 537.7566459 | 572.5094488 | 327.8993828 | 318.1664626 |
| SSR1 | signal sequence receptor. alpha | 0.00084778 | -1.717885771 | 801.925756 | 682.8096957 | 437.9510988 | 423.6621408 |
| HSF2 | heat shock transcription factor 2 | 0.0025972 | -1.719587095 | 212.2253654 | 171.3178606 | 101.8752392 | 120.6932374 |
| NUCKS1 | nuclear casein kinase and cyclin-dependent kinase substrate 1 | 0.00073921 | -1.719858362 | 2843.42043 | 3181.884644 | 1698.929545 | 1800.382473 |
| ARAP2 | ArfGAP with RhoGAP domain. ankyrin repeat and PH domain 2 | 0.00189012 | -1.720006563 | 139.5776714 | 110.7411346 | 69.92478026 | 74.7194384 |
| CSNK2A1 | casein kinase 2. alpha 1 polypeptide | 0.00168976 | -1.720309203 | 324.0991713 | 365.6955004 | 220.9403163 | 181.2631572 |
| LOC644037 | heterogeneous nuclear ribonucleoprotein A1 pseudogene | 0.00136576 | -1.722349222 | 76.07618349 | 83.93537629 | 42.14881006 | 51.07001843 |
| TP63 | tumor protein p63 | 0.00062695 | -1.722616526 | 1626.04904 | 1718.908844 | 967.6373307 | 973.4119013 |
| CTCF | CCCTC-binding factor (zinc finger protein) | 0.00160681 | -1.724043454 | 308.4334232 | 305.2634153 | 164.5259783 | 192.5330518 |
| PCBP4 | poly(rC) binding protein 4 | 0.00291636 | -1.724851684 | 29.38820271 | 36.32224421 | 17.53554048 | 20.4608198 |
| C7orf64 | chromosome 7 open reading frame 64 | 0.00118925 | -1.726020938 | 58.46854495 | 62.94331019 | 32.17839118 | 38.38976191 |
| FEN1 | flap structure-specific endonuclease 1 | 0.00174967 | -1.72644826 | 260.834883 | 227.3325471 | 127.5505124 | 155.9688777 |
| SLC20A1 | solute carrier family 20 (phosphate transporter). member 1 | 0.00104221 | -1.726694589 | 660.0695039 | 627.8902598 | 352.4063425 | 394.4560687 |
| PPP3R1 | protein phosphatase 3. regulatory subunit B. alpha | 0.00090786 | -1.726892114 | 484.4119454 | 439.6043922 | 257.7158719 | 277.0800696 |
| FAM60A | family with sequence similarity 60. member A | 0.00045331 | -1.727057573 | 1760.78938 | 1757.145653 | 1003.761085 | 1033.406497 |
| TATDN3 | TatD DNase domain containing 3 | 0.00059976 | -1.727436325 | 342.7232314 | 380.7371929 | 201.4479436 | 217.0710532 |
| CENPP | centromere protein P | 0.00692975 | -1.728872661 | 46.28162183 | 44.67783191 | 21.73198493 | 31.83283005 |
| EAF2 | ELL associated factor 2 | 0.00079962 | -1.728998782 | 192.1424494 | 203.3704297 | 116.652797 | 112.0538024 |
| PCGF1 | polycomb group ring finger 1 | 0.00168577 | -1.729236092 | 104.4362666 | 115.0067242 | 68.60585649 | 58.5470205 |
| BZW1 | basic leucine zipper and W2 domains 1 | 0.00393709 | -1.729255033 | 66.13547564 | 50.04733186 | 30.41271501 | 36.39503333 |
| HSPA13 | heat shock protein 70kDa family. member 13 | 0.00142695 | -1.729460215 | 79.57304018 | 86.67397689 | 43.2878341 | 53.26815647 |
| DHFR | dihydrofolate reductase | 0.01395567 | -1.729479064 | 65.92036254 | 46.4057138 | 26.52952224 | 38.55058717 |
| FAM117A | family with sequence similarity 117. member A | 0.04816309 | -1.730945037 | 63.62428679 | 104.4999914 | 60.57062658 | 36.63621168 |
| PRMT1 | protein arginine methyltransferase 1 | 0.00042499 | -1.731054854 | 2071.852478 | 2100.44259 | 1239.168798 | 1171.972754 |
| ATP6V1G2 | ATPase. H+ transporting. lysosomal 13kDa. V1 subunit G2 | 0.01339516 | -1.731160201 | 55.65660745 | 68.42058477 | 30.22947557 | 42.03377358 |
| MID1IP1 | MID1 interacting protein 1 (gastrulation specific G12 homolog (zebrafish)) | 0.00104633 | -1.731194431 | 523.0185133 | 519.3157128 | 309.1701268 | 293.1293523 |
| SRSF3 | serine/arginine-rich splicing factor 3 | 0.00132836 | -1.733121583 | 366.8357721 | 316.4551377 | 181.5705527 | 212.8532834 |
| DIAPH3 | diaphanous homolog 3 (Drosophila) | 0.00112789 | -1.733151614 | 81.62448777 | 93.67386521 | 46.68379376 | 54.52549986 |
| ARHGEF10 | Rho guanine nucleotide exchange factor (GEF) 10 | 0.00317012 | -1.733223693 | 120.5965443 | 88.26097442 | 57.52333526 | 61.59568481 |
| PPM1M | protein phosphatase. Mg2+/Mn2+ dependent. 1M | 0.0004322 | -1.734669671 | 157.2838955 | 161.3752471 | 91.5719097 | 92.11393496 |
| KCNK12 | potassium channel. subfamily K. member 12 | 0.00045161 | -1.736044914 | 348.8913525 | 368.6697 | 214.6500126 | 198.8267524 |
| LOC399491 | GPS. PLAT and transmembrane domain-containing protein | 0.00171224 | -1.736769786 | 199.602025 | 161.90015 | 96.92793008 | 110.5296374 |
| IARS | isoleucyl-tRNA synthetase | 0.00811789 | -1.738916402 | 34.87414166 | 31.4327143 | 15.50042879 | 23.38752649 |
| EIF5 | eukaryotic translation initiation factor 5 | 0.00251767 | -1.739469578 | 31.47289522 | 23.31607783 | 15.55559743 | 15.59092282 |
| UBA2 | ubiquitin-like modifier activating enzyme 2 | 0.0009776 | -1.739571436 | 138.5536123 | 124.5243748 | 80.75976711 | 70.59803758 |
| GAR1 | GAR1 ribonucleoprotein homolog (yeast) | 0.00107067 | -1.740013176 | 101.0058587 | 102.7747263 | 54.84933862 | 62.5109969 |
| PLRG1 | pleiotropic regulator 1 (PRL1 homolog. Arabidopsis) | 0.00056278 | -1.740664335 | 1216.705072 | 1172.561726 | 664.0206021 | 709.103153 |
| MXRA7 | matrix-remodelling associated 7 | 0.00581653 | -1.741057937 | 37.62241189 | 28.73178938 | 21.56795573 | 16.53384562 |
| PAG1 | phosphoprotein associated with glycosphingolipid microdomains 1 | 0.00064659 | -1.74142325 | 210.5219752 | 191.6481072 | 120.011265 | 110.8590825 |
| PPM1B | protein phosphatase. Mg2+/Mn2+ dependent. 1B | 0.00992115 | -1.741636133 | 40.3082637 | 53.49172404 | 31.11790396 | 22.84312404 |
| CREB3L4 | cAMP responsive element binding protein 3-like 4 | 0.00329831 | -1.741831388 | 46.32610341 | 40.07563916 | 23.28230296 | 26.28258449 |
| MRPS14 | mitochondrial ribosomal protein S14 | 0.00081246 | -1.741865433 | 136.9122419 | 116.7215305 | 75.36595072 | 69.88578888 |
| TARS | threonyl-tRNA synthetase | 0.0011208 | -1.745098064 | 925.0119693 | 857.5442532 | 551.1917505 | 472.5649543 |
| ST8SIA4 | ST8 alpha-N-acetyl-neuraminide alpha-2.8-sialyltransferase 4 | 0.00046388 | -1.745242165 | 71.77817197 | 67.54814537 | 39.49605347 | 40.30331111 |
| TSPAN7 | tetraspanin 7 | 0.01063868 | -1.746115207 | 44.34833178 | 27.73756979 | 20.96507372 | 19.24436178 |
| CLK2P | CDC-like kinase 2. pseudogene | 0.00301461 | -1.746155 | 33.21062891 | 45.60645487 | 23.03683064 | 21.56329384 |
| ATAD1 | ATPase family. AAA domain containing 1 | 0.00045671 | -1.746849074 | 447.9020025 | 423.5538982 | 242.1305242 | 256.7623707 |
| SKA1 | spindle and kinetochore associated complex subunit 1 | 0.00140235 | -1.747008643 | 273.2672336 | 262.9773454 | 166.1494966 | 141.7153065 |
| SPC25 | SPC25. NDC80 kinetochore complex component. homolog (S. cerevisiae) | 0.01095736 | -1.749244593 | 63.67320719 | 47.59437276 | 29.63758 | 33.41708119 |
| TNKS1BP1 | tankyrase 1 binding protein 1. 182kDa | 0.00601535 | -1.749583806 | 57.11804778 | 66.46967484 | 29.14042333 | 42.5629335 |
| NUPL1 | nucleoporin like 1 | 0.00774014 | -1.749735821 | 24.49615429 | 38.00324625 | 17.04460546 | 17.83966464 |
| HIST2H2BE | histone cluster 2. H2be | 0.00368379 | -1.749930655 | 47.30542238 | 61.45057638 | 28.79978668 | 32.96142175 |
| MNAT1 | menage a trois homolog 1. cyclin H assembly factor (Xenopus laevis) | 0.00120689 | -1.750428779 | 94.503669 | 78.2348889 | 50.47352515 | 47.80756369 |
| VEGFA | vascular endothelial growth factor A | 0.01557018 | -1.75115108 | 52.82593 | 77.30595085 | 42.6654046 | 31.21310524 |
| TSPAN1 | tetraspanin 1 | 0.00210143 | -1.751386724 | 30.65011028 | 32.11390046 | 15.70844866 | 20.42808683 |
| MSH5 | mutS homolog 5 (E. coli) | 0.00539968 | -1.752250962 | 65.21971718 | 81.94304008 | 42.27083978 | 41.17726463 |
| BANF1 | barrier to autointegration factor 1 | 0.00179836 | -1.75261799 | 92.76770781 | 115.6674109 | 56.39185694 | 61.94652002 |
| MCCC2 | methylcrotonoyl-CoA carboxylase 2 (beta) | 0.00556648 | -1.752622788 | 66.61773847 | 84.78744591 | 45.99782605 | 39.97672601 |
| CTDSPL2 | CTD (carboxy-terminal domain. RNA polymerase II. polypeptide A) small phosphatase like 2 | 0.00040634 | -1.752685929 | 123.7379906 | 119.9934909 | 71.1704554 | 67.91298227 |
| PRPF19 | PRP19/PSO4 pre-mRNA processing factor 19 homolog (S. cerevisiae) | 0.00190287 | -1.75287859 | 397.7523703 | 473.8686577 | 271.2030621 | 226.1895307 |
| RNF219 | ring finger protein 219 | 0.00469665 | -1.753448588 | 226.7518201 | 166.7562165 | 116.1735143 | 105.8618829 |
| RFC2 | replication factor C (activator 1) 2. 40kDa | 0.00696463 | -1.753587352 | 117.3281704 | 113.4830785 | 65.19829982 | 66.41135287 |
| RNPS1 | RNA binding protein S1. serine-rich domain | 0.00115607 | -1.754279508 | 1060.632271 | 1197.194783 | 664.4170264 | 620.9995974 |
| XBP1 | X-box binding protein 1 | 0.00032065 | -1.754420237 | 3510.489372 | 3408.35387 | 1943.809877 | 1999.821809 |
| GMPPA | GDP-mannose pyrophosphorylase A | 0.00154442 | -1.754621303 | 50.50689386 | 64.95849701 | 31.91257832 | 33.39319233 |
| ACBD3 | acyl-CoA binding domain containing 3 | 0.00045707 | -1.755858233 | 547.8854264 | 573.809078 | 315.0317145 | 323.6860553 |
| MIR25 | microRNA 25 | 0.00263845 | -1.755893358 | 45.78628401 | 39.94560405 | 23.90361378 | 24.81672998 |
| KAT5 | K(lysine) acetyltransferase 5 | 0.00313969 | -1.756241295 | 39.60835468 | 37.98722171 | 18.70771625 | 26.07565282 |
| KRR1 | KRR1. small subunit (SSU) processome component. homolog (yeast) | 0.00042931 | -1.757301277 | 57.55572154 | 61.14274019 | 34.16165315 | 33.35815891 |
| SCMH1 | sex comb on midleg homolog 1 (Drosophila) | 0.0131085 | -1.758360931 | 53.76432348 | 65.49926216 | 42.57939005 | 26.74946535 |
| BCL7A | B-cell CLL/lymphoma 7A | 0.00033786 | -1.758524853 | 233.3986359 | 246.2110936 | 134.0238701 | 138.6521969 |
| PPP3CB | protein phosphatase 3. catalytic subunit. beta isozyme | 0.0011259 | -1.759211526 | 201.2603804 | 191.2350814 | 100.2531237 | 124.0485858 |
| FAM129C | family with sequence similarity 129. member C | 0.0015348 | -1.763550985 | 96.30322115 | 91.10282418 | 59.21615103 | 47.63832018 |
| TBPL1 | TBP-like 1 | 0.00069871 | -1.76398036 | 1447.740261 | 1321.451492 | 811.4206398 | 757.7192769 |
| LOC648927 | similar to MYST histone acetyltransferase 2 | 0.00509561 | -1.764676401 | 117.6465644 | 171.6350875 | 83.57911174 | 77.58136524 |
| BSPH1 | binder of sperm protein homolog 1 | 0.00731573 | -1.765712846 | 25.32093598 | 38.23787458 | 19.1417007 | 16.22380901 |
| NFKB2 | nuclear factor of kappa light polypeptide gene enhancer in B-cells 2 (p49/p100) | 0.01942459 | -1.766527808 | 91.26018894 | 91.26018894 | 43.90506117 | 60.78650645 |
| PHF1 | PHD finger protein 1 | 0.00048546 | -1.766903836 | 58.84449412 | 52.44748926 | 30.65872161 | 32.24413027 |
| USP37 | ubiquitin specific peptidase 37 | 0.00298958 | -1.767222063 | 111.7742534 | 125.2903065 | 69.64660593 | 64.38386483 |
| MRPL21 | mitochondrial ribosomal protein L21 | 0.00280175 | -1.767481643 | 701.9558742 | 566.7200025 | 386.725318 | 329.2804335 |
| LOC723972 | hepatopoietin PCn127 | 0.00173305 | -1.767682517 | 361.6810404 | 390.0162633 | 236.4216512 | 190.9469303 |
| ASXL1 | additional sex combs like 1 (Drosophila) | 0.00162743 | -1.767918626 | 271.6946315 | 215.8198078 | 146.082862 | 128.4246961 |
| FAM122A | family with sequence similarity 122A | 0.00095685 | -1.768099677 | 63.93566655 | 70.94541495 | 34.74331332 | 41.76214606 |
| RAN | RAN. member RAS oncogene family | 0.00353773 | -1.769239983 | 614.8105951 | 544.8673689 | 277.1802311 | 386.0969754 |
| COPB1 | coatomer protein complex. subunit beta 1 | 0.00053124 | -1.769848459 | 1749.704307 | 1537.283183 | 897.3145865 | 956.9773203 |
| PPAPDC1B | phosphatidic acid phosphatase type 2 domain containing 1B | 0.01422316 | -1.769967709 | 47.90789173 | 57.65153399 | 23.27140079 | 37.88477519 |
| SNX5 | sorting nexin 5 | 0.00046523 | -1.770136998 | 1157.651891 | 1246.255201 | 678.9642102 | 678.147755 |
| ZNF655 | zinc finger protein 655 | 0.00177634 | -1.770174191 | 75.53215011 | 96.91588666 | 51.48634753 | 45.37354675 |
| GEMIN7 | gem (nuclear organelle) associated protein 7 | 0.00944053 | -1.770242661 | 31.79372434 | 49.63514125 | 24.42453054 | 20.61763451 |
| AHCYL1 | adenosylhomocysteinase-like 1 | 0.00056956 | -1.77109897 | 949.5083561 | 959.115945 | 539.2723244 | 538.3641206 |
| BNIP1 | BCL2/adenovirus E1B 19kDa interacting protein 1 | 0.00201222 | -1.771267218 | 111.6461089 | 121.5497986 | 68.74893723 | 62.91637269 |
| LOC390705 | protein phosphatase 2. regulatory subunit B''. beta pseudogene | 0.01692194 | -1.77428074 | 42.14881006 | 37.13751483 | 17.90435675 | 27.7712071 |
| TMEM200B | transmembrane protein 200B | 0.03642431 | -1.775331586 | 15.76312389 | 32.43528635 | 13.20254814 | 12.28692778 |
| TLK2 | tousled-like kinase 2 | 0.00109511 | -1.775474914 | 281.5712022 | 305.1801392 | 148.7224962 | 183.2899931 |
| CCDC47 | coiled-coil domain containing 47 | 0.00114208 | -1.776016552 | 130.6672201 | 145.7327199 | 75.2445862 | 80.23326133 |
| SKA3 | spindle and kinetochore associated complex subunit 3 | 0.00048377 | -1.776964551 | 111.3580337 | 123.0235413 | 63.96896358 | 67.82392759 |
| IL32 | interleukin 32 | 0.00146629 | -1.777526228 | 62.24790632 | 53.32418362 | 34.71341975 | 30.26352618 |
| IVNS1ABP | influenza virus NS1A binding protein | 0.00147305 | -1.778207165 | 246.433409 | 240.3696735 | 140.037192 | 133.7738274 |
| USP1 | ubiquitin specific peptidase 1 | 0.00142501 | -1.778462295 | 470.1512649 | 506.6492013 | 251.7895133 | 299.1010989 |
| SIP1 | survival of motor neuron protein interacting protein 1 | 0.00151525 | -1.778941509 | 163.5634529 | 163.649551 | 88.55150455 | 95.51724906 |
| PMF1 | polyamine-modulated factor 1 | 0.00050189 | -1.77941007 | 148.1306557 | 158.802425 | 84.14973406 | 88.28709754 |
| PKD1 | polycystic kidney disease 1 (autosomal dominant) | 0.00096334 | -1.78090793 | 60.20804099 | 58.71623328 | 36.743227 | 30.33559048 |
| NACC2 | NACC family member 2. BEN and BTB (POZ) domain containing | 0.00333934 | -1.781039499 | 43.532834 | 38.37264199 | 26.32770713 | 20.00223392 |
| SMC3 | structural maintenance of chromosomes 3 | 0.00037266 | -1.781603276 | 712.7819264 | 667.2987742 | 402.1615246 | 372.6099795 |
| MIR586 | microRNA 586 | 0.01674062 | -1.781901165 | 52.73295369 | 67.60144183 | 38.14930881 | 29.42958643 |
| CNOT2 | CCR4-NOT transcription complex. subunit 2 | 0.00039821 | -1.783004466 | 1012.186434 | 1067.601918 | 571.2798051 | 594.998847 |
| BCLAF1 | BCL2-associated transcription factor 1 | 0.00033209 | -1.78354999 | 177.0494326 | 171.3695151 | 100.9273486 | 94.503669 |
| RALGPS2 | Ral GEF with PH domain and SH3 binding motif 2 | 0.00290407 | -1.783908969 | 90.71894775 | 91.94309963 | 43.94876248 | 59.63828093 |
| ELMO1 | engulfment and cell motility 1 | 0.00044741 | -1.784305955 | 90.26617172 | 98.66072054 | 50.83473553 | 55.02627173 |
| KIF5B | kinesin family member 5B | 0.00126315 | -1.784869301 | 250.113476 | 274.4306913 | 132.1464921 | 163.0426752 |
| ZCCHC8 | zinc finger. CCHC domain containing 8 | 0.00042749 | -1.785522912 | 399.4518155 | 431.5642427 | 229.2774679 | 235.8400844 |
| KLF12 | Kruppel-like factor 12 | 0.00059704 | -1.786715882 | 89.00494686 | 94.56262406 | 50.43659832 | 52.27293393 |
| AARS | alanyl-tRNA synthetase | 0.00042831 | -1.788363438 | 4968.281548 | 5497.555445 | 2905.958721 | 2938.832347 |
| SKA2 | spindle and kinetochore associated complex subunit 2 | 0.00123578 | -1.788510424 | 802.4934428 | 806.802226 | 488.518586 | 414.3282424 |
| DBNL | drebrin-like | 0.00349667 | -1.788633505 | 311.7612217 | 403.7069533 | 182.4292789 | 215.6507929 |
| ERN1 | endoplasmic reticulum to nucleus signaling 1 | 0.01048283 | -1.789251554 | 32.1769485 | 50.30841605 | 23.92051256 | 21.13840488 |
| DHRS7B | dehydrogenase/reductase (SDR family) member 7B | 0.00067405 | -1.790548718 | 96.4650377 | 88.71364383 | 54.46099788 | 49.01200598 |
| TXNDC11 | thioredoxin domain containing 11 | 0.00041113 | -1.792844003 | 293.3045372 | 322.3808692 | 178.8761986 | 164.4562869 |
| WDHD1 | WD repeat and HMG-box DNA binding protein 1 | 0.00059811 | -1.796578246 | 41.78843112 | 37.10738784 | 23.06841685 | 20.82602504 |
| RWDD2B | RWD domain containing 2B | 0.00094249 | -1.797218778 | 68.86415416 | 84.30939378 | 44.56905825 | 40.3305209 |
| FIGNL1 | fidgetin-like 1 | 0.00521097 | -1.797580586 | 124.0485858 | 123.7088848 | 66.87031931 | 71.02031172 |
| INO80C | INO80 complex subunit C | 0.00152191 | -1.798111912 | 233.1658946 | 288.1052409 | 146.8191984 | 141.5141606 |
| KIAA0020 | KIAA0020 | 0.00035772 | -1.798629518 | 683.9794667 | 637.697037 | 361.47159 | 372.9922951 |
| AP1AR | adaptor-related protein complex 1 associated regulatory protein | 0.02659001 | -1.801318919 | 44.50306484 | 22.87724458 | 17.54509544 | 17.88367136 |
| DIDO1 | death inducer-obliterator 1 | 0.00122819 | -1.801786184 | 35.53462472 | 40.08885901 | 22.4148617 | 19.57640138 |
| PLS1 | plastin 1 | 0.00212664 | -1.802122044 | 70.97063826 | 53.01934863 | 32.51922212 | 35.62907538 |
| LOC642513 | similar to Potassium channel tetramerisation domain containing 9 | 0.00590154 | -1.802159439 | 47.82224648 | 33.19591454 | 22.15325337 | 22.06432649 |
| PHGDH | phosphoglycerate dehydrogenase | 0.00029809 | -1.802253032 | 8531.81192 | 8760.716429 | 4968.281548 | 4631.729283 |
| DYRK1A | dual-specificity tyrosine-(Y)-phosphorylation regulated kinase 1A | 0.00045406 | -1.807021833 | 327.6985283 | 364.4185057 | 197.4669062 | 185.2054627 |
| ZNF655 | zinc finger protein 655 | 0.00337857 | -1.808501764 | 64.12664038 | 70.05363968 | 36.77562658 | 37.34833431 |
| HSPD1 | heat shock 60kDa protein 1 (chaperonin) | 0.00643726 | -1.80976757 | 72.17346069 | 97.33444449 | 39.74306125 | 53.96810457 |
| KIAA0040 | KIAA0040 | 0.00106879 | -1.810375917 | 65.49926216 | 82.17729335 | 42.50172474 | 38.64062649 |
| KLHL4 | kelch-like 4 (Drosophila) | 0.0541921 | -1.812159363 | 34.02197237 | 26.05277198 | 17.13200769 | 15.75478173 |
| LOC728800 | similar to FLJ00402 protein | 0.00217466 | -1.812408596 | 26.14168079 | 34.03585594 | 16.77091024 | 16.15107088 |
| GRPEL2 | GrpE-like 2. mitochondrial (E. coli) | 0.00036028 | -1.813161036 | 435.3253095 | 433.0559311 | 231.8432688 | 247.3378957 |
| FAM111A | family with sequence similarity 111. member A | 0.00139263 | -1.813302491 | 170.7494428 | 168.9941532 | 105.3752286 | 83.28215006 |
| LOC643668 | proteasome (prosome. macropain) 26S subunit. ATPase. 1 pseudogene | 0.00027295 | -1.813333711 | 1620.993303 | 1573.956022 | 915.5352288 | 847.5069782 |
| DENND1A | DENN/MADD domain containing 1A | 0.00052322 | -1.813392879 | 41.08595045 | 36.10052831 | 20.10145951 | 22.43858635 |
| ZFYVE16 | zinc finger. FYVE domain containing 16 | 0.00820321 | -1.813566673 | 62.18492919 | 40.6705892 | 25.28559786 | 30.41062381 |
| KIF23 | kinesin family member 23 | 0.00097656 | -1.815087408 | 234.1456842 | 284.5266655 | 138.7576619 | 145.7327199 |
| VEGFA | vascular endothelial growth factor A | 0.00041494 | -1.815646499 | 130.4826314 | 139.2566619 | 74.29394538 | 74.19124718 |
| ZWINT | ZW10 interactor | 0.00155927 | -1.816105579 | 158.8413613 | 164.5847829 | 78.42122415 | 101.07354 |
| PSMD7 | proteasome (prosome. macropain) 26S subunit. non-ATPase. 7 | 0.00025514 | -1.816109402 | 1886.253153 | 1943.809877 | 1025.309056 | 1084.213864 |
| RFC1 | replication factor C (activator 1) 1. 145kDa | 0.0058727 | -1.816425646 | 186.277501 | 231.2367452 | 100.0528109 | 130.4826314 |
| H1FX | H1 histone family. member X | 0.01143176 | -1.81642883 | 438.8526155 | 431.4220774 | 262.80835 | 218.3459418 |
| C10orf125 | chromosome 10 open reading frame 125 | 0.00107759 | -1.816781637 | 91.22171538 | 82.04554226 | 49.58011962 | 45.73410567 |
| C4orf46 | chromosome 4 open reading frame 46 | 0.00025591 | -1.817749593 | 146.2697783 | 136.361665 | 77.35036845 | 78.0398453 |
| C6orf211 | chromosome 6 open reading frame 211 | 0.00746637 | -1.819704535 | 183.1097051 | 131.7735862 | 77.24340823 | 94.33580198 |
| SH3BP5 | SH3-domain binding protein 5 (BTK-associated) | 0.04524696 | -1.820075612 | 17.75103629 | 39.8254998 | 15.16362143 | 14.07353355 |
| C4orf32 | chromosome 4 open reading frame 32 | 0.00268317 | -1.820987476 | 321.4007679 | 302.8996281 | 182.1772589 | 161.1526937 |
| C1orf135 | chromosome 1 open reading frame 135 | 0.00032245 | -1.821682944 | 97.06302955 | 99.94622593 | 51.35647333 | 56.92191008 |
| SF1 | splicing factor 1 | 0.05944736 | -1.822684525 | 104.4767522 | 108.6365625 | 37.50082526 | 91.10282418 |
| PRR13 | proline rich 13 | 0.00232035 | -1.824315538 | 50.81021137 | 64.12664038 | 30.69794882 | 31.89188703 |
| MCM5 | minichromosome maintenance complex component 5 | 0.00029393 | -1.82460226 | 338.9791937 | 375.933703 | 196.405732 | 194.8918731 |
| TAF5 | TAF5 RNA polymerase II. TATA box binding protein (TBP)-associated factor. 100kDa | 0.00088996 | -1.825386508 | 144.111229 | 158.3888193 | 79.33888989 | 86.34288415 |
| RASGRP3 | RAS guanyl releasing protein 3 (calcium and DAG-regulated) | 0.00032877 | -1.825634355 | 1700.458473 | 1613.577236 | 944.4712493 | 871.6446065 |
| RPS7 | ribosomal protein S7 | 0.00095108 | -1.828876107 | 519.4753654 | 457.7968924 | 268.933539 | 264.3773579 |
| HAX1 | HCLS1 associated protein X-1 | 0.0003664 | -1.829698667 | 462.9814549 | 479.5255873 | 259.7621131 | 255.2938949 |
| USMG5 | up-regulated during skeletal muscle growth 5 homolog (mouse) | 0.00951219 | -1.8298873 | 44.95058544 | 49.03092804 | 22.07289997 | 29.81929591 |
| TP63 | tumor protein p63 | 0.00021792 | -1.830337897 | 968.7756474 | 962.6173472 | 519.8356702 | 535.4861919 |
| ARHGAP9 | Rho GTPase activating protein 9 | 0.00495995 | -1.830593136 | 88.49752189 | 74.76659301 | 36.71147472 | 53.78401395 |
| MT1E | metallothionein 1E | 0.00586147 | -1.830801281 | 49.99429249 | 59.31745774 | 24.37200497 | 36.3018939 |
| LOC728734 | similar to NPIP-like protein ENSP00000283050 | 0.00031398 | -1.831993547 | 959.7617967 | 886.634228 | 526.2282801 | 481.821182 |
| ERH | enhancer of rudimentary homolog (Drosophila) | 0.00303045 | -1.833243753 | 1459.889145 | 1909.048803 | 903.0617237 | 918.2882925 |
| LHFP | lipoma HMGIC fusion partner | 0.00020987 | -1.834448756 | 657.1187575 | 649.2756266 | 356.0028076 | 356.1298463 |
| LOC100133076 | similar to hCG1984118 | 0.03795962 | -1.83447553 | 33.75081525 | 46.95233208 | 29.6322343 | 15.89107183 |
| ASS1 | argininosuccinate synthase 1 | 0.00025242 | -1.836376681 | 2822.632561 | 2663.012096 | 1453.985307 | 1533.006265 |
| GFM2 | G elongation factor. mitochondrial 2 | 0.00049329 | -1.838529501 | 133.633579 | 151.2496982 | 72.84247474 | 82.08885894 |
| MID1IP1 | MID1 interacting protein 1 (gastrulation specific G12 homolog (zebrafish)) | 0.00059751 | -1.838848777 | 662.5216732 | 626.3705015 | 339.572776 | 361.4152645 |
| PYCR1 | pyrroline-5-carboxylate reductase 1 | 0.00107646 | -1.839109408 | 487.3005024 | 527.0318768 | 299.4520029 | 253.5662152 |
| MOBKL3 | MOB1. Mps One Binder kinase activator-like 3 (yeast) | 0.00029352 | -1.839909179 | 241.1078647 | 265.8438829 | 141.7296288 | 133.5932842 |
| FCER2 | Fc fragment of IgE. low affinity II. receptor for (CD23) | 0.00028597 | -1.840297975 | 210.6522022 | 223.7010628 | 123.3356728 | 112.8155714 |
| DLD | dihydrolipoamide dehydrogenase | 0.00042313 | -1.840408005 | 1083.240312 | 1059.919936 | 624.9480001 | 542.4075983 |
| EPS15 | epidermal growth factor receptor pathway substrate 15 | 0.00112109 | -1.840429656 | 246.6547269 | 204.5332957 | 110.9319211 | 134.2636593 |
| UNG | uracil-DNA glycosylase | 0.002052 | -1.841019518 | 123.0999624 | 154.0466418 | 84.52345448 | 66.19361984 |
| FAM18B1 | family with sequence similarity 18. member B1 | 0.0007621 | -1.841596764 | 134.49208 | 107.8997896 | 64.00922703 | 66.84757224 |
| ADAM10 | ADAM metallopeptidase domain 10 | 0.00231887 | -1.841993978 | 39.19616384 | 41.46239772 | 22.93047445 | 20.88856109 |
| PCNA | proliferating cell nuclear antigen | 0.00025626 | -1.847717637 | 1054.952158 | 1054.196397 | 600.2349195 | 542.702505 |
| THOC6 | THO complex 6 homolog (Drosophila) | 0.00050705 | -1.848696418 | 145.3806563 | 172.3383026 | 83.05934825 | 88.26097442 |
| PHF1 | PHD finger protein 1 | 0.00160999 | -1.84945034 | 87.97466729 | 74.17547584 | 40.4168343 | 47.20307936 |
| NUP88 | nucleoporin 88kDa | 0.00043289 | -1.851473383 | 819.4018557 | 738.3616152 | 447.7537231 | 394.1775654 |
| CDK10 | cyclin-dependent kinase 10 | 0.017687 | -1.852230597 | 87.39464805 | 83.34168955 | 51.49498871 | 41.22798058 |
| FNDC3B | fibronectin type III domain containing 3B | 0.00056377 | -1.853116267 | 379.0311887 | 341.5422224 | 179.7836971 | 209.6832329 |
| SHOC2 | soc-2 suppressor of clear homolog (C. elegans) | 0.0004249 | -1.854158893 | 698.1301171 | 765.6377261 | 401.9460112 | 386.8103431 |
| MRPL54 | mitochondrial ribosomal protein L54 | 0.00050174 | -1.855386647 | 846.7861258 | 842.7943911 | 499.413485 | 415.1129215 |
| ACTR10 | actin-related protein 10 homolog (S. cerevisiae) | 0.00180203 | -1.855696153 | 587.7940718 | 642.2056818 | 320.852213 | 341.6493285 |
| CPSF6 | cleavage and polyadenylation specific factor 6. 68kDa | 0.00694789 | -1.856078434 | 55.13811441 | 70.52810301 | 27.93284596 | 40.41162155 |
| LBR | lamin B receptor | 0.00047174 | -1.8569641 | 254.5547128 | 260.5862426 | 126.560394 | 151.9944717 |
| IFI44L | interferon-induced protein 44-like | 0.01877237 | -1.858171208 | 46.85404475 | 78.76825712 | 32.37923716 | 33.01111992 |
| IFI44L | interferon-induced protein 44-like | 0.07752658 | -1.864334603 | 214.4448688 | 404.4736379 | 158.3142553 | 157.6297318 |
| EXO1 | exonuclease 1 | 0.00024356 | -1.865811793 | 238.0239346 | 227.0757018 | 130.549583 | 118.9269111 |
| YWHAE | tyrosine 3-monooxygenase/tryptophan 5-monooxygenase activation protein. epsilon polypeptide | 0.00024337 | -1.870867427 | 369.3662214 | 338.8676947 | 191.1102808 | 187.1187324 |
| BLM | Bloom syndrome. RecQ helicase-like | 0.00045238 | -1.873517459 | 124.1568241 | 142.9507839 | 75.6669475 | 66.8244561 |
| MTHFD1L | methylenetetrahydrofolate dehydrogenase (NADP+ dependent) 1-like | 0.000374 | -1.874213047 | 687.5590099 | 799.3631942 | 402.4188119 | 388.8104573 |
| TSEN15 | tRNA splicing endonuclease 15 homolog (S. cerevisiae) | 0.00194093 | -1.875044841 | 75.04663129 | 82.13167487 | 47.76899693 | 36.70053906 |
| ACTA2 | actin. alpha 2. smooth muscle. aorta | 0.00097254 | -1.879257073 | 112.248467 | 110.8343973 | 68.01722574 | 51.79211922 |
| PYCR1 | pyrroline-5-carboxylate reductase 1 | 0.0106176 | -1.881021095 | 27.79998081 | 40.6850123 | 15.61994742 | 20.4650141 |
| AFTPH | aftiphilin | 0.00447833 | -1.881351537 | 71.19541143 | 64.87063543 | 29.72767753 | 43.89340472 |
| ASPHD2 | aspartate beta-hydroxylase domain containing 2 | 0.00020524 | -1.882831104 | 262.302779 | 285.2293647 | 146.1968289 | 144.3565338 |
| HAX1 | HCLS1 associated protein X-1 | 0.00034503 | -1.885516015 | 375.039019 | 405.71884 | 202.7922755 | 211.0521447 |
| BCL7A | B-cell CLL/lymphoma 7A | 0.00067139 | -1.886190731 | 107.3587556 | 128.6221248 | 65.1238988 | 59.59935272 |
| SRSF7 | serine/arginine-rich splicing factor 7 | 0.00015386 | -1.886867152 | 411.6482148 | 421.9864603 | 218.6857465 | 223.1113312 |
| EIF3CL | eukaryotic translation initiation factor 3. subunit C-like | 0.00076191 | -1.88891795 | 161.6051981 | 185.4153 | 92.02235494 | 91.26018894 |
| YIPF1 | Yip1 domain family. member 1 | 0.00046636 | -1.888956472 | 1196.592962 | 1332.839322 | 722.6520347 | 618.5162446 |
| DNAJC10 | DnaJ (Hsp40) homolog. subfamily C. member 10 | 0.00117509 | -1.889276385 | 100.4112951 | 97.21926964 | 45.12048197 | 60.61361698 |
| RAD51AP1 | RAD51 associated protein 1 | 0.00029874 | -1.893062338 | 521.2480356 | 501.4941512 | 290.1034425 | 251.436076 |
| UVRAG | UV radiation resistance associated gene | 0.01109158 | -1.897798189 | 56.5672338 | 45.96783454 | 27.78127888 | 25.98763134 |
| CCT6P1 | chaperonin containing TCP1. subunit 6 (zeta) pseudogene 1 | 0.01640519 | -1.900323168 | 51.77492917 | 28.74337132 | 23.22139265 | 17.74655382 |
| HMGB1 | high-mobility group box 1 | 0.00051284 | -1.900605589 | 271.2030621 | 252.1714174 | 138.7028527 | 136.496354 |
| RIOK1 | RIO kinase 1 (yeast) | 0.00313185 | -1.903027004 | 207.8302503 | 229.5353486 | 93.9847421 | 140.1558021 |
| RELL1 | RELT-like 1 | 0.00163637 | -1.903473703 | 94.1515 | 82.93242346 | 50.15748838 | 42.96571629 |
| LOC100132330 | similar to mal. T-cell differentiation protein-like | 0.00024253 | -1.904067977 | 91.94309963 | 102.1312643 | 53.04234446 | 48.83039521 |
| TCEA1 | transcription elongation factor A (SII). 1 | 0.00045189 | -1.904333077 | 2905.958721 | 2499.55837 | 1408.362722 | 1422.17284 |
| TCEA1 | transcription elongation factor A (SII). 1 | 0.00158062 | -1.905613763 | 276.9232344 | 236.1771208 | 130.6061026 | 137.8999117 |
| PEMT | phosphatidylethanolamine N-methyltransferase | 0.03402986 | -1.908355822 | 31.3318275 | 40.24481668 | 20.77962886 | 16.66246251 |
| CLGN | calmegin | 0.00035623 | -1.910343501 | 124.5090081 | 105.7234347 | 60.1267321 | 59.99036531 |
| SLC25A4 | solute carrier family 25 (mitochondrial carrier; adenine nucleotide translocator). member 4 | 0.00056899 | -1.910650509 | 1648.88418 | 1720.713245 | 871.1228952 | 892.1886953 |
| LOC729991-MEF2B | LOC729991-MEF2B readthrough transcript | 0.00546314 | -1.911865062 | 365.2298892 | 388.52012 | 156.7965708 | 247.5875441 |
| PICALM | phosphatidylinositol binding clathrin assembly protein | 0.00378559 | -1.915859761 | 58.90257219 | 85.63127653 | 38.65880175 | 35.54600991 |
| SGOL1 | shugoshin-like 1 (S. pombe) | 0.00018445 | -1.918127925 | 151.1012312 | 154.5870102 | 77.52067628 | 81.89706009 |
| MPDU1 | mannose-P-dolichol utilization defect 1 | 0.00032724 | -1.918708154 | 77.95817283 | 75.46485215 | 43.24729188 | 36.95129107 |
| TIMM44 | translocase of inner mitochondrial membrane 44 homolog (yeast) | 0.00062753 | -1.91961262 | 346.2249049 | 274.9461028 | 164.2660867 | 157.2645254 |
| PYHIN1 | pyrin and HIN domain family. member 1 | 0.00140979 | -1.923166419 | 145.2832514 | 126.6846027 | 81.96512713 | 60.71227872 |
| WNT5B | wingless-type MMTV integration site family. member 5B | 0.00314321 | -1.923556658 | 113.8796326 | 129.9521739 | 65.28048856 | 61.26835842 |
| GFPT1 | glutamine--fructose-6-phosphate transaminase 1 | 0.00084841 | -1.924059278 | 285.302715 | 289.8797078 | 131.0609735 | 170.4564479 |
| MCM4 | minichromosome maintenance complex component 4 | 0.00021894 | -1.924688587 | 524.6129496 | 506.463798 | 260.7910515 | 275.0260215 |
| IFRD1 | interferon-related developmental regulator 1 | 0.00017378 | -1.925163655 | 818.3118813 | 798.9598204 | 440.243613 | 400.6963846 |
| ZNF280D | zinc finger protein 280D | 0.00160846 | -1.929050916 | 53.24723564 | 52.03868008 | 23.30866567 | 31.94615134 |
| NCRNA00120 | non-protein coding RNA 120 | 0.02873296 | -1.929123398 | 50.28855472 | 38.41265636 | 20.60949318 | 25.18582412 |
| MOBKL3 | MOB1. Mps One Binder kinase activator-like 3 (yeast) | 0.00037377 | -1.931886954 | 400.2363942 | 337.7483336 | 199.78232 | 181.2964586 |
| TP53 | tumor protein p53 | 0.00754256 | -1.932533139 | 96.28270295 | 117.1602853 | 63.66078644 | 47.44634857 |
| EIF4EBP1 | eukaryotic translation initiation factor 4E binding protein 1 | 0.000974 | -1.932866994 | 128.8373377 | 113.6194492 | 71.28614317 | 54.96488995 |
| MX2 | myxovirus (influenza virus) resistance 2 (mouse) | 0.00017204 | -1.934504823 | 223.8465187 | 219.6012507 | 109.0069993 | 120.5011344 |
| DCK | deoxycytidine kinase | 0.00016673 | -1.935730637 | 1059.919936 | 1078.770928 | 526.4146968 | 579.6748281 |
| THRSP | thyroid hormone responsive | 0.00012897 | -1.935937374 | 298.9026245 | 305.4831427 | 156.595264 | 155.5807091 |
| CDC25A | cell division cycle 25 homolog A (S. pombe) | 0.00352936 | -1.937988816 | 49.8246136 | 62.67005052 | 24.39342286 | 34.08228524 |
| SBDS | Shwachman-Bodian-Diamond syndrome | 0.00172316 | -1.938140504 | 574.6268675 | 512.4928339 | 236.5902388 | 331.3648111 |
| PAG1 | phosphoprotein associated with glycosphingolipid microdomains 1 | 0.00043742 | -1.939394371 | 620.4448426 | 499.299647 | 288.9415857 | 285.0507082 |
| AP1AR | adaptor-related protein complex 1 associated regulatory protein | 0.00032362 | -1.939675745 | 57.61197296 | 50.81021137 | 26.23834484 | 29.65302774 |
| CUL4B | cullin 4B | 0.00099251 | -1.940075482 | 64.99673779 | 57.56516424 | 36.09706059 | 27.53862263 |
| PSPH | phosphoserine phosphatase | 0.00049557 | -1.940301877 | 957.4332317 | 1063.548001 | 469.1795465 | 576.4848713 |
| SBDSP1 | Shwachman-Bodian-Diamond syndrome pseudogene 1 | 0.00032091 | -1.941712658 | 260.6751979 | 278.7656031 | 127.2662599 | 151.4452624 |
| HAT1 | histone acetyltransferase 1 | 0.00169053 | -1.944762809 | 203.8850043 | 184.704954 | 85.13530095 | 116.9555597 |
| SLC43A1 | solute carrier family 43. member 1 | 0.00248364 | -1.945710072 | 228.5675411 | 180.3205184 | 96.01381077 | 113.3886911 |
| HVCN1 | hydrogen voltage-gated channel 1 | 0.01489317 | -1.946358079 | 60.33222909 | 72.03053577 | 34.62549177 | 33.13024 |
| CBX4 | chromobox homolog 4 | 0.00047191 | -1.946703696 | 932.7232861 | 934.5427285 | 483.237481 | 475.9832426 |
| SYTL1 | synaptotagmin-like 1 | 0.00025676 | -1.94697533 | 265.108716 | 231.8432688 | 131.8393452 | 122.9851183 |
| SP2 | Sp2 transcription factor | 0.00015745 | -1.947567186 | 154.3915301 | 148.9645178 | 81.55662224 | 74.3467825 |
| LOC100130503 | hypothetical LOC100130503 | 0.005593 | -1.948270756 | 22.60332976 | 33.79556449 | 13.00092135 | 15.47958708 |
| RAD23A | RAD23 homolog A (S. cerevisiae) | 0.00050816 | -1.950111027 | 1006.507198 | 912.9919597 | 547.0562431 | 441.7058827 |
| PATE3 | prostate and testis expressed 3 | 0.00129304 | -1.950519522 | 174.3730026 | 140.037192 | 81.8652467 | 78.40115523 |
| SEL1L | sel-1 suppressor of lin-12-like (C. elegans) | 0.00866388 | -1.950609277 | 63.33985605 | 39.67973654 | 27.78863475 | 23.77048653 |
| GAB2 | GRB2-associated binding protein 2 | 0.00137549 | -1.952463975 | 62.22402472 | 68.4112106 | 34.2977923 | 32.55765741 |
| GARS | glycyl-tRNA synthetase | 0.00011999 | -1.953530885 | 7853.387026 | 7998.21873 | 4015.548946 | 4098.876093 |
| TSEN15 | tRNA splicing endonuclease 15 homolog (S. cerevisiae) | 0.00039877 | -1.953899994 | 224.9121694 | 212.34686 | 105.0079001 | 119.1330227 |
| NTF3 | neurotrophin 3 | 0.00071772 | -1.953962611 | 59.37965396 | 52.43219288 | 30.51628376 | 26.72215882 |
| XPOT | exportin. tRNA (nuclear export receptor for tRNAs) | 0.00096254 | -1.956417185 | 373.8714879 | 446.3717922 | 184.2889638 | 236.5902388 |
| ZP3 | zona pellucida glycoprotein 3 (sperm receptor) | 0.00041713 | -1.956487605 | 44.76552392 | 51.51800229 | 24.9672395 | 24.13114653 |
| FBXO5 | F-box protein 5 | 0.00010636 | -1.960360105 | 549.6773677 | 556.2464403 | 282.4865193 | 281.6473449 |
| PLTP | phospholipid transfer protein | 0.00069804 | -1.962164854 | 88.05708801 | 68.93008252 | 42.17358307 | 37.38192249 |
| OXNAD1 | oxidoreductase NAD-binding domain containing 1 | 0.00039727 | -1.963457924 | 157.0841654 | 156.2368499 | 78.61782299 | 80.97513324 |
| GOT1 | glutamic-oxaloacetic transaminase 1. soluble (aspartate aminotransferase 1) | 0.00010716 | -1.965660721 | 1468.131603 | 1442.829517 | 731.6634311 | 749.2927621 |
| FBXO22 | F-box protein 22 | 0.00051954 | -1.966845401 | 188.8103704 | 154.6379889 | 82.59652911 | 91.37760907 |
| EZH2 | enhancer of zeste homolog 2 (Drosophila) | 0.00458124 | -1.967014928 | 206.7940977 | 162.0201035 | 98.45948709 | 87.94965975 |
| EIF1 | eukaryotic translation initiation factor 1 | 0.00016285 | -1.967194415 | 8196.761383 | 8531.81192 | 4054.371888 | 4457.23383 |
| LOC730167 | similar to protein tyrosine phosphatase 4a1 | 0.00143636 | -1.96978686 | 97.85810894 | 93.7530145 | 44.0166032 | 53.71889409 |
| MLX | MAX-like protein X | 0.00651885 | -1.97121981 | 123.0351169 | 170.5524556 | 91.2328343 | 59.19237775 |
| SOX5 | SRY (sex determining region Y)-box 5 | 0.00048127 | -1.973593003 | 32.93107651 | 29.54726284 | 16.21933087 | 15.40192686 |
| LMNB1 | lamin B1 | 0.01325008 | -1.977719514 | 57.47766631 | 48.07030438 | 20.10756436 | 35.13072675 |
| SH2B3 | SH2B adaptor protein 3 | 0.0097114 | -1.981680916 | 505.852799 | 488.2079999 | 185.5190675 | 338.9791937 |
| VLDLR | very low density lipoprotein receptor | 0.00155803 | -1.984813893 | 51.8736302 | 49.53705805 | 22.54766937 | 28.92914593 |
| RTN3 | reticulon 3 | 0.00046388 | -1.985165838 | 1372.562361 | 1318.702247 | 610.2683685 | 752.6002974 |
| HSPA5 | heat shock 70kDa protein 5 (glucose-regulated protein. 78kDa) | 0.00012061 | -1.98844606 | 233.1056877 | 224.2814558 | 118.5735099 | 111.5143949 |
| MLF1IP | MLF1 interacting protein | 0.00043942 | -1.990202458 | 109.9565358 | 101.2425922 | 59.26224797 | 47.42547257 |
| CDC45 | cell division cycle 45 homolog (S. cerevisiae) | 0.0001491 | -1.992306386 | 506.906315 | 530.5658654 | 273.0646483 | 248.135713 |
| CEBPB | CCAAT/enhancer binding protein (C/EBP). beta | 0.00016001 | -1.992550586 | 3543.258039 | 3135.710501 | 1662.324213 | 1683.465803 |
| LOC729898 | similar to zinc finger and BTB domain containing 8 opposite strand | 0.00505801 | -1.999877381 | 68.50731664 | 45.44963482 | 27.95945311 | 27.84402057 |
| PTS | 6-pyruvoyltetrahydropterin synthase | 0.00053937 | -2.004714174 | 193.5449243 | 203.6020048 | 100.1096089 | 97.94519837 |
| UBE2E1 | ubiquitin-conjugating enzyme E2E 1 (UBC4/5 homolog. yeast) | 0.0001711 | -2.006728178 | 201.0479944 | 218.6342435 | 107.8671048 | 101.1933117 |
| POLD3 | polymerase (DNA-directed). delta 3. accessory subunit | 0.00033357 | -2.009141679 | 106.5534839 | 94.11468263 | 48.13442455 | 51.6116954 |
| NOMO3 | NODAL modulator 3 | 0.00117049 | -2.010487897 | 60.30987423 | 59.71146106 | 33.11912345 | 26.90074827 |
| ATXN2L | ataxin 2-like | 0.00031388 | -2.010601144 | 114.0711641 | 127.0689968 | 65.60492891 | 54.65465471 |
| YARS | tyrosyl-tRNA synthetase | 0.00018408 | -2.011305728 | 3044.638253 | 3487.421366 | 1584.145405 | 1656.871779 |
| CNOT7 | CCR4-NOT transcription complex. subunit 7 | 0.00030793 | -2.014573495 | 346.0477617 | 404.2007823 | 184.5877865 | 186.7084617 |
| USP47 | ubiquitin specific peptidase 47 | 0.00066332 | -2.018098554 | 68.21781615 | 57.35859508 | 27.61698405 | 34.78846979 |
| CHAF1A | chromatin assembly factor 1. subunit A (p150) | 9.35E-05 | -2.018123122 | 83.72254476 | 87.30464108 | 43.31957721 | 41.42859698 |
| ZNF16 | zinc finger protein 16 | 0.00188956 | -2.018545729 | 37.01789334 | 49.55721116 | 23.38144209 | 19.25617745 |
| EGR1 | early growth response 1 | 0.00023869 | -2.018550206 | 107.8387638 | 119.2086598 | 51.89015995 | 60.8021008 |
| MNS1 | meiosis-specific nuclear structural 1 | 0.00104516 | -2.020194446 | 273.0646483 | 256.6416471 | 110.7244943 | 155.0824172 |
| XRCC3 | X-ray repair complementing defective repair in Chinese hamster cells 3 | 0.00049424 | -2.024090658 | 150.7308731 | 135.4243132 | 73.7135993 | 67.59138075 |
| UBE2L3 | ubiquitin-conjugating enzyme E2L 3 | 0.00453719 | -2.027009278 | 34.35067919 | 40.81201514 | 15.88393313 | 21.48096782 |
| CSTF1 | cleavage stimulation factor. 3' pre-RNA. subunit 1. 50kDa | 0.00165735 | -2.051235226 | 86.84348182 | 91.72376713 | 37.18999376 | 50.90519117 |
| SARS | seryl-tRNA synthetase | 0.00011372 | -2.052812142 | 950.6255102 | 1043.641483 | 492.7733487 | 477.765924 |
| TNFRSF10B | tumor necrosis factor receptor superfamily. member 10b | 0.00360212 | -2.053194983 | 39.87426368 | 27.02178541 | 16.97115523 | 15.06034506 |
| C15orf42 | chromosome 15 open reading frame 42 | 0.00237508 | -2.056642364 | 93.78223647 | 108.287101 | 60.68090379 | 39.56650751 |
| GFPT1 | glutamine--fructose-6-phosphate transaminase 1 | 0.00186014 | -2.059100696 | 237.9891192 | 169.2198619 | 88.84694749 | 106.9081355 |
| BEST1 | bestrophin 1 | 0.00165656 | -2.05912115 | 65.44321219 | 62.07280735 | 28.34930993 | 33.79556449 |
| IARS | isoleucyl-tRNA synthetase | 0.00083141 | -2.063645341 | 1849.207271 | 1735.277483 | 933.9364239 | 806.802226 |
| STT3A | STT3. subunit of the oligosaccharyltransferase complex. homolog A (S. cerevisiae) | 0.00025976 | -2.069673165 | 1074.59619 | 1114.255968 | 492.4198475 | 567.6637719 |
| CDC25A | cell division cycle 25 homolog A (S. pombe) | 0.00032018 | -2.073316084 | 292.3976776 | 316.3939679 | 143.5713965 | 149.9005255 |
| PTPDC1 | protein tyrosine phosphatase domain containing 1 | 0.00312353 | -2.074012489 | 39.57700735 | 39.8032465 | 15.12842711 | 24.20722107 |
| EDEM1 | ER degradation enhancer. mannosidase alpha-like 1 | 0.00012473 | -2.07486368 | 1748.462716 | 1905.384051 | 846.296984 | 914.4015869 |
| E2F7 | E2F transcription factor 7 | 0.00052834 | -2.075297171 | 40.45897006 | 52.95567363 | 23.5513083 | 21.12281008 |
| NPIP | nuclear pore complex interacting protein | 0.00030964 | -2.082036681 | 586.4495194 | 636.7310461 | 329.0337711 | 261.7998736 |
| SMAP1 | small ArfGAP 1 | 0.00024496 | -2.087331484 | 312.1469997 | 273.9326952 | 141.1687336 | 139.0214037 |
| SBDSP1 | Shwachman-Bodian-Diamond syndrome pseudogene 1 | 0.00020641 | -2.092323412 | 496.5142854 | 551.9013482 | 235.9161325 | 265.3248541 |
| VANGL2 | vang-like 2 (van gogh. Drosophila) | 0.00058906 | -2.093690311 | 83.96917733 | 77.07910415 | 44.19341461 | 33.40981814 |
| CARS | cysteinyl-tRNA synthetase | 0.00012134 | -2.09389403 | 595.9219038 | 547.8854264 | 283.2111452 | 262.9416353 |
| C4orf32 | chromosome 4 open reading frame 32 | 0.00045515 | -2.094416693 | 54.51371152 | 50.15283014 | 24.97311488 | 24.95757017 |
| SLC3A2 | solute carrier family 3 (activators of dibasic and neutral amino acid transport). member 2 | 0.00011098 | -2.094831589 | 1365.420858 | 1371.389279 | 649.7450313 | 656.7288381 |
| FBXO11 | F-box protein 11 | 0.00070275 | -2.097858619 | 127.8396723 | 135.9347574 | 54.50741149 | 72.44162342 |
| RTN3 | reticulon 3 | 9.10E-05 | -2.101269398 | 303.8483456 | 316.5303688 | 145.4736601 | 149.7353778 |
| SPNS3 | spinster homolog 3 (Drosophila) | 9.11E-05 | -2.102519846 | 97.59967143 | 91.44436046 | 47.08620917 | 42.87768674 |
| CHEK1 | CHK1 checkpoint homolog (S. pombe) | 0.00011204 | -2.110851219 | 346.3110483 | 315.2561207 | 160.4919998 | 152.672648 |
| HSPA9 | heat shock 70kDa protein 9 (mortalin) | 8.48E-05 | -2.11504442 | 3495.101835 | 3190.336935 | 1577.759173 | 1579.853026 |
| SLC16A6 | solute carrier family 16. member 6 (monocarboxylic acid transporter 7) | 7.82E-05 | -2.118412432 | 110.4677619 | 120.8031229 | 53.30072821 | 55.7904085 |
| VIPR1 | vasoactive intestinal peptide receptor 1 | 0.00013298 | -2.126643943 | 42.64632612 | 41.50581276 | 21.19719623 | 18.46386258 |
| MZT1 | mitotic spindle organizing protein 1 | 0.00034217 | -2.127138408 | 232.9508816 | 224.441954 | 121.3392802 | 95.23037324 |
| TCEA1 | transcription elongation factor A (SII). 1 | 0.00022282 | -2.127724688 | 243.703681 | 270.9397144 | 120.4820653 | 121.0546534 |
| TMEM39A | transmembrane protein 39A | 0.00098901 | -2.12848954 | 121.0546534 | 109.975851 | 48.37848244 | 60.74122387 |
| WEE1 | WEE1 homolog (S. pombe) | 7.46E-05 | -2.134881472 | 350.7055511 | 382.3427806 | 174.7624344 | 168.34488 |
| PPP1R3F | protein phosphatase 1. regulatory (inhibitor) subunit 3F | 0.00037526 | -2.138804473 | 158.2587738 | 185.5612649 | 89.98261498 | 71.34348884 |
| DNAJB9 | DnaJ (Hsp40) homolog. subfamily B. member 9 | 6.61E-05 | -2.139694884 | 675.9979757 | 706.1399904 | 329.2804335 | 316.6405866 |
| SLC6A9 | solute carrier family 6 (neurotransmitter transporter. glycine). member 9 | 5.40E-05 | -2.146786523 | 404.0327414 | 410.0650076 | 187.1646666 | 192.0736041 |
| MCM4 | minichromosome maintenance complex component 4 | 0.00123313 | -2.146801169 | 164.5847829 | 146.4648181 | 64.31281549 | 81.32832479 |
| SLC25A40 | solute carrier family 25. member 40 | 7.72E-05 | -2.149274867 | 111.0876009 | 101.9401383 | 47.84708335 | 51.23555166 |
| MORN4 | MORN repeat containing 4 | 0.00051204 | -2.15305802 | 74.02729524 | 79.73326633 | 41.18458675 | 30.9161395 |
| DDX21 | DEAD (Asp-Glu-Ala-Asp) box polypeptide 21 | 0.00024686 | -2.155390846 | 2672.205779 | 2144.434862 | 1128.495201 | 1093.027209 |
| CLDND1 | claudin domain containing 1 | 0.00010796 | -2.158435404 | 1031.322162 | 956.5995487 | 448.9215833 | 471.7109136 |
| INHBE | inhibin. beta E | 5.74E-05 | -2.161099202 | 1340.56749 | 1278.619088 | 595.7955114 | 616.0035766 |
| TRUB1 | TruB pseudouridine (psi) synthase homolog 1 (E. coli) | 0.00492045 | -2.16311569 | 66.19361984 | 68.23623118 | 28.07452167 | 34.38422996 |
| PTP4A1 | protein tyrosine phosphatase type IVA. member 1 | 0.00233403 | -2.168930689 | 82.1370925 | 102.8085044 | 52.38800778 | 34.26455819 |
| OSCAR | osteoclast associated. immunoglobulin-like receptor | 0.00221363 | -2.170159088 | 44.10569182 | 39.65703064 | 20.31279429 | 18.28361287 |
| SLC1A5 | solute carrier family 1 (neutral amino acid transporter). member 5 | 0.00010888 | -2.176407371 | 539.2723244 | 476.8873054 | 243.556296 | 222.9174969 |
| SLC3A2 | solute carrier family 3 (activators of dibasic and neutral amino acid transport). member 2 | 0.00039619 | -2.176667842 | 205.0929813 | 243.1842238 | 114.3953919 | 92.02235494 |
| SEH1L | SEH1-like (S. cerevisiae) | 0.00141309 | -2.17709514 | 122.2410865 | 86.25431681 | 51.90593994 | 42.85743395 |
| CCT8P1 | chaperonin containing TCP1. subunit 8 (theta) pseudogene 1 | 0.00058865 | -2.181147989 | 58.06053511 | 58.67231619 | 24.52417696 | 29.1977617 |
| ALG14 | asparagine-linked glycosylation 14 homolog (S. cerevisiae) | 0.0006266 | -2.182070828 | 110.5875773 | 125.9279473 | 52.91196903 | 55.27603178 |
| ATF3 | activating transcription factor 3 | 0.00045692 | -2.18914898 | 244.792241 | 196.6703288 | 111.9569736 | 89.72938803 |
| ASNS | asparagine synthetase (glutamine-hydrolyzing) | 7.76E-05 | -2.189395248 | 5735.439052 | 5989.945156 | 2590.568874 | 2766.597816 |
| MTMR12 | myotubularin related protein 12 | 0.00251539 | -2.191090209 | 169.5131989 | 200.8366685 | 67.00898183 | 105.8261491 |
| C13orf18 | chromosome 13 open reading frame 18 | 0.00023434 | -2.192216802 | 860.6134613 | 697.8818077 | 331.0614422 | 377.4977828 |
| AMMECR1 | Alport syndrome. mental retardation. midface hypoplasia and elliptocytosis chromosomal region gene 1 | 0.00013224 | -2.193279473 | 889.3656656 | 785.8439889 | 400.9536627 | 362.3552529 |
| RGS16 | regulator of G-protein signaling 16 | 0.00038893 | -2.200148886 | 773.9056734 | 885.8177573 | 391.4539788 | 361.7825028 |
| C4orf46 | chromosome 4 open reading frame 46 | 0.00050819 | -2.201632709 | 120.8958224 | 105.3995121 | 53.59282651 | 49.05163572 |
| PKD2L1 | polycystic kidney disease 2-like 1 | 0.00405529 | -2.208223117 | 69.68435013 | 54.58877391 | 33.70365656 | 23.14598512 |
| TUBE1 | tubulin. epsilon 1 | 0.00015623 | -2.208289443 | 144.7959949 | 141.1256163 | 61.24401112 | 68.42058477 |
| HNRNPA3 | heterogeneous nuclear ribonucleoprotein A3 | 0.00354277 | -2.209646814 | 187.296498 | 324.175819 | 103.8677832 | 119.7246804 |
| SLC7A5 | solute carrier family 7 (cationic amino acid transporter. y+ system). member 5 | 5.77E-05 | -2.215680626 | 2872.102154 | 3166.10813 | 1327.365984 | 1395.470375 |
| MARS | methionyl-tRNA synthetase | 3.87E-05 | -2.217103631 | 1507.620208 | 1480.26951 | 676.4831802 | 671.1255215 |
| CARS | cysteinyl-tRNA synthetase | 0.00078386 | -2.234719194 | 157.966976 | 112.0825539 | 64.16345318 | 55.25488449 |
| TIFA | TRAF-interacting protein with forkhead-associated domain | 0.00015135 | -2.23721534 | 317.0375512 | 314.9043102 | 128.1438556 | 155.6596128 |
| ASNS | asparagine synthetase (glutamine-hydrolyzing) | 0.00013639 | -2.238745599 | 1284.861052 | 1417.855081 | 607.5485313 | 598.2704371 |
| DEPDC6 | DEP domain containing 6 | 0.0003499 | -2.238770261 | 1096.330714 | 925.440357 | 509.3161968 | 397.4508861 |
| TSC1 | tuberous sclerosis 1 | 6.85E-05 | -2.239111884 | 405.5388992 | 457.4296063 | 196.3397405 | 188.4503662 |
| KIF21B | kinesin family member 21B | 0.00074501 | -2.240093852 | 299.1010989 | 327.7630465 | 165.0185153 | 118.38942 |
| MTHFD2 | methylenetetrahydrofolate dehydrogenase (NADP+ dependent) 2. methenyltetrahydrofolate cyclohydrolase | 4.65E-05 | -2.242829967 | 2357.225606 | 2266.053624 | 1069.464343 | 992.9155745 |
| PKMYT1 | protein kinase. membrane associated tyrosine/threonine 1 | 0.00078315 | -2.244478796 | 145.6671086 | 133.1531982 | 70.20471049 | 54.84232212 |
| NPIP | nuclear pore complex interacting protein | 0.00105582 | -2.245925796 | 331.1579785 | 249.7929605 | 149.7989278 | 109.4752401 |
| VAPA | VAMP (vesicle-associated membrane protein)-associated protein A. 33kDa | 0.00033213 | -2.251153993 | 203.4546275 | 214.5203178 | 81.02939586 | 106.2876963 |
| MCM10 | minichromosome maintenance complex component 10 | 0.00118026 | -2.253200934 | 172.4311978 | 185.2322029 | 96.62690107 | 65.10801274 |
| MAP1LC3B | microtubule-associated protein 1 light chain 3 beta | 0.00036593 | -2.260949576 | 969.4213651 | 1138.528266 | 416.5258195 | 518.361482 |
| TRIB3 | tribbles homolog 3 (Drosophila) | 0.00010947 | -2.26397414 | 2527.775909 | 2509.712608 | 1173.652797 | 1054.581143 |
| HES4 | hairy and enhancer of split 4 (Drosophila) | 0.00746722 | -2.265068214 | 38.41265636 | 73.21981646 | 25.68194772 | 21.34580061 |
| GTPBP2 | GTP binding protein 2 | 0.00025042 | -2.267305257 | 45.65496254 | 38.73345386 | 17.63794498 | 19.50321651 |
| PSMC3IP | PSMC3 interacting protein | 0.00028784 | -2.271346875 | 72.24191224 | 75.31839667 | 37.61467747 | 28.03922657 |
| CEBPG | CCAAT/enhancer binding protein (C/EBP). gamma | 6.60E-05 | -2.273192605 | 1116.108063 | 1048.961596 | 477.4981972 | 474.4840617 |
| SCN4A | sodium channel. voltage-gated. type IV. alpha subunit | 7.45E-05 | -2.282598518 | 52.09493397 | 45.90487749 | 20.27578884 | 22.6369287 |
| CHRAC1 | chromatin accessibility complex 1 | 0.00015843 | -2.283258119 | 115.6674109 | 112.5038505 | 44.4691158 | 56.13191841 |
| PSAT1 | phosphoserine aminotransferase 1 | 4.09E-05 | -2.284489017 | 4311.994505 | 4133.717487 | 1893.210713 | 1804.022245 |
| SLC7A3 | solute carrier family 7 (cationic amino acid transporter. y+ system). member 3 | 0.00040108 | -2.287116684 | 40.86100798 | 45.89694462 | 17.69119805 | 20.26557651 |
| UHRF1 | ubiquitin-like with PHD and ring finger domains 1 | 6.32E-05 | -2.287438264 | 989.406474 | 869.6296463 | 417.8397339 | 393.5506053 |
| MNS1 | meiosis-specific nuclear structural 1 | 0.01244026 | -2.296425961 | 89.44644984 | 65.64926131 | 22.8809833 | 48.66462393 |
| ALG14 | asparagine-linked glycosylation 14 homolog (S. cerevisiae) | 0.00033145 | -2.30338464 | 60.55998517 | 54.52549986 | 28.27111645 | 22.01453535 |
| PLAGL2 | pleiomorphic adenoma gene-like 2 | 4.14E-05 | -2.307133517 | 881.0295931 | 916.4098532 | 380.8394664 | 398.283983 |
| CLK1 | CDC-like kinase 1 | 0.00018866 | -2.310113573 | 102.3605266 | 95.3805586 | 43.85308169 | 41.71820285 |
| ETV5 | ets variant 5 | 4.81E-05 | -2.313119946 | 63.66078644 | 71.77817197 | 28.59947516 | 29.8613902 |
| PKMYT1 | protein kinase. membrane associated tyrosine/threonine 1 | 0.00034783 | -2.323800511 | 55.23063248 | 45.68265318 | 24.69224721 | 18.92226122 |
| CLDND1 | claudin domain containing 1 | 0.00010992 | -2.326260988 | 1243.521307 | 999.5828811 | 472.9936946 | 485.6236341 |
| MCM7 | minichromosome maintenance complex component 7 | 0.00124826 | -2.327091582 | 119.1681472 | 117.8071991 | 55.03121462 | 47.10814131 |
| WARS | tryptophanyl-tRNA synthetase | 0.00012012 | -2.327880868 | 5138.085838 | 4947.096678 | 2295.177757 | 2043.684844 |
| SLC3A2 | solute carrier family 3 (activators of dibasic and neutral amino acid transport). member 2 | 0.0004186 | -2.340928816 | 284.5266655 | 235.2436845 | 115.0235757 | 106.1885444 |
| NCAPG2 | non-SMC condensin II complex. subunit G2 | 0.00324799 | -2.34307169 | 103.5237128 | 96.44493343 | 36.64562567 | 49.62790728 |
| SLC7A1 | solute carrier family 7 (cationic amino acid transporter. y+ system). member 1 | 3.19E-05 | -2.350528292 | 1755.357782 | 1662.91105 | 732.6255052 | 721.1434589 |
| RABL3 | RAB. member of RAS oncogene family-like 3 | 0.00016733 | -2.360735686 | 96.62690107 | 109.860635 | 42.87768674 | 44.4236337 |
| FBXO11 | F-box protein 11 | 0.00019476 | -2.360950628 | 70.07403064 | 64.56845488 | 32.61491952 | 24.88784945 |
| SLC25A4 | solute carrier family 25 (mitochondrial carrier; adenine nucleotide translocator). member 4 | 0.00018235 | -2.361261544 | 515.1269277 | 609.4909337 | 238.0958416 | 236.505641 |
| PAOX | polyamine oxidase (exo-N4-amino) | 7.37E-05 | -2.366040174 | 243.6039666 | 246.5728952 | 111.2838506 | 96.41693394 |
| HELLS | helicase. lymphoid-specific | 0.00017002 | -2.385302363 | 76.32374156 | 68.65516215 | 30.34577513 | 30.34923423 |
| HYOU1 | hypoxia up-regulated 1 | 0.00015457 | -2.411407522 | 330.1504779 | 304.7799633 | 138.3225424 | 125.1019725 |
| PCNA | proliferating cell nuclear antigen | 0.00085966 | -2.416047342 | 43.95241675 | 52.98230633 | 21.98970226 | 18.14192597 |
| OSCAR | osteoclast associated. immunoglobulin-like receptor | 0.00015258 | -2.419366203 | 52.15372413 | 54.39628373 | 25.2067287 | 19.22803259 |
| SLC7A3 | solute carrier family 7 (cationic amino acid transporter. y+ system). member 3 | 0.00023009 | -2.420329573 | 213.273618 | 270.8766796 | 100.5302441 | 98.09864345 |
| AMMECR1 | Alport syndrome. mental retardation. midface hypoplasia and elliptocytosis chromosomal region gene 1 | 2.65E-05 | -2.424416741 | 379.6996931 | 397.0348416 | 154.5870102 | 165.9133451 |
| MATR3 | matrin 3 | 0.000259 | -2.425559891 | 83.3621915 | 62.9582208 | 27.29674253 | 32.68035316 |
| EXO1 | exonuclease 1 | 0.00026415 | -2.430129753 | 70.09734874 | 52.4256293 | 27.14791164 | 22.92186192 |
| CDKN1A | cyclin-dependent kinase inhibitor 1A (p21. Cip1) | 2.99E-05 | -2.430564865 | 4126.375845 | 4087.605444 | 1760.78938 | 1621.496106 |
| YWHAE | tyrosine 3-monooxygenase/tryptophan 5-monooxygenase activation protein. epsilon polypeptide | 0.00194059 | -2.441647784 | 57.52333526 | 81.89706009 | 24.88858429 | 31.75017665 |
| CLEC2D | C-type lectin domain family 2. member D | 0.00058507 | -2.470646296 | 81.60473384 | 74.65033872 | 36.99739283 | 26.97459074 |
| HELLS | helicase. lymphoid-specific | 0.000232 | -2.47520386 | 103.9079695 | 122.3435284 | 52.41113261 | 39.58989659 |
| SLC39A9 | solute carrier family 39 (zinc transporter). member 9 | 0.00016839 | -2.475223951 | 107.6240771 | 85.81419717 | 35.29324975 | 42.71180618 |
| LONP1 | lon peptidase 1. mitochondrial | 0.00015115 | -2.476265464 | 1740.02331 | 1966.204336 | 833.3272168 | 669.5357142 |
| PCK2 | phosphoenolpyruvate carboxykinase 2 (mitochondrial) | 0.00030854 | -2.492743473 | 129.1765726 | 175.806172 | 66.93723339 | 54.60028897 |
| ULBP1 | UL16 binding protein 1 | 8.37E-05 | -2.502565992 | 137.9461777 | 164.2213919 | 55.22451561 | 65.49926216 |
| PCK2 | phosphoenolpyruvate carboxykinase 2 (mitochondrial) | 9.10E-05 | -2.507999107 | 88.24841015 | 79.46729763 | 30.11819077 | 37.01789334 |
| CTH | cystathionase (cystathionine gamma-lyase) | 8.17E-05 | -2.512952405 | 141.9057699 | 118.608342 | 54.21039694 | 49.16592063 |
| ASCC3 | activating signal cointegrator 1 complex subunit 3 | 9.98E-05 | -2.541104462 | 68.04890891 | 69.06859238 | 24.00382059 | 30.32329297 |
| H2AFY | H2A histone family. member Y | 0.00116938 | -2.547569671 | 60.8788882 | 43.79385579 | 22.9888424 | 17.86942811 |
| DTL | denticleless homolog (Drosophila) | 2.36E-05 | -2.562976549 | 107.7657473 | 99.1068534 | 38.46398633 | 42.27083978 |
| KLHL4 | kelch-like 4 (Drosophila) | 0.00118067 | -2.603299593 | 57.67623673 | 56.33782615 | 17.36188528 | 27.61543048 |
| PIM1 | pim-1 oncogene | 2.27E-05 | -2.640845111 | 419.8262407 | 416.0360217 | 154.4505099 | 162.1531196 |
| PPP1R15A | protein phosphatase 1. regulatory (inhibitor) subunit 15A | 3.91E-05 | -2.652334097 | 976.2990469 | 1070.1962 | 419.1147448 | 354.3699386 |
| C13orf18 | chromosome 13 open reading frame 18 | 3.55E-05 | -2.655253111 | 748.0915101 | 802.6772707 | 283.9879523 | 299.9051492 |
| FAM18B2 | family with sequence similarity 18. member B2 | 0.00024118 | -2.665611062 | 88.84694749 | 85.13530095 | 26.93198135 | 39.52667819 |
| STC2 | stanniocalcin 2 | 6.59E-05 | -2.684179617 | 1013.839602 | 972.4478863 | 413.8397767 | 330.658894 |
| PSMC3IP | PSMC3 interacting protein | 0.00028185 | -2.690457108 | 57.40505193 | 63.32939868 | 24.93306842 | 20.14316008 |
| GPT2 | glutamic pyruvate transaminase (alanine aminotransferase) 2 | 1.47E-05 | -2.696869115 | 2316.334373 | 2085.721586 | 814.9095869 | 815.1328982 |
| NFE2L1 | nuclear factor (erythroid-derived 2)-like 1 | 2.84E-05 | -2.704263853 | 715.9439328 | 776.2913673 | 260.2028047 | 292.0745189 |
| PKD1 | polycystic kidney disease 1 (autosomal dominant) | 0.00020448 | -2.713060387 | 98.01445038 | 69.23767061 | 32.20104119 | 28.63147501 |
| RELB | v-rel reticuloendotheliosis viral oncogene homolog B | 1.35E-05 | -2.716667862 | 307.5328762 | 307.4173687 | 119.0478377 | 107.6032402 |
| CADPS | Ca++-dependent secretion activator | 1.00E-05 | -2.764613265 | 84.30939378 | 88.94438783 | 31.9144931 | 30.74240156 |
| CTH | cystathionase (cystathionine gamma-lyase) | 5.45E-05 | -2.764993499 | 342.5021064 | 320.5483204 | 113.4830785 | 126.5427928 |
| WDR76 | WD repeat domain 76 | 0.00022654 | -2.793225446 | 76.2802985 | 108.0863558 | 30.89818303 | 34.20096322 |
| WARS | tryptophanyl-tRNA synthetase | 9.67E-06 | -2.826624807 | 3339.592818 | 3091.14403 | 1135.713208 | 1137.647589 |
| TGM2 | transglutaminase 2 (C polypeptide. protein-glutamine-gamma-glutamyltransferase) | 3.87E-05 | -2.828218886 | 74.65033872 | 84.39139186 | 29.8645763 | 26.37227154 |
| PCK2 | phosphoenolpyruvate carboxykinase 2 (mitochondrial) | 2.43E-05 | -2.857303854 | 1172.561726 | 1203.880534 | 445.9151224 | 387.7520043 |
| STMN1 | stathmin 1 | 2.09E-05 | -2.919791193 | 62.98606821 | 72.88991991 | 22.85442178 | 23.56341031 |
| PELI1 | pellino homolog 1 (Drosophila) | 7.09E-06 | -2.979845879 | 847.5069782 | 901.2570298 | 292.3976776 | 294.1917057 |
| CLIC4 | chloride intracellular channel 4 | 0.00022802 | -2.986711192 | 133.5043457 | 157.7010401 | 41.1723037 | 57.32424371 |
| FLJ35024 | hypothetical LOC401491 | 5.80E-05 | -3.025210346 | 83.81037754 | 91.27993746 | 32.9673018 | 25.3558733 |
| PLEK | pleckstrin | 7.82E-06 | -3.02621127 | 2425.700845 | 2390.772875 | 830.7255173 | 762.2887665 |
| PAOX | polyamine oxidase (exo-N4-amino) | 5.55E-05 | -3.029944787 | 114.4541431 | 130.4981613 | 46.45447634 | 35.02183621 |
| PSMC3IP | PSMC3 interacting protein | 0.00033264 | -3.08281067 | 91.51018572 | 111.9569736 | 25.99994726 | 41.46239772 |
| RRM2 | ribonucleotide reductase M2 | 2.65E-05 | -3.094618616 | 115.3658915 | 90.34845409 | 32.01192286 | 33.99946812 |
| CTH | cystathionase (cystathionine gamma-lyase) | 0.00012321 | -3.176278 | 130.4981613 | 156.2002574 | 37.24076297 | 54.25384905 |
| SESN2 | sestrin 2 | 0.00439969 | -3.213595347 | 83.79456103 | 127.5505124 | 36.77054171 | 28.14593172 |
| ARMCX3 | armadillo repeat containing. X-linked 3 | 4.20E-05 | -3.351707373 | 88.79926282 | 93.36097067 | 25.06354382 | 29.44421987 |
| ALPK2 | alpha-kinase 2 | 2.01E-05 | -3.36184172 | 299.6304103 | 323.2441533 | 88.63837363 | 96.68080627 |
| MYB | v-myb myeloblastosis viral oncogene homolog (avian) | 7.95E-06 | -3.369173635 | 1201.639853 | 1126.912172 | 333.2191915 | 358.0037698 |
| SGK1 | serum/glucocorticoid regulated kinase 1 | 5.54E-06 | -3.468237563 | 166.3247125 | 145.154374 | 46.64817403 | 43.02635784 |
| STS | steroid sulfatase (microsomal). isozyme S | 3.71E-06 | -3.508804967 | 120.5011344 | 115.9213719 | 32.06061547 | 35.3886776 |
| DDIT3 | DNA-damage-inducible transcript 3 | 1.77E-06 | -4.047893669 | 517.222231 | 497.638666 | 122.0093586 | 128.7478439 |
| SGK1 | serum/glucocorticoid regulated kinase 1 | 3.39E-05 | -4.103195974 | 150.2971919 | 120.6932374 | 34.02916112 | 31.66197782 |
| CHAC1 | ChaC. cation transport regulator homolog 1 (E. coli) | 3.10E-05 | -4.23669158 | 253.6137323 | 254.102819 | 49.77082615 | 72.13627288 |
| SGK1 | serum/glucocorticoid regulated kinase 1 | 4.16E-06 | -4.834588007 | 146.329825 | 144.7959949 | 26.39786025 | 34.34011431 |
| **Symbol** | **Description** | **P.Value** | **Foldchange** | **Control -1** | **Control -2** | **CpdA 1uM 16h - 1** | **CpdA 1uM 16h - 2** |
| LAMP3 | lysosomal-associated membrane protein 3 | 0.001227 | 2.158999116 | 27.44384 | 27.47773102 | 72.45062301 | 48.51639635 |
| CHTF8 | CTF8. chromosome transmission fidelity factor 8 homolog (S. cerevisiae) | 0.06741 | 2.096262785 | 18.39117 | 27.18045612 | 81.47333302 | 26.96137992 |
| NAP1L1 | nucleosome assembly protein 1-like 1 | 0.00043 | 1.98946815 | 20.10497 | 16.0459567 | 34.69927957 | 36.79785936 |
| MIR320C1 | microRNA 320c-1 | 0.003885 | 1.975092893 | 11.81303 | 16.88615995 | 32.14566323 | 24.20722107 |
| C15orf29 | chromosome 15 open reading frame 29 | 0.002341 | 1.974155246 | 38.25072 | 26.09445254 | 66.89418087 | 58.15165632 |
| HSPC159 | galectin-related protein | 0.006912 | 1.964442862 | 46.55627 | 33.41708119 | 91.45252132 | 65.64926131 |
| BLVRB | biliverdin reductase B (flavin reductase (NADPH)) | 0.000257 | 1.942178399 | 75.13022 | 68.89093271 | 143.2228416 | 136.3146946 |
| PRR15 | proline rich 15 | 0.000865 | 1.936846997 | 21.45278 | 19.3503572 | 43.79385579 | 35.55903113 |
| LOC100130518 | similar to Zinc finger protein ENSP00000350085 | 0.012841 | 1.856687872 | 19.70917 | 18.47273296 | 47.35304539 | 26.50509242 |
| TMEM106B | transmembrane protein 106B | 0.005543 | 1.825811159 | 41.52971 | 27.63740866 | 62.7001442 | 61.02382195 |
| MAPK8 | mitogen-activated protein kinase 8 | 0.027247 | 1.81617119 | 14.33829 | 20.14837411 | 39.77447452 | 23.95776941 |
| MYEF2 | myelin expression factor 2 | 0.084936 | 1.788266734 | 20.22247 | 16.65450318 | 23.80376043 | 45.24649043 |
| MLL5 | myeloid/lymphoid or mixed-lineage leukemia 5 (trithorax homolog. Drosophila) | 0.024435 | 1.783069882 | 30.50294 | 16.77319236 | 36.86411317 | 44.12557597 |
| RNU4-2 | RNA. U4 small nuclear 2 | 0.016 | 1.772872697 | 49.00566 | 28.30697649 | 69.01393771 | 63.17685555 |
| MOBKL1A | MOB1. Mps One Binder kinase activator-like 1A (yeast) | 0.000486 | 1.764709894 | 49.08946 | 51.40428742 | 93.18193187 | 84.33395941 |
| IDS | iduronate 2-sulfatase | 0.027407 | 1.762133891 | 35.85015 | 29.67360251 | 71.95818449 | 45.90487749 |
| RNU6ATAC | RNA. U6atac small nuclear (U12-dependent splicing) | 0.016859 | 1.758267785 | 24.58351 | 15.96516287 | 38.23787458 | 31.73170851 |
| USP25 | ubiquitin specific peptidase 25 | 0.001416 | 1.755714068 | 22.2445 | 18.81523468 | 34.92422883 | 36.94137594 |
| LOC100133017 | hypothetical LOC100133017 | 0.017403 | 1.754336518 | 42.38553 | 33.99946812 | 85.75283422 | 51.72102093 |
| PPIL3 | peptidylprolyl isomerase (cyclophilin)-like 3 | 0.007944 | 1.753533802 | 29.2038 | 22.649599 | 52.47195198 | 38.76152738 |
| SLC12A2 | solute carrier family 12 (sodium/potassium/chloride transporters). member 2 | 0.007969 | 1.748798136 | 24.70242 | 24.66752355 | 53.51487274 | 34.82329558 |
| NUP43 | nucleoporin 43kDa | 0.002281 | 1.747713938 | 37.90635 | 29.54516992 | 54.17649539 | 63.14343476 |
| ORAI2 | ORAI calcium release-activated calcium modulator 2 | 0.038382 | 1.744439217 | 31.54184 | 35.13812981 | 60.91788763 | 55.3646498 |
| INF2 | inverted formin. FH2 and WH2 domain containing | 0.000786 | 1.743668966 | 28.45925 | 32.28485857 | 54.08454908 | 51.65082151 |
| LOC100134067 | hypothetical protein LOC100134067 | 0.207344 | 1.729320824 | 28.11516 | 18.53148697 | 20.76848235 | 75.02347589 |
| TELO2 | TEL2. telomere maintenance 2. homolog (S. cerevisiae) | 0.082129 | 1.728448935 | 36.83724 | 74.55158837 | 120.207237 | 68.25373539 |
| TMEM128 | transmembrane protein 128 | 0.026289 | 1.727937918 | 27.75443 | 30.21706353 | 43.26109597 | 57.88198212 |
| LOC648987 | hypothetical LOC648987 | 0.000924 | 1.726540439 | 20.5224 | 22.77197532 | 40.19378069 | 34.65959793 |
| GSTTP2 | glutathione S-transferase theta pseudogene 2 | 0.003388 | 1.72088781 | 49.0736 | 64.45063326 | 99.2895857 | 94.33580198 |
| GPR135 | G protein-coupled receptor 135 | 0.097739 | 1.717603123 | 14.31648 | 17.87985979 | 17.42983194 | 43.32640702 |
| FAM75B | family with sequence similarity 75. member B | 0.006832 | 1.716046377 | 30.96612 | 30.5561286 | 58.23547982 | 47.84708335 |
| MAPK9 | mitogen-activated protein kinase 9 | 0.015369 | 1.713220579 | 34.74852 | 29.21201738 | 60.71227872 | 49.0736023 |
| PTGR2 | prostaglandin reductase 2 | 0.058032 | 1.709750714 | 44.65022 | 48.67541009 | 89.74228122 | 70.7948634 |
| OR2L13 | olfactory receptor. family 2. subfamily L. member 13 | 0.050593 | 1.707833038 | 17.00984 | 15.6640893 | 19.29890061 | 40.26834216 |
| C1orf63 | chromosome 1 open reading frame 63 | 0.013879 | 1.705431793 | 200.2471 | 136.6358494 | 296.9756542 | 267.9654664 |
| ZRSR2 | zinc finger (CCCH type). RNA-binding motif and serine/arginine rich 2 | 0.024144 | 1.702515864 | 25.95892 | 36.86411317 | 41.9921817 | 66.05479789 |
| B9D1 | B9 protein domain 1 | 0.000638 | 1.70212408 | 39.34695 | 39.29394397 | 65.84583642 | 68.02849357 |
| LOC729222 | similar to PTPRF interacting protein binding protein 1 | 0.003147 | 1.697989115 | 21.14541 | 16.68933713 | 33.06471058 | 30.772324 |
| WASH7P | WAS protein family homolog 7 pseudogene | 0.007057 | 1.695086002 | 43.7152 | 37.99856247 | 81.40997319 | 58.62805285 |
| LOC100129002 | hypothetical LOC100129002 | 0.063127 | 1.689769027 | 14.64234 | 22.09480754 | 42.34436018 | 21.81522841 |
| C14orf104 | chromosome 14 open reading frame 104 | 0.001944 | 1.688576648 | 18.71184 | 20.80166748 | 33.50484376 | 33.12444392 |
| MBD2 | methyl-CpG binding domain protein 2 | 0.024811 | 1.683808957 | 16.94043 | 15.23572719 | 21.72812751 | 33.67836498 |
| ECE2 | endothelin converting enzyme 2 | 0.029728 | 1.674762126 | 18.66323 | 16.55505518 | 21.93662973 | 39.50514695 |
| EFR3B | EFR3 homolog B (S. cerevisiae) | 0.002615 | 1.673297055 | 30.43951 | 26.83935378 | 42.6654046 | 53.61421146 |
| C1orf88 | chromosome 1 open reading frame 88 | 0.041758 | 1.669389911 | 16.09156 | 24.0602019 | 42.37920684 | 25.46010125 |
| GLI4 | GLI family zinc finger 4 | 0.032337 | 1.669163309 | 37.30324 | 23.67157646 | 59.42827219 | 41.39789694 |
| ZNF563 | zinc finger protein 563 | 0.158142 | 1.667921021 | 40.96879 | 33.09280193 | 58.49514035 | 64.47893939 |
| LOC100133578 | similar to acetylserotonin O-methyltransferase-like | 0.033611 | 1.666446128 | 30.93233 | 28.4259759 | 63.32939868 | 38.55718119 |
| RIBC1 | RIB43A domain with coiled-coils 1 | 0.028375 | 1.659297368 | 17.96531 | 25.30753359 | 40.48005718 | 30.9237381 |
| MIR1323 | microRNA 1323 | 0.03181 | 1.658303371 | 18.39076 | 23.06650153 | 44.38812948 | 26.28103316 |
| CALN1 | calneuron 1 | 0.03494 | 1.658290781 | 16.93679 | 18.49296643 | 39.62019126 | 21.73914634 |
| CCDC50 | coiled-coil domain containing 50 | 0.017966 | 1.655404641 | 23.22382 | 28.84265503 | 53.44839492 | 34.34334485 |
| CBFA2T3 | core-binding factor. runt domain. alpha subunit 2; translocated to. 3 | 0.029789 | 1.655109874 | 21.71607 | 24.0847892 | 49.00565817 | 29.23691008 |
| RRM2B | ribonucleotide reductase M2 B (TP53 inducible) | 0.012609 | 1.647532821 | 57.1513 | 39.22883456 | 83.3621915 | 73.00128631 |
| C18orf32 | chromosome 18 open reading frame 32 | 0.034818 | 1.64715189 | 41.50125 | 50.66988163 | 84.42834733 | 67.57562993 |
| SNAR-A1 | small ILF3/NF90-associated RNA A1 | 0.006839 | 1.639444713 | 18.36554 | 24.59277086 | 34.30595942 | 35.38630438 |
| CYorf15A | chromosome Y open reading frame 15A | 0.00438 | 1.638660379 | 19.70213 | 16.7591193 | 26.41965883 | 33.55947621 |
| KCNMB2 | potassium large conductance calcium-activated channel. subfamily M. beta member 2 | 0.013987 | 1.634483671 | 23.39439 | 22.9680625 | 46.72553902 | 30.72153511 |
| GLTPD1 | glycolipid transfer protein domain containing 1 | 0.000688 | 1.633301929 | 52.34379 | 51.30900401 | 86.02798505 | 83.28215006 |
| CCDC132 | coiled-coil domain containing 132 | 0.064885 | 1.632444389 | 15.13246 | 13.87328529 | 16.41484552 | 34.08228524 |
| SLC6A1 | solute carrier family 6 (neurotransmitter transporter. GABA). member 1 | 0.089331 | 1.63113207 | 13.77178 | 16.07140577 | 16.36806944 | 35.97697567 |
| ASCL5 | achaete-scute complex homolog 5 (Drosophila) | 0.011489 | 1.627432175 | 30.16927 | 21.58158188 | 37.91065595 | 45.48754875 |
| GFER | growth factor. augmenter of liver regeneration | 0.018378 | 1.627430243 | 17.30685 | 23.47227602 | 28.84805627 | 37.29592268 |
| IDH3G | isocitrate dehydrogenase 3 (NAD+) gamma | 0.039809 | 1.624126366 | 18.10226 | 19.19773738 | 40.79355922 | 22.47143929 |
| KCNQ2 | potassium voltage-gated channel. KQT-like subfamily. member 2 | 0.059612 | 1.623699368 | 18.96425 | 14.09137457 | 36.0040771 | 19.56809941 |
| MYO3B | myosin IIIB | 0.042931 | 1.620394826 | 38.79889 | 30.83129898 | 43.78656125 | 71.73184839 |
| ITGA3 | integrin. alpha 3 (antigen CD49C. alpha 3 subunit of VLA-3 receptor) | 0.015166 | 1.61897749 | 36.11524 | 24.74371109 | 44.82407283 | 52.25473397 |
| ZBTB20 | zinc finger and BTB domain containing 20 | 0.023869 | 1.609901178 | 30.32947 | 33.00724944 | 61.53709911 | 42.163392 |
| GMPPB | GDP-mannose pyrophosphorylase B | 0.026604 | 1.609678835 | 59.41536 | 58.90257219 | 114.139793 | 79.44642564 |
| ZBTB7B | zinc finger and BTB domain containing 7B | 0.004112 | 1.609463478 | 25.16869 | 25.96156882 | 39.94560405 | 42.37257354 |
| ASXL2 | additional sex combs like 2 (Drosophila) | 0.001615 | 1.607493182 | 190.013 | 213.3373601 | 337.0724961 | 310.7600866 |
| CFHR2 | complement factor H-related 2 | 0.034769 | 1.607296056 | 18.17143 | 19.95129079 | 24.30822867 | 38.52995566 |
| OR51F1 | olfactory receptor. family 51. subfamily F. member 1 | 0.011164 | 1.607258282 | 16.64352 | 16.06303603 | 31.69743477 | 21.78813992 |
| ARRDC3 | arrestin domain containing 3 | 0.100804 | 1.603751855 | 32.63749 | 15.54300863 | 40.3082637 | 32.36920931 |
| LOC729324 | hCG1986447 | 0.017912 | 1.601407828 | 21.22416 | 22.59080955 | 43.85308169 | 28.03922657 |
| C9orf38 | chromosome 9 open reading frame 38 | 0.002797 | 1.600506259 | 28.31815 | 30.86531487 | 51.70353891 | 43.30420298 |
| MREG | melanoregulin | 0.023943 | 1.600325012 | 30.44238 | 27.68569198 | 43.15805263 | 50.0136466 |
| SPRED3 | sprouty-related. EVH1 domain containing 3 | 0.029968 | 1.599667352 | 18.8217 | 15.57759372 | 35.12358443 | 21.36091429 |
| C15orf2 | chromosome 15 open reading frame 2 | 0.092721 | 1.599520025 | 15.96419 | 21.88379704 | 43.2878341 | 20.6482388 |
| CYP2C9 | cytochrome P450. family 2. subfamily C. polypeptide 9 | 0.127806 | 1.599489177 | 16.6615 | 16.2735007 | 40.76196442 | 17.01776805 |
| MIB1 | mindbomb homolog 1 (Drosophila) | 0.00125 | 1.594457898 | 37.03486 | 39.4284719 | 62.93818221 | 58.98377064 |
| LOC441481 | glutathione peroxidase 1 pseudogene | 0.044588 | 1.59235817 | 59.47927 | 39.11948409 | 88.1058553 | 66.96309655 |
| C1QTNF9B | C1q and tumor necrosis factor related protein 9B | 0.127411 | 1.592306893 | 16.3343 | 13.64306869 | 15.22153478 | 37.11997114 |
| TIMM13 | translocase of inner mitochondrial membrane 13 homolog (yeast) | 0.042763 | 1.590702944 | 29.88643 | 17.89459907 | 35.53462472 | 38.0822374 |
| TEAD2 | TEA domain family member 2 | 0.008113 | 1.590557152 | 35.845 | 34.28609031 | 50.2227297 | 61.90772349 |
| TMPRSS12 | transmembrane (C-terminal) protease. serine 12 | 0.012197 | 1.590532577 | 37.13751 | 48.86648807 | 77.6823683 | 59.09988643 |
| CFB | complement factor B | 0.13562 | 1.590162629 | 19.2687 | 18.51199097 | 18.99237521 | 47.49078889 |
| CRCT1 | cysteine-rich C-terminal 1 | 0.034809 | 1.588763391 | 20.79803 | 19.23772095 | 40.54023289 | 24.91196561 |
| LOC100132701 | hypothetical LOC100132701 | 0.088395 | 1.584098374 | 41.53647 | 24.09719627 | 56.10145991 | 44.76990969 |
| ZFYVE16 | zinc finger. FYVE domain containing 16 | 0.016833 | 1.579965361 | 32.86375 | 49.59536362 | 62.36215663 | 65.24274308 |
| TEX19 | testis expressed 19 | 0.001067 | 1.579700499 | 488.8123 | 495.5754733 | 767.7476124 | 787.3774063 |
| ZSCAN5A | zinc finger and SCAN domain containing 5A | 0.024604 | 1.579580386 | 46.09805 | 32.94868098 | 51.76571277 | 73.20856562 |
| FAM101A | family with sequence similarity 101. member A | 0.026998 | 1.579338136 | 16.86211 | 21.82188926 | 36.8831261 | 24.88438128 |
| C12orf68 | chromosome 12 open reading frame 68 | 0.004992 | 1.5775259 | 32.13393 | 25.06137625 | 42.83847542 | 46.78296155 |
| CDKN1C | cyclin-dependent kinase inhibitor 1C (p57. Kip2) | 0.05139 | 1.574642575 | 16.87455 | 21.52519133 | 36.59751979 | 24.60886269 |
| CREB3 | cAMP responsive element binding protein 3 | 0.010845 | 1.573936779 | 46.17229 | 35.03948534 | 56.45320387 | 70.99457188 |
| CENPQ | centromere protein Q | 0.040323 | 1.572350365 | 40.26415 | 25.16700475 | 50.6036868 | 49.50698991 |
| PIK3R1 | phosphoinositide-3-kinase. regulatory subunit 1 (alpha) | 0.013482 | 1.571031581 | 28.35343 | 22.82423177 | 45.66075149 | 34.9807061 |
| PIGX | phosphatidylinositol glycan anchor biosynthesis. class X | 0.001694 | 1.566754626 | 81.37824 | 88.1058553 | 127.0689968 | 138.5081841 |
| HHATL | hedgehog acyltransferase-like | 0.003825 | 1.565229671 | 20.75283 | 19.62929562 | 28.37532873 | 35.17201645 |
| SNORD13 | small nucleolar RNA. C/D box 13 | 0.056462 | 1.565028423 | 139.7083 | 84.03130475 | 145.8771 | 197.1154824 |
| TNK2 | tyrosine kinase. non-receptor. 2 | 0.027574 | 1.564903987 | 21.18739 | 25.83227217 | 42.52034051 | 31.52235496 |
| UFM1 | ubiquitin-fold modifier 1 | 0.001868 | 1.563479924 | 193.7552 | 181.5297476 | 279.7619906 | 307.3244124 |
| TMC4 | transmembrane channel-like 4 | 0.016167 | 1.559774407 | 21.48304 | 18.93380117 | 38.04054082 | 26.01420346 |
| SSTR1 | somatostatin receptor 1 | 0.243014 | 1.558369412 | 14.69968 | 15.87511924 | 13.11653164 | 43.20626899 |
| RAB14 | RAB14. member RAS oncogene family | 0.035387 | 1.55767616 | 25.80558 | 26.81323462 | 40.68129932 | 41.26884184 |
| LOC100130745 | hypothetical LOC100130745 | 0.003546 | 1.555316407 | 25.69513 | 24.20010634 | 36.36337817 | 41.36579119 |
| SF1 | splicing factor 1 | 0.004221 | 1.555030616 | 25.3136 | 27.55302256 | 36.94137594 | 45.65496254 |
| NR2C1 | nuclear receptor subfamily 2. group C. member 1 | 0.047101 | 1.554072216 | 88.16521 | 59.06389919 | 94.40385395 | 133.2207951 |
| NEK8 | NIMA (never in mitosis gene a)- related kinase 8 | 0.001873 | 1.55203256 | 29.08918 | 26.21767402 | 43.98904764 | 41.76214606 |
| TAF4B | TAF4b RNA polymerase II. TATA box binding protein (TBP)-associated factor. 105kDa | 0.020044 | 1.551715032 | 34.41966 | 40.1296141 | 54.62037005 | 60.88928536 |
| SCARNA14 | small Cajal body-specific RNA 14 | 0.047789 | 1.548662777 | 96.71051 | 56.20666563 | 104.3635173 | 124.9184238 |
| MPST | mercaptopyruvate sulfurtransferase | 0.00278 | 1.546445396 | 68.87485 | 80.50518921 | 118.1068343 | 112.2738671 |
| LOC100133313 | similar to melanoma antigen | 0.057834 | 1.545922585 | 23.192 | 16.87687464 | 39.53881234 | 23.65822556 |
| UXT | ubiquitously-expressed transcript | 0.034771 | 1.543302799 | 27.70444 | 21.79077585 | 42.29142197 | 33.99946812 |
| NOD1 | nucleotide-binding oligomerization domain containing 1 | 0.014131 | 1.541073257 | 42.76669 | 36.97826426 | 51.84544227 | 72.44162342 |
| LPPR2 | lipid phosphate phosphatase-related protein type 2 | 0.017713 | 1.540904245 | 26.4358 | 21.11529012 | 36.0618158 | 36.75303057 |
| IMPDH1 | IMP (inosine 5'-monophosphate) dehydrogenase 1 | 0.077803 | 1.538778834 | 57.06706 | 30.46137081 | 58.11329556 | 70.82911683 |
| ZNF562 | zinc finger protein 562 | 0.013739 | 1.538507642 | 59.53441 | 65.54725256 | 113.9932893 | 81.02939586 |
| WDR47 | WD repeat domain 47 | 0.023006 | 1.538216382 | 65.07869 | 62.01411973 | 108.8028633 | 87.76551639 |
| FCHSD2 | FCH and double SH3 domains 2 | 0.002377 | 1.537609027 | 131.8112 | 135.717359 | 206.4689993 | 204.844755 |
| INSM1 | insulinoma-associated 1 | 0.012828 | 1.535293161 | 19.34071 | 23.00965534 | 37.66086433 | 27.85318239 |
| EYS | eyes shut homolog (Drosophila) | 0.094187 | 1.533774179 | 32.718 | 20.076854 | 51.30498345 | 30.11935615 |
| PPM1K | protein phosphatase. Mg2+/Mn2+ dependent. 1K | 0.019432 | 1.533686223 | 74.4677 | 71.02031172 | 122.4050956 | 101.6304993 |
| C2orf68 | chromosome 2 open reading frame 68 | 0.003252 | 1.533635278 | 42.33437 | 36.09706059 | 61.94652002 | 58.02193186 |
| ZNF512B | zinc finger protein 512B | 0.007049 | 1.53028638 | 29.21202 | 33.8147826 | 51.57472069 | 44.85147098 |
| POLR2E | polymerase (RNA) II (DNA directed) polypeptide E. 25kDa | 0.002049 | 1.527594117 | 32.11802 | 32.56088001 | 52.03184067 | 46.9020348 |
| CLTC | clathrin. heavy chain (Hc) | 0.001836 | 1.52703668 | 43.3042 | 41.43731385 | 67.00898183 | 62.44354691 |
| ARL16 | ADP-ribosylation factor-like 16 | 0.011003 | 1.526028869 | 114.7016 | 101.1933117 | 189.4946755 | 142.6427302 |
| NAAA | N-acylethanolamine acid amidase | 0.003436 | 1.524617595 | 30.67101 | 27.39676779 | 41.8842989 | 46.63349535 |
| ICA1L | islet cell autoantigen 1.69kDa-like | 0.140387 | 1.52445182 | 30.8313 | 38.18936116 | 34.94387785 | 78.30518571 |
| KIF23 | kinesin family member 23 | 0.00168 | 1.523923966 | 34.77767 | 32.87249594 | 51.68024658 | 51.37302302 |
| MFSD6 | major facilitator superfamily domain containing 6 | 0.002572 | 1.523152503 | 64.87064 | 68.84295899 | 107.1653025 | 96.68080627 |
| MAP3K5 | mitogen-activated protein kinase kinase kinase 5 | 0.003046 | 1.522362933 | 36.51214 | 31.23067795 | 51.45882553 | 51.35647333 |
| ZNF75A | zinc finger protein 75a | 0.078709 | 1.521495309 | 65.01114 | 41.55758856 | 94.41775278 | 66.24082092 |
| PIK3CG | phosphoinositide-3-kinase. catalytic. gamma polypeptide | 0.003976 | 1.519644668 | 80.21115 | 84.16501569 | 136.5271829 | 114.1908511 |
| HLA-DPB1 | major histocompatibility complex. class II. DP beta 1 | 0.004285 | 1.515715693 | 45.55952 | 40.11392822 | 69.33046013 | 60.55998517 |
| CSDE1 | cold shock domain containing E1. RNA-binding | 0.014136 | 1.514518393 | 38.93864 | 32.68546181 | 46.64817403 | 62.58206438 |
| LUC7L2 | LUC7-like 2 (S. cerevisiae) | 0.026066 | 1.514292686 | 108.8299 | 72.7253879 | 136.6358494 | 132.8278418 |
| AIG1 | androgen-induced 1 | 0.033415 | 1.51242636 | 42.65483 | 31.45304241 | 49.89048278 | 61.5122515 |
| LIPE | lipase. hormone-sensitive | 0.084283 | 1.511946753 | 42.41809 | 22.24734751 | 49.34803588 | 43.71519707 |
| PPIG | peptidylprolyl isomerase G (cyclophilin G) | 0.067889 | 1.510463594 | 18.92601 | 15.46512707 | 34.36227084 | 19.43351185 |
| SCARNA3 | small Cajal body-specific RNA 3 | 0.110897 | 1.50864506 | 21.11383 | 15.80987544 | 37.60069151 | 20.20569928 |
| FAM153B | family with sequence similarity 153. member B | 0.00303 | 1.507113587 | 39.98081 | 41.72220527 | 65.74847676 | 57.62688168 |
| TMEM64 | transmembrane protein 64 | 0.004804 | 1.506996124 | 22.97275 | 20.31170006 | 35.17576704 | 30.12589132 |
| DENND5B | DENN/MADD domain containing 5B | 0.039036 | 1.502633226 | 39.41133 | 29.36001619 | 49.77427264 | 52.4902247 |
| LAIR1 | leukocyte-associated immunoglobulin-like receptor 1 | 0.018016 | 1.502480547 | 20.93853 | 20.37041402 | 35.74796931 | 26.93472105 |
| THRB | thyroid hormone receptor. beta (erythroblastic leukemia viral (v-erb-a) oncogene homolog 2. avian) | 0.116966 | 1.500771565 | 15.02808 | 16.25627896 | 34.08038349 | 16.14542023 |
| PRMT2 | protein arginine methyltransferase 2 | 0.022318 | -1.500907771 | 123.4932 | 122.5336684 | 99.76718146 | 67.32911274 |
| FBXO7 | F-box protein 7 | 0.018676 | -1.500939305 | 147.4071 | 128.9760151 | 77.7937974 | 108.4816443 |
| TSC22D4 | TSC22 domain family. member 4 | 0.006423 | -1.50100483 | 184.4009 | 165.8945647 | 116.2006595 | 116.8482936 |
| DHODH | dihydroorotate dehydrogenase | 0.003758 | -1.501902016 | 34.75769 | 35.95306446 | 23.97535205 | 23.10673766 |
| CCL22 | chemokine (C-C motif) ligand 22 | 0.03891 | -1.502262386 | 39.69619 | 25.18291556 | 20.17859165 | 21.95191208 |
| SLC25A40 | solute carrier family 25. member 40 | 0.002721 | -1.502362666 | 111.0876 | 101.9401383 | 72.30417254 | 69.39015862 |
| OPN3 | opsin 3 | 0.005119 | -1.504720493 | 79.66818 | 84.52345448 | 60.16641994 | 49.4306466 |
| BCL2L1 | BCL2-like 1 | 0.021718 | -1.504843464 | 31.19835 | 37.32937683 | 26.23112043 | 19.60572207 |
| ABCF2 | ATP-binding cassette. sub-family F (GCN20). member 2 | 0.007723 | -1.505412293 | 257.5331 | 239.5219911 | 170.4857245 | 159.6538963 |
| AP1B1 | adaptor-related protein complex 1. beta 1 subunit | 0.067643 | -1.506238921 | 31.85614 | 21.31293539 | 18.89384873 | 15.83901823 |
| RRN3P2 | RNA polymerase I transcription factor homolog (S. cerevisiae) pseudogene 2 | 0.01641 | -1.506287113 | 67.45377 | 58.40789748 | 41.54960275 | 41.79221859 |
| KIAA2026 | KIAA2026 | 0.057363 | -1.506610645 | 24.74832 | 38.09339023 | 17.11925313 | 24.26099162 |
| PPM1B | protein phosphatase. Mg2+/Mn2+ dependent. 1B | 0.034591 | -1.507034912 | 40.30826 | 53.49172404 | 27.68428381 | 34.29262791 |
| IL32 | interleukin 32 | 0.008035 | -1.507427634 | 62.24791 | 53.32418362 | 35.22588866 | 41.46809272 |
| RBBP6 | retinoblastoma binding protein 6 | 0.003916 | -1.509709546 | 184.1665 | 188.607526 | 134.5669896 | 113.2516015 |
| DMPK | dystrophia myotonica-protein kinase | 0.003478 | -1.510153853 | 33.91775 | 31.99191378 | 23.27787845 | 20.44002782 |
| BAT2 | HLA-B associated transcript 2 | 0.003908 | -1.510208531 | 143.2228 | 149.3875133 | 105.3995121 | 89.00494686 |
| C9orf41 | chromosome 9 open reading frame 41 | 0.017845 | -1.510817541 | 45.86891 | 48.77519137 | 35.70457148 | 27.45171867 |
| OXNAD1 | oxidoreductase NAD-binding domain containing 1 | 0.005451 | -1.511243546 | 157.0842 | 156.2368499 | 115.5651513 | 92.98653666 |
| TBC1D25 | TBC1 domain family. member 25 | 0.0454 | -1.51125922 | 33.92625 | 37.85227881 | 30.38948184 | 18.50233948 |
| CD79A | CD79a molecule. immunoglobulin-associated alpha | 0.003243 | -1.51152734 | 691.3707 | 774.905154 | 477.8441902 | 490.7284307 |
| TSPYL2 | TSPY-like 2 | 0.002402 | -1.511721231 | 83.16646 | 78.15571736 | 50.97277081 | 55.79911638 |
| TBC1D17 | TBC1 domain family. member 17 | 0.087984 | -1.512395118 | 41.49105 | 79.06482953 | 33.95561899 | 42.23724553 |
| NOMO3 | NODAL modulator 3 | 0.015077 | -1.514849501 | 60.30987 | 59.71146106 | 45.634268 | 34.38872355 |
| C19orf20 | chromosome 19 open reading frame 20 | 0.002997 | -1.516662407 | 35.81397 | 40.56506241 | 25.07804653 | 25.18448606 |
| RABGGTA | Rab geranylgeranyltransferase. alpha subunit | 0.007776 | -1.518182974 | 35.78658 | 42.81398745 | 26.83699565 | 24.7698708 |
| SLC38A1 | solute carrier family 38. member 1 | 0.002769 | -1.518486781 | 119.008 | 128.8850546 | 81.94304008 | 81.17918201 |
| GGA1 | golgi-associated. gamma adaptin ear containing. ARF binding protein 1 | 0.012283 | -1.518654212 | 24.95506 | 33.03172322 | 18.11460545 | 19.7307326 |
| CSGALNACT2 | chondroitin sulfate N-acetylgalactosaminyltransferase 2 | 0.00771 | -1.519770497 | 54.82802 | 62.5109969 | 42.98682577 | 34.51974561 |
| VPS25 | vacuolar protein sorting 25 homolog (S. cerevisiae) | 0.008932 | -1.519906038 | 403.8726 | 337.1412381 | 268.52648 | 219.5005535 |
| CNOT4 | CCR4-NOT transcription complex. subunit 4 | 0.001719 | -1.52029354 | 35.24857 | 35.44357436 | 23.54362537 | 22.95888231 |
| C1orf89 | chromosome 1 open reading frame 89 | 0.010219 | -1.520409987 | 42.37257 | 35.85014754 | 22.6123397 | 29.06088669 |
| FURIN | furin (paired basic amino acid cleaving enzyme) | 0.018436 | -1.520494056 | 38.5804 | 27.69749221 | 23.67421142 | 19.52370109 |
| KAT5 | K(lysine) acetyltransferase 5 | 0.031021 | -1.521106844 | 34.08038 | 53.71889409 | 29.09229008 | 27.19782572 |
| CELF1 | CUGBP. Elav-like family member 1 | 0.011566 | -1.521403978 | 56.15716 | 48.51639635 | 39.2486726 | 29.9902147 |
| PPARD | peroxisome proliferator-activated receptor delta | 0.07208 | -1.521612511 | 47.83984 | 67.00898183 | 31.14610584 | 44.45400203 |
| C1orf130 | chromosome 1 open reading frame 130 | 0.009819 | -1.522411503 | 32.8755 | 31.32518476 | 19.5689458 | 22.70567966 |
| NPEPPS | aminopeptidase puromycin sensitive | 0.115405 | -1.522491188 | 27.2047 | 25.50277985 | 15.00409794 | 19.94858322 |
| PDLIM5 | PDZ and LIM domain 5 | 0.170465 | -1.523401212 | 26.40855 | 20.1917128 | 17.72349716 | 12.96402144 |
| SUOX | sulfite oxidase | 0.008066 | -1.523450593 | 32.10678 | 33.91461739 | 20.19518256 | 23.23160287 |
| PDIA3P | protein disulfide isomerase family A. member 3 pseudogene | 0.021031 | -1.523556813 | 195.1627 | 272.6409181 | 168.9941532 | 135.6434286 |
| UBE2L3 | ubiquitin-conjugating enzyme E2L 3 | 0.041535 | -1.523756109 | 34.35068 | 40.81201514 | 29.84530601 | 20.2309563 |
| HOXB6 | homeobox B6 | 0.040668 | -1.524436048 | 25.30891 | 37.91600846 | 18.15887465 | 22.73991169 |
| RIMBP3B | RIMS binding protein 3B | 0.050359 | -1.524812431 | 27.94218 | 36.3018939 | 23.99703922 | 18.1802148 |
| DCAF6 | DDB1 and CUL4 associated factor 6 | 0.061722 | -1.525101216 | 87.62323 | 72.40879514 | 54.24304635 | 50.28855472 |
| ULBP2 | UL16 binding protein 2 | 0.076744 | -1.525303711 | 40.40456 | 51.7399646 | 22.28617407 | 40.31887 |
| SLC29A2 | solute carrier family 29 (nucleoside transporters). member 2 | 0.003155 | -1.526781628 | 29.82629 | 29.00087503 | 18.09941542 | 20.50179848 |
| HSPA4 | heat shock 70kDa protein 4 | 0.002765 | -1.527309452 | 69.7563 | 64.98744514 | 42.35692855 | 45.88115405 |
| BST2 | bone marrow stromal cell antigen 2 | 0.005092 | -1.527838843 | 707.0231 | 865.5186039 | 495.76432 | 528.7856532 |
| WRNIP1 | Werner helicase interacting protein 1 | 0.006867 | -1.528347246 | 96.34621 | 84.2297822 | 65.89518626 | 52.72329538 |
| DNMT3B | DNA (cytosine-5-)-methyltransferase 3 beta | 0.034681 | -1.528456306 | 41.17726 | 49.60657713 | 33.22998724 | 26.31240375 |
| RCE1 | RCE1 homolog. prenyl protein peptidase (S. cerevisiae) | 0.010651 | -1.528789279 | 43.62094 | 47.39097525 | 30.93872861 | 28.58860494 |
| SC4MOL | sterol-C4-methyl oxidase-like | 0.004431 | -1.529543149 | 208.5741 | 184.5103134 | 136.4365483 | 120.5665728 |
| ACSS3 | acyl-CoA synthetase short-chain family member 3 | 0.036588 | -1.529998627 | 31.1927 | 19.79045298 | 15.76072977 | 16.73209355 |
| TAGAP | T-cell activation RhoGTPase activating protein | 0.048617 | -1.530048112 | 135.5511 | 208.2842251 | 105.2112333 | 114.6270856 |
| STARD7 | StAR-related lipid transfer (START) domain containing 7 | 0.004104 | -1.530239777 | 33.55586 | 40.13448601 | 25.1357822 | 22.8809833 |
| SNAP23 | synaptosomal-associated protein. 23kDa | 0.003651 | -1.530246237 | 400.5788 | 470.6580006 | 297.1143964 | 270.9861269 |
| CDC42SE1 | CDC42 small effector 1 | 0.002533 | -1.531118334 | 86.54147 | 81.62448777 | 58.40789748 | 51.58882531 |
| PIN4 | protein (peptidylprolyl cis/trans isomerase) NIMA-interacting. 4 (parvulin) | 0.025889 | -1.531144955 | 33.52444 | 27.44216115 | 18.09802993 | 21.68282499 |
| MIR1228 | microRNA 1228 | 0.016679 | -1.531356978 | 89.76268 | 79.57304018 | 61.6886065 | 49.37463515 |
| SNCB | synuclein. beta | 0.039972 | -1.531387243 | 37.65374 | 50.73022649 | 35.00813188 | 23.26676316 |
| USP1 | ubiquitin specific peptidase 1 | 0.01298 | -1.53172832 | 48.32912 | 50.74596646 | 27.77972733 | 37.62867515 |
| INO80E | INO80 complex subunit E | 0.002729 | -1.531921085 | 305.111 | 351.7787967 | 210.2639048 | 217.5154025 |
| CCL3L1 | chemokine (C-C motif) ligand 3-like 1 | 0.004019 | -1.5325664 | 4007.143 | 3834.447057 | 2594.737162 | 2521.19049 |
| MAGOH | mago-nashi homolog. proliferation-associated (Drosophila) | 0.01215 | -1.532897131 | 192.0736 | 239.0390888 | 128.2299626 | 152.3775799 |
| TBC1D2 | TBC1 domain family. member 2 | 0.003981 | -1.533276925 | 49.8493 | 54.5722464 | 31.41250972 | 36.83723562 |
| BEND7 | BEN domain containing 7 | 0.006401 | -1.533610831 | 30.17205 | 37.01789334 | 23.36176652 | 20.32733791 |
| DHX40 | DEAH (Asp-Glu-Ala-His) box polypeptide 40 | 0.005872 | -1.533894501 | 88.43639 | 102.5321757 | 67.78248763 | 56.85683875 |
| THRAP3 | thyroid hormone receptor associated protein 3 | 0.0144 | -1.534250702 | 133.5788 | 143.3862522 | 75.6669475 | 107.5340698 |
| HSPA13 | heat shock protein 70kDa family. member 13 | 0.005066 | -1.534642886 | 79.57304 | 86.67397689 | 55.74856003 | 52.52987405 |
| PLK3 | polo-like kinase 3 | 0.03509 | -1.534955335 | 32.89633 | 24.9672395 | 15.30180546 | 22.78157586 |
| GRB2 | growth factor receptor-bound protein 2 | 0.014602 | -1.536083673 | 340.0194 | 425.89008 | 276.3293979 | 222.0978529 |
| FSHB | follicle stimulating hormone. beta polypeptide | 0.004899 | -1.536509532 | 29.43072 | 28.53394549 | 17.12724114 | 20.76848235 |
| ABTB1 | ankyrin repeat and BTB (POZ) domain containing 1 | 0.007347 | -1.536587405 | 44.79035 | 41.16639855 | 30.86805069 | 25.29901529 |
| SEH1L | SEH1-like (S. cerevisiae) | 0.023423 | -1.538410466 | 122.2411 | 86.25431681 | 73.09829062 | 60.94616287 |
| PRR13 | proline rich 13 | 0.011672 | -1.538748887 | 50.81021 | 64.12664038 | 32.8502574 | 41.89046767 |
| HTRA4 | HtrA serine peptidase 4 | 0.04538 | -1.539431634 | 29.71129 | 28.91063344 | 23.15318205 | 15.65480177 |
| NCRNA00120 | non-protein coding RNA 120 | 0.111583 | -1.539859667 | 50.28855 | 38.41265636 | 19.96149979 | 40.81201514 |
| C1orf43 | chromosome 1 open reading frame 43 | 0.007118 | -1.540255445 | 347.6454 | 395.0124945 | 256.2604594 | 225.881236 |
| TBC1D3C | TBC1 domain family. member 3C | 0.091816 | -1.540402466 | 53.92543 | 41.19220621 | 26.60796082 | 35.1826568 |
| SPG21 | spastic paraplegia 21 (autosomal recessive. Mast syndrome) | 0.008718 | -1.540556658 | 117.8826 | 118.38942 | 69.82788093 | 84.21283708 |
| PTP4A3 | protein tyrosine phosphatase type IVA. member 3 | 0.006118 | -1.542879552 | 234.5867 | 262.9416353 | 152.9341221 | 169.4315826 |
| GAR1 | GAR1 ribonucleoprotein homolog (yeast) | 0.003867 | -1.543031963 | 101.0059 | 102.7747263 | 71.52392733 | 60.95813846 |
| FAM21C | family with sequence similarity 21. member C | 0.061931 | -1.544331763 | 26.8366 | 38.20064451 | 23.01857109 | 18.67405557 |
| TBCK | TBC1 domain containing kinase | 0.010092 | -1.545045027 | 33.42374 | 38.15565998 | 26.81717417 | 19.92134695 |
| BSDC1 | BSD domain containing 1 | 0.009331 | -1.545386377 | 175.0456 | 238.5482755 | 129.7749046 | 134.7295029 |
| CDC25A | cell division cycle 25 homolog A (S. pombe) | 0.02366 | -1.545570883 | 49.82461 | 62.67005052 | 32.96142175 | 39.65703064 |
| TFEC | transcription factor EC | 0.008941 | -1.546503087 | 25.54103 | 31.75017665 | 18.31114714 | 18.5168633 |
| FEM1B | fem-1 homolog b (C. elegans) | 0.003994 | -1.547590384 | 38.34614 | 41.37621869 | 25.57630793 | 25.90136215 |
| SIPA1 | signal-induced proliferation-associated 1 | 0.007047 | -1.550829315 | 92.25583 | 91.37313177 | 68.18439233 | 51.40428742 |
| SLC3A2 | solute carrier family 3 (activators of dibasic and neutral amino acid transport). member 2 | 0.012534 | -1.551147616 | 284.5267 | 235.2436845 | 193.1698674 | 144.0108807 |
| ZC3H12A | zinc finger CCCH-type containing 12A | 0.007935 | -1.551429716 | 61.88761 | 60.94616287 | 45.04541142 | 34.78846979 |
| HP1BP3 | heterochromatin protein 1. binding protein 3 | 0.044039 | -1.551548646 | 71.69529 | 77.11770238 | 56.8668581 | 40.38821593 |
| CKAP5 | cytoskeleton associated protein 5 | 0.004634 | -1.552658553 | 69.39562 | 80.10110401 | 51.03219268 | 45.18293266 |
| ACTA2 | actin. alpha 2. smooth muscle. aorta | 0.006234 | -1.553333621 | 112.2485 | 110.8343973 | 71.37338008 | 72.24191224 |
| ATHL1 | ATH1. acid trehalase-like 1 (yeast) | 0.005305 | -1.553381504 | 61.40619 | 76.97288908 | 44.08013772 | 44.43761333 |
| IL1R2 | interleukin 1 receptor. type II | 0.236929 | -1.553595768 | 30.37141 | 25.75654682 | 31.10113639 | 10.42078071 |
| EIF4A1P4 | eukaryotic translation initiation factor 4A1 pseudogene 4 | 0.012516 | -1.553938164 | 1209.488 | 1201.028819 | 921.3010162 | 652.9588985 |
| SLC15A4 | solute carrier family 15. member 4 | 0.003519 | -1.556842997 | 350.4348 | 416.3858507 | 257.5331051 | 233.7655314 |
| ARPC1B | actin related protein 2/3 complex. subunit 1B. 41kDa | 0.009861 | -1.557024467 | 128.5322 | 165.3113349 | 98.35130374 | 89.11348416 |
| SGK1 | serum/glucocorticoid regulated kinase 1 | 0.006121 | -1.557652466 | 146.3298 | 144.7959949 | 89.8846463 | 97.1544628 |
| TRAF2 | TNF receptor-associated factor 2 | 0.010095 | -1.558518742 | 48.99151 | 58.53970779 | 33.44198944 | 35.30654155 |
| IPO7 | importin 7 | 0.003737 | -1.559640677 | 43.28783 | 50.67708966 | 29.6322343 | 30.434349 |
| PLS1 | plastin 1 | 0.008401 | -1.562647132 | 70.97064 | 53.01934863 | 37.62241189 | 40.95855501 |
| CCDC36 | coiled-coil domain containing 36 | 0.005667 | -1.565714287 | 23.60686 | 28.78266346 | 16.4771365 | 16.82141438 |
| ATP2A3 | ATPase. Ca++ transporting. ubiquitous | 0.009748 | -1.566126557 | 129.5507 | 113.1258247 | 66.40325137 | 89.98261498 |
| ANKDD1A | ankyrin repeat and death domain containing 1A | 0.006911 | -1.566453705 | 45.43052 | 58.49514035 | 30.47342001 | 35.5394871 |
| ZNF3 | zinc finger protein 3 | 0.13924 | -1.566826776 | 39.97074 | 37.77473757 | 38.45495838 | 15.99371557 |
| ARRB2 | arrestin. beta 2 | 0.019618 | -1.567566042 | 34.86586 | 27.85318239 | 23.50818084 | 16.81142489 |
| MAT2B | methionine adenosyltransferase II. beta | 0.001109 | -1.567863258 | 186.8358 | 184.5877865 | 120.4820653 | 116.4459382 |
| PEMT | phosphatidylethanolamine N-methyltransferase | 0.107616 | -1.568521906 | 31.33183 | 40.24481668 | 15.60536659 | 32.84282664 |
| CCL4L2 | chemokine (C-C motif) ligand 4-like 2 | 0.00506 | -1.570405598 | 1998.66 | 2234.123249 | 1200.401189 | 1508.328417 |
| SON | SON DNA binding protein | 0.008968 | -1.57244035 | 92.2896 | 100.7615336 | 52.82593 | 71.19541143 |
| USP48 | ubiquitin specific peptidase 48 | 0.004386 | -1.573217328 | 48.90076 | 41.86441383 | 31.65512388 | 26.12998671 |
| LOC100132106 | hypothetical LOC100132106 | 0.023458 | -1.574191254 | 47.9279 | 54.33389946 | 29.94223851 | 35.0961917 |
| PPHLN1 | periphilin 1 | 0.008842 | -1.576594707 | 31.52235 | 27.79443491 | 16.32028563 | 21.5977555 |
| FBXL17 | F-box and leucine-rich repeat protein 17 | 0.020966 | -1.578678806 | 23.27362 | 35.97046832 | 17.38806818 | 19.31839771 |
| ZXDA | zinc finger. X-linked. duplicated A | 0.001405 | -1.579268941 | 27.4718 | 30.5988108 | 18.27789257 | 18.43967761 |
| BCL7A | B-cell CLL/lymphoma 7A | 0.003824 | -1.580579203 | 107.3588 | 128.6221248 | 79.2111822 | 69.780508 |
| LOC728728 | similar to hCG1779533 | 0.032374 | -1.581346624 | 51.98256 | 61.47816519 | 30.40036228 | 42.03834515 |
| CSNK2A1 | casein kinase 2. alpha 1 polypeptide | 0.0018 | -1.581464922 | 45.15058 | 46.0388102 | 27.40503873 | 30.32758903 |
| VPS39 | vacuolar protein sorting 39 homolog (S. cerevisiae) | 0.002289 | -1.584422098 | 118.4578 | 130.3468956 | 82.40683476 | 74.63787309 |
| DYRK2 | dual-specificity tyrosine-(Y)-phosphorylation regulated kinase 2 | 0.00301 | -1.584451298 | 24.14235 | 29.26538868 | 16.32815322 | 17.23609571 |
| EPB41 | erythrocyte membrane protein band 4.1 (elliptocytosis 1. RH-linked) | 0.064174 | -1.584867502 | 62.71567 | 43.15805263 | 43.65287499 | 24.6853415 |
| NDRG3 | NDRG family member 3 | 0.009836 | -1.585975191 | 140.3773 | 114.419903 | 78.42122415 | 81.42770239 |
| APOE | apolipoprotein E | 0.039924 | -1.585986192 | 36.70524 | 32.8755032 | 27.39435885 | 17.5121927 |
| SMPDL3B | sphingomyelin phosphodiesterase. acid-like 3B | 0.044296 | -1.586348827 | 27.51054 | 27.85763436 | 18.04736441 | 16.87454989 |
| CLSTN1 | calsyntenin 1 | 0.001711 | -1.588016686 | 39.62019 | 38.11984138 | 25.16378463 | 23.80025281 |
| TPM3 | tropomyosin 3 | 0.001281 | -1.589149416 | 435.6482 | 429.1594792 | 281.2758558 | 263.204136 |
| U2AF2 | U2 small nuclear RNA auxiliary factor 2 | 0.004496 | -1.590126327 | 58.1856 | 46.44279023 | 30.51628376 | 35.02183621 |
| PKM2 | pyruvate kinase. muscle | 0.002354 | -1.591759141 | 165.8261 | 172.4654361 | 116.1323714 | 97.19566323 |
| SGSM3 | small G protein signaling modulator 3 | 0.007839 | -1.592735189 | 56.77131 | 56.73072645 | 39.48899323 | 32.15022699 |
| DDR1 | discoidin domain receptor tyrosine kinase 1 | 0.025858 | -1.592775789 | 30.46137 | 47.85321251 | 26.73928329 | 21.48827179 |
| TAP2 | transporter 2. ATP-binding cassette. sub-family B (MDR/TAP) | 0.080166 | -1.593350407 | 36.32224 | 23.87574574 | 21.64913979 | 15.77852431 |
| MARCH3 | membrane-associated ring finger (C3HC4) 3 | 0.022808 | -1.596316994 | 66.46967 | 68.00396085 | 33.38174511 | 53.13864211 |
| ZNF155 | zinc finger protein 155 | 0.068347 | -1.598519042 | 29.62024 | 33.92624833 | 24.30691302 | 16.17927129 |
| SUV420H1 | suppressor of variegation 4-20 homolog 1 (Drosophila) | 0.00493 | -1.600241417 | 188.0052 | 165.7631931 | 107.5784433 | 113.1258247 |
| LOC100133076 | similar to hCG1984118 | 0.08668 | -1.601201643 | 33.75082 | 46.95233208 | 29.55794416 | 20.91101711 |
| KAT5 | K(lysine) acetyltransferase 5 | 0.00735 | -1.605712809 | 39.60835 | 37.98722171 | 24.27267189 | 24.0420228 |
| SMAP2 | small ArfGAP2 | 0.017237 | -1.606433567 | 297.1144 | 459.6155468 | 218.5462321 | 242.1305242 |
| RECQL4 | RecQ protein-like 4 | 0.022329 | -1.607027143 | 77.93235 | 49.88243208 | 35.3627012 | 42.56705012 |
| SRGAP2 | SLIT-ROBO Rho GTPase activating protein 2 | 0.008099 | -1.607318916 | 20.72077 | 28.56313113 | 14.91205165 | 15.36279364 |
| PICALM | phosphatidylinositol binding clathrin assembly protein | 0.016345 | -1.607391811 | 58.90257 | 85.63127653 | 49.3892985 | 39.52667819 |
| SLC10A7 | solute carrier family 10 (sodium/bile acid cotransporter family). member 7 | 0.002127 | -1.608851168 | 39.83349 | 36.5575288 | 25.29421407 | 22.24191801 |
| APOBEC3F | apolipoprotein B mRNA editing enzyme. catalytic polypeptide-like 3F | 0.051868 | -1.609296296 | 29.74171 | 30.71431605 | 16.75467842 | 21.05225582 |
| NACC1 | nucleus accumbens associated 1. BEN and BTB (POZ) domain containing | 0.015293 | -1.611767637 | 30.58522 | 25.46152247 | 14.32455654 | 20.9271393 |
| SDCBP | syndecan binding protein (syntenin) | 0.005957 | -1.611781602 | 198.0398 | 253.0459965 | 152.898191 | 126.1645218 |
| CCT8L1 | chaperonin containing TCP1. subunit 8 (theta)-like 1 | 0.039926 | -1.611832026 | 25.35374 | 33.32918845 | 16.11181105 | 20.18751742 |
| NIPA2 | non imprinted in Prader-Willi/Angelman syndrome 2 | 0.002156 | -1.61466928 | 162.1531 | 133.4161179 | 89.81085293 | 92.39269203 |
| LOC728288 | hypothetical LOC728288 | 0.047666 | -1.61890971 | 42.49348 | 28.72969535 | 16.67910319 | 27.92771042 |
| CTSS | cathepsin S | 0.124446 | -1.619418482 | 50.07271 | 50.47352515 | 19.44642114 | 49.55721116 |
| TREML2 | triggering receptor expressed on myeloid cells-like 2 | 0.012215 | -1.62319646 | 34.54979 | 35.53705583 | 17.55906294 | 26.53892369 |
| PCSK4 | proprotein convertase subtilisin/kexin type 4 | 0.070921 | -1.623286394 | 29.58877 | 32.13392711 | 13.09462082 | 27.55544909 |
| CHI3L2 | chitinase 3-like 2 | 0.011727 | -1.624315905 | 116.9263 | 139.8454376 | 65.64926131 | 94.40385395 |
| C3orf52 | chromosome 3 open reading frame 52 | 0.004254 | -1.624823828 | 31.78661 | 31.56963054 | 22.44092428 | 16.93793503 |
| SUSD5 | sushi domain containing 5 | 0.018685 | -1.62560394 | 29.33246 | 41.56660896 | 18.9778455 | 24.31177873 |
| LOC344593 | protein tyrosine phosphatase. non-receptor type 11 pseudogene | 0.07188 | -1.627810171 | 28.29622 | 38.06404263 | 28.61208277 | 14.20647553 |
| HVCN1 | hydrogen voltage-gated channel 1 | 0.049572 | -1.6285139 | 60.33223 | 72.03053577 | 55.75836751 | 29.38820271 |
| C17orf56 | chromosome 17 open reading frame 56 | 0.006183 | -1.628586394 | 61.82578 | 53.72637098 | 41.00510481 | 30.5420099 |
| ERCC8 | excision repair cross-complementing rodent repair deficiency. complementation group 8 | 0.035796 | -1.631402835 | 51.17117 | 49.74395004 | 24.59955039 | 38.87908304 |
| UBE2I | ubiquitin-conjugating enzyme E2I (UBC9 homolog. yeast) | 0.005137 | -1.631618605 | 52.16322 | 58.89007874 | 29.53683274 | 39.06657338 |
| NSF | N-ethylmaleimide-sensitive factor | 0.053812 | -1.632864353 | 37.77474 | 20.70608846 | 20.21452022 | 14.51228728 |
| CYTIP | cytohesin 1 interacting protein | 0.004577 | -1.638437578 | 52.03184 | 39.27684118 | 28.32841313 | 26.87346673 |
| ATE1 | arginyltransferase 1 | 0.039037 | -1.638471144 | 36.02742 | 35.05965913 | 16.41264803 | 28.66715691 |
| RACGAP1 | Rac GTPase activating protein 1 | 0.00926 | -1.638494232 | 34.4141 | 37.40101909 | 26.44490389 | 18.12959356 |
| IFFO1 | intermediate filament family orphan 1 | 0.005633 | -1.63933099 | 31.10292 | 30.26352618 | 20.59584885 | 17.00621412 |
| VPS41 | vacuolar protein sorting 41 homolog (S. cerevisiae) | 0.000655 | -1.640116045 | 66.51212 | 64.76437857 | 40.16849428 | 39.8659642 |
| SRSF2IP | serine/arginine-rich splicing factor 2. interacting protein | 0.038054 | -1.640767975 | 27.87363 | 39.00432219 | 18.67599612 | 21.62360365 |
| SLC39A9 | solute carrier family 39 (zinc transporter). member 9 | 0.004588 | -1.641568221 | 107.6241 | 85.81419717 | 56.4729613 | 60.68902363 |
| TNFRSF18 | tumor necrosis factor receptor superfamily. member 18 | 0.004987 | -1.641989769 | 61.57869 | 81.1417528 | 46.1646106 | 40.14437913 |
| OTUD5 | OTU domain containing 5 | 0.056745 | -1.642305776 | 79.58821 | 113.053332 | 47.59437276 | 70.09199838 |
| PSMC4 | proteasome (prosome. macropain) 26S subunit. ATPase. 4 | 0.000772 | -1.64265042 | 438.3275 | 444.2602929 | 270.7326122 | 266.5663607 |
| PPIL2 | peptidylprolyl isomerase (cyclophilin)-like 2 | 0.001045 | -1.643036884 | 30.03567 | 31.94427526 | 18.40421527 | 19.31160597 |
| DIDO1 | death inducer-obliterator 1 | 0.002998 | -1.644103327 | 35.53462 | 40.08885901 | 25.16173494 | 20.94481986 |
| LOC645434 | hypothetical LOC645434 | 0.018632 | -1.644621081 | 20.10243 | 30.51252686 | 17.02411859 | 13.32080016 |
| COMT | catechol-O-methyltransferase | 0.013202 | -1.644784057 | 124.2097 | 171.4744229 | 96.79394936 | 81.3371343 |
| TGM2 | transglutaminase 2 (C polypeptide. protein-glutamine-gamma-glutamyltransferase) | 0.002593 | -1.645366732 | 74.65034 | 84.39139186 | 44.34833178 | 52.47195198 |
| HSPD1 | heat shock 60kDa protein 1 (chaperonin) | 0.014185 | -1.646745545 | 72.17346 | 97.33444449 | 54.91199036 | 47.17630305 |
| PQLC2 | PQ loop repeat containing 2 | 0.009732 | -1.648776329 | 26.25859 | 37.23485735 | 17.45733508 | 20.60248516 |
| NOL4 | nucleolar protein 4 | 0.001994 | -1.649168179 | 27.44043 | 24.67766366 | 17.31334808 | 14.38083137 |
| SIT1 | signaling threshold regulating transmembrane adaptor 1 | 0.006232 | -1.652668351 | 57.80728 | 55.67632529 | 31.32095981 | 37.62241189 |
| SHPRH | SNF2 histone linker PHD RING helicase | 0.024584 | -1.654464701 | 21.3894 | 30.28455381 | 17.05500459 | 13.8756346 |
| KLHL4 | kelch-like 4 (Drosophila) | 0.024872 | -1.656605558 | 57.67624 | 56.33782615 | 29.64079036 | 39.94560405 |
| HNRNPA3 | heterogeneous nuclear ribonucleoprotein A3 | 0.026426 | -1.661459236 | 187.2965 | 324.175819 | 146.5241215 | 150.1141968 |
| PDIA3P | protein disulfide isomerase family A. member 3 pseudogene | 0.000656 | -1.666127602 | 37.98722 | 38.8483868 | 23.58620021 | 22.5390715 |
| SEZ6L2 | seizure related 6 homolog (mouse)-like 2 | 0.006734 | -1.667772202 | 39.48346 | 53.44839492 | 30.05814535 | 25.24144797 |
| ENSA | endosulfine alpha | 0.00932 | -1.669024836 | 307.6254 | 384.067139 | 238.391728 | 177.9150243 |
| ZGPAT | zinc finger. CCCH-type with G patch domain | 0.004881 | -1.670188241 | 30.31351 | 40.86100798 | 20.34785066 | 21.82206677 |
| EIF4EBP3 | eukaryotic translation initiation factor 4E binding protein 3 | 0.083873 | -1.670427239 | 84.78745 | 55.95366438 | 59.88225299 | 28.3927088 |
| MCCC2 | methylcrotonoyl-CoA carboxylase 2 (beta) | 0.00839 | -1.672986817 | 66.61774 | 84.78744591 | 38.90359713 | 51.8736302 |
| NPSR1 | neuropeptide S receptor 1 | 0.006544 | -1.673714102 | 24.87341 | 35.06629439 | 17.99389266 | 17.30363909 |
| ARMC5 | armadillo repeat containing 5 | 0.026105 | -1.674135635 | 22.58782 | 38.71991753 | 19.54380101 | 15.96682125 |
| SDCCAG3 | serologically defined colon cancer antigen 3 | 0.032719 | -1.675022054 | 39.16608 | 32.42385727 | 17.05287986 | 26.54214099 |
| ZFX | zinc finger protein. X-linked | 0.006082 | -1.67726225 | 77.00434 | 59.46103361 | 36.99252295 | 43.99787903 |
| POTEE | POTE ankyrin domain family. member E | 0.006744 | -1.678471736 | 25.89464 | 36.78256869 | 19.4585693 | 17.37452415 |
| EGR1 | early growth response 1 | 0.001317 | -1.679789185 | 107.8388 | 119.2086598 | 68.60105858 | 66.41135287 |
| DNAJB12 | DnaJ (Hsp40) homolog. subfamily B. member 12 | 0.028688 | -1.680107029 | 25.37321 | 44.66923063 | 18.60822598 | 21.57769114 |
| EIF4G2 | eukaryotic translation initiation factor 4 gamma. 2 | 0.000509 | -1.682183402 | 463.706 | 466.5307497 | 273.2672336 | 279.7619906 |
| TRUB1 | TruB pseudouridine (psi) synthase homolog 1 (E. coli) | 0.02791 | -1.682724412 | 66.19362 | 68.23623118 | 30.20329602 | 52.81421552 |
| ELF2 | E74-like factor 2 (ets domain transcription factor) | 0.004196 | -1.68318138 | 27.25512 | 30.80988843 | 17.29990846 | 17.13296732 |
| CMTM3 | CKLF-like MARVEL transmembrane domain containing 3 | 0.004041 | -1.68837341 | 37.64014 | 30.22947557 | 20.9923362 | 19.01446143 |
| TRIM9 | tripartite motif-containing 9 | 0.008457 | -1.688533561 | 25.47792 | 30.90242673 | 19.39863153 | 14.2352794 |
| RNF216L | ring finger protein 216-like | 0.010402 | -1.692927366 | 24.27789 | 37.74782669 | 17.93623868 | 17.82768285 |
| LSAMP | limbic system-associated membrane protein | 0.001628 | -1.694205074 | 28.37533 | 29.31034464 | 19.00372981 | 15.24723251 |
| ZBED3 | zinc finger. BED-type containing 3 | 0.022393 | -1.695900364 | 40.62876 | 71.08531892 | 31.30280737 | 32.079646 |
| EIF5 | eukaryotic translation initiation factor 5 | 0.003178 | -1.697316063 | 31.4729 | 23.31607783 | 16.03548888 | 15.88490218 |
| VWA1 | von Willebrand factor A domain containing 1 | 0.019378 | -1.700347882 | 20.38389 | 33.1766818 | 13.54661981 | 17.26686282 |
| SH3BP5 | SH3-domain binding protein 5 (BTK-associated) | 0.067167 | -1.701332009 | 17.75104 | 39.8254998 | 14.8235911 | 16.47606036 |
| ABHD8 | abhydrolase domain containing 8 | 0.003948 | -1.703653469 | 69.94317 | 65.21971718 | 34.36540588 | 45.73410567 |
| PPAP2C | phosphatidic acid phosphatase type 2C | 0.003773 | -1.703688423 | 35.3364 | 46.06754739 | 25.61715848 | 21.89304361 |
| HNRNPA1L2 | heterogeneous nuclear ribonucleoprotein A1-like 2 | 0.002173 | -1.709788869 | 104.8525 | 108.3715956 | 70.28607792 | 55.30187549 |
| MRPL54 | mitochondrial ribosomal protein L54 | 0.001103 | -1.710183114 | 846.7861 | 842.7943911 | 482.1947874 | 506.0432439 |
| SNORD89 | small nucleolar RNA. C/D box 89 | 0.012076 | -1.711261465 | 61.38782 | 56.70517505 | 27.28538011 | 43.56544304 |
| ISLR | immunoglobulin superfamily containing leucine-rich repeat | 0.038224 | -1.711778264 | 18.5918 | 32.76121799 | 13.36060014 | 15.55823981 |
| ZNF341 | zinc finger protein 341 | 0.001198 | -1.711977421 | 35.43381 | 41.4138629 | 22.343865 | 22.40831363 |
| SLC30A6 | solute carrier family 30 (zinc transporter). member 6 | 0.011446 | -1.712895398 | 31.71016 | 32.7367229 | 23.50425247 | 15.0530857 |
| LOC727848 | similar to actin-like protein | 0.00494 | -1.712908959 | 42.31452 | 34.64167519 | 22.18558156 | 22.5189923 |
| CCM2 | cerebral cavernous malformation 2 | 0.000723 | -1.71520637 | 59.39636 | 55.5360688 | 35.64835687 | 31.45304241 |
| SNRNP35 | small nuclear ribonucleoprotein 35kDa (U11/U12) | 0.014708 | -1.716194811 | 61.52676 | 78.25967137 | 50.61772566 | 32.29730167 |
| LOC100131733 | hypothetical LOC100131733 | 0.013286 | -1.725527618 | 36.13994 | 40.40109362 | 17.77585374 | 27.58711072 |
| PLD4 | phospholipase D family. member 4 | 0.038033 | -1.726468703 | 37.92641 | 32.61682974 | 15.35861289 | 27.02178541 |
| MATR3 | matrin 3 | 0.003648 | -1.726534355 | 83.36219 | 62.9582208 | 41.51855783 | 42.40615152 |
| UBE2E1 | ubiquitin-conjugating enzyme E2E 1 (UBC4/5 homolog. yeast) | 0.000689 | -1.727400326 | 201.048 | 218.6342435 | 115.2761219 | 127.7887369 |
| TXNDC5 | thioredoxin domain containing 5 (endoplasmic reticulum) | 0.001605 | -1.728558932 | 66.10703 | 65.87697759 | 33.88876957 | 43.0087808 |
| TSC22D3 | TSC22 domain family. member 3 | 0.00071 | -1.732309906 | 252.5379 | 228.0772885 | 130.7847421 | 146.7575381 |
| GABRE | gamma-aminobutyric acid (GABA) A receptor. epsilon | 0.050021 | -1.733900235 | 43.6771 | 32.78343447 | 25.62806639 | 18.5842132 |
| CSAD | cysteine sulfinic acid decarboxylase | 0.006717 | -1.737721397 | 26.02226 | 33.19295971 | 20.01062087 | 14.29453706 |
| MAPK14 | mitogen-activated protein kinase 14 | 0.011583 | -1.744838192 | 25.78686 | 34.80666376 | 14.19753255 | 20.76527579 |
| UGT2B4 | UDP glucuronosyltransferase 2 family. polypeptide B4 | 0.002959 | -1.74939643 | 33.03567 | 30.414777 | 19.4721266 | 16.86079014 |
| GALNT2 | UDP-N-acetyl-alpha-D-galactosamine:polypeptide N-acetylgalactosaminyltransferase 2 (GalNAc-T2) | 0.025676 | -1.757800566 | 70.49419 | 86.45668786 | 58.33190097 | 33.8147826 |
| PTCH1 | patched 1 | 0.034441 | -1.763595435 | 28.87045 | 42.53832841 | 15.90707027 | 24.82251619 |
| HNRNPM | heterogeneous nuclear ribonucleoprotein M | 0.014157 | -1.766152456 | 24.582 | 41.58250148 | 17.13810539 | 19.12091657 |
| TAF1A | TATA box binding protein (TBP)-associated factor. RNA polymerase I. A. 48kDa | 0.009424 | -1.767020355 | 26.52721 | 34.1531376 | 15.19491304 | 19.09593528 |
| KHK | ketohexokinase (fructokinase) | 0.008536 | -1.767699146 | 25.24447 | 34.84815286 | 17.37497018 | 16.20336654 |
| ACTR3B | ARP3 actin-related protein 3 homolog B (yeast) | 0.015847 | -1.779679945 | 76.13373 | 67.35196208 | 53.0764418 | 30.50294149 |
| CLIC4 | chloride intracellular channel 4 | 0.006729 | -1.790066879 | 133.5043 | 157.7010401 | 71.46152919 | 91.94309963 |
| LOC730167 | similar to protein tyrosine phosphatase 4a1 | 0.003062 | -1.798368455 | 97.85811 | 93.7530145 | 46.02746338 | 61.63219944 |
| RNF146 | ring finger protein 146 | 0.000351 | -1.799606562 | 142.2307 | 129.0204615 | 73.94115554 | 76.63206466 |
| ZNHIT2 | zinc finger. HIT type 2 | 0.001953 | -1.806432469 | 55.40917 | 66.21048712 | 37.85227881 | 29.70112748 |
| RABL3 | RAB. member of RAS oncogene family-like 3 | 0.001356 | -1.810873521 | 96.6269 | 109.860635 | 64.10842334 | 50.49505199 |
| YWHAZ | tyrosine 3-monooxygenase/tryptophan 5-monooxygenase activation protein. zeta polypeptide | 0.000259 | -1.814165887 | 297.9231 | 289.6106403 | 162.3555769 | 161.472077 |
| ARPC2 | actin related protein 2/3 complex. subunit 2. 34kDa | 0.018408 | -1.815368452 | 44.30328 | 29.14979967 | 16.57477558 | 23.64254435 |
| BIRC3 | baculoviral IAP repeat-containing 3 | 0.00089 | -1.823292289 | 74.41011 | 61.06028958 | 39.10571033 | 34.94926565 |
| MAX | MYC associated factor X | 0.001542 | -1.825301942 | 41.75206 | 54.96488995 | 26.14168079 | 26.34877079 |
| TPRKB | TP53RK binding protein | 0.006463 | -1.833026321 | 32.2973 | 36.08752692 | 14.84294288 | 23.37038906 |
| P4HA2 | prolyl 4-hydroxylase. alpha polypeptide II | 0.055979 | -1.846307361 | 50.44185 | 51.77492917 | 30.27280949 | 25.30753359 |
| PSCA | prostate stem cell antigen | 0.014919 | -1.853433984 | 33.15722 | 29.76389592 | 12.58568514 | 22.82636702 |
| LOC644063 | heterogeneous nuclear ribonucleoprotein K pseudogene | 0.001007 | -1.886825704 | 1299.987 | 1149.25755 | 647.0144759 | 648.6026231 |
| RALGPS2 | Ral GEF with PH domain and SH3 binding motif 2 | 0.001703 | -1.898436207 | 90.71895 | 91.94309963 | 44.55759096 | 51.94017712 |
| PHF1 | PHD finger protein 1 | 0.000194 | -1.949027419 | 58.84449 | 52.44748926 | 27.94897773 | 29.06890756 |
| HSPA6 | heat shock 70kDa protein 6 (HSP70B') | 0.017476 | -1.949515335 | 38.64547 | 59.03510686 | 30.98107015 | 19.37577899 |
| MPHOSPH6 | M-phase phosphoprotein 6 | 0.031347 | -1.950244591 | 46.81609 | 39.25650741 | 14.69437972 | 32.88344832 |
| SCAF1 | SR-related CTD-associated factor 1 | 0.000229 | -1.960472517 | 36.82068 | 36.83723562 | 17.37351107 | 20.31279429 |
| VNN2 | vanin 2 | 0.000253 | -1.974930197 | 79.8546 | 79.33888989 | 38.2973432 | 42.41445436 |
| HNRNPK | heterogeneous nuclear ribonucleoprotein K | 0.007812 | -2.002896859 | 372.61 | 397.2051607 | 255.1941822 | 144.5710797 |
| CLSTN1 | calsyntenin 1 | 0.019883 | -2.005010687 | 44.80064 | 47.21508258 | 33.21062891 | 15.84362155 |
| TSPAN14 | tetraspanin 14 | 0.002413 | -2.048145697 | 43.21856 | 57.33543245 | 21.83573216 | 27.05228621 |
| LBR | lamin B receptor | 0.00019 | -2.063239788 | 254.5547 | 260.5862426 | 123.9083321 | 125.7571557 |
| GTF2IP1 | general transcription factor IIi. pseudogene 1 | 0.002895 | -2.063408646 | 162.868 | 202.416452 | 106.2575437 | 72.87040669 |
| CLK2P | CDC-like kinase 2. pseudogene | 0.000723 | -2.070043938 | 33.21063 | 45.60645487 | 18.92375687 | 18.67828197 |
| PAX3 | paired box 3 | 0.002001 | -2.074427367 | 31.68555 | 33.63131113 | 19.5393504 | 12.67355698 |
| IFI44L | interferon-induced protein 44-like | 0.044805 | -2.091226171 | 214.4449 | 404.4736379 | 94.11468263 | 210.7397629 |
| STK24 | serine/threonine kinase 24 | 0.001556 | -2.113321933 | 69.47282 | 77.95817283 | 37.67878039 | 32.18462252 |
| EIF3CL | eukaryotic translation initiation factor 3. subunit C-like | 0.00013 | -2.373803542 | 161.6052 | 185.4153 | 81.05407947 | 65.60492891 |
