# Supplementary material for: Discovery of Compound A – a selective activator of the glucocorticoid receptor with anti-inflammatory and anti-cancer activity
Source: Oncotarget. 2015 Oct 2;6(31):30730–44. doi: 10.18632/oncotarget.5078 (PMC4741564; doi:10.18632/oncotarget.5078)
Supplement: Supplementary file 3 [file oncotarget-06-30730-s003.docx]

| **Symbol** | **Description** | **FoldChange** | **P.value** | **Control - 1** | **Control - 2** | **Dex. 10uM. 8h - 1** | **Dex. 10uM. 8h - 2** |
| --- | --- | --- | --- | --- | --- | --- | --- |
| TIPARP | TCDD-inducible poly(ADP-ribose) polymerase | 6.5909 | 8.15E-13 | 498.47 | 453.08 | 2937.9 | 3339.3 |
| PNLIP | pancreatic lipase | 5.9801 | 2.48E-12 | 638.07 | 518.49 | 3272 | 3615.8 |
| ING1 | inhibitor of growth family. member 1 | 5.4612 | 1.28E-10 | 632.58 | 497.54 | 3094.5 | 3033.3 |
| RASD1 | RAS. dexamethasone-induced 1 | 5.1986 | 6.45E-11 | 569.08 | 475.26 | 2560 | 2855.2 |
| CHST3 | carbohydrate (chondroitin 6) sulfotransferase 3 | 4.5361 | 7.17E-13 | 168.33 | 171.11 | 842.5 | 703.45 |
| COL6A1 | collagen. type VI. alpha 1 | 4.3171 | 1.18E-11 | 804.95 | 758.26 | 3084.3 | 3688.2 |
| TRIM48 | tripartite motif-containing 48 | 4.0585 | 9.95E-12 | 432.97 | 374.05 | 1611.5 | 1655.4 |
| SPRYD5 | SPRY domain containing 5 | 3.9335 | 2.41E-11 | 2100.1 | 1654.6 | 7298.6 | 7366.4 |
| PGC | progastricsin (pepsinogen C) | 3.6916 | 4.99E-11 | 530.87 | 456.57 | 1857.3 | 1778.5 |
| SRD5A1 | steroid-5-alpha-reductase. alpha polypeptide 1 (3-oxo-5 alpha-steroid delta 4-dehydrogenase alpha 1) | 3.6342 | 2.22E-12 | 381.3 | 361.6 | 1439.3 | 1265.2 |
| TAF5L | TAF5-like RNA polymerase II. p300/CBP-associated factor (PCAF)-associated factor. 65kDa | 3.5972 | 2.10E-13 | 309.43 | 331.77 | 1100.9 | 1206.7 |
| TUBA3E | tubulin. alpha 3e | 3.5388 | 3.16E-12 | 585.22 | 632.4 | 2147 | 2158.6 |
| PTGER4 | prostaglandin E receptor 4 (subtype EP4) | 3.4176 | 1.06E-10 | 275.13 | 254 | 857.41 | 952 |
| FKBP5 | FK506 binding protein 5 | 3.4008 | 5.45E-10 | 1547.2 | 1332 | 4736.2 | 5032.5 |
| CTGF | connective tissue growth factor | 3.2108 | 4.10E-10 | 159.44 | 145.72 | 458.41 | 522.51 |
| AZGP1 | alpha-2-glycoprotein 1. zinc-binding | 3.2061 | 1.66E-08 | 158.21 | 153.5 | 484.6 | 515.14 |
| PQLC1 | PQ loop repeat containing 1 | 2.9148 | 3.58E-12 | 1773.4 | 1818.2 | 5185.2 | 5283 |
| SGK | serum/glucocorticoid regulated kinase | 2.8761 | 6.27E-08 | 4135.9 | 3104.1 | 10937 | 9709.5 |
| OGFRL1 | opioid growth factor receptor-like 1 | 2.867 | 4.48E-11 | 204.87 | 206.65 | 607.45 | 572.85 |
| FLJ20160 | FLJ20160 protein | 2.8097 | 4.84E-10 | 984.74 | 913.79 | 2621.6 | 2709.7 |
| GNMT | glycine N-methyltransferase | 2.7892 | 3.72E-10 | 249.67 | 244.22 | 711.7 | 666.52 |
| NSDHL | NAD(P) dependent steroid dehydrogenase-like | 2.7647 | 1.75E-10 | 957.91 | 850.42 | 2377.7 | 2618.8 |
| STOM | stomatin | 2.732 | 2.55E-11 | 134.3 | 126.83 | 345.22 | 368.24 |
| FAM104A | family with sequence similarity 104. member A | 2.6595 | 1.63E-06 | 694.23 | 726.45 | 1663.3 | 2144.4 |
| SLC39A14 | solute carrier family 39 (zinc transporter). member 14 | 2.6251 | 8.64E-11 | 547.61 | 517.79 | 1408.5 | 1387.2 |
| CRISPLD2 | cysteine-rich secretory protein LCCL domain containing 2 | 2.6095 | 4.59E-09 | 245.81 | 234.22 | 672.76 | 582.73 |
| TUBA3C | tubulin. alpha 3c | 2.5773 | 1.56E-08 | 222.47 | 241.86 | 542.21 | 659.21 |
| LONRF1 | LON peptidase N-terminal domain and ring finger 1 | 2.571 | 1.85E-07 | 486.36 | 404.5 | 1272.4 | 1022 |
| ACSL1 | acyl-CoA synthetase long-chain family member 1 | 2.5228 | 0.00233 | 1452.1 | 1976.8 | 3123.1 | 5849.8 |
| PRIC285 | peroxisomal proliferator-activated receptor A interacting complex 285 | 2.5082 | 4.82E-09 | 624.68 | 615.97 | 1555.1 | 1556.6 |
| TUBA3D | tubulin. alpha 3d | 2.5034 | 1.99E-11 | 476.92 | 438.61 | 1121.5 | 1168.9 |
| RHOB | ras homolog gene family. member B | 2.4615 | 0.00095 | 671.68 | 872.92 | 1447.5 | 2454.3 |
| ZNF26 | zinc finger protein 26 | 2.4295 | 3.66E-08 | 252.98 | 228.8 | 565.58 | 604.03 |
| HPCAL1 | hippocalcin-like 1 | 2.4294 | 3.14E-11 | 1775.1 | 1629.5 | 4261.6 | 4005.9 |
| ZNF18 | zinc finger protein 18 | 2.4181 | 8.37E-11 | 408.56 | 369.23 | 894.15 | 986.49 |
| ZNF189 | zinc finger protein 189 | 2.3939 | 4.96E-07 | 695.77 | 552.84 | 1432.9 | 1538.4 |
| S100P | S100 calcium binding protein P | 2.3641 | 2.22E-07 | 9765.9 | 7606.5 | 20751 | 20007 |
| ATAD2 | ATPase family. AAA domain containing 2 | 2.3614 | 5.33E-07 | 555.3 | 461.54 | 1252 | 1141.5 |
| NET1 | neuroepithelial cell transforming gene 1 | 2.3608 | 8.61E-11 | 607.17 | 645.26 | 1499.4 | 1456.3 |
| FOXO1 | forkhead box O1 | 2.3597 | 5.12E-09 | 467.99 | 437.6 | 1081.4 | 1054.5 |
| COL6A2 | collagen. type VI. alpha 2 | 2.2962 | 4.79E-10 | 183.92 | 178.43 | 415.38 | 416.55 |
| ST3GAL4 | ST3 beta-galactoside alpha-2.3-sialyltransferase 4 | 2.293 | 2.21E-08 | 457.86 | 425.57 | 995.89 | 1028.7 |
| CDC20 | cell division cycle 20 homolog (S. cerevisiae) | 2.2876 | 0.00016 | 1409.5 | 989.92 | 2737 | 2667.7 |
| TSPYL2 | TSPY-like 2 | 2.2749 | 3.05E-09 | 309.04 | 285.49 | 634.83 | 719.23 |
| ATP1A1 | ATPase. Na+/K+ transporting. alpha 1 polypeptide | 2.271 | 2.24E-09 | 1324.8 | 1240.3 | 3011 | 2814.5 |
| RAB20 | RAB20. member RAS oncogene family | 2.2592 | 2.20E-09 | 2134.7 | 1935.5 | 4784.2 | 4407.6 |
| HK2 | hexokinase 2 | 2.2537 | 7.91E-10 | 256.07 | 243.82 | 587.56 | 539.71 |
| TMEM56 | transmembrane protein 56 | 2.2472 | 5.19E-09 | 308.17 | 283.77 | 685.02 | 644.67 |
| CDC42EP4 | CDC42 effector protein (Rho GTPase binding) 4 | 2.2255 | 2.60E-09 | 1534.5 | 1483.5 | 3364.7 | 3351 |
| CNKSR3 | CNKSR family member 3 | 2.2041 | 5.83E-07 | 395.62 | 341.05 | 838.85 | 781.38 |
| ELK1 | ELK1. member of ETS oncogene family | 2.2034 | 7.67E-11 | 672.48 | 751.08 | 1536.7 | 1595.8 |
| PLOD2 | procollagen-lysine. 2-oxoglutarate 5-dioxygenase 2 | 2.1686 | 3.80E-05 | 263.65 | 160.13 | 440.19 | 451.09 |
| OSGIN1 | oxidative stress induced growth inhibitor 1 | 2.131 | 8.23E-10 | 598.77 | 577.4 | 1338.7 | 1172.7 |
| UBE2C | ubiquitin-conjugating enzyme E2C | 2.1207 | 6.86E-07 | 367.19 | 329.66 | 689.13 | 789.97 |
| SLC25A18 | solute carrier family 25 (mitochondrial carrier). member 18 | 2.1053 | 6.56E-11 | 142.22 | 134.11 | 295.56 | 286.02 |
| WIPI1 | WD repeat domain. phosphoinositide interacting 1 | 2.1006 | 1.35E-09 | 260.46 | 239.04 | 518.92 | 529.41 |
| HMGB2 | high-mobility group box 2 | 2.0787 | 5.50E-06 | 313.23 | 261.02 | 641.68 | 550.55 |
| GHR | growth hormone receptor | 2.0752 | 2.17E-06 | 725.51 | 796.88 | 1413.1 | 1761.9 |
| FMO5 | flavin containing monooxygenase 5 | 2.0522 | 1.03E-10 | 156.56 | 159.16 | 324.37 | 323.52 |
| IQGAP3 | IQ motif containing GTPase activating protein 3 | 2.0443 | 0.00026 | 558.79 | 412.63 | 1006 | 957.91 |
| FAM90A1 | family with sequence similarity 90. member A1 | 2.0346 | 8.48E-08 | 134.42 | 132.64 | 249.22 | 296.16 |
| PHACTR3 | phosphatase and actin regulator 3 | 2.0334 | 6.92E-10 | 168.25 | 159.99 | 331.87 | 335.37 |
| TMEM43 | transmembrane protein 43 | 2.029 | 1.41E-09 | 1228.9 | 1185.1 | 2513.3 | 2385.5 |
| GADD45B | growth arrest and DNA-damage-inducible. beta | 2.0127 | 2.96E-09 | 190.81 | 198.52 | 389.69 | 393.77 |
| SLC31A2 | solute carrier family 31 (copper transporters). member 2 | 2.0117 | 7.53E-08 | 454.24 | 376.91 | 878.22 | 788.97 |
| CHKA | choline kinase alpha | 2.001 | 2.66E-09 | 896.75 | 887.36 | 1795.7 | 1774.2 |
| LRIG1 | leucine-rich repeats and immunoglobulin-like domains 1 | 1.9942 | 5.85E-09 | 492.27 | 495.77 | 1028.7 | 943.4 |
| SLC2A3 | solute carrier family 2 (facilitated glucose transporter). member 3 | 1.9904 | 2.51E-08 | 226.46 | 191.67 | 428.32 | 401.48 |
| FAM64A | family with sequence similarity 64. member A | 1.9489 | 1.25E-05 | 206.98 | 176.81 | 390.54 | 355.91 |
| P4HA1 | procollagen-proline. 2-oxoglutarate 4-dioxygenase (proline 4-hydroxylase). alpha polypeptide I | 1.9265 | 1.77E-05 | 389.91 | 452.07 | 783.32 | 835.15 |
| ERRFI1 | ERBB receptor feedback inhibitor 1 | 1.9256 | 2.69E-07 | 2741.6 | 2235.4 | 4767.1 | 4767.1 |
| ZFP36 | zinc finger protein 36. C3H type. homolog (mouse) | 1.9177 | 4.55E-08 | 424.96 | 410.09 | 755.42 | 848.38 |
| TAF15 | TAF15 RNA polymerase II. TATA box binding protein (TBP)-associated factor. 68kDa | 1.9176 | 0.00067 | 1942.9 | 2957.9 | 4628.4 | 4565.8 |
| SC4MOL | sterol-C4-methyl oxidase-like | 1.9169 | 1.35E-08 | 244.17 | 234.51 | 442.08 | 475.92 |
| NUSAP1 | nucleolar and spindle associated protein 1 | 1.9119 | 0.00067 | 430.98 | 338.46 | 723.31 | 737.17 |
| TUBB2A | tubulin. beta 2A | 1.8989 | 5.90E-05 | 673.25 | 658.02 | 1094.4 | 1459.8 |
| AP3S1 | adaptor-related protein complex 3. sigma 1 subunit | 1.8971 | 1.89E-08 | 581.99 | 546.43 | 1127.8 | 1014.8 |
| SELS | selenoprotein S | 1.895 | 2.17E-08 | 4122.7 | 3733.3 | 7366.4 | 7503.4 |
| OSBPL5 | oxysterol binding protein-like 5 | 1.8934 | 2.76E-10 | 470.37 | 457.73 | 901.39 | 856.34 |
| SLC27A3 | solute carrier family 27 (fatty acid transporter). member 3 | 1.8904 | 1.76E-06 | 400.27 | 350 | 658.02 | 760.82 |
| ULK1 | unc-51-like kinase 1 (C. elegans) | 1.8878 | 8.92E-07 | 1768.1 | 1638.4 | 3202.7 | 3223.5 |
| CCNB2 | cyclin B2 | 1.8815 | 0.00043 | 697.92 | 545.98 | 1159.3 | 1163.6 |
| TRIM24 | tripartite motif-containing 24 | 1.867 | 2.54E-07 | 1183 | 1049.8 | 2160.6 | 2003.7 |
| VCL | vinculin | 1.8657 | 3.60E-07 | 5283 | 4225.6 | 8471.8 | 9171.9 |
| CMIP | c-Maf-inducing protein | 1.8468 | 3.25E-10 | 1209.3 | 1222.1 | 2285.7 | 2205.1 |
| FBXO31 | F-box protein 31 | 1.829 | 7.86E-10 | 566.73 | 572.61 | 1075.2 | 1009.7 |
| AASS | aminoadipate-semialdehyde synthase | 1.8194 | 2.01E-08 | 167.68 | 174.57 | 288.18 | 336.24 |
| ANKRD37 | ankyrin repeat domain 37 | 1.814 | 1.06E-08 | 261.31 | 243.68 | 481.93 | 434.77 |
| C17orf58 | chromosome 17 open reading frame 58 | 1.8098 | 6.10E-09 | 470.93 | 471.08 | 852.29 | 852.58 |
| PTTG1 | pituitary tumor-transforming 1 | 1.8074 | 0.00058 | 688.84 | 501.15 | 1047.7 | 1076.3 |
| SCNN1G | sodium channel. nonvoltage-gated 1. gamma | 1.8071 | 3.75E-08 | 277.16 | 264.89 | 487.39 | 491.9 |
| KCTD3 | potassium channel tetramerisation domain containing 3 | 1.7996 | 2.11E-05 | 561.75 | 516.34 | 1043.6 | 900.11 |
| CAMK2N1 | calcium/calmodulin-dependent protein kinase II inhibitor 1 | 1.7995 | 5.50E-08 | 2137.4 | 2017.6 | 4001.9 | 3489.5 |
| SLCO4A1 | solute carrier organic anion transporter family. member 4A1 | 1.7978 | 6.32E-09 | 507.88 | 486.86 | 945.25 | 845.47 |
| CREB3L2 | cAMP responsive element binding protein 3-like 2 | 1.7915 | 2.49E-05 | 587.18 | 563.6 | 1136 | 934.96 |
| C17orf80 | chromosome 17 open reading frame 80 | 1.791 | 2.62E-07 | 203.14 | 177.21 | 344.95 | 334.75 |
| BCL6 | B-cell CLL/lymphoma 6 (zinc finger protein 51) | 1.7653 | 2.78E-05 | 1804.5 | 1527.9 | 2884.4 | 2978.6 |
| NCAPG | non-SMC condensin I complex. subunit G | 1.761 | 0.00084 | 246.91 | 209.59 | 420.04 | 382.07 |
| OLAH | oleoyl-ACP hydrolase | 1.7579 | 8.35E-09 | 161.05 | 152.67 | 267.65 | 283.88 |
| IRS2 | insulin receptor substrate 2 | 1.7549 | 1.71E-06 | 260.09 | 220.15 | 418.33 | 421.52 |
| KIAA1826 | KIAA1826 | 1.7531 | 5.91E-05 | 738.75 | 637.61 | 1205.9 | 1200.5 |
| PRC1 | protein regulator of cytokinesis 1 | 1.7484 | 8.51E-05 | 451.49 | 374.3 | 712.7 | 724.86 |
| C9orf152 | chromosome 9 open reading frame 152 | 1.7462 | 1.46E-07 | 1432.9 | 1349.5 | 2349.2 | 2510 |
| CEBPD | CCAAT/enhancer binding protein (C/EBP). delta | 1.7387 | 6.39E-08 | 939.83 | 952.42 | 1713.1 | 1579.6 |
| PAK1IP1 | PAK1 interacting protein 1 | 1.7345 | 3.41E-08 | 1120 | 1047.7 | 1849.8 | 1908.5 |
| DUSP1 | dual specificity phosphatase 1 | 1.7325 | 2.45E-05 | 1304.7 | 1026.4 | 1933.6 | 2078.7 |
| FAM105A | family with sequence similarity 105. member A | 1.7294 | 5.98E-10 | 274.49 | 269.77 | 478.68 | 462.67 |
| CKB | creatine kinase. brain | 1.7289 | 1.79E-07 | 3591.1 | 3683.4 | 6484.7 | 6097.4 |
| SLC39A11 | solute carrier family 39 (metal ion transporter). member 11 | 1.7221 | 1.57E-09 | 380.41 | 391.87 | 672.48 | 657.38 |
| C16orf44 | chromosome 16 open reading frame 44 | 1.7166 | 4.32E-09 | 685.38 | 688.84 | 1207.2 | 1152.4 |
| CDC2 | cell division cycle 2. G1 to S and G2 to M | 1.7153 | 0.00031 | 404.98 | 339.08 | 551.3 | 732.89 |
| ALDH6A1 | aldehyde dehydrogenase 6 family. member A1 | 1.715 | 1.49E-06 | 1437.3 | 1384.7 | 2483.8 | 2356.7 |
| KIF2C | kinesin family member 2C | 1.7077 | 6.62E-05 | 238.54 | 202.55 | 383.06 | 367.84 |
| SERPINA3 | serpin peptidase inhibitor. clade A (alpha-1 antiproteinase. antitrypsin). member 3 | 1.705 | 1.84E-07 | 444.51 | 469.19 | 766.5 | 791.03 |
| EGFR | epidermal growth factor receptor (erythroblastic leukemia viral (v-erb-b) oncogene homolog. avian) | 1.7022 | 3.06E-07 | 229.3 | 211.71 | 394.46 | 356.56 |
| ASB9 | ankyrin repeat and SOCS box-containing 9 | 1.6969 | 8.59E-08 | 149.31 | 146.41 | 247.13 | 254.72 |
| STX12 | syntaxin 12 | 1.695 | 1.12E-07 | 454.84 | 495.23 | 754.76 | 857.41 |
| CENPF | centromere protein F. 350/400ka (mitosin) | 1.6911 | 8.41E-05 | 215.47 | 178.45 | 336.82 | 326.47 |
| FAM129B | family with sequence similarity 129. member B | 1.6878 | 8.72E-06 | 4793.9 | 4157.7 | 6995.1 | 8116.8 |
| FAM83D | family with sequence similarity 83. member D | 1.6859 | 3.97E-05 | 199.93 | 177.1 | 321.91 | 312.61 |
| LBR | lamin B receptor | 1.6857 | 1.67E-05 | 1944.4 | 1771.4 | 3492.3 | 2802.6 |
| NETO1 | neuropilin (NRP) and tolloid (TLL)-like 1 | 1.6796 | 1.85E-07 | 210.37 | 208.38 | 370.89 | 333.42 |
| PSRC1 | proline/serine-rich coiled-coil 1 | 1.678 | 1.25E-05 | 203.25 | 181.09 | 334.3 | 309.99 |
| TMEM16F | transmembrane protein 16F | 1.6766 | 1.03E-06 | 289.83 | 309.9 | 554.12 | 455.63 |
| CAP2 | CAP. adenylate cyclase-associated protein. 2 (yeast) | 1.6762 | 7.57E-05 | 2472 | 1893.5 | 3473.7 | 3785.8 |
| SCAP | SREBF chaperone | 1.674 | 1.05E-07 | 4261.6 | 4084.1 | 7160.8 | 6811 |
| NUDT16 | nudix (nucleoside diphosphate linked moiety X)-type motif 16 | 1.6664 | 2.41E-08 | 274.1 | 275.97 | 457.73 | 458.92 |
| HMGB3 | high-mobility group box 3 | 1.6658 | 6.96E-08 | 194.82 | 191.53 | 323.01 | 320.53 |
| DHCR7 | 7-dehydrocholesterol reductase | 1.6654 | 1.49E-07 | 795.44 | 789.97 | 1359.7 | 1281.8 |
| CDH2 | cadherin 2. type 1. N-cadherin (neuronal) | 1.6566 | 8.45E-06 | 123.73 | 133.36 | 186.35 | 243 |
| TRPM4 | transient receptor potential cation channel. subfamily M. member 4 | 1.6543 | 2.91E-08 | 5132 | 5118.7 | 8969.7 | 8015.2 |
| PRKAB2 | protein kinase. AMP-activated. beta 2 non-catalytic subunit | 1.6543 | 8.34E-09 | 236.61 | 246.72 | 394.35 | 405.1 |
| ZNF259 | zinc finger protein 259 | 1.6535 | 1.16E-08 | 334.95 | 362.45 | 577.7 | 574.58 |
| LDHA | lactate dehydrogenase A | 1.6485 | 5.51E-06 | 6756.3 | 6172.3 | 11258 | 10066 |
| ZDHHC9 | zinc finger. DHHC-type containing 9 | 1.6372 | 4.78E-06 | 442.73 | 475.69 | 820.79 | 687.78 |
| MCFD2 | multiple coagulation factor deficiency 2 | 1.6366 | 1.39E-07 | 466.85 | 429.16 | 729.98 | 735.11 |
| KLF15 | Kruppel-like factor 15 | 1.6339 | 6.34E-09 | 212.59 | 212.42 | 338.36 | 356.28 |
| SHC1 | SHC (Src homology 2 domain containing) transforming protein 1 | 1.6303 | 1.55E-07 | 2301.8 | 2367.3 | 3798 | 3813.4 |
| RBM24 | RNA binding motif protein 24 | 1.6291 | 3.52E-08 | 299.65 | 298.99 | 477.73 | 497.69 |
| FADS1 | fatty acid desaturase 1 | 1.6283 | 1.02E-07 | 782.24 | 729.05 | 1198 | 1262.1 |
| CEP55 | centrosomal protein 55kDa | 1.628 | 0.00024 | 224.67 | 184.97 | 327.65 | 336.15 |
| SLC30A7 | solute carrier family 30 (zinc transporter). member 7 | 1.6267 | 6.87E-06 | 540.65 | 512.71 | 888 | 826.05 |
| C5orf4 | chromosome 5 open reading frame 4 | 1.6224 | 1.13E-08 | 171.56 | 157.85 | 276.06 | 258.21 |
| CCNA2 | cyclin A2 | 1.6152 | 4.11E-06 | 203.67 | 187.71 | 332.7 | 299.8 |
| DNAJB9 | DnaJ (Hsp40) homolog. subfamily B. member 9 | 1.6126 | 2.14E-05 | 1517.8 | 1407.4 | 2283.7 | 2432.4 |
| CDCA8 | cell division cycle associated 8 | 1.606 | 3.25E-05 | 221.12 | 200.12 | 355.45 | 321.08 |
| PFKFB4 | 6-phosphofructo-2-kinase/fructose-2.6-biphosphatase 4 | 1.6044 | 1.85E-06 | 412.63 | 386.45 | 678 | 605.4 |
| PRPS2 | phosphoribosyl pyrophosphate synthetase 2 | 1.6042 | 6.98E-05 | 339.75 | 362.24 | 505.47 | 626.57 |
| ADNP2 | ADNP homeobox 2 | 1.6032 | 1.43E-05 | 816.56 | 835.89 | 1367.9 | 1282.4 |
| CALU | calumenin | 1.5963 | 4.23E-06 | 789.97 | 711.14 | 1252.7 | 1142.7 |
| ITGB1 | integrin. beta 1 (fibronectin receptor. beta polypeptide. antigen CD29 includes MDF2. MSK12) | 1.5953 | 1.75E-05 | 661.29 | 718.71 | 1149.8 | 1052 |
| VPS33B | vacuolar protein sorting 33 homolog B (yeast) | 1.5926 | 2.29E-07 | 282.98 | 272.15 | 474.32 | 411.84 |
| CTDSPL | CTD (carboxy-terminal domain. RNA polymerase II. polypeptide A) small phosphatase-like | 1.5922 | 2.96E-07 | 640.32 | 674.57 | 1039.9 | 1053 |
| AURKB | aurora kinase B | 1.5908 | 4.75E-06 | 200.15 | 182.34 | 311.9 | 296.09 |
| C8orf42 | chromosome 8 open reading frame 42 | 1.5889 | 3.03E-07 | 375.66 | 316.36 | 529.41 | 566.73 |
| SCNN1A | sodium channel. nonvoltage-gated 1 alpha | 1.5883 | 1.85E-06 | 4636.9 | 4697.9 | 6832.4 | 8043.4 |
| ABHD2 | abhydrolase domain containing 2 | 1.5868 | 4.05E-05 | 513.35 | 482.05 | 828.74 | 751.82 |
| EFNA1 | ephrin-A1 | 1.5866 | 8.24E-09 | 3356.7 | 3444.4 | 5588.4 | 5208 |
| DOCK5 | dedicator of cytokinesis 5 | 1.584 | 4.44E-07 | 198.66 | 184.87 | 306.96 | 300.22 |
| ELL2 | elongation factor. RNA polymerase II. 2 | 1.5818 | 9.06E-08 | 334.21 | 324.37 | 522.34 | 519.28 |
| HIST1H4C | histone cluster 1. H4c | 1.5791 | 6.99E-05 | 5879.9 | 5996.2 | 9090.3 | 9671.2 |
| CST3 | cystatin C (amyloid angiopathy and cerebral hemorrhage) | 1.5779 | 2.35E-08 | 5621.3 | 6131.2 | 9319.8 | 9207.4 |
| NT5DC3 | 5'-nucleotidase domain containing 3 | 1.5776 | 4.55E-08 | 580.36 | 568.35 | 884.86 | 927.7 |
| MED30 | mediator complex subunit 30 | 1.5759 | 7.95E-06 | 415.18 | 385.6 | 636.03 | 625.1 |
| KLF9 | Kruppel-like factor 9 | 1.5695 | 1.92E-05 | 2504.8 | 2281.8 | 3791.9 | 3712.6 |
| CTH | cystathionase (cystathionine gamma-lyase) | 1.5685 | 1.92E-08 | 1252 | 1287.7 | 1885.8 | 2103.4 |
| CKS1B | CDC28 protein kinase regulatory subunit 1B | 1.568 | 0.00106 | 1560.8 | 1280.7 | 2341.9 | 2098.4 |
| HSPA5 | heat shock 70kDa protein 5 (glucose-regulated protein. 78kDa) | 1.5655 | 1.62E-05 | 594.07 | 511.28 | 839.19 | 886.99 |
| FAT | FAT tumor suppressor homolog 1 (Drosophila) | 1.5649 | 7.67E-06 | 778.33 | 757.06 | 1116.7 | 1292.3 |
| KRT8 | keratin 8 | 1.5608 | 7.80E-07 | 2314.9 | 2205.1 | 3444.4 | 3610.3 |
| AARS | alanyl-tRNA synthetase | 1.5587 | 7.59E-05 | 10014 | 9563.3 | 15105 | 15403 |
| CLPTM1L | CLPTM1-like | 1.5543 | 8.81E-05 | 1470.4 | 1563.5 | 2539.8 | 2186.8 |
| NNT | nicotinamide nucleotide transhydrogenase | 1.5426 | 6.17E-05 | 213.37 | 180.63 | 296.46 | 309.35 |
| BIRC3 | baculoviral IAP repeat-containing 3 | 1.5404 | 0.00016 | 338.97 | 299.41 | 482.44 | 499.2 |
| KIAA0182 | KIAA0182 | 1.539 | 5.71E-06 | 1363.6 | 1314.4 | 2005.4 | 2116.8 |
| BAT1 | HLA-B associated transcript 1 | 1.5382 | 1.77E-06 | 1599.5 | 1425.5 | 2330.5 | 2314.9 |
| PHF17 | PHD finger protein 17 | 1.538 | 1.39E-07 | 425.41 | 405.1 | 640.72 | 636.27 |
| FOXO3 | forkhead box O3 | 1.5348 | 1.60E-05 | 4029.9 | 3604.5 | 5706.3 | 5996.2 |
| NDRG1 | N-myc downstream regulated gene 1 | 1.5345 | 1.55E-05 | 802.47 | 723.31 | 1224.3 | 1116.3 |
| UNC84B | unc-84 homolog B (C. elegans) | 1.5314 | 7.25E-06 | 351.99 | 375.66 | 598.09 | 518.49 |
| ZNF364 | zinc finger protein 364 | 1.5307 | 9.22E-06 | 867.9 | 888 | 1407.4 | 1283 |
| CDCA5 | cell division cycle associated 5 | 1.5288 | 4.74E-05 | 258.83 | 227.85 | 373.21 | 369.31 |
| STK35 | serine/threonine kinase 35 | 1.5267 | 3.14E-05 | 927.7 | 960.37 | 1442.9 | 1439.3 |
| WDR60 | WD repeat domain 60 | 1.5252 | 3.69E-05 | 295.56 | 242.65 | 406.8 | 410.09 |
| KIAA0101 | KIAA0101 | 1.5251 | 0.00025 | 316.01 | 264.18 | 457.09 | 424.82 |
| TNFRSF19 | tumor necrosis factor receptor superfamily. member 19 | 1.5225 | 4.10E-06 | 271.24 | 257.73 | 406.39 | 398.75 |
| ARMET | arginine-rich. mutated in early stage tumors | 1.5222 | 8.40E-08 | 8043.4 | 7875.8 | 12402 | 11835 |
| DENND1A | DENN/MADD domain containing 1A | 1.5209 | 5.39E-07 | 1093.8 | 1104 | 1710.6 | 1632.8 |
| PDIA5 | protein disulfide isomerase family A. member 5 | 1.5187 | 6.55E-05 | 610.74 | 613.33 | 1030.6 | 838.32 |
| PDZD8 | PDZ domain containing 8 | 1.5179 | 3.80E-06 | 290.96 | 280.53 | 463.74 | 405.51 |
| PHLDA1 | pleckstrin homology-like domain. family A. member 1 | 1.515 | 6.20E-07 | 147.58 | 134.37 | 218.06 | 208.73 |
| ATP11B | ATPase. Class VI. type 11B | 1.5146 | 1.21E-06 | 191.55 | 221.49 | 320.99 | 303.24 |
| USP33 | ubiquitin specific peptidase 33 | 1.5123 | 2.80E-05 | 182.27 | 194.92 | 257.56 | 315.49 |
| SLC2A1 | solute carrier family 2 (facilitated glucose transporter). member 1 | 1.5123 | 7.56E-07 | 187.58 | 170.91 | 287.1 | 255.37 |
| PTPRM | protein tyrosine phosphatase. receptor type. M | 1.5114 | 9.02E-06 | 510.53 | 490.17 | 796.5 | 717.66 |
| DCXR | dicarbonyl/L-xylulose reductase | 1.5103 | 7.38E-07 | 3090.3 | 3437.8 | 4873 | 4972.7 |
| TCEAL3 | transcription elongation factor A (SII)-like 3 | 1.51 | 5.17E-06 | 660.3 | 576.48 | 880.16 | 986.07 |
| FUT3 | fucosyltransferase 3 (galactoside 3(4)-L-fucosyltransferase. Lewis blood group) | 1.5097 | 2.31E-06 | 189.68 | 192.6 | 295.18 | 282.07 |
| TXLNA | taxilin alpha | 1.5087 | 7.34E-05 | 3870.5 | 3663.3 | 5178 | 6232.5 |
| LOX | lysyl oxidase | 1.5079 | 3.34E-07 | 125.75 | 117.94 | 179.66 | 187.71 |
| MMP25 | matrix metallopeptidase 25 | 1.5034 | 1.49E-07 | 139.21 | 140.36 | 206.78 | 213.59 |
| GBA | glucosidase. beta; acid (includes glucosylceramidase) | 1.4955 | 5.33E-06 | 301.29 | 308.5 | 473.3 | 439.22 |
| ADM | adrenomedullin | 1.4953 | 3.10E-06 | 217.94 | 182.74 | 291.69 | 305.29 |
| HECTD2 | HECT domain containing 2 | 1.4916 | 6.25E-09 | 200.82 | 207.27 | 305.82 | 302.82 |
| C5orf13 | chromosome 5 open reading frame 13 | 1.4912 | 5.87E-06 | 511.1 | 505.47 | 770.16 | 745.95 |
| SYT4 | synaptotagmin IV | 1.4884 | 1.66E-05 | 3351 | 3473.7 | 5325 | 4842.7 |
| MACROD1 | MACRO domain containing 1 | 1.4866 | 4.06E-07 | 295.75 | 283.58 | 450.59 | 411.38 |
| CD55 | CD55 molecule. decay accelerating factor for complement (Cromer blood group) | 1.4861 | 2.88E-05 | 581.37 | 652.28 | 956.5 | 875.6 |
| TMED5 | transmembrane emp24 protein transport domain containing 5 | 1.4854 | 0.0037 | 2014 | 1557.6 | 2750.9 | 2516.1 |
| GLUD1 | glutamate dehydrogenase 1 | 1.4844 | 4.40E-07 | 4088.8 | 4254.8 | 5849.8 | 6553.1 |
| SRF | serum response factor (c-fos serum response element-binding transcription factor) | 1.4802 | 4.86E-06 | 1274 | 1151.9 | 1803.1 | 1783.3 |
| MFGE8 | milk fat globule-EGF factor 8 protein | 1.4753 | 6.97E-06 | 506.81 | 491.78 | 742.77 | 730.34 |
| MYADM | myeloid-associated differentiation marker | 1.4743 | 0.00052 | 333.94 | 351.46 | 491.08 | 519.52 |
| SDF2L1 | stromal cell-derived factor 2-like 1 | 1.4722 | 8.06E-06 | 2162.3 | 2237.2 | 3420.4 | 3065.3 |
| PUS1 | pseudouridylate synthase 1 | 1.4697 | 4.06E-06 | 1175 | 1110.8 | 1596.6 | 1765.8 |
| IFNGR1 | interferon gamma receptor 1 | 1.4678 | 3.85E-05 | 1182.1 | 1078.6 | 1718.6 | 1598.5 |
| H2AFZ | H2A histone family. member Z | 1.4649 | 1.14E-05 | 13782 | 11906 | 17722 | 19870 |
| C12orf44 | chromosome 12 open reading frame 44 | 1.4612 | 6.08E-05 | 1073.8 | 1041.3 | 1595.8 | 1496.1 |
| PDPK1 | 3-phosphoinositide dependent protein kinase-1 | 1.4604 | 1.73E-06 | 355.36 | 380.18 | 514.34 | 560.2 |
| PLEKHC1 | pleckstrin homology domain containing. family C (with FERM domain) member 1 | 1.4591 | 3.89E-05 | 940.38 | 927.7 | 1369.2 | 1356.4 |
| C17orf48 | chromosome 17 open reading frame 48 | 1.4579 | 7.41E-08 | 458.26 | 466.85 | 688.3 | 660.67 |
| RALY | RNA binding protein. autoantigenic (hnRNP-associated with lethal yellow homolog (mouse)) | 1.4565 | 6.32E-06 | 1038.2 | 1079.4 | 1603.9 | 1482.1 |
| MYBPC1 | myosin binding protein C. slow type | 1.4561 | 1.54E-06 | 168.02 | 158.28 | 230.47 | 244.67 |
| ALDOC | aldolase C. fructose-bisphosphate | 1.4446 | 1.22E-05 | 2680.9 | 2554.8 | 3635.7 | 3931.4 |
| TSC2 | tuberous sclerosis 2 | 1.4443 | 2.36E-05 | 539.71 | 606.32 | 783.01 | 871.83 |
| STRBP | spermatid perinuclear RNA binding protein | 1.443 | 0.00147 | 670.69 | 558.08 | 825.72 | 943.9 |
| CNN2 | calponin 2 | 1.4414 | 5.51E-06 | 525.24 | 497.32 | 711.14 | 763.12 |
| TSC22D3 | TSC22 domain family. member 3 | 1.441 | 1.94E-05 | 4427.2 | 3971.9 | 6131.2 | 5955 |
| DNAJB11 | DnaJ (Hsp40) homolog. subfamily B. member 11 | 1.44 | 3.83E-05 | 4360.7 | 3521.8 | 5720.1 | 5567.7 |
| MBNL1 | muscleblind-like (Drosophila) | 1.4391 | 0.00026 | 622.22 | 502.84 | 754.19 | 859.15 |
| LDLR | low density lipoprotein receptor (familial hypercholesterolemia) | 1.439 | 6.47E-06 | 1215.6 | 1020.2 | 1622.2 | 1583 |
| CLEC16A | C-type lectin domain family 16. member A | 1.4379 | 0.00016 | 621.4 | 580.85 | 850.03 | 877.89 |
| TDRD9 | tudor domain containing 9 | 1.433 | 8.56E-07 | 162.62 | 157.79 | 228.91 | 230.21 |
| CBX4 | chromobox homolog 4 (Pc class homolog. Drosophila) | 1.428 | 9.07E-07 | 1084.1 | 1111.6 | 1548.1 | 1587.2 |
| RPN1 | ribophorin I | 1.4279 | 8.91E-06 | 6399.7 | 6354.3 | 9519 | 8709.9 |
| LGMN | legumain | 1.427 | 0.00016 | 1343.4 | 1530.8 | 2116.8 | 1978.2 |
| PDHA1 | pyruvate dehydrogenase (lipoamide) alpha 1 | 1.4265 | 2.52E-05 | 1830.1 | 2021.1 | 2568.2 | 2930.7 |
| KLF6 | Kruppel-like factor 6 | 1.4221 | 0.00014 | 741.43 | 767.04 | 966.04 | 1190.5 |
| BCKDHB | branched chain keto acid dehydrogenase E1. beta polypeptide (maple syrup urine disease) | 1.4204 | 0.00012 | 417.54 | 417.02 | 628.23 | 559.17 |
| AXUD1 | AXIN1 up-regulated 1 | 1.4148 | 3.11E-05 | 773.35 | 711.7 | 1105.7 | 996.31 |
| SLC38A2 | solute carrier family 38. member 2 | 1.414 | 0.00959 | 2136.1 | 2125.4 | 2647.7 | 3428.4 |
| SGSM2 | small G protein signaling modulator 2 | 1.4136 | 2.52E-05 | 1980.9 | 1931.1 | 2667.7 | 2865.1 |
| SLC38A1 | solute carrier family 38. member 1 | 1.4132 | 4.99E-06 | 2377.7 | 2270 | 3263.6 | 3302.7 |
| FLVCR2 | feline leukemia virus subgroup C cellular receptor family. member 2 | 1.4022 | 8.82E-06 | 296.09 | 274.65 | 406.24 | 393.62 |
| SPOCK1 | sparc/osteonectin. cwcv and kazal-like domains proteoglycan (testican) 1 | 1.4022 | 2.22E-06 | 174.89 | 164.81 | 238.88 | 237.23 |
| TPCN2 | two pore segment channel 2 | 1.3991 | 1.16E-05 | 321.22 | 311.75 | 433.23 | 452.48 |
| TGIF1 | TGFB-induced factor homeobox 1 | 1.3991 | 3.94E-05 | 436.21 | 420.29 | 622.76 | 576.26 |
| DDC | dopa decarboxylase (aromatic L-amino acid decarboxylase) | 1.397 | 0.00055 | 5057.9 | 6040.7 | 7790.1 | 7654.4 |
| SSR1 | signal sequence receptor. alpha (translocon-associated protein alpha) | 1.3955 | 0.00042 | 3094.5 | 2651.9 | 4185.5 | 3818.4 |
| DDOST | dolichyl-diphosphooligosaccharide-protein glycosyltransferase | 1.3929 | 8.35E-06 | 2919.8 | 3277.1 | 4314.8 | 4302.6 |
| CDR2 | cerebellar degeneration-related protein 2. 62kDa | 1.3915 | 0.00012 | 389.12 | 365.55 | 498.72 | 552.24 |
| PPP3R1 | protein phosphatase 3 (formerly 2B). regulatory subunit B. alpha isoform | 1.3899 | 4.39E-05 | 841.86 | 855.48 | 1209.3 | 1150.5 |
| SPSB1 | splA/ryanodine receptor domain and SOCS box containing 1 | 1.3885 | 2.99E-06 | 233.26 | 214.7 | 325.49 | 296.63 |
| TP53BP2 | tumor protein p53 binding protein. 2 | 1.3879 | 0.00033 | 648.35 | 584.04 | 846.06 | 862.13 |
| F2RL1 | coagulation factor II (thrombin) receptor-like 1 | 1.3859 | 0.00212 | 938.6 | 875.95 | 1376 | 1147.6 |
| SLC39A1 | solute carrier family 39 (zinc transporter). member 1 | 1.3847 | 0.00039 | 614.2 | 582.89 | 863 | 795.44 |
| FAM58A | family with sequence similarity 58. member A | 1.3824 | 2.84E-06 | 503.22 | 464.18 | 666.52 | 669.76 |
| GRB10 | growth factor receptor-bound protein 10 | 1.3806 | 0.00029 | 970.16 | 1116.3 | 1517 | 1360.8 |
| CRELD2 | cysteine-rich with EGF-like domains 2 | 1.3799 | 2.26E-05 | 2930.7 | 2780.7 | 4116.2 | 3769.7 |
| ERICH1 | glutamate-rich 1 | 1.3795 | 2.95E-06 | 296.76 | 298.92 | 435.65 | 387.49 |
| ZCCHC11 | zinc finger. CCHC domain containing 11 | 1.3792 | 0.00044 | 340.26 | 306.06 | 447.88 | 442.29 |
| FLJ37464 | hypothetical protein FLJ37464 | 1.378 | 0.00032 | 151.92 | 137.65 | 189.07 | 210.03 |
| GOLPH3L | golgi phosphoprotein 3-like | 1.3716 | 0.00012 | 957.48 | 938.6 | 1304.7 | 1295.8 |
| LMO4 | LIM domain only 4 | 1.3703 | 0.00104 | 1186.4 | 1004.1 | 1453.2 | 1539.4 |
| MYO6 | myosin VI | 1.3698 | 0.00203 | 861.69 | 904.42 | 1313.7 | 1113.1 |
| HYOU1 | hypoxia up-regulated 1 | 1.3676 | 3.79E-06 | 354.67 | 351.81 | 482.51 | 483.66 |
| ZNF23 | zinc finger protein 23 (KOX 16) | 1.3675 | 2.74E-05 | 218.11 | 216.96 | 307.02 | 288.25 |
| CDH15 | cadherin 15. M-cadherin (myotubule) | 1.3652 | 1.07E-06 | 177.36 | 178.04 | 252.05 | 233.48 |
| ENPP4 | ectonucleotide pyrophosphatase/phosphodiesterase 4 (putative function) | 1.355 | 0.00107 | 396.21 | 342.7 | 516.01 | 483.09 |
| ERN1 | endoplasmic reticulum to nucleus signaling 1 | 1.3549 | 6.23E-06 | 1195.6 | 1059.3 | 1535.7 | 1513.8 |
| PYGB | phosphorylase. glycogen; brain | 1.3533 | 5.64E-05 | 1807 | 1780.5 | 2454.3 | 2400.7 |
| ACTB | actin. beta | 1.3506 | 0.00507 | 8471.8 | 7314.3 | 11331 | 9976.4 |
| SEC23B | Sec23 homolog B (S. cerevisiae) | 1.3506 | 1.31E-06 | 2130.5 | 1997.3 | 2829.3 | 2743.7 |
| PEG3 | paternally expressed 3 | 1.3498 | 0.00012 | 333.52 | 324.06 | 471.08 | 418 |
| LRRFIP2 | leucine rich repeat (in FLII) interacting protein 2 | 1.3491 | 5.88E-06 | 594.9 | 549.87 | 773.02 | 770.16 |
| TMED9 | transmembrane emp24 protein transport domain containing 9 | 1.3476 | 0.00033 | 2281.8 | 2551.4 | 3239.2 | 3263.6 |
| ERGIC1 | endoplasmic reticulum-golgi intermediate compartment (ERGIC) 1 | 1.3448 | 0.00186 | 2881 | 3302.7 | 4199 | 4098.2 |
| BBS1 | Bardet-Biedl syndrome 1 | 1.3447 | 1.45E-05 | 390.07 | 422.23 | 565.15 | 526.99 |
| SOCS2 | suppressor of cytokine signaling 2 | 1.3446 | 5.61E-06 | 217.61 | 214.04 | 305.77 | 275.4 |
| MDC1 | mediator of DNA damage checkpoint 1 | 1.3441 | 0.00013 | 456.24 | 418.53 | 563.28 | 612.4 |
| TFF3 | trefoil factor 3 (intestinal) | 1.3434 | 0.00039 | 347.29 | 403.67 | 519.11 | 487.39 |
| GOT1 | glutamic-oxaloacetic transaminase 1. soluble (aspartate aminotransferase 1) | 1.3434 | 0.00072 | 4025.7 | 3428.4 | 4672.6 | 5330.3 |
| B2M | beta-2-microglobulin | 1.343 | 0.00961 | 3740.6 | 4001.9 | 4812.4 | 5610.2 |
| DC2 | DC2 protein | 1.3416 | 3.00E-05 | 3011 | 2973 | 3777.2 | 4265.3 |
| TF | transferrin | 1.3411 | 7.87E-06 | 131.43 | 132.75 | 170.89 | 183.63 |
| EAF1 | ELL associated factor 1 | 1.3409 | 0.08912 | 344.35 | 430.3 | 418.08 | 637.22 |
| ZMYND8 | zinc finger. MYND-type containing 8 | 1.3408 | 6.61E-06 | 305.29 | 281.38 | 400.59 | 385.53 |
| TSPAN3 | tetraspanin 3 | 1.3383 | 5.15E-05 | 1030.6 | 1158.2 | 1522.2 | 1404.5 |
| SH3PX3 | SH3 and PX domain containing 3 | 1.3374 | 9.93E-07 | 163.64 | 159.39 | 221.26 | 210.85 |
| ABCD3 | ATP-binding cassette. sub-family D (ALD). member 3 | 1.3371 | 3.21E-06 | 354.58 | 365.46 | 469.38 | 493.55 |
| FNDC3B | fibronectin type III domain containing 3B | 1.3342 | 0.00085 | 374.78 | 387.05 | 475.08 | 543.48 |
| CCPG1 | cell cycle progression 1 | 1.3323 | 0.0037 | 483.96 | 451.49 | 650.73 | 595.98 |
| NBN | nibrin | 1.3311 | 0.004 | 651.17 | 586.46 | 845.47 | 800.26 |
| PALLD | palladin. cytoskeletal associated protein | 1.33 | 0.00253 | 834.48 | 679.58 | 985.44 | 1017.9 |
| PDLIM1 | PDZ and LIM domain 1 (elfin) | 1.3269 | 0.00173 | 325.21 | 362.34 | 438.61 | 473.02 |
| CDKN3 | cyclin-dependent kinase inhibitor 3 (CDK2-associated dual specificity phosphatase) | 1.3248 | 0.01184 | 380.89 | 313.89 | 475.57 | 441.22 |
| TMEM61 | transmembrane protein 61 | 1.3248 | 2.63E-06 | 146.2 | 148.1 | 201.32 | 188.75 |
| ACAT2 | acetyl-Coenzyme A acetyltransferase 2 (acetoacetyl Coenzyme A thiolase) | 1.3223 | 2.49E-07 | 1326.7 | 1350.7 | 1773.4 | 1766.9 |
| ARG1 | arginase. liver | 1.3196 | 1.88E-06 | 166.28 | 174.06 | 217.03 | 232.21 |
| RANBP10 | RAN binding protein 10 | 1.3193 | 0.00014 | 244.67 | 255.21 | 354.46 | 306.63 |
| IL2RB | interleukin 2 receptor. beta | 1.3182 | 6.62E-05 | 164.53 | 158.15 | 207.86 | 217.53 |
| PRAC | small nuclear protein PRAC | 1.3162 | 0.0001 | 10313 | 10166 | 14184 | 12805 |
| WDR54 | WD repeat domain 54 | 1.3156 | 0.00024 | 1243.4 | 1373.5 | 1777.1 | 1663.3 |
| AKR1D1 | aldo-keto reductase family 1. member D1 (delta 4-3-ketosteroid-5-beta-reductase) | 1.3126 | 5.54E-07 | 129.69 | 124.12 | 165.03 | 168.03 |
| ZNF165 | zinc finger protein 165 | 1.3112 | 0.00078 | 296.46 | 272.52 | 367.19 | 378.26 |
| EGFLAM | EGF-like. fibronectin type III and laminin G domains | 1.3102 | 5.94E-07 | 133.22 | 133.27 | 178.43 | 170.81 |
| USP38 | ubiquitin specific peptidase 38 | 1.3094 | 0.00082 | 551.72 | 522.14 | 664.93 | 742.77 |
| ITPR3 | inositol 1.4.5-triphosphate receptor. type 3 | 1.3091 | 6.95E-05 | 1257.4 | 1147.6 | 1615.5 | 1530.8 |
| C6orf85 | chromosome 6 open reading frame 85 | 1.3091 | 0.00039 | 583.81 | 588.67 | 826.05 | 713.02 |
| PNPLA8 | patatin-like phospholipase domain containing 8 | 1.3084 | 0.01227 | 273.42 | 292.5 | 315.08 | 434.51 |
| C15orf23 | chromosome 15 open reading frame 23 | 1.3062 | 0.00133 | 238.69 | 216.25 | 295.29 | 298.24 |
| NLGN1 | neuroligin 1 | 1.306 | 9.58E-06 | 183.14 | 173.13 | 237.3 | 227.89 |
| VIL2 | villin 2 (ezrin) | 1.2991 | 6.28E-05 | 2091.9 | 2134.7 | 2791.5 | 2699.8 |
| RPS6KA2 | ribosomal protein S6 kinase. 90kDa. polypeptide 2 | 1.2949 | 2.37E-06 | 1334.2 | 1338 | 1681.2 | 1780.5 |
| KCNG1 | potassium voltage-gated channel. subfamily G. member 1 | 1.2948 | 0.0021 | 429.47 | 482.66 | 573.71 | 605.73 |
| ZFAND5 | zinc finger. AN1-type domain 5 | 1.2935 | 0.00061 | 4437.4 | 4244.1 | 5645.7 | 5581.2 |
| RGS19 | regulator of G-protein signaling 19 | 1.2918 | 4.47E-05 | 192.94 | 190.94 | 240.67 | 255.43 |
| KIAA1009 | KIAA1009 | 1.2913 | 2.83E-06 | 180.77 | 177.86 | 225.92 | 237.3 |
| BBS10 | Bardet-Biedl syndrome 10 | 1.2858 | 0.00026 | 266.85 | 255.86 | 341.44 | 330.62 |
| APBB3 | amyloid beta (A4) precursor protein-binding. family B. member 3 | 1.2842 | 0.0012 | 942.94 | 878.57 | 1172 | 1165.7 |
| DSP | desmoplakin | 1.2831 | 0.00013 | 268.29 | 271.54 | 343.87 | 348.81 |
| TXNDC5 | thioredoxin domain containing 5 | 1.2829 | 0.00015 | 1738.5 | 2015 | 2488.2 | 2317 |
| VEZF1 | vascular endothelial zinc finger 1 | 1.2808 | 0.00056 | 980.45 | 891.59 | 1147.1 | 1250 |
| ACTG1 | actin. gamma 1 | 1.2799 | 0.00073 | 2141.2 | 2130.5 | 2687.4 | 2780.7 |
| HADH | hydroxyacyl-Coenzyme A dehydrogenase | 1.2789 | 6.57E-05 | 2349.2 | 2405.1 | 2847.2 | 3245.6 |
| FXYD3 | FXYD domain containing ion transport regulator 3 | 1.277 | 8.17E-05 | 180.27 | 174.45 | 229.71 | 223.24 |
| RCL1 | RNA terminal phosphate cyclase-like 1 | 1.2761 | 8.23E-06 | 421.33 | 418 | 555.8 | 516.01 |
| STK39 | serine threonine kinase 39 (STE20/SPS1 homolog. yeast) | 1.2726 | 0.01029 | 4834.1 | 3659.7 | 5474.1 | 5234.3 |
| SLC4A7 | solute carrier family 4. sodium bicarbonate cotransporter. member 7 | 1.2706 | 0.00917 | 210.2 | 169.71 | 247.42 | 232.76 |
| NFIL3 | nuclear factor. interleukin 3 regulated | 1.269 | 0.0003 | 411.84 | 396.04 | 471.53 | 557.06 |
| AZI2 | 5-azacytidine induced 2 | 1.2665 | 0.00075 | 323.61 | 336.51 | 380.7 | 458.81 |
| SLC7A8 | solute carrier family 7 (cationic amino acid transporter. y+ system). member 8 | 1.2637 | 6.17E-06 | 329.59 | 316.84 | 419.4 | 397.6 |
| SESN1 | sestrin 1 | 1.2622 | 0.00151 | 959.63 | 996.31 | 1302.6 | 1169.4 |
| PRKCE | protein kinase C. epsilon | 1.2622 | 2.87E-05 | 173.08 | 173.41 | 219.49 | 217.83 |
| CDK2AP2 | CDK2-associated protein 2 | 1.2622 | 5.14E-05 | 845.22 | 858.36 | 1087.5 | 1062.8 |
| STYK1 | serine/threonine/tyrosine kinase 1 | 1.2621 | 0.00047 | 194.87 | 165.88 | 235.08 | 219.04 |
| ADFP | adipose differentiation-related protein | 1.2612 | 0.00031 | 155.94 | 143.26 | 188.07 | 188.95 |
| SSR2 | signal sequence receptor. beta (translocon-associated protein beta) | 1.2608 | 0.01735 | 1731.4 | 2103.4 | 2276 | 2543.6 |
| ABHD6 | abhydrolase domain containing 6 | 1.2595 | 0.00072 | 295.69 | 291.02 | 386.74 | 352.96 |
| NOTCH2 | Notch homolog 2 (Drosophila) | 1.2525 | 0.04372 | 256.96 | 299.74 | 323.17 | 373.86 |
| ABCC8 | ATP-binding cassette. sub-family C (CFTR/MRP). member 8 | 1.2524 | 0.00019 | 199.74 | 192.35 | 236.95 | 254.3 |
| SMPDL3B | sphingomyelin phosphodiesterase. acid-like 3B | 1.2518 | 0.00014 | 365.1 | 384.37 | 473.02 | 464.87 |
| C12orf24 | chromosome 12 open reading frame 24 | 1.2513 | 0.00013 | 1313.7 | 1260.9 | 1580.5 | 1641 |
| CCDC6 | coiled-coil domain containing 6 | 1.2493 | 0.00261 | 1717.1 | 1783.3 | 2133.4 | 2240.3 |
| KCNH2 | potassium voltage-gated channel. subfamily H (eag-related). member 2 | 1.2477 | 1.54E-05 | 147.9 | 142.38 | 180.04 | 182.08 |
| ASCC1 | activating signal cointegrator 1 complex subunit 1 | 1.2465 | 0.00048 | 1294.9 | 1306.6 | 1563.5 | 1681.2 |
| USP10 | ubiquitin specific peptidase 10 | 1.2464 | 0.01115 | 554.79 | 486.36 | 647.45 | 647.45 |
| ADAMTS8 | ADAM metallopeptidase with thrombospondin type 1 motif. 8 | 1.2459 | 0.00507 | 147.19 | 133.61 | 180.77 | 168.87 |
| SNAI2 | snail homolog 2 (Drosophila) | 1.244 | 8.16E-05 | 170.75 | 159.34 | 211.94 | 198.66 |
| MNX1 | motor neuron and pancreas homeobox 1 | 1.2432 | 0.0013 | 243.93 | 249.11 | 285.67 | 328.73 |
| MSX2 | msh homeobox 2 | 1.2429 | 0.00017 | 143.47 | 142.37 | 170.51 | 185.05 |
| JAG1 | jagged 1 (Alagille syndrome) | 1.2428 | 2.80E-05 | 243.36 | 236.12 | 303.59 | 292.35 |
| C1orf122 | chromosome 1 open reading frame 122 | 1.2419 | 0.00605 | 1125.9 | 1063.8 | 1343.4 | 1375.1 |
| MCM7 | minichromosome maintenance complex component 7 | 1.2416 | 0.00235 | 1332 | 1614.6 | 1774.2 | 1868.6 |
| CYR61 | cysteine-rich. angiogenic inducer. 61 | 1.2416 | 7.67E-05 | 165.94 | 163.43 | 197.69 | 211.48 |
| SLC35C1 | solute carrier family 35. member C1 | 1.2387 | 0.00099 | 277.69 | 255.72 | 339.66 | 320.77 |
| KLHL2 | kelch-like 2. Mayven (Drosophila) | 1.2384 | 0.00703 | 302.88 | 269.9 | 335.91 | 373.21 |
| PRKAG2 | protein kinase. AMP-activated. gamma 2 non-catalytic subunit | 1.2379 | 5.26E-05 | 388.09 | 375.21 | 485.73 | 459.37 |
| MAP1LC3B | microtubule-associated protein 1 light chain 3 beta | 1.2369 | 0.0015 | 1482.1 | 1275.3 | 1673.3 | 1728.3 |
| LIFR | leukemia inhibitory factor receptor alpha | 1.2358 | 0.0027 | 144.16 | 142.85 | 162.61 | 193.43 |
| AKAP14 | A kinase (PRKA) anchor protein 14 | 1.2355 | 0.0002 | 119.81 | 121.62 | 142.54 | 156.05 |
| STAT3 | signal transducer and activator of transcription 3 (acute-phase response factor) | 1.2347 | 0.0003 | 390.41 | 398.36 | 507.48 | 467.17 |
| DUSP4 | dual specificity phosphatase 4 | 1.2347 | 0.0005 | 221.69 | 233.55 | 274.35 | 287.67 |
| HES6 | hairy and enhancer of split 6 (Drosophila) | 1.2337 | 0.01428 | 5198.8 | 4360.7 | 5567.7 | 6197.4 |
| KIAA1199 | KIAA1199 | 1.2334 | 0.00462 | 270.1 | 257.56 | 290.01 | 364.91 |
| EDG3 | endothelial differentiation. sphingolipid G-protein-coupled receptor. 3 | 1.2332 | 6.96E-05 | 157.33 | 155.58 | 195.46 | 190.43 |
| PHLDA2 | pleckstrin homology-like domain. family A. member 2 | 1.2322 | 0.00358 | 477.61 | 422.52 | 567.93 | 539.54 |
| C1orf198 | chromosome 1 open reading frame 198 | 1.2317 | 0.00025 | 432.85 | 484.89 | 554.4 | 574.34 |
| SAFB2 | scaffold attachment factor B2 | 1.2314 | 0.00204 | 678.51 | 623.56 | 751.33 | 853.83 |
| C1RL | complement component 1. r subcomponent-like | 1.2303 | 0.01379 | 497.38 | 387.8 | 550.26 | 530.61 |
| FKBP11 | FK506 binding protein 11. 19 kDa | 1.2295 | 0.00134 | 1226.3 | 1359.7 | 1577.7 | 1597.7 |
| TBC1D8 | TBC1 domain family. member 8 (with GRAM domain) | 1.229 | 2.89E-05 | 261.13 | 254.3 | 315.94 | 317.5 |
| ANAPC10 | anaphase promoting complex subunit 10 | 1.2277 | 0.00337 | 352.67 | 341.34 | 454.08 | 399.58 |
| TACC2 | transforming. acidic coiled-coil containing protein 2 | 1.2277 | 0.03011 | 3242.6 | 2644.8 | 3744.3 | 3452.1 |
| ELOVL1 | elongation of very long chain fatty acids (FEN1/Elo2. SUR4/Elo3. yeast)-like 1 | 1.2262 | 0.00793 | 397.11 | 412.38 | 528.34 | 466.05 |
| RNASEH1 | ribonuclease H1 | 1.2252 | 0.00035 | 573.46 | 535.49 | 670.04 | 688.02 |
| B4GALT1 | UDP-Gal:betaGlcNAc beta 1.4- galactosyltransferase. polypeptide 1 | 1.223 | 0.13016 | 500.79 | 636.5 | 672.93 | 708.48 |
| HARS | histidyl-tRNA synthetase | 1.2225 | 0.00036 | 1988.3 | 2090.6 | 2551.4 | 2435 |
| FBXO18 | F-box protein. helicase. 18 | 1.2208 | 0.00057 | 1124.2 | 1185.8 | 1378.7 | 1441.1 |
| PHYHD1 | phytanoyl-CoA dioxygenase domain containing 1 | 1.2179 | 2.76E-05 | 137.12 | 134.44 | 168.51 | 162.27 |
| PHACTR2 | phosphatase and actin regulator 2 | 1.2172 | 0.0031 | 363.81 | 339.46 | 429.94 | 425.57 |
| CLINT1 | clathrin interactor 1 | 1.2169 | 0.00948 | 1054.8 | 927.24 | 1213.1 | 1193.9 |
| LEPREL1 | leprecan-like 1 | 1.2162 | 7.30E-05 | 205.44 | 198.84 | 253.47 | 238.39 |
| PLCG1 | phospholipase C. gamma 1 | 1.2152 | 0.00671 | 380.76 | 364.74 | 448.78 | 456.95 |
| ZNF57 | zinc finger protein 57 | 1.2132 | 0.00065 | 158.69 | 168.55 | 192.42 | 204.6 |
| KIAA1324L | KIAA1324-like | 1.2131 | 0.00852 | 483.66 | 447.47 | 571.25 | 557.5 |
| HBQ1 | hemoglobin. theta 1 | 1.2124 | 0.00041 | 285.55 | 276.39 | 338.26 | 342.97 |
| JAK2 | Janus kinase 2 (a protein tyrosine kinase) | 1.2124 | 0.00384 | 250.02 | 226.07 | 289.24 | 287.24 |
| NCKIPSD | NCK interacting protein with SH3 domain | 1.2108 | 0.06593 | 462.22 | 486.49 | 521.57 | 632.11 |
| POFUT2 | protein O-fucosyltransferase 2 | 1.2101 | 0.00241 | 502.84 | 492.45 | 638.27 | 568.14 |
| SEC13 | SEC13 homolog (S. cerevisiae) | 1.2091 | 0.09242 | 603.09 | 699.08 | 732.18 | 841.86 |
| FNTB | farnesyltransferase. CAAX box. beta | 1.2089 | 0.01397 | 277.22 | 247.8 | 330.71 | 303.59 |
| MBOAT2 | membrane bound O-acyltransferase domain containing 2 | 1.2088 | 0.00417 | 458.92 | 517.95 | 579.14 | 599.75 |
| NMU | neuromedin U | 1.2036 | 0.03338 | 272.66 | 229.39 | 307.73 | 294.42 |
| PDCD10 | programmed cell death 10 | 1.2028 | 0.02602 | 1168.9 | 900.11 | 1154.3 | 1318.8 |
| C1orf112 | chromosome 1 open reading frame 112 | 1.2027 | 0.00071 | 282.94 | 266.9 | 329.92 | 331.07 |
| TUFT1 | tuftelin 1 | 1.2005 | 0.00113 | 1058.4 | 962.53 | 1187.5 | 1236.4 |
| MAN2B2 | mannosidase. alpha. class 2B. member 2 | 1.1997 | 0.00533 | 1540.8 | 1648.3 | 1851.5 | 1974.3 |
| MED10 | mediator complex subunit 10 | 1.199 | 0.0017 | 1474.1 | 1398.1 | 1705.6 | 1737.2 |
| IL13RA1 | interleukin 13 receptor. alpha 1 | 1.199 | 0.00066 | 612.4 | 613.55 | 719.23 | 751.08 |
| OCEL1 | occludin/ELL domain containing 1 | 1.1983 | 9.01E-05 | 669.13 | 667.93 | 773.63 | 829.61 |
| CRYAB | crystallin. alpha B | 1.1975 | 0.0018 | 229.6 | 227.8 | 254.69 | 294.49 |
| SCRG1 | scrapie responsive protein 1 | 1.1966 | 0.00148 | 144.29 | 151.76 | 172.27 | 182.01 |
| HNRPD | heterogeneous nuclear ribonucleoprotein D (AU-rich element RNA binding protein 1. 37kDa) | 1.1951 | 0.01035 | 2574.8 | 2789.1 | 3188.1 | 3217.5 |
| LZTR1 | leucine-zipper-like transcription regulator 1 | 1.1928 | 0.00123 | 985.44 | 930.17 | 1145.1 | 1138.9 |
| NAGK | N-acetylglucosamine kinase | 1.1908 | 0.00307 | 840.75 | 854.1 | 1028.2 | 990.38 |
| TFPI | tissue factor pathway inhibitor (lipoprotein-associated coagulation inhibitor) | 1.19 | 0.00666 | 3447.6 | 3405.5 | 4217.5 | 3942 |
| MGMT | O-6-methylguanine-DNA methyltransferase | 1.1884 | 0.06372 | 854.1 | 986.91 | 1034.2 | 1151.2 |
| NFKBIA | nuclear factor of kappa light polypeptide gene enhancer in B-cells inhibitor. alpha | 1.185 | 0.01275 | 4414.2 | 3870.5 | 5057.9 | 4743.5 |
| PACSIN2 | protein kinase C and casein kinase substrate in neurons 2 | 1.1844 | 0.00143 | 5295.2 | 4972.7 | 6172.3 | 5984 |
| SIPA1L2 | signal-induced proliferation-associated 1 like 2 | 1.1835 | 0.00503 | 601.11 | 556.39 | 641.98 | 729.67 |
| EDN1 | endothelin 1 | 1.1832 | 8.56E-05 | 138.6 | 137 | 162.86 | 163.21 |
| KIAA1191 | KIAA1191 | 1.1827 | 0.01514 | 2025.1 | 2022.7 | 2321.7 | 2467.9 |
| SORT1 | sortilin 1 | 1.1826 | 0.00622 | 280.3 | 304.12 | 353.17 | 337.55 |
| LOC203547 | hypothetical protein LOC203547 | 1.1798 | 0.00496 | 1923.9 | 1918.1 | 2231.3 | 2301.8 |
| DGKD | diacylglycerol kinase. delta 130kDa | 1.1779 | 0.00029 | 192.1 | 182.9 | 218.52 | 223.1 |
| FEM1C | fem-1 homolog c (C. elegans) | 1.1716 | 0.00752 | 665.5 | 613.77 | 770.4 | 727.79 |
| TBC1D22A | TBC1 domain family. member 22A | 1.1716 | 0.00089 | 685.85 | 697.67 | 818.84 | 802.11 |
| CHCHD7 | coiled-coil-helix-coiled-coil-helix domain containing 7 | 1.17 | 0.00139 | 611.54 | 591.88 | 668.77 | 740.87 |
| GBE1 | glucan (1.4-alpha-). branching enzyme 1 (glycogen branching enzyme. Andersen disease. glycogen storage disease type IV) | 1.17 | 0.01873 | 672.17 | 636.03 | 721.7 | 810.86 |
| PGM3 | phosphoglucomutase 3 | 1.169 | 0.00285 | 636.27 | 626.33 | 740.5 | 735.43 |
| H2AFX | H2A histone family. member X | 1.1657 | 0.06276 | 657.38 | 558.29 | 736.49 | 677.19 |
| MTMR9 | myotubularin related protein 9 | 1.1645 | 0.03252 | 622.61 | 595.98 | 682.36 | 737.42 |
| C2orf30 | chromosome 2 open reading frame 30 | 1.1633 | 0.10011 | 1342.2 | 1136.9 | 1540.8 | 1340.3 |
| TMEM165 | transmembrane protein 165 | 1.163 | 0.00564 | 443 | 419.4 | 485.99 | 517.1 |
| ELL | elongation factor RNA polymerase II | 1.1595 | 0.03131 | 272.37 | 284.35 | 301.61 | 345.22 |
| CDKN2B | cyclin-dependent kinase inhibitor 2B (p15. inhibits CDK4) | 1.1557 | 0.01298 | 156.66 | 151.81 | 172.75 | 183.88 |
| GIPC1 | GIPC PDZ domain containing family. member 1 | 1.1531 | 0.00284 | 215.51 | 219.52 | 259.48 | 242.42 |
| ANGPT2 | angiopoietin 2 | 1.1517 | 0.009 | 202.69 | 176.7 | 215.39 | 220.55 |
| SLC26A3 | solute carrier family 26. member 3 | 1.1511 | 0.01341 | 174.21 | 155.32 | 193.33 | 185.44 |
| CDKN1A | cyclin-dependent kinase inhibitor 1A (p21. Cip1) | 1.151 | 0.00703 | 6536.1 | 6116.1 | 7503.4 | 7058.2 |
| EP400 | E1A binding protein p400 | 1.1497 | 0.0043 | 283.98 | 271.79 | 325.39 | 313.53 |
| ZYX | zyxin | 1.1478 | 0.04652 | 936.38 | 915.15 | 1007.6 | 1120.4 |
| SLC3A2 | solute carrier family 3 (activators of dibasic and neutral amino acid transport). member 2 | 1.1474 | 0.00973 | 2867.7 | 2890.5 | 3396.2 | 3213.2 |
| HSP90AB1 | heat shock protein 90kDa alpha (cytosolic). class B member 1 | 1.1461 | 0.01308 | 3669.1 | 4078.9 | 4476.9 | 4391.3 |
| RPS29 | ribosomal protein S29 | 1.1421 | 0.03127 | 1795.7 | 1630.7 | 1951.6 | 1957.1 |
| ABCC4 | ATP-binding cassette. sub-family C (CFTR/MRP). member 4 | 1.1418 | 0.16122 | 3311.6 | 3209.6 | 4054.8 | 3417.5 |
| KIAA0513 | KIAA0513 | 1.1417 | 0.02977 | 264.85 | 249.79 | 323.31 | 266.72 |
| RWDD2A | RWD domain containing 2A | 1.1411 | 0.03064 | 1583 | 1376 | 1714.3 | 1654.6 |
| PCSK6 | proprotein convertase subtilisin/kexin type 6 | 1.1407 | 0.00115 | 154.75 | 148.72 | 171.32 | 174.79 |
| TMEM120A | transmembrane protein 120A | 1.1406 | 0.06731 | 566.53 | 557.06 | 643.22 | 638.27 |
| SLC22A18 | solute carrier family 22 (organic cation transporter). member 18 | 1.1391 | 0.08803 | 386.24 | 385.53 | 471.69 | 409.64 |
| IFRD1 | interferon-related developmental regulator 1 | 1.1357 | 0.04379 | 1513.8 | 1250.7 | 1478.9 | 1651.2 |
| MYL5 | myosin. light chain 5. regulatory | 1.1355 | 0.00708 | 1042.4 | 1128.3 | 1258 | 1205.5 |
| BCAP31 | B-cell receptor-associated protein 31 | 1.1338 | 0.01334 | 3659.7 | 3903.7 | 4327.3 | 4244.1 |
| LIN7B | lin-7 homolog B (C. elegans) | 1.1338 | 0.00799 | 254.97 | 262.03 | 308.89 | 278.04 |
| C14orf132 | chromosome 14 open reading frame 132 | 1.1327 | 0.07132 | 387.49 | 338.6 | 391.4 | 430.07 |
| PARP4 | poly (ADP-ribose) polymerase family. member 4 | 1.1315 | 0.02567 | 2358.9 | 2191.7 | 2548.5 | 2597.4 |
| TBC1D19 | TBC1 domain family. member 19 | 1.1312 | 0.00347 | 258.33 | 263.62 | 295.39 | 295.04 |
| BSPRY | B-box and SPRY domain containing | 1.1306 | 0.00093 | 643.22 | 627.52 | 708.48 | 728.24 |
| SDC4 | syndecan 4 | 1.1292 | 0.04455 | 930.61 | 1048.4 | 1132.6 | 1098.4 |
| TAF5 | TAF5 RNA polymerase II. TATA box binding protein (TBP)-associated factor. 100kDa | 1.1289 | 0.00903 | 232.31 | 240.93 | 274.43 | 259.91 |
| SPSB3 | splA/ryanodine receptor domain and SOCS box containing 3 | 1.1286 | 0.00653 | 832.37 | 846.64 | 976.85 | 918.9 |
| IQGAP1 | IQ motif containing GTPase activating protein 1 | 1.1261 | 0.24786 | 241.65 | 250.52 | 261.9 | 293.15 |
| SAR1B | SAR1 gene homolog B (S. cerevisiae) | 1.1257 | 0.10503 | 3202.7 | 3058 | 3683.4 | 3369.1 |
| SLC6A3 | solute carrier family 6 (neurotransmitter transporter. dopamine). member 3 | 1.1232 | 0.00139 | 136.73 | 135.48 | 154.02 | 151.73 |
| HMGCS2 | 3-hydroxy-3-methylglutaryl-Coenzyme A synthase 2 (mitochondrial) | 1.1221 | 0.01289 | 663.97 | 679.82 | 761.09 | 746.76 |
| CDR2L | cerebellar degeneration-related protein 2-like | 1.1213 | 0.01225 | 260.78 | 249.51 | 287.17 | 284.89 |
| IMPDH2 | IMP (inosine monophosphate) dehydrogenase 2 | 1.1208 | 0.02143 | 4704.8 | 4926.2 | 5746 | 5067.3 |
| ARRDC1 | arrestin domain containing 1 | 1.1206 | 0.04197 | 318.62 | 303.3 | 345.67 | 351.04 |
| PRMT1 | protein arginine methyltransferase 1 | 1.1196 | 0.00427 | 2660 | 2642 | 3058 | 2881 |
| PEA15 | phosphoprotein enriched in astrocytes 15 | 1.1194 | 0.03732 | 1785.8 | 1618.5 | 1793.4 | 2019.5 |
| MOSC1 | MOCO sulphurase C-terminal domain containing 1 | 1.1186 | 0.06133 | 1848.1 | 1763.2 | 2156.6 | 1890.4 |
| ZXDB | zinc finger. X-linked. duplicated B | 1.1163 | 0.21092 | 428.18 | 397.97 | 426.15 | 498.27 |
| GDPD5 | glycerophosphodiester phosphodiesterase domain containing 5 | 1.1161 | 0.01135 | 154.05 | 152.96 | 169.06 | 173.62 |
| P4HA2 | procollagen-proline. 2-oxoglutarate 4-dioxygenase (proline 4-hydroxylase). alpha polypeptide II | 1.1137 | 0.02783 | 519.11 | 484.31 | 560.69 | 556.12 |
| SLC2A5 | solute carrier family 2 (facilitated glucose/fructose transporter). member 5 | 1.1112 | 0.0144 | 126.31 | 120.2 | 131.84 | 142.2 |
| C14orf147 | chromosome 14 open reading frame 147 | 1.1098 | 0.03078 | 646.21 | 582.52 | 684.77 | 677.01 |
| RELN | reelin | 1.1095 | 0.03029 | 284.35 | 279.16 | 293.87 | 332.5 |
| ITSN1 | intersectin 1 (SH3 domain protein) | 1.108 | 0.0338 | 523.72 | 566.32 | 598.51 | 608.37 |
| PLXNA1 | plexin A1 | 1.1066 | 0.05555 | 217.07 | 202.58 | 241.44 | 223.05 |
| PIGG | phosphatidylinositol glycan anchor biosynthesis. class G | 1.1064 | 0.13227 | 287.67 | 292 | 348.23 | 295.29 |
| SERP1 | stress-associated endoplasmic reticulum protein 1 | 1.1038 | 0.20835 | 1012.2 | 1148.1 | 1100.2 | 1287 |
| ZNF541 | zinc finger protein 541 | 1.1027 | 0.00738 | 161.75 | 157.27 | 174.75 | 177.01 |
| TST | thiosulfate sulfurtransferase (rhodanese) | 1.1003 | 0.04125 | 3339.3 | 3378 | 3591.1 | 3802.6 |
| FADS3 | fatty acid desaturase 3 | 1.0998 | 0.17637 | 582.33 | 566.73 | 652.75 | 611.54 |
| SYNCRIP | synaptotagmin binding. cytoplasmic RNA interacting protein | 1.0988 | 0.11486 | 1191 | 1317.8 | 1320.5 | 1435.1 |
| CPEB4 | cytoplasmic polyadenylation element binding protein 4 | 1.0985 | 0.16654 | 266.48 | 264.12 | 292.64 | 290.24 |
| SLC1A3 | solute carrier family 1 (glial high affinity glutamate transporter). member 3 | 1.098 | 0.02155 | 127.6 | 125.81 | 136.47 | 141.82 |
| AHSA2 | AHA1. activator of heat shock 90kDa protein ATPase homolog 2 (yeast) | 1.0976 | 0.34402 | 526.85 | 457.86 | 464.18 | 626.06 |
| SLC22A16 | solute carrier family 22 (organic cation transporter). member 16 | 1.0973 | 0.01165 | 142.95 | 142.98 | 158.31 | 155.45 |
| IMPDH1 | IMP (inosine monophosphate) dehydrogenase 1 | 1.0969 | 0.07442 | 1317.1 | 1259 | 1502.6 | 1327.8 |
| C9orf46 | chromosome 9 open reading frame 46 | 1.0937 | 0.19113 | 1914.6 | 2332.1 | 2502.3 | 2134.7 |
| CALD1 | caldesmon 1 | 1.0937 | 0.3457 | 193.06 | 211.52 | 199.6 | 244.72 |
| EBPL | emopamil binding protein-like | 1.0926 | 0.2132 | 2304.1 | 2425.7 | 2817.4 | 2368.3 |
| GFPT1 | glutamine-fructose-6-phosphate transaminase 1 | 1.092 | 0.15604 | 2667.7 | 2334.2 | 2725.1 | 2725.1 |
| ZFAND2A | zinc finger. AN1-type domain 2A | 1.092 | 0.16063 | 1842.2 | 1679.1 | 1799.2 | 2050.1 |
| PPP1R13B | protein phosphatase 1. regulatory (inhibitor) subunit 13B | 1.0896 | 0.1765 | 631.86 | 597.66 | 707.28 | 633.93 |
| MKNK1 | MAP kinase interacting serine/threonine kinase 1 | 1.0893 | 0.05583 | 473.48 | 443 | 481.06 | 517.34 |
| NRBP2 | nuclear receptor binding protein 2 | 1.0863 | 0.23079 | 291.61 | 232.82 | 284.1 | 282 |
| GALNTL4 | UDP-N-acetyl-alpha-D-galactosamine:polypeptide N-acetylgalactosaminyltransferase-like 4 | 1.0853 | 0.18695 | 324.28 | 319.45 | 377.94 | 322.83 |
| NAP1L4 | nucleosome assembly protein 1-like 4 | 1.085 | 0.10525 | 1876.3 | 1805.7 | 2141.2 | 1862.9 |
| TP53I3 | tumor protein p53 inducible protein 3 | 1.0833 | 0.12948 | 251.38 | 230.03 | 272.28 | 249.22 |
| STXBP5 | syntaxin binding protein 5 (tomosyn) | 1.0832 | 0.0807 | 337.55 | 314.28 | 345.59 | 360.21 |
| TGFBR3 | transforming growth factor. beta receptor III | 1.0823 | 0.21771 | 225.06 | 222.37 | 243.36 | 240.88 |
| NCAM2 | neural cell adhesion molecule 2 | 1.0806 | 0.22089 | 3936 | 3927.2 | 4565.8 | 3953.5 |
| TPM1 | tropomyosin 1 (alpha) | 1.0797 | 0.11037 | 1697.1 | 1739.6 | 1855.9 | 1854.5 |
| YWHAQ | tyrosine 3-monooxygenase/tryptophan 5-monooxygenase activation protein. theta polypeptide | 1.0796 | 0.24235 | 8996.7 | 7958.2 | 8545.8 | 9765.9 |
| TNFRSF10B | tumor necrosis factor receptor superfamily. member 10b | 1.0789 | 0.21713 | 2762.6 | 2608 | 3109.7 | 2696.8 |
| PTTG1IP | pituitary tumor-transforming 1 interacting protein | 1.0785 | 0.06015 | 1067.4 | 1152.4 | 1224.8 | 1168.1 |
| MYH13 | myosin. heavy chain 13. skeletal muscle | 1.0779 | 0.02535 | 133.99 | 127.99 | 141.29 | 141.01 |
| ATP1A2 | ATPase. Na+/K+ transporting. alpha 2 (+) polypeptide | 1.0771 | 0.03688 | 145.29 | 140.93 | 147.29 | 161.28 |
| ATG16L1 | ATG16 autophagy related 16-like 1 (S. cerevisiae) | 1.0729 | 0.3968 | 216.1 | 237.26 | 220.35 | 267.85 |
| NAT2 | N-acetyltransferase 2 (arylamine N-acetyltransferase) | 1.0677 | 0.45372 | 168.23 | 172.84 | 165.24 | 200.61 |
| CETN2 | centrin. EF-hand protein. 2 | 1.0654 | 0.06336 | 3492.3 | 3574.3 | 3736.7 | 3791.9 |
| IKBKG | inhibitor of kappa light polypeptide gene enhancer in B-cells. kinase gamma | 1.0648 | 0.21377 | 1165.7 | 1039.9 | 1148.1 | 1197.1 |
| ATP1B1 | ATPase. Na+/K+ transporting. beta 1 polypeptide | 1.0621 | 0.38482 | 1222.8 | 1276.7 | 1444.1 | 1219.4 |
| DHRS13 | dehydrogenase/reductase (SDR family) member 13 | 1.0617 | 0.27876 | 568.63 | 626.06 | 644.97 | 622.22 |
| IL20RB | interleukin 20 receptor beta | 1.0605 | 0.04485 | 151.42 | 145.96 | 156.1 | 159.24 |
| MYH9 | myosin. heavy chain 9. non-muscle | 1.0593 | 0.26701 | 5706.3 | 5185.2 | 5941 | 5588.4 |
| RBMX | RNA binding motif protein. X-linked | 1.0574 | 0.25754 | 1398.8 | 1313.7 | 1383.2 | 1485.4 |
| PBEF1 | pre-B-cell colony enhancing factor 1 | 1.0554 | 0.12525 | 660.05 | 647.45 | 677.19 | 702.93 |
| PPA2 | pyrophosphatase (inorganic) 2 | 1.0552 | 0.51705 | 1438.4 | 1643.9 | 1524.6 | 1727 |
| SPON2 | spondin 2. extracellular matrix protein | 1.0537 | 0.48634 | 697.23 | 735.43 | 797.26 | 714.05 |
| C9orf140 | chromosome 9 open reading frame 140 | 1.0529 | 0.42754 | 387.17 | 379.71 | 414.17 | 393.51 |
| TMSB10 | thymosin. beta 10 | 1.0522 | 0.26212 | 3456.2 | 3265.3 | 3432.2 | 3640.2 |
| MGC14376 | hypothetical protein MGC14376 | 1.0514 | 0.54635 | 674.57 | 480.73 | 599 | 598.51 |
| SLC22A5 | solute carrier family 22 (organic cation transporter). member 5 | 1.0514 | 0.1209 | 503.02 | 516.53 | 533.35 | 538.54 |
| NPC1 | Niemann-Pick disease. type C1 | 1.0485 | 0.48804 | 784.35 | 704.63 | 807.66 | 752.33 |
| PTPLA | protein tyrosine phosphatase-like (proline instead of catalytic arginine). member A | 1.0473 | 0.19595 | 150.47 | 143.09 | 152.06 | 155.31 |
| FIG4 | FIG4 homolog (S. cerevisiae) | 1.0472 | 0.38123 | 524.77 | 458.81 | 538.11 | 490.66 |
| C21orf34 | chromosome 21 open reading frame 34 | 1.046 | 0.4534 | 1591.8 | 1627.8 | 1669.4 | 1698.2 |
| FNBP1L | formin binding protein 1-like | 1.0436 | 0.55091 | 1327.8 | 1150.5 | 1273.3 | 1306.6 |
| CREG1 | cellular repressor of E1A-stimulated genes 1 | 1.0427 | 0.56008 | 1376 | 1498.3 | 1625.8 | 1378.7 |
| IGF2BP2 | insulin-like growth factor 2 mRNA binding protein 2 | 1.0426 | 0.3578 | 151.76 | 147.69 | 161.32 | 151.03 |
| PIGH | phosphatidylinositol glycan anchor biosynthesis. class H | 1.0424 | 0.50692 | 355.22 | 399.09 | 372.19 | 413.91 |
| ABCA4 | ATP-binding cassette. sub-family A (ABC1). member 4 | 1.0419 | 0.31793 | 156 | 137.49 | 148.06 | 157.28 |
| PNLIPRP3 | pancreatic lipase-related protein 3 | 1.0411 | 0.46564 | 153.08 | 140.14 | 151.87 | 153.09 |
| TPD52L1 | tumor protein D52-like 1 | 1.041 | 0.59895 | 2657.7 | 2438.6 | 2727.8 | 2574.8 |
| TGIF2 | TGFB-induced factor homeobox 2 | 1.0409 | 0.34449 | 271.43 | 295.87 | 290.89 | 299.11 |
| CENPN | centromere protein N | 1.0397 | 0.36867 | 5390.1 | 5325 | 5826.7 | 5325 |
| PPP1R15A | protein phosphatase 1. regulatory (inhibitor) subunit 15A | 1.0392 | 0.46797 | 364.48 | 349.04 | 359.2 | 382.52 |
| PRODH | proline dehydrogenase (oxidase) 1 | 1.0367 | 0.27041 | 123.46 | 127.11 | 127.53 | 132.25 |
| SLC9A1 | solute carrier family 9 (sodium/hydrogen exchanger). member 1 (antiporter. Na+/H+. amiloride sensitive) | 1.0367 | 0.44656 | 2861.7 | 2937.9 | 3024.9 | 2986.9 |
| C6orf108 | chromosome 6 open reading frame 108 | 1.0355 | 0.66027 | 2945.9 | 3539.8 | 3549.5 | 3149.9 |
| C6orf81 | chromosome 6 open reading frame 81 | 1.0339 | 0.37879 | 175.67 | 170.28 | 186.17 | 171.77 |
| WDR41 | WD repeat domain 41 | 1.0313 | 0.58558 | 638.46 | 565.33 | 585.39 | 655.76 |
| D4S234E | DNA segment on chromosome 4 (unique) 234 expressed sequence | 1.0291 | 0.54 | 207.99 | 229.36 | 227.35 | 222.22 |
| FBXL17 | F-box and leucine-rich repeat protein 17 | 1.029 | 0.72075 | 477.9 | 507.69 | 585.22 | 438.99 |
| FOXC1 | forkhead box C1 | 1.0263 | 0.43308 | 156.07 | 141.43 | 155.25 | 149.77 |
| RAB32 | RAB32. member RAS oncogene family | 1.0244 | 0.70862 | 341.84 | 322.93 | 323.25 | 358.41 |
| GPR1 | G protein-coupled receptor 1 | 1.0228 | 0.60975 | 206.33 | 206.33 | 196.31 | 226.86 |
| C19orf48 | chromosome 19 open reading frame 48 | 1.0181 | 0.67845 | 4491.6 | 4657 | 4584.5 | 4728.9 |
| HSD17B8 | hydroxysteroid (17-beta) dehydrogenase 8 | 1.0166 | 0.74835 | 1479.8 | 1680.1 | 1609.4 | 1596.6 |
| PRUNE2 | prune homolog 2 (Drosophila) | 1.0164 | 0.75697 | 2851.4 | 2570.1 | 2894.1 | 2615.9 |
| LOC388610 | hypothetical LOC388610 | 1.0149 | 0.72503 | 351.25 | 370.82 | 366.57 | 366 |
| MSX1 | msh homeobox 1 | 1.0134 | 0.85891 | 1487.3 | 1399.7 | 1454 | 1470.4 |
| ATF3 | activating transcription factor 3 | 1.0119 | 0.86726 | 3260.6 | 2621.6 | 2887.6 | 3031.1 |
| SCARB1 | scavenger receptor class B. member 1 | 1.0118 | 0.80282 | 2730.3 | 2773.1 | 2953.7 | 2624.3 |
| TRIOBP | TRIO and F-actin binding protein | 1.0106 | 0.75958 | 445.32 | 450.29 | 439.22 | 466.27 |
| CLDN3 | claudin 3 | 1.0099 | 0.85078 | 919.42 | 958.37 | 924.62 | 971.87 |
| DECR1 | 2.4-dienoyl CoA reductase 1. mitochondrial | 1.0098 | 0.88358 | 3378 | 3769.7 | 3409.5 | 3808.8 |
| SLC45A3 | solute carrier family 45. member 3 | 1.0088 | 0.87477 | 952 | 976.51 | 1058.4 | 893.87 |
| EPHX2 | epoxide hydrolase 2. cytoplasmic | 1.0082 | 0.85764 | 711.36 | 779.52 | 771.01 | 731.06 |
| TMTC4 | transmembrane and tetratricopeptide repeat containing 4 | 1.0072 | 0.88662 | 574.58 | 571.44 | 588.42 | 566.06 |
| ELF1 | E74-like factor 1 (ets domain transcription factor) | 1.0066 | 0.87088 | 1475.9 | 1385.8 | 1427.1 | 1452.1 |
| MT1X | metallothionein 1X | 1.0065 | 0.93482 | 3802.6 | 3256.5 | 3749.4 | 3346 |
| SCGN | secretagogin. EF-hand calcium binding protein | 1.0055 | 0.88081 | 143.25 | 135.8 | 138.17 | 142.33 |
| GLIPR1 | GLI pathogenesis-related 1 (glioma) | 1.0055 | 0.86074 | 140.83 | 144.63 | 148.28 | 138.87 |
| NUDT5 | nudix (nucleoside diphosphate linked moiety X)-type motif 5 | 1.0026 | 0.96205 | 2206 | 2398.9 | 2312.9 | 2300.1 |
| SELM | selenoprotein M | 1.0018 | 0.97971 | 1739.6 | 1944.4 | 1976.8 | 1717.1 |
| NME3 | non-metastatic cells 3. protein expressed in | 1.0013 | 0.98429 | 1576.3 | 1669.4 | 1728.3 | 1526.6 |
| FSTL3 | follistatin-like 3 (secreted glycoprotein) | -1.0014 | 0.97972 | 176.02 | 181.82 | 175.14 | 182.22 |
| ACPP | acid phosphatase. prostate | -1.0018 | 0.97476 | 548.98 | 625.1 | 600.82 | 569.08 |
| PROS1 | protein S (alpha) | -1.0022 | 0.95904 | 281.01 | 264.47 | 280.91 | 263.4 |
| TGM1 | transglutaminase 1 (K polypeptide epidermal type I. protein-glutamine-gamma-glutamyltransferase) | -1.0058 | 0.90881 | 156.95 | 157.88 | 146.28 | 167.45 |
| ACTG2 | actin. gamma 2. smooth muscle. enteric | -1.0103 | 0.74828 | 153.07 | 155.15 | 153.25 | 151.81 |
| ACY1 | aminoacylase 1 | -1.0104 | 0.81489 | 1780.5 | 1934.8 | 1883.9 | 1791.3 |
| C1orf80 | chromosome 1 open reading frame 80 | -1.0107 | 0.87312 | 339.31 | 422.4 | 396.43 | 353.95 |
| CALCB | calcitonin-related polypeptide. beta | -1.011 | 0.7109 | 141.34 | 145.1 | 141.84 | 141.47 |
| AHNAK | AHNAK nucleoprotein | -1.0121 | 0.86902 | 709.43 | 604.22 | 628.81 | 665.5 |
| ACSM3 | acyl-CoA synthetase medium-chain family member 3 | -1.0131 | 0.86536 | 704.32 | 644.41 | 687.78 | 642.98 |
| TPM4 | tropomyosin 4 | -1.0138 | 0.80093 | 217.76 | 215.47 | 212.29 | 215.02 |
| C6orf32 | chromosome 6 open reading frame 32 | -1.0148 | 0.72986 | 548.61 | 594.9 | 592.96 | 534.44 |
| CKMT1A | creatine kinase. mitochondrial 1A | -1.0177 | 0.61224 | 453.08 | 487.61 | 456.95 | 466.85 |
| SRM | spermidine synthase | -1.0179 | 0.81273 | 1279.6 | 1415.2 | 1423.5 | 1227.8 |
| DNM1L | dynamin 1-like | -1.0192 | 0.7981 | 635.71 | 589.57 | 623.23 | 578.96 |
| PRKCD | protein kinase C. delta | -1.0206 | 0.55781 | 2023.9 | 1864.3 | 1891.8 | 1914.6 |
| LEO1 | Leo1. Paf1/RNA polymerase II complex component. homolog (S. cerevisiae) | -1.0207 | 0.74175 | 621.73 | 603.09 | 617.47 | 582.89 |
| ACTN1 | actinin. alpha 1 | -1.0209 | 0.80394 | 1701.2 | 1784.6 | 1508.4 | 1931.1 |
| TFDP1 | transcription factor Dp-1 | -1.0238 | 0.57868 | 732.18 | 781.38 | 760.22 | 718 |
| ARG2 | arginase. type II | -1.0243 | 0.54779 | 386.86 | 409.22 | 406.55 | 371.14 |
| PFKM | phosphofructokinase. muscle | -1.0272 | 0.51644 | 844.35 | 888.67 | 891.59 | 797.67 |
| MCCC2 | methylcrotonoyl-Coenzyme A carboxylase 2 (beta) | -1.0283 | 0.5793 | 406.11 | 390.81 | 399.7 | 375.54 |
| FLJ40852 | hypothetical protein FLJ40852 | -1.0293 | 0.47466 | 222.65 | 216.35 | 218.55 | 208.02 |
| AUH | AU RNA binding protein/enoyl-Coenzyme A hydratase | -1.0297 | 0.44954 | 991.86 | 925.68 | 943.4 | 917.85 |
| IYD | iodotyrosine deiodinase | -1.0298 | 0.38551 | 205.47 | 211.45 | 201.71 | 203.09 |
| CPNE3 | copine III | -1.0321 | 0.56327 | 6484.7 | 6263.9 | 6342.1 | 6012.4 |
| DERA | 2-deoxyribose-5-phosphate aldolase homolog (C. elegans) | -1.0344 | 0.36722 | 1955.7 | 2002.8 | 1882.6 | 1944.4 |
| TMCO3 | transmembrane and coiled-coil domains 3 | -1.0348 | 0.38797 | 6381.3 | 5862.6 | 5897.2 | 5924.8 |
| RDH11 | retinol dehydrogenase 11 (all-trans/9-cis/11-cis) | -1.0349 | 0.65475 | 6149 | 6606.4 | 6055.7 | 6263.9 |
| ATIC | 5-aminoimidazole-4-carboxamide ribonucleotide formyltransferase/IMP cyclohydrolase | -1.0381 | 0.3656 | 7587.7 | 7229.6 | 7276.9 | 6995.1 |
| WDR1 | WD repeat domain 1 | -1.0397 | 0.43016 | 3772.8 | 3772.8 | 3402.2 | 3870.5 |
| CPNE1 | copine I | -1.0399 | 0.44993 | 800.26 | 746.3 | 800.47 | 689.91 |
| CXCR7 | chemokine (C-X-C motif) receptor 7 | -1.0425 | 0.47497 | 2111.9 | 2276 | 2012.1 | 2198.1 |
| DCUN1D3 | DCN1. defective in cullin neddylation 1. domain containing 3 (S. cerevisiae) | -1.0483 | 0.40888 | 484.46 | 432.43 | 436.95 | 436.32 |
| GAMT | guanidinoacetate N-methyltransferase | -1.0493 | 0.32 | 2416.8 | 2436.9 | 2486.6 | 2151.2 |
| PRR7 | proline rich 7 (synaptic) | -1.052 | 0.28359 | 420.88 | 429.94 | 428.18 | 381.83 |
| SOX8 | SRY (sex determining region Y)-box 8 | -1.0522 | 0.35919 | 449.47 | 459.94 | 453.58 | 411.65 |
| CYB5A | cytochrome b5 type A (microsomal) | -1.0543 | 0.23238 | 7563.8 | 8677 | 7762.4 | 7606.5 |
| MYC | v-myc myelocytomatosis viral oncogene homolog (avian) | -1.0559 | 0.35051 | 2215.9 | 2510 | 2123.7 | 2349.2 |
| MT1G | metallothionein 1G | -1.0564 | 0.42416 | 445.45 | 387.49 | 399.91 | 386.74 |
| ACTA2 | actin. alpha 2. smooth muscle. aorta | -1.0577 | 0.3693 | 708.14 | 609.82 | 568.63 | 678.85 |
| C5orf32 | chromosome 5 open reading frame 32 | -1.0591 | 0.37961 | 593.9 | 601.32 | 574.58 | 554.12 |
| 3-Sep | septin 3 | -1.0594 | 0.22432 | 555.12 | 547.51 | 512.87 | 527.98 |
| TNFRSF10D | tumor necrosis factor receptor superfamily. member 10d. decoy with truncated death domain | -1.0606 | 0.3086 | 260.72 | 267.85 | 253.31 | 245.07 |
| TRIB3 | tribbles homolog 3 (Drosophila) | -1.0612 | 0.37992 | 8151.8 | 7112.7 | 7720.4 | 6669.3 |
| E2F5 | E2F transcription factor 5. p130-binding | -1.0627 | 0.20381 | 370.69 | 369.5 | 360.32 | 336.59 |
| CABC1 | chaperone. ABC1 activity of bc1 complex homolog (S. pombe) | -1.0627 | 0.23894 | 903.64 | 1058.4 | 929.08 | 911.54 |
| FOLH1 | folate hydrolase (prostate-specific membrane antigen) 1 | -1.0649 | 0.52562 | 1049.5 | 1211.6 | 968.66 | 1157.7 |
| ZNF323 | zinc finger protein 323 | -1.0654 | 0.20947 | 307.55 | 295.29 | 284.83 | 280.91 |
| THOP1 | thimet oligopeptidase 1 | -1.0683 | 0.22609 | 891.59 | 851.57 | 828.15 | 803.33 |
| ZNF511 | zinc finger protein 511 | -1.0683 | 0.1618 | 2554.8 | 2498.9 | 2491.6 | 2245.2 |
| CGNL1 | cingulin-like 1 | -1.0692 | 0.10913 | 623.82 | 601.57 | 576.05 | 569.85 |
| HMOX1 | heme oxygenase (decycling) 1 | -1.0697 | 0.19254 | 197.85 | 177.46 | 169.99 | 180.51 |
| BOP1 | block of proliferation 1 | -1.075 | 0.22514 | 1725 | 1826.9 | 1626.8 | 1676.3 |
| CXCL16 | chemokine (C-X-C motif) ligand 16 | -1.0753 | 0.14317 | 1070.4 | 1115.2 | 1089.4 | 947.64 |
| LRRN1 | leucine rich repeat neuronal 1 | -1.0757 | 0.25146 | 343.58 | 360.12 | 346.08 | 308.97 |
| SDC1 | syndecan 1 | -1.0764 | 0.04475 | 498.83 | 469.7 | 459.37 | 440.19 |
| C4orf18 | chromosome 4 open reading frame 18 | -1.0765 | 0.38672 | 1293.1 | 1519.9 | 1334.2 | 1271 |
| DKC1 | dyskeratosis congenita 1. dyskerin | -1.0805 | 0.13468 | 2435 | 2364.5 | 2308.5 | 2136.1 |
| HOMER2 | homer homolog 2 (Drosophila) | -1.082 | 0.10534 | 1269.5 | 1246.2 | 1142.1 | 1183 |
| VARS | valyl-tRNA synthetase | -1.0841 | 0.05686 | 2064.8 | 2289.4 | 1993.3 | 2017.6 |
| CACYBP | calcyclin binding protein | -1.0869 | 0.091 | 778.05 | 709.78 | 715.45 | 653.36 |
| JAG2 | jagged 2 | -1.0885 | 0.10373 | 1063.8 | 1054.5 | 1017.9 | 930.17 |
| EFNB2 | ephrin-B2 | -1.0887 | 0.31272 | 1100.2 | 952.95 | 950.57 | 930.61 |
| WDR57 | WD repeat domain 57 (U5 snRNP specific) | -1.0938 | 0.10352 | 931.65 | 994.46 | 935.67 | 827.66 |
| CMTM7 | CKLF-like MARVEL transmembrane domain containing 7 | -1.0941 | 0.05831 | 560.3 | 498.58 | 511.53 | 456.24 |
| CCND1 | cyclin D1 | -1.0944 | 0.08047 | 3874.9 | 3728.9 | 3564.9 | 3384.1 |
| APOD | apolipoprotein D | -1.0947 | 0.14266 | 636.5 | 619.78 | 597.66 | 550.85 |
| RPL13A | ribosomal protein L13a | -1.0954 | 0.22819 | 12700 | 14770 | 12135 | 12881 |
| MRPL24 | mitochondrial ribosomal protein L24 | -1.0955 | 0.07516 | 4265.3 | 4636.9 | 4205.6 | 3918.5 |
| CDK4 | cyclin-dependent kinase 4 | -1.0992 | 0.05573 | 1979.5 | 2070.6 | 1785.8 | 1899.8 |
| RRAS | related RAS viral (r-ras) oncogene homolog | -1.1006 | 0.03971 | 816.98 | 856.99 | 747.16 | 773.63 |
| TNFRSF12A | tumor necrosis factor receptor superfamily. member 12A | -1.1027 | 0.32941 | 333.16 | 323.17 | 289.11 | 306.27 |
| C1orf128 | chromosome 1 open reading frame 128 | -1.1039 | 0.01253 | 734.01 | 764.01 | 693.91 | 663.17 |
| VWF | von Willebrand factor | -1.1039 | 0.10811 | 338.26 | 346.75 | 327.28 | 294.11 |
| AKR1A1 | aldo-keto reductase family 1. member A1 (aldehyde reductase) | -1.1067 | 0.05879 | 3452.1 | 3669.1 | 3295.1 | 3138.2 |
| ATP5D | ATP synthase. H+ transporting. mitochondrial F1 complex. delta subunit | -1.1087 | 0.01531 | 5797.2 | 5694.1 | 5364.9 | 5006 |
| ARHGEF2 | rho/rac guanine nucleotide exchange factor (GEF) 2 | -1.1093 | 0.02685 | 2027 | 1849.8 | 1697.1 | 1795.7 |
| SRXN1 | sulfiredoxin 1 homolog (S. cerevisiae) | -1.1098 | 0.11662 | 971.17 | 963.36 | 899.69 | 844.35 |
| D15Wsu75e | DNA segment. Chr 15. Wayne State University 75. expressed | -1.1114 | 0.08729 | 1039.6 | 968.24 | 926.73 | 879.38 |
| NOL5A | nucleolar protein 5A (56kDa with KKE/D repeat) | -1.1118 | 0.22894 | 2978.6 | 3339.3 | 2594.8 | 3101.2 |
| ALS2CR13 | amyotrophic lateral sclerosis 2 (juvenile) chromosome region. candidate 13 | -1.1139 | 0.1134 | 1596.6 | 1604.6 | 1542.3 | 1338.7 |
| SERPINB6 | serpin peptidase inhibitor. clade B (ovalbumin). member 6 | -1.1162 | 0.02974 | 7033.3 | 7742.2 | 6366.7 | 6864.2 |
| PDCD2L | programmed cell death 2-like | -1.1199 | 0.07067 | 729.98 | 735.11 | 690.84 | 619.33 |
| TNFRSF14 | tumor necrosis factor receptor superfamily. member 14 (herpesvirus entry mediator) | -1.1217 | 0.10326 | 909.77 | 892.75 | 826.32 | 781.19 |
| CA12 | carbonic anhydrase XII | -1.1224 | 0.03626 | 423.1 | 376.49 | 372.49 | 339.46 |
| SLC7A5 | solute carrier family 7 (cationic amino acid transporter. y+ system). member 5 | -1.1233 | 0.01867 | 789.66 | 811.2 | 765.33 | 663.38 |
| CTXN1 | cortexin 1 | -1.1259 | 0.06528 | 2133.4 | 2051.6 | 1879 | 1837.4 |
| CDCA7 | cell division cycle associated 7 | -1.1268 | 0.07307 | 331.33 | 337.64 | 291.02 | 302.74 |
| GEMIN4 | gem (nuclear organelle) associated protein 4 | -1.127 | 0.02328 | 960.37 | 999.32 | 882.06 | 856.64 |
| PUS7 | pseudouridylate synthase 7 homolog (S. cerevisiae) | -1.1274 | 0.04684 | 831.09 | 748.2 | 673.25 | 726.72 |
| ALCAM | activated leukocyte cell adhesion molecule | -1.1275 | 0.0522 | 1460.8 | 1609.4 | 1287.7 | 1436.1 |
| SARS2 | seryl-tRNA synthetase 2. mitochondrial | -1.1283 | 0.02363 | 1161.2 | 1281.8 | 1058.9 | 1104 |
| RAB26 | RAB26. member RAS oncogene family | -1.1296 | 0.06866 | 1138 | 1066.2 | 962.53 | 988.01 |
| GCLM | glutamate-cysteine ligase. modifier subunit | -1.1308 | 0.14517 | 1128.3 | 939.83 | 928.63 | 893.1 |
| TUBB3 | tubulin. beta 3 | -1.1319 | 0.12309 | 358.24 | 318.58 | 294.11 | 302.88 |
| ATAD4 | ATPase family. AAA domain containing 4 | -1.1341 | 0.03272 | 1116.3 | 1173.2 | 949.38 | 1072.5 |
| CCDC58 | coiled-coil domain containing 58 | -1.1341 | 0.2017 | 823.67 | 912.44 | 718.42 | 813.41 |
| ID2 | inhibitor of DNA binding 2. dominant negative helix-loop-helix protein | -1.1347 | 0.02362 | 430.44 | 382.78 | 366.66 | 349.04 |
| ANK3 | ankyrin 3. node of Ranvier (ankyrin G) | -1.1381 | 0.00895 | 2823.2 | 2640.3 | 2443.7 | 2354.9 |
| COQ2 | coenzyme Q2 homolog. prenyltransferase (yeast) | -1.1404 | 0.00941 | 1307.4 | 1295.8 | 1125.9 | 1157 |
| MRPL12 | mitochondrial ribosomal protein L12 | -1.1408 | 0.03948 | 735.66 | 771.2 | 680.76 | 640.32 |
| OPTN | optineurin | -1.1408 | 0.00413 | 202.13 | 185.13 | 166.63 | 172.56 |
| SAMD13 | sterile alpha motif domain containing 13 | -1.1411 | 0.00255 | 308.08 | 298.58 | 261.27 | 270.38 |
| SH3BP4 | SH3-domain binding protein 4 | -1.143 | 0.00747 | 1216.1 | 1235.7 | 1113.1 | 1033.3 |
| FKBP4 | FK506 binding protein 4. 59kDa | -1.1451 | 0.13053 | 1561.8 | 1972 | 1496.1 | 1569.9 |
| CAPN13 | calpain 13 | -1.1468 | 0.03453 | 582.73 | 500.24 | 450.83 | 491.64 |
| FAM60A | family with sequence similarity 60. member A | -1.1477 | 0.02733 | 2066.7 | 1870.4 | 1703.7 | 1722.6 |
| LYSMD2 | LysM. putative peptidoglycan-binding. domain containing 2 | -1.1498 | 0.00272 | 769.59 | 714.05 | 643.92 | 645.5 |
| POLD2 | polymerase (DNA directed). delta 2. regulatory subunit 50kDa | -1.1503 | 0.0083 | 11579 | 11202 | 9881.1 | 9920.9 |
| MGC23985 | similar to AVLV472 | -1.1524 | 0.0009 | 184.58 | 178.52 | 158.78 | 156.27 |
| ZMIZ1 | zinc finger. MIZ-type containing 1 | -1.1542 | 0.07252 | 1220.4 | 1059.8 | 1040.3 | 933.28 |
| MAFB | v-maf musculoaponeurotic fibrosarcoma oncogene homolog B (avian) | -1.1559 | 0.00294 | 1849.8 | 1705.6 | 1566.4 | 1507.5 |
| PSME2 | proteasome (prosome. macropain) activator subunit 2 (PA28 beta) | -1.1564 | 0.00992 | 2010.4 | 1962.8 | 1828.6 | 1613.7 |
| GBL | G protein beta subunit-like | -1.158 | 0.01216 | 1505.1 | 1496.1 | 1305.5 | 1286.3 |
| PRKD1 | protein kinase D1 | -1.1591 | 0.00792 | 2675.3 | 2446.3 | 2166.8 | 2248.1 |
| C18orf56 | chromosome 18 open reading frame 56 | -1.1593 | 0.10281 | 1242.7 | 1023.4 | 978.88 | 966.77 |
| PAICS | phosphoribosylaminoimidazole carboxylase. phosphoribosylaminoimidazole succinocarboxamide synthetase | -1.1616 | 0.00686 | 4964.2 | 5140.7 | 4444.7 | 4254.8 |
| MARCKSL1 | MARCKS-like 1 | -1.1649 | 0.02829 | 3256.5 | 3808.8 | 2993.5 | 3053.3 |
| FOXA1 | forkhead box A1 | -1.1714 | 0.05319 | 10206 | 10066 | 8677 | 8627.5 |
| UGDH | UDP-glucose dehydrogenase | -1.1716 | 0.02544 | 979.25 | 949.38 | 758.71 | 892.75 |
| RETSAT | retinol saturase (all-trans-retinol 13.14-reductase) | -1.1722 | 0.07008 | 473.71 | 441.64 | 421.33 | 361.35 |
| AR | androgen receptor (dihydrotestosterone receptor; testicular feminization; spinal and bulbar muscular atrophy; Kennedy disease) | -1.1732 | 0.00645 | 647.68 | 715.79 | 617.84 | 545.17 |
| SEPP1 | selenoprotein P. plasma. 1 | -1.1738 | 0.05524 | 554.12 | 638.27 | 483.19 | 531.27 |
| NFIX | nuclear factor I/X (CCAAT-binding transcription factor) | -1.1744 | 0.02978 | 3480.9 | 3736.7 | 2950.6 | 3196.3 |
| GATA2 | GATA binding protein 2 | -1.175 | 0.01206 | 620.39 | 666.15 | 590.34 | 507.06 |
| RBPJ | recombination signal binding protein for immunoglobulin kappa J region | -1.1752 | 0.00405 | 397.02 | 343.66 | 321.16 | 307.63 |
| MT1A | metallothionein 1A | -1.1777 | 0.05405 | 3615.8 | 2976.5 | 2814.5 | 2756.9 |
| SELENBP1 | selenium binding protein 1 | -1.178 | 0.07377 | 1562.6 | 1765.8 | 1399.7 | 1420.8 |
| PNPLA7 | patatin-like phospholipase domain containing 7 | -1.1785 | 0.004 | 1246.7 | 1157.7 | 1044 | 995.38 |
| BCL11B | B-cell CLL/lymphoma 11B (zinc finger protein) | -1.1795 | 0.00167 | 229.25 | 215.65 | 183.82 | 193.33 |
| LPIN1 | lipin 1 | -1.1796 | 0.06653 | 2753.7 | 1933.6 | 1919.9 | 1993.3 |
| MT2A | metallothionein 2A | -1.1852 | 0.01802 | 3947.5 | 3813.4 | 3256.5 | 3290.9 |
| KIAA1244 | KIAA1244 | -1.1874 | 0.18626 | 630.15 | 844.35 | 512.71 | 736.04 |
| GP1BB | glycoprotein Ib (platelet). beta polypeptide | -1.1874 | 0.00197 | 1130.8 | 1142.1 | 945.74 | 968.66 |
| NME1 | non-metastatic cells 1. protein (NM23A) expressed in | -1.189 | 0.00448 | 8769.2 | 8321.8 | 7257.6 | 7112.7 |
| WARS | tryptophanyl-tRNA synthetase | -1.1895 | 0.08112 | 1455.6 | 1329 | 1287 | 1062.3 |
| ATP6V0E2 | ATPase. H+ transporting V0 subunit e2 | -1.1899 | 0.00077 | 6739.5 | 6981.7 | 5911.7 | 5621.3 |
| MME | membrane metallo-endopeptidase | -1.192 | 0.00457 | 1698.2 | 1791.3 | 1504 | 1423.5 |
| F12 | coagulation factor XII (Hageman factor) | -1.1926 | 0.00088 | 2061.5 | 2048.3 | 1750.4 | 1696.2 |
| CAMK2B | calcium/calmodulin-dependent protein kinase (CaM kinase) II beta | -1.1961 | 0.00019 | 966.77 | 946.28 | 804.51 | 794.86 |
| IFRD2 | interferon-related developmental regulator 2 | -1.1971 | 0.00115 | 512.1 | 543.67 | 452.9 | 428.95 |
| UBE2H | ubiquitin-conjugating enzyme E2H (UBC8 homolog. yeast) | -1.1977 | 0.06487 | 739.71 | 860.45 | 606.21 | 731.92 |
| MPP6 | membrane protein. palmitoylated 6 (MAGUK p55 subfamily member 6) | -1.2017 | 9.81E-05 | 449.93 | 452.9 | 381.51 | 369.91 |
| KHDRBS3 | KH domain containing. RNA binding. signal transduction associated 3 | -1.2094 | 0.01429 | 645.26 | 769.1 | 569.64 | 595.64 |
| KLK3 | kallikrein-related peptidase 3 | -1.2167 | 0.02776 | 4254.8 | 4448.5 | 3719 | 3437.8 |
| GPT2 | glutamic pyruvate transaminase (alanine aminotransferase) 2 | -1.2208 | 0.00193 | 4938.2 | 4427.2 | 3942 | 3721.4 |
| PPP1R14B | protein phosphatase 1. regulatory (inhibitor) subunit 14B | -1.2214 | 0.00324 | 7958.2 | 8136.1 | 6693.3 | 6484.7 |
| AMHR2 | anti-Mullerian hormone receptor. type II | -1.2229 | 0.00063 | 319.84 | 314.69 | 261.95 | 256.92 |
| MFSD3 | major facilitator superfamily domain containing 3 | -1.2236 | 0.00169 | 4644.3 | 4544.1 | 4036.6 | 3492.3 |
| PAFAH1B3 | platelet-activating factor acetylhydrolase. isoform Ib. gamma subunit 29kDa | -1.2246 | 0.0005 | 514.54 | 532.37 | 436.21 | 418.77 |
| MYCBP2 | MYC binding protein 2 | -1.2251 | 0.0051 | 956.5 | 844.77 | 717.11 | 750.75 |
| LAMA3 | laminin. alpha 3 | -1.2271 | 0.01871 | 1384.7 | 1168.9 | 1087.5 | 988.48 |
| IMPA2 | inositol(myo)-1(or 4)-monophosphatase 2 | -1.2319 | 0.00196 | 487.39 | 542.85 | 413.34 | 421.81 |
| PARP10 | poly (ADP-ribose) polymerase family. member 10 | -1.2339 | 0.01621 | 559.6 | 601.85 | 445.32 | 496.72 |
| SC65 | synaptonemal complex protein SC65 | -1.2366 | 0.00122 | 1136.9 | 1176.5 | 934.19 | 936.38 |
| CAPN5 | calpain 5 | -1.2414 | 4.30E-05 | 514.14 | 517.52 | 412 | 419.07 |
| CMBL | carboxymethylenebutenolidase homolog (Pseudomonas) | -1.2416 | 0.00136 | 2820.4 | 2719.1 | 2381.4 | 2088.9 |
| KRT80 | keratin 80 | -1.2421 | 3.16E-05 | 214.58 | 204.35 | 173 | 164.29 |
| RANBP1 | RAN binding protein 1 | -1.2443 | 0.00024 | 1389.9 | 1344.4 | 1072.5 | 1125.4 |
| LOC153364 | similar to metallo-beta-lactamase superfamily protein | -1.2456 | 0.00046 | 346.19 | 364.48 | 286.02 | 284.35 |
| KLK4 | kallikrein-related peptidase 4 | -1.2457 | 0.00253 | 6995.1 | 6342.1 | 5543.5 | 5157.1 |
| SDCCAG3 | serologically defined colon cancer antigen 3 | -1.2473 | 0.00028 | 1312.9 | 1348.5 | 1059.8 | 1073.8 |
| C15orf52 | chromosome 15 open reading frame 52 | -1.2494 | 0.01351 | 715.45 | 575.69 | 488.01 | 540.65 |
| PAK4 | p21(CDKN1A)-activated kinase 4 | -1.2559 | 0.00055 | 1084.6 | 1271 | 925.68 | 944.25 |
| TACC1 | transforming. acidic coiled-coil containing protein 1 | -1.2572 | 7.57E-05 | 3897.7 | 3620.9 | 2965.5 | 3011 |
| STEAP1 | six transmembrane epithelial antigen of the prostate 1 | -1.2591 | 0.00073 | 7204.7 | 7257.6 | 6040.7 | 5460.5 |
| ST7 | suppression of tumorigenicity 7 | -1.2593 | 0.00344 | 1311.4 | 1622.2 | 1247.5 | 1075.2 |
| RPL29 | ribosomal protein L29 | -1.2688 | 0.00065 | 1106.2 | 1121.5 | 899.14 | 856.99 |
| PEX10 | peroxisome biogenesis factor 10 | -1.2735 | 0.00032 | 3360.6 | 3396.2 | 2618.8 | 2687.4 |
| PTPLB | protein tyrosine phosphatase-like (proline instead of catalytic arginine). member b | -1.2793 | 0.0062 | 1431.9 | 1477.3 | 1056.3 | 1223.7 |
| NUAK1 | NUAK family. SNF1-like kinase. 1 | -1.2805 | 2.76E-05 | 365.24 | 348.62 | 279.5 | 277.86 |
| C9orf58 | chromosome 9 open reading frame 58 | -1.2811 | 0.00074 | 2481.5 | 2798.5 | 2003.7 | 2111.9 |
| MT1F | metallothionein 1F | -1.2814 | 8.98E-05 | 737.17 | 716.88 | 551.46 | 583.64 |
| KIF22 | kinesin family member 22 | -1.2839 | 0.00024 | 369.82 | 424.28 | 316.01 | 301.22 |
| ADAMTS1 | ADAM metallopeptidase with thrombospondin type 1 motif. 1 | -1.2839 | 0.00263 | 1124.9 | 1136 | 803.68 | 964.55 |
| PSPH | phosphoserine phosphatase | -1.2884 | 0.00437 | 1229.8 | 1275.9 | 1021.2 | 925.68 |
| COASY | Coenzyme A synthase | -1.2887 | 0.00079 | 2989.2 | 2678.2 | 2287.9 | 2107.1 |
| EFCAB4A | EF-hand calcium binding domain 4A | -1.2915 | 0.00445 | 805.37 | 851.92 | 640.12 | 642.58 |
| IQGAP2 | IQ motif containing GTPase activating protein 2 | -1.2944 | 3.44E-05 | 280.45 | 273.47 | 222.28 | 205.93 |
| 9-Sep | septin 9 | -1.2991 | 0.00032 | 5836.3 | 5911.7 | 4775.6 | 4281.2 |
| GULP1 | GULP. engulfment adaptor PTB domain containing 1 | -1.3027 | 0.00227 | 742.77 | 702.93 | 591.17 | 520.4 |
| MAP2 | microtubule-associated protein 2 | -1.3124 | 1.19E-05 | 255.21 | 235.92 | 184.73 | 189.22 |
| IFI6 | interferon. alpha-inducible protein 6 | -1.3149 | 0.0005 | 2262.1 | 2050.1 | 1659.3 | 1616.4 |
| BEST1 | bestrophin 1 | -1.3166 | 0.00224 | 744.33 | 691.44 | 541.73 | 548.04 |
| AMACR | alpha-methylacyl-CoA racemase | -1.3197 | 0.00014 | 422.52 | 451.34 | 337.55 | 324.37 |
| SASH1 | SAM and SH3 domain containing 1 | -1.3274 | 0.00018 | 833.99 | 808.21 | 605.73 | 631.5 |
| C8orf55 | chromosome 8 open reading frame 55 | -1.3315 | 4.25E-05 | 6366.7 | 6553.1 | 5067.3 | 4644.3 |
| RFX5 | regulatory factor X. 5 (influences HLA class II expression) | -1.3319 | 0.00021 | 851.05 | 853.41 | 678.85 | 603.09 |
| SPIRE1 | spire homolog 1 (Drosophila) | -1.3335 | 0.00202 | 1703.7 | 1534.5 | 1204.7 | 1220.4 |
| SLC27A5 | solute carrier family 27 (fatty acid transporter). member 5 | -1.3393 | 4.91E-05 | 651.47 | 671.89 | 522.95 | 466.61 |
| TMSL3 | thymosin-like 3 | -1.3426 | 1.72E-05 | 1753.8 | 1706.8 | 1297.8 | 1279.6 |
| RAMP1 | receptor (G protein-coupled) activity modifying protein 1 | -1.3455 | 9.75E-05 | 1072 | 1001.8 | 823.67 | 720.22 |
| AK2 | adenylate kinase 2 | -1.3509 | 0.00653 | 4784.2 | 5810.2 | 3574.3 | 4261.6 |
| ZNF30 | zinc finger protein 30 | -1.3625 | 1.93E-05 | 468.19 | 454.24 | 332.42 | 344.63 |
| TMPRSS2 | transmembrane protease. serine 2 | -1.3644 | 6.30E-07 | 3853.5 | 4166.1 | 2945.9 | 2927.3 |
| RTN1 | reticulon 1 | -1.3657 | 5.80E-06 | 455.08 | 456.24 | 338.53 | 328.82 |
| CYP11A1 | cytochrome P450. family 11. subfamily A. polypeptide 1 | -1.3688 | 9.63E-05 | 230.65 | 197.48 | 155.75 | 156.1 |
| PPAP2A | phosphatidic acid phosphatase type 2A | -1.3696 | 7.79E-07 | 273.29 | 290.12 | 207.2 | 203.98 |
| SQSTM1 | sequestosome 1 | -1.3696 | 2.07E-05 | 3319.6 | 3002.4 | 2264.2 | 2346.7 |
| ALDH1A3 | aldehyde dehydrogenase 1 family. member A3 | -1.3712 | 0.00019 | 599.55 | 590.85 | 437.28 | 430.89 |
| C1orf93 | chromosome 1 open reading frame 93 | -1.3752 | 9.10E-07 | 686.7 | 713.76 | 523.72 | 494.85 |
| STMN3 | stathmin-like 3 | -1.3767 | 3.32E-07 | 389.01 | 407.52 | 288.48 | 289.93 |
| OVGP1 | oviductal glycoprotein 1. 120kDa (mucin 9. oviductin) | -1.3779 | 7.17E-07 | 462.95 | 443.23 | 325.1 | 332.42 |
| KIAA0746 | KIAA0746 protein | -1.3783 | 7.63E-05 | 3116 | 3018.3 | 2295.8 | 2156.6 |
| CASP4 | caspase 4. apoptosis-related cysteine peptidase | -1.3792 | 6.62E-07 | 1063.3 | 1092.9 | 753.77 | 810.42 |
| BAMBI | BMP and activin membrane-bound inhibitor homolog (Xenopus laevis) | -1.3863 | 1.29E-07 | 1410.9 | 1392.3 | 1032.5 | 989.92 |
| TAX1BP3 | Tax1 (human T-cell leukemia virus type I) binding protein 3 | -1.3914 | 0.00036 | 1306.6 | 1405.8 | 925.08 | 1025.7 |
| ABHD7 | abhydrolase domain containing 7 | -1.3992 | 9.32E-06 | 273.72 | 274.35 | 194.79 | 196.92 |
| IDH1 | isocitrate dehydrogenase 1 (NADP+). soluble | -1.4021 | 6.71E-06 | 1915.5 | 1979.5 | 1400.9 | 1376.7 |
| ABLIM1 | actin binding LIM protein 1 | -1.406 | 0.00017 | 1052.4 | 1076.9 | 787.94 | 727.65 |
| SLC25A10 | solute carrier family 25 (mitochondrial carrier; dicarboxylate transporter). member 10 | -1.4066 | 0.00279 | 1436.1 | 1474.1 | 944.25 | 1133.2 |
| H2AFJ | H2A histone family. member J | -1.4094 | 0.00026 | 3200.3 | 3364.7 | 2438.6 | 2222.8 |
| NQO1 | NAD(P)H dehydrogenase. quinone 1 | -1.417 | 1.52E-05 | 2802.6 | 2747.7 | 1906 | 2012.1 |
| LOC493869 | similar to RIKEN cDNA 2310016C16 | -1.4205 | 0.00058 | 330.98 | 334.41 | 210.06 | 261.13 |
| SPECC1L | SPECC1-like | -1.4236 | 0.00014 | 4897.6 | 5077.7 | 3559.1 | 3447.6 |
| ATP6V1E2 | ATPase. H+ transporting. lysosomal 31kDa. V1 subunit E2 | -1.4241 | 1.12E-07 | 650.94 | 641.98 | 452.67 | 455.17 |
| AADAT | aminoadipate aminotransferase | -1.4261 | 0.00109 | 977.39 | 849.65 | 649.74 | 628.46 |
| VIM | vimentin | -1.4277 | 1.21E-05 | 314.22 | 318.73 | 215.96 | 227.53 |
| PRPS1 | phosphoribosyl pyrophosphate synthetase 1 | -1.4289 | 3.26E-05 | 2002.8 | 2038.2 | 1311.4 | 1524.6 |
| TXNRD1 | thioredoxin reductase 1 | -1.4336 | 0.00019 | 2436.9 | 2458.3 | 1707.9 | 1706.8 |
| TMEM2 | transmembrane protein 2 | -1.4367 | 9.86E-05 | 776.77 | 675.48 | 545.31 | 466.14 |
| HSPB8 | heat shock 22kDa protein 8 | -1.4392 | 0.00143 | 248.73 | 262.83 | 169.76 | 185.93 |
| AMD1 | adenosylmethionine decarboxylase 1 | -1.4396 | 0.00026 | 2186.8 | 2039.1 | 1549.3 | 1388.9 |
| CAB39L | calcium binding protein 39-like | -1.4471 | 3.10E-05 | 1359.7 | 1548.1 | 947.64 | 1060.7 |
| BANK1 | B-cell scaffold protein with ankyrin repeats 1 | -1.4577 | 3.80E-06 | 719.83 | 736.72 | 478.8 | 521.26 |
| PANX2 | pannexin 2 | -1.4578 | 9.63E-06 | 572.4 | 581.06 | 371.14 | 421.69 |
| PPIC | peptidylprolyl isomerase C (cyclophilin C) | -1.4616 | 6.88E-05 | 772.54 | 702.27 | 506 | 501.92 |
| UGT2B10 | UDP glucuronosyltransferase 2 family. polypeptide B10 | -1.4629 | 1.76E-06 | 201.26 | 220.93 | 145.83 | 142.47 |
| TMEFF2 | transmembrane protein with EGF-like and two follistatin-like domains 2 | -1.4635 | 1.11E-06 | 1252.7 | 1341.4 | 863.61 | 908.44 |
| ST6GALNAC1 | ST6 (alpha-N-acetyl-neuraminyl-2.3-beta-galactosyl-1.3)-N-acetylgalactosaminide alpha-2.6-sialyltransferase 1 | -1.4656 | 9.29E-08 | 398.24 | 388.09 | 267.85 | 268.65 |
| COL4A5 | collagen. type IV. alpha 5 (Alport syndrome) | -1.4667 | 4.91E-05 | 1527.9 | 1316.5 | 1017 | 919.42 |
| COL16A1 | collagen. type XVI. alpha 1 | -1.4684 | 4.57E-05 | 469.07 | 412.13 | 289.53 | 309.67 |
| KCNN2 | potassium intermediate/small conductance calcium-activated channel. subfamily N. member 2 | -1.4775 | 3.66E-06 | 1669.4 | 1583 | 1170.6 | 1034.2 |
| ZDHHC14 | zinc finger. DHHC-type containing 14 | -1.479 | 1.81E-06 | 948.44 | 1013.5 | 645.5 | 680.76 |
| GLRX | glutaredoxin (thioltransferase) | -1.4848 | 3.68E-06 | 353.47 | 329.33 | 245.81 | 214.81 |
| TMEM158 | transmembrane protein 158 | -1.4881 | 3.40E-09 | 385.17 | 406.11 | 268.96 | 262.63 |
| MESP1 | mesoderm posterior 1 homolog (mouse) | -1.4896 | 2.43E-06 | 1676.3 | 1764.6 | 1178.4 | 1131.3 |
| HOXC6 | homeobox C6 | -1.5018 | 6.21E-06 | 2095.1 | 1840.2 | 1317.1 | 1297.8 |
| H2BFS | H2B histone family. member S | -1.5029 | 1.84E-05 | 1415.2 | 1288.3 | 927.7 | 870.15 |
| LPXN | leupaxin | -1.5061 | 6.37E-07 | 525.86 | 464.06 | 326.69 | 329.33 |
| RP11-298P3.3 | CG016 | -1.5082 | 2.58E-05 | 1235.7 | 1015.6 | 718.99 | 767.34 |
| MAF | v-maf musculoaponeurotic fibrosarcoma oncogene homolog (avian) | -1.5119 | 3.04E-08 | 253.82 | 245.95 | 168.76 | 161.82 |
| CYP39A1 | cytochrome P450. family 39. subfamily A. polypeptide 1 | -1.5144 | 2.31E-07 | 243.82 | 235.48 | 158.08 | 158.37 |
| PPFIBP2 | PTPRF interacting protein. binding protein 2 (liprin beta 2) | -1.5155 | 9.96E-07 | 1256 | 1217.5 | 817.77 | 814.16 |
| TP53INP1 | tumor protein p53 inducible nuclear protein 1 | -1.5166 | 7.53E-07 | 1610.4 | 1523.5 | 1002.7 | 1063.8 |
| ENC1 | ectodermal-neural cortex (with BTB-like domain) | -1.5242 | 1.91E-06 | 544.26 | 491.33 | 338.81 | 339.75 |
| MRPL27 | mitochondrial ribosomal protein L27 | -1.5247 | 0.0001 | 1478.9 | 1572.2 | 1090.2 | 917.43 |
| HIST2H2BE | histone cluster 2. H2be | -1.5309 | 6.45E-08 | 1681.2 | 1644.9 | 1103.4 | 1069.4 |
| PHOSPHO2 | phosphatase. orphan 2 | -1.538 | 3.61E-09 | 542.85 | 514.78 | 339.95 | 347.53 |
| SPATA20 | spermatogenesis associated 20 | -1.5587 | 1.94E-05 | 2955.8 | 3084.3 | 2199.9 | 1705.6 |
| RPS15 | ribosomal protein S15 | -1.5661 | 0.00041 | 822.45 | 991.86 | 572.61 | 580.85 |
| CLDN8 | claudin 8 | -1.571 | 8.09E-08 | 412.49 | 423.8 | 263.95 | 268.35 |
| P2RY11 | purinergic receptor P2Y. G-protein coupled. 11 | -1.5763 | 6.10E-07 | 428.06 | 428.95 | 280.68 | 263.28 |
| TP53AP1 | TP53 activated protein 1 | -1.585 | 5.24E-06 | 632.9 | 622.41 | 427.67 | 366.66 |
| HIST1H2BD | histone cluster 1. H2bd | -1.6164 | 7.96E-08 | 516.18 | 468.93 | 309.23 | 299.61 |
| C1orf144 | chromosome 1 open reading frame 144 | -1.6173 | 4.85E-05 | 1096.2 | 1113.1 | 639.54 | 729.37 |
| DNASE2B | deoxyribonuclease II beta | -1.6366 | 3.13E-08 | 1189.7 | 1205.5 | 738.4 | 725.15 |
| ID1 | inhibitor of DNA binding 1. dominant negative helix-loop-helix protein | -1.6527 | 6.30E-09 | 237.59 | 229.91 | 142.26 | 140.57 |
| MB | myoglobin | -1.6646 | 9.50E-08 | 550.55 | 566.53 | 343.08 | 328.09 |
| SLC39A8 | solute carrier family 39 (zinc transporter). member 8 | -1.6677 | 4.25E-07 | 987.38 | 1067.9 | 606.32 | 625.33 |
| SERPINE2 | serpin peptidase inhibitor. clade E (nexin. plasminogen activator inhibitor type 1). member 2 | -1.6712 | 7.66E-06 | 511.53 | 400.04 | 268.73 | 272.66 |
| ZC3HAV1 | zinc finger CCCH-type. antiviral 1 | -1.6728 | 1.05E-07 | 2164.9 | 2183.3 | 1244.6 | 1357.1 |
| LCN2 | lipocalin 2 (oncogene 24p3) | -1.6811 | 2.34E-06 | 359.2 | 366.97 | 218.17 | 213.79 |
| TMEPAI | transmembrane. prostate androgen induced RNA | -1.6867 | 7.97E-08 | 1323.8 | 1382.5 | 796.15 | 807.96 |
| PKIB | protein kinase (cAMP-dependent. catalytic) inhibitor beta | -1.6933 | 9.40E-06 | 451.09 | 488.68 | 300.71 | 255.67 |
| ALDH3A2 | aldehyde dehydrogenase 3 family. member A2 | -1.7041 | 3.40E-09 | 3913.2 | 3936 | 2293.1 | 2312.9 |
| TMEM45B | transmembrane protein 45B | -1.7076 | 9.47E-07 | 601.85 | 602.57 | 374.49 | 332.09 |
| ANKRD25 | ankyrin repeat domain 25 | -1.7149 | 1.85E-09 | 412 | 408.93 | 239.7 | 239 |
| SH3BGRL | SH3 domain binding glutamic acid-rich protein like | -1.7169 | 1.42E-06 | 713.22 | 653.08 | 393.2 | 401.85 |
| NKX3-1 | NK3 homeobox 1 | -1.7367 | 0.00178 | 3290.9 | 4005.9 | 1768.1 | 2472 |
| GBP2 | guanylate binding protein 2. interferon-inducible | -1.7419 | 1.75E-06 | 470.67 | 399.49 | 256.33 | 241.76 |
| IFI35 | interferon-induced protein 35 | -1.7518 | 3.98E-07 | 714.05 | 773.02 | 427.24 | 420.99 |
| ISG15 | ISG15 ubiquitin-like modifier | -1.763 | 5.16E-07 | 2328.1 | 2351.9 | 1417.5 | 1242.7 |
| CDT1 | chromatin licensing and DNA replication factor 1 | -1.7753 | 1.06E-09 | 617.29 | 590.05 | 345.31 | 334.68 |
| GUCY1A3 | guanylate cyclase 1. soluble. alpha 3 | -1.7917 | 1.43E-09 | 1737.2 | 1691.8 | 963.36 | 950.36 |
| UGT2B28 | UDP glucuronosyltransferase 2 family. polypeptide B28 | -1.7975 | 4.46E-09 | 422.4 | 433.09 | 226.42 | 250.06 |
| HIST2H2AC | histone cluster 2. H2ac | -1.8 | 5.14E-07 | 2116.8 | 2111.9 | 1168.9 | 1180.4 |
| HIST1H3H | histone cluster 1. H3h | -1.8127 | 1.79E-07 | 475.26 | 402.03 | 250.02 | 232.58 |
| AK1 | adenylate kinase 1 | -1.8241 | 3.20E-07 | 880.16 | 865.72 | 447.2 | 512.1 |
| GPNMB | glycoprotein (transmembrane) nmb | -1.8522 | 6.86E-10 | 391.52 | 389.77 | 214.58 | 207.3 |
| ID3 | inhibitor of DNA binding 3. dominant negative helix-loop-helix protein | -1.8553 | 5.92E-08 | 303.7 | 271.7 | 153.17 | 156.5 |
| PPFIA2 | protein tyrosine phosphatase. receptor type. f polypeptide (PTPRF). interacting protein (liprin). alpha 2 | -1.8782 | 1.48E-08 | 682 | 729.37 | 380.18 | 370.89 |
| HIST1H2AC | histone cluster 1. H2ac | -1.88 | 1.03E-07 | 662.56 | 589.35 | 318.62 | 346.75 |
| C1orf116 | chromosome 1 open reading frame 116 | -1.9258 | 1.21E-08 | 3503.8 | 3387.6 | 1945.7 | 1644.9 |
| HIST2H2AA3 | histone cluster 2. H2aa3 | -1.9556 | 1.11E-07 | 1294.1 | 1390.7 | 676.09 | 695.98 |
| NOS3 | nitric oxide synthase 3 (endothelial cell) | -1.9723 | 7.11E-07 | 505.47 | 476.04 | 249.18 | 248.23 |
| HIST1H4H | histone cluster 1. H4h | -1.981 | 4.50E-08 | 1279.2 | 1181.4 | 601.85 | 639.83 |
| HIST1H1C | histone cluster 1. H1c | -2.0184 | 7.99E-08 | 4094.5 | 3785.8 | 1874.8 | 2029.5 |
| TRPV6 | transient receptor potential cation channel. subfamily V. member 6 | -2.0805 | 6.53E-10 | 1039.9 | 961.17 | 477.05 | 484.08 |
| VCX3B | variable charge. X-linked 3B | -2.4255 | 2.89E-09 | 667.64 | 668.77 | 270.95 | 280.11 |
| VCY | variable charge. Y-linked | -2.5139 | 3.58E-09 | 645.96 | 694.23 | 264.05 | 268.73 |
| VCX2 | variable charge. X-linked 2 | -2.7981 | 8.12E-11 | 774.84 | 767.34 | 287.24 | 264.38 |
| UGT2B11 | UDP glucuronosyltransferase 2 family. polypeptide B11 | -2.8232 | 1.72E-06 | 825.72 | 993.08 | 293.96 | 350 |
| UGT2B17 | UDP glucuronosyltransferase 2 family. polypeptide B17 | -3.0224 | 3.31E-11 | 1211.6 | 1364.9 | 424.28 | 426.66 |
| VCX | variable charge. X-linked | -3.0585 | 3.97E-09 | 2647.7 | 2578.2 | 910.58 | 801.38 |
| UGT2B7 | UDP glucuronosyltransferase 2 family. polypeptide B7 | -3.3367 | 4.25E-12 | 3574.3 | 3583.3 | 1137.6 | 1011.2 |
| TXNIP | thioredoxin interacting protein | -7.2749 | 9.39E-12 | 10390 | 10123 | 1344.4 | 1478.2 |
| **Symbol** | **Description** | **FoldChange** | **P.value** | **Control - 1** | **Control - 2** | **CpdA. 10uM. 8h - 1** | **CpdA. 10uM. 8h - 2** |
| TIPARP | TCDD-inducible poly(ADP-ribose) polymerase | -1.926 | 1.35E-07 | 498.47 | 453.08 | 244.72 | 248.8 |
| PNLIP | pancreatic lipase | -4.7123 | 1.30E-11 | 638.07 | 518.49 | 124.25 | 119.9 |
| ING1 | inhibitor of growth family. member 1 | -1.9115 | 4.87E-06 | 632.58 | 497.54 | 296.38 | 290.63 |
| RASD1 | RAS. dexamethasone-induced 1 | 1.5314 | 0.000111 | 569.08 | 475.26 | 828.74 | 765.33 |
| CHST3 | carbohydrate (chondroitin 6) sulfotransferase 3 | -1.1729 | 0.005292 | 168.33 | 171.11 | 145.57 | 143.81 |
| COL6A1 | collagen. type VI. alpha 1 | -1.173 | 0.017132 | 804.95 | 758.26 | 655.2 | 677.01 |
| TRIM48 | tripartite motif-containing 48 | -2.9196 | 2.19E-10 | 432.97 | 374.05 | 137.95 | 137.73 |
| SPRYD5 | SPRY domain containing 5 | -8.6497 | 1.22E-13 | 2100.1 | 1654.6 | 222.01 | 209.2 |
| PGC | progastricsin (pepsinogen C) | -2.7445 | 9.54E-10 | 530.87 | 456.57 | 188.25 | 170.94 |
| SRD5A1 | steroid-5-alpha-reductase. alpha polypeptide 1 (3-oxo-5 alpha-steroid delta 4-dehydrogenase alpha 1) | -1.3865 | 8.82E-06 | 381.3 | 361.6 | 272.81 | 262.89 |
| TAF5L | TAF5-like RNA polymerase II. p300/CBP-associated factor (PCAF)-associated factor. 65kDa | -1.0064 | 0.86205 | 309.43 | 331.77 | 317.82 | 318.94 |
| TUBA3E | tubulin. alpha 3e | -3.9121 | 1.30E-12 | 585.22 | 632.4 | 157.3 | 153.74 |
| PTGER4 | prostaglandin E receptor 4 (subtype EP4) | -1.3769 | 0.000154 | 275.13 | 254 | 183.6 | 200.78 |
| FKBP5 | FK506 binding protein 5 | -3.0163 | 1.76E-09 | 1547.2 | 1332 | 498.47 | 454.44 |
| CTGF | connective tissue growth factor | -1.1223 | 0.089982 | 159.44 | 145.72 | 137.33 | 134.31 |
| AZGP1 | alpha-2-glycoprotein 1. zinc-binding | 1.01 | 0.91063 | 158.21 | 153.5 | 150.1 | 165.04 |
| PQLC1 | PQ loop repeat containing 1 | -1.0551 | 0.18391 | 1773.4 | 1818.2 | 1701.2 | 1702.5 |
| SGK | serum/glucocorticoid regulated kinase | -21.24 | 3.38E-13 | 4135.9 | 3104.1 | 180.39 | 157.75 |
| OGFRL1 | opioid growth factor receptor-like 1 | -1.1447 | 0.013413 | 204.87 | 206.65 | 184.87 | 174.75 |
| FLJ20160 | FLJ20160 protein | -1.5811 | 3.47E-06 | 984.74 | 913.79 | 618.13 | 582.33 |
| GNMT | glycine N-methyltransferase | 1.074 | 0.21493 | 249.67 | 244.22 | 267.43 | 263 |
| NSDHL | NAD(P) dependent steroid dehydrogenase-like | -1.1089 | 0.064013 | 957.91 | 850.42 | 836.26 | 792.24 |
| STOM | stomatin | 1.0333 | 0.45399 | 134.3 | 126.83 | 134.13 | 135.57 |
| FAM104A | family with sequence similarity 104. member A | -1.3292 | 0.025482 | 694.23 | 726.45 | 488.92 | 583.81 |
| SLC39A14 | solute carrier family 39 (zinc transporter). member 14 | 1.0562 | 0.24935 | 547.61 | 517.79 | 577.4 | 547.83 |
| CRISPLD2 | cysteine-rich secretory protein LCCL domain containing 2 | -1.1031 | 0.14923 | 245.81 | 234.22 | 221.66 | 213.46 |
| TUBA3C | tubulin. alpha 3c | -1.8751 | 1.30E-06 | 222.47 | 241.86 | 124.14 | 123.28 |
| LONRF1 | LON peptidase N-terminal domain and ring finger 1 | -1.0168 | 0.85261 | 486.36 | 404.5 | 445.32 | 427.33 |
| ACSL1 | acyl-CoA synthetase long-chain family member 1 | -1.2121 | 0.4379 | 1452.1 | 1976.8 | 1188.6 | 1643.9 |
| PRIC285 | peroxisomal proliferator-activated receptor A interacting complex 285 | -1.1243 | 0.080254 | 624.68 | 615.97 | 569.08 | 534.94 |
| TUBA3D | tubulin. alpha 3d | -1.9882 | 5.51E-10 | 476.92 | 438.61 | 237.48 | 222.83 |
| RHOB | ras homolog gene family. member B | -1.8113 | 0.013951 | 671.68 | 872.92 | 364.61 | 490.17 |
| ZNF26 | zinc finger protein 26 | 1.0028 | 0.96886 | 252.98 | 228.8 | 241.69 | 240.84 |
| HPCAL1 | hippocalcin-like 1 | -1.4362 | 7.03E-07 | 1775.1 | 1629.5 | 1190.5 | 1177.9 |
| ZNF18 | zinc finger protein 18 | -1.0676 | 0.13881 | 408.56 | 369.23 | 358.41 | 369.31 |
| ZNF189 | zinc finger protein 189 | -1.3578 | 0.004944 | 695.77 | 552.84 | 471.53 | 442.46 |
| S100P | S100 calcium binding protein P | -1.7681 | 1.52E-05 | 9765.9 | 7606.5 | 5057.9 | 4697.9 |
| ATAD2 | ATPase family. AAA domain containing 2 | -1.7114 | 5.67E-05 | 555.3 | 461.54 | 296.09 | 295.56 |
| NET1 | neuroepithelial cell transforming gene 1 | -1.0174 | 0.67545 | 607.17 | 645.26 | 613.98 | 616.48 |
| FOXO1 | forkhead box O1 | -1.3355 | 0.000309 | 467.99 | 437.6 | 345.76 | 332.09 |
| COL6A2 | collagen. type VI. alpha 2 | -1.077 | 0.12663 | 183.92 | 178.43 | 168.2 | 168.21 |
| ST3GAL4 | ST3 beta-galactoside alpha-2.3-sialyltransferase 4 | -1.4636 | 6.56E-05 | 457.86 | 425.57 | 308.08 | 295.25 |
| CDC20 | cell division cycle 20 homolog (S. cerevisiae) | 1.9436 | 0.000975 | 1409.5 | 989.92 | 2432.4 | 2166.8 |
| TSPYL2 | TSPY-like 2 | 1.1776 | 0.009182 | 309.04 | 285.49 | 343.48 | 356.17 |
| ATP1A1 | ATPase. Na+/K+ transporting. alpha 1 polypeptide | 1.0585 | 0.28721 | 1324.8 | 1240.3 | 1325.6 | 1388.9 |
| RAB20 | RAB20. member RAS oncogene family | -2.8536 | 1.24E-10 | 2134.7 | 1935.5 | 708.48 | 716.17 |
| HK2 | hexokinase 2 | 1.6343 | 2.13E-07 | 256.07 | 243.82 | 382.07 | 436.45 |
| TMEM56 | transmembrane protein 56 | -1.3117 | 0.000326 | 308.17 | 283.77 | 236.39 | 215.02 |
| CDC42EP4 | CDC42 effector protein (Rho GTPase binding) 4 | -1.4286 | 1.43E-05 | 1534.5 | 1483.5 | 1039.6 | 1073 |
| CNKSR3 | CNKSR family member 3 | 1.4471 | 0.00072 | 395.62 | 341.05 | 553.4 | 510.53 |
| ELK1 | ELK1. member of ETS oncogene family | 1.0704 | 0.087763 | 672.48 | 751.08 | 737.17 | 785.09 |
| PLOD2 | procollagen-lysine. 2-oxoglutarate 5-dioxygenase 2 | 1.5877 | 0.002555 | 263.65 | 160.13 | 305.03 | 348.91 |
| OSGIN1 | oxidative stress induced growth inhibitor 1 | 1.3725 | 9.83E-06 | 598.77 | 577.4 | 821.46 | 792.79 |
| UBE2C | ubiquitin-conjugating enzyme E2C | 1.8956 | 3.64E-06 | 367.19 | 329.66 | 665.74 | 653.36 |
| SLC25A18 | solute carrier family 25 (mitochondrial carrier). member 18 | -1.0545 | 0.1446 | 142.22 | 134.11 | 131.56 | 130.37 |
| WIPI1 | WD repeat domain. phosphoinositide interacting 1 | 1.0621 | 0.19745 | 260.46 | 239.04 | 275.85 | 254.63 |
| HMGB2 | high-mobility group box 2 | 1.7962 | 4.70E-05 | 313.23 | 261.02 | 527.15 | 500.38 |
| GHR | growth hormone receptor | -2.0627 | 2.36E-06 | 725.51 | 796.88 | 361.85 | 375.54 |
| FMO5 | flavin containing monooxygenase 5 | -1.1662 | 0.000756 | 156.56 | 159.16 | 131.13 | 139.73 |
| IQGAP3 | IQ motif containing GTPase activating protein 3 | 1.6632 | 0.003428 | 558.79 | 412.63 | 827.66 | 770.65 |
| FAM90A1 | family with sequence similarity 90. member A1 | -1.0511 | 0.43217 | 134.42 | 132.64 | 122.26 | 132 |
| PHACTR3 | phosphatase and actin regulator 3 | -1.3284 | 1.31E-05 | 168.25 | 159.99 | 128.1 | 119.09 |
| TMEM43 | transmembrane protein 43 | 1.0932 | 0.056934 | 1228.9 | 1185.1 | 1385.8 | 1256 |
| GADD45B | growth arrest and DNA-damage-inducible. beta | 1.0618 | 0.20416 | 190.81 | 198.52 | 208.52 | 204.79 |
| SLC31A2 | solute carrier family 31 (copper transporters). member 2 | -1.3769 | 0.00018 | 454.24 | 376.91 | 308.5 | 292.7 |
| CHKA | choline kinase alpha | -1.9762 | 3.26E-09 | 896.75 | 887.36 | 468.93 | 434.51 |
| LRIG1 | leucine-rich repeats and immunoglobulin-like domains 1 | 1.2509 | 0.000465 | 492.27 | 495.77 | 638.46 | 598.09 |
| SLC2A3 | solute carrier family 2 (facilitated glucose transporter). member 3 | -1.5551 | 2.87E-06 | 226.46 | 191.67 | 135.68 | 132.29 |
| FAM64A | family with sequence similarity 64. member A | 1.2738 | 0.023416 | 206.98 | 176.81 | 257.6 | 230.53 |
| P4HA1 | procollagen-proline. 2-oxoglutarate 4-dioxygenase (proline 4-hydroxylase). alpha polypeptide I | 1.3914 | 0.004618 | 389.91 | 452.07 | 534.8 | 638.07 |
| ERRFI1 | ERBB receptor feedback inhibitor 1 | -1.8834 | 3.88E-07 | 2741.6 | 2235.4 | 1338 | 1291.3 |
| ZFP36 | zinc finger protein 36. C3H type. homolog (mouse) | -1.0513 | 0.36447 | 424.96 | 410.09 | 371.96 | 423.91 |
| TAF15 | TAF15 RNA polymerase II. TATA box binding protein (TBP)-associated factor. 68kDa | 1.7766 | 0.001707 | 1942.9 | 2957.9 | 4522 | 4011.1 |
| SC4MOL | sterol-C4-methyl oxidase-like | 1.2622 | 0.000383 | 244.17 | 234.51 | 285.22 | 319.84 |
| NUSAP1 | nucleolar and spindle associated protein 1 | 1.5814 | 0.007287 | 430.98 | 338.46 | 645.26 | 565.33 |
| TUBB2A | tubulin. beta 2A | -1.258 | 0.050449 | 673.25 | 658.02 | 465.5 | 601.32 |
| AP3S1 | adaptor-related protein complex 3. sigma 1 subunit | -1.0746 | 0.16166 | 581.99 | 546.43 | 511.53 | 538.38 |
| SELS | selenoprotein S | 1.5903 | 6.98E-07 | 4122.7 | 3733.3 | 6330.5 | 6149 |
| OSBPL5 | oxysterol binding protein-like 5 | -1.134 | 0.002575 | 470.37 | 457.73 | 412.63 | 405.73 |
| SLC27A3 | solute carrier family 27 (fatty acid transporter). member 3 | -1.2581 | 0.008655 | 400.27 | 350 | 305.62 | 289.61 |
| ULK1 | unc-51-like kinase 1 (C. elegans) | 1.0536 | 0.45939 | 1768.1 | 1638.4 | 1899.8 | 1692.7 |
| CCNB2 | cyclin B2 | 1.5484 | 0.006023 | 697.92 | 545.98 | 986.91 | 925.68 |
| TRIM24 | tripartite motif-containing 24 | -1.0394 | 0.52901 | 1183 | 1049.8 | 1090.2 | 1054.5 |
| VCL | vinculin | -1.0855 | 0.20731 | 5283 | 4225.6 | 4452.7 | 4254.8 |
| CMIP | c-Maf-inducing protein | -1.163 | 0.000549 | 1209.3 | 1222.1 | 1079.9 | 1011.8 |
| FBXO31 | F-box protein 31 | 1.0453 | 0.22069 | 566.73 | 572.61 | 602.57 | 588.42 |
| AASS | aminoadipate-semialdehyde synthase | 1.3012 | 8.88E-05 | 167.68 | 174.57 | 218.59 | 226.75 |
| ANKRD37 | ankyrin repeat domain 37 | 1.4788 | 1.01E-06 | 261.31 | 243.68 | 363.25 | 383.33 |
| C17orf58 | chromosome 17 open reading frame 58 | 1.2239 | 0.000321 | 470.93 | 471.08 | 581.76 | 571.25 |
| PTTG1 | pituitary tumor-transforming 1 | 1.4041 | 0.020583 | 688.84 | 501.15 | 872.17 | 780.34 |
| SCNN1G | sodium channel. nonvoltage-gated 1. gamma | -1.9949 | 6.72E-09 | 277.16 | 264.89 | 132.69 | 139.03 |
| KCTD3 | potassium channel tetramerisation domain containing 3 | 1.0103 | 0.90758 | 561.75 | 516.34 | 565.15 | 523.87 |
| CAMK2N1 | calcium/calmodulin-dependent protein kinase II inhibitor 1 | 2.3261 | 9.53E-10 | 2137.4 | 2017.6 | 4818.3 | 4842.7 |
| SLCO4A1 | solute carrier organic anion transporter family. member 4A1 | -1.2694 | 7.09E-05 | 507.88 | 486.86 | 392.92 | 390.54 |
| CREB3L2 | cAMP responsive element binding protein 3-like 2 | 1.4467 | 0.001219 | 587.18 | 563.6 | 911.54 | 759.83 |
| C17orf80 | chromosome 17 open reading frame 80 | -1.0043 | 0.94061 | 203.14 | 177.21 | 198.7 | 179.63 |
| BCL6 | B-cell CLL/lymphoma 6 (zinc finger protein 51) | -1.2077 | 0.049331 | 1804.5 | 1527.9 | 1460.8 | 1294.1 |
| NCAPG | non-SMC condensin I complex. subunit G | 1.4882 | 0.008992 | 246.91 | 209.59 | 355.91 | 322.02 |
| OLAH | oleoyl-ACP hydrolase | -1.0996 | 0.033426 | 161.05 | 152.67 | 136.62 | 148.84 |
| IRS2 | insulin receptor substrate 2 | -1.0977 | 0.17314 | 260.09 | 220.15 | 234.71 | 202.45 |
| KIAA1826 | KIAA1826 | -1.0458 | 0.63649 | 738.75 | 637.61 | 649.74 | 662.9 |
| PRC1 | protein regulator of cytokinesis 1 | 1.478 | 0.001565 | 451.49 | 374.3 | 619.59 | 595.8 |
| C9orf152 | chromosome 9 open reading frame 152 | -1.7117 | 2.16E-07 | 1432.9 | 1349.5 | 840.37 | 785.38 |
| CEBPD | CCAAT/enhancer binding protein (C/EBP). delta | -1.5508 | 7.69E-07 | 939.83 | 952.42 | 621.13 | 599.25 |
| PAK1IP1 | PAK1 interacting protein 1 | 1.038 | 0.41019 | 1120 | 1047.7 | 1163.6 | 1086.6 |
| DUSP1 | dual specificity phosphatase 1 | -1.0889 | 0.32067 | 1304.7 | 1026.4 | 1040.3 | 1085.6 |
| FAM105A | family with sequence similarity 105. member A | -1.565 | 5.84E-09 | 274.49 | 269.77 | 177.23 | 170.6 |
| CKB | creatine kinase. brain | -1.0649 | 0.2377 | 3591.1 | 3683.4 | 3437.8 | 3392.8 |
| SLC39A11 | solute carrier family 39 (metal ion transporter). member 11 | 1.0561 | 0.12196 | 380.41 | 391.87 | 407.76 | 407.76 |
| C16orf44 | chromosome 16 open reading frame 44 | -1.0267 | 0.4734 | 685.38 | 688.84 | 643.68 | 695.77 |
| CDC2 | cell division cycle 2. G1 to S and G2 to M | 1.4535 | 0.00463 | 404.98 | 339.08 | 503.76 | 575.93 |
| ALDH6A1 | aldehyde dehydrogenase 6 family. member A1 | 1.7926 | 6.56E-07 | 1437.3 | 1384.7 | 2568.2 | 2490.2 |
| KIF2C | kinesin family member 2C | 1.7805 | 3.31E-05 | 238.54 | 202.55 | 426.03 | 359.55 |
| SERPINA3 | serpin peptidase inhibitor. clade A (alpha-1 antiproteinase. antitrypsin). member 3 | -1.5689 | 1.11E-06 | 444.51 | 469.19 | 283.4 | 298.99 |
| EGFR | epidermal growth factor receptor (erythroblastic leukemia viral (v-erb-b) oncogene homolog. avian) | 1.0212 | 0.69218 | 229.3 | 211.71 | 232.21 | 218 |
| ASB9 | ankyrin repeat and SOCS box-containing 9 | -1.1038 | 0.051857 | 149.31 | 146.41 | 136.99 | 130.98 |
| STX12 | syntaxin 12 | 1.0491 | 0.32519 | 454.84 | 495.23 | 480.26 | 516.18 |
| CENPF | centromere protein F. 350/400ka (mitosin) | 1.4899 | 0.000842 | 215.47 | 178.45 | 315.73 | 270.33 |
| FAM129B | family with sequence similarity 129. member B | -1.5076 | 8.63E-05 | 4793.9 | 4157.7 | 2773.1 | 3162.3 |
| FAM83D | family with sequence similarity 83. member D | 1.6261 | 7.65E-05 | 199.93 | 177.1 | 327.28 | 286.08 |
| LBR | lamin B receptor | 1.454 | 0.000332 | 1944.4 | 1771.4 | 2780.7 | 2618.8 |
| NETO1 | neuropilin (NRP) and tolloid (TLL)-like 1 | 1.4368 | 7.61E-06 | 210.37 | 208.38 | 302.39 | 299.28 |
| PSRC1 | proline/serine-rich coiled-coil 1 | 1.5591 | 5.35E-05 | 203.25 | 181.09 | 319.68 | 279.87 |
| TMEM16F | transmembrane protein 16F | -1.1429 | 0.035529 | 289.83 | 309.9 | 272.06 | 252.75 |
| CAP2 | CAP. adenylate cyclase-associated protein. 2 (yeast) | 1.1986 | 0.060431 | 2472 | 1893.5 | 2785.5 | 2414 |
| SCAP | SREBF chaperone | -1.1529 | 0.008689 | 4261.6 | 4084.1 | 3659.7 | 3577.9 |
| NUDT16 | nudix (nucleoside diphosphate linked moiety X)-type motif 16 | -1.0233 | 0.56836 | 274.1 | 275.97 | 279.16 | 258.77 |
| HMGB3 | high-mobility group box 3 | 1.0239 | 0.59414 | 194.82 | 191.53 | 194.73 | 200.89 |
| DHCR7 | 7-dehydrocholesterol reductase | 1.2084 | 0.00157 | 795.44 | 789.97 | 1004.6 | 913.29 |
| CDH2 | cadherin 2. type 1. N-cadherin (neuronal) | 1.0264 | 0.70638 | 123.73 | 133.36 | 127.65 | 136.18 |
| TRPM4 | transient receptor potential cation channel. subfamily M. member 4 | 1.6753 | 2.21E-08 | 5132 | 5118.7 | 8627.5 | 8545.8 |
| PRKAB2 | protein kinase. AMP-activated. beta 2 non-catalytic subunit | 1.05 | 0.19147 | 236.61 | 246.72 | 250.56 | 256.85 |
| ZNF259 | zinc finger protein 259 | 1.0037 | 0.92137 | 334.95 | 362.45 | 338.77 | 360.99 |
| LDHA | lactate dehydrogenase A | 1.368 | 0.000396 | 6756.3 | 6172.3 | 8545.8 | 9132.1 |
| ZDHHC9 | zinc finger. DHHC-type containing 9 | -1.0719 | 0.28765 | 442.73 | 475.69 | 436.45 | 419.94 |
| MCFD2 | multiple coagulation factor deficiency 2 | 1.6449 | 1.24E-07 | 466.85 | 429.16 | 772.23 | 701.96 |
| KLF15 | Kruppel-like factor 15 | 1.0344 | 0.33222 | 212.59 | 212.42 | 222.77 | 216.92 |
| SHC1 | SHC (Src homology 2 domain containing) transforming protein 1 | 1.2345 | 0.000514 | 2301.8 | 2367.3 | 2937.9 | 2826.8 |
| RBM24 | RNA binding motif protein 24 | 1.4778 | 3.94E-07 | 299.65 | 298.99 | 443.45 | 441.22 |
| FADS1 | fatty acid desaturase 1 | 1.4605 | 1.50E-06 | 782.24 | 729.05 | 1101.9 | 1104 |
| CEP55 | centrosomal protein 55kDa | 1.4709 | 0.001509 | 224.67 | 184.97 | 315.22 | 285.22 |
| SLC30A7 | solute carrier family 30 (zinc transporter). member 7 | 1.6772 | 3.73E-06 | 540.65 | 512.71 | 926.24 | 841.86 |
| C5orf4 | chromosome 5 open reading frame 4 | -1.3868 | 8.00E-07 | 171.56 | 157.85 | 121.67 | 115.74 |
| CCNA2 | cyclin A2 | 1.4144 | 9.07E-05 | 203.67 | 187.71 | 278.97 | 274.18 |
| DNAJB9 | DnaJ (Hsp40) homolog. subfamily B. member 9 | 1.3983 | 0.00049 | 1517.8 | 1407.4 | 2001.5 | 2086.8 |
| CDCA8 | cell division cycle associated 8 | 1.5161 | 0.000106 | 221.12 | 200.12 | 340.26 | 298.92 |
| PFKFB4 | 6-phosphofructo-2-kinase/fructose-2.6-biphosphatase 4 | 1.3521 | 0.000137 | 412.63 | 386.45 | 525.86 | 554.4 |
| PRPS2 | phosphoribosyl pyrophosphate synthetase 2 | -1.2685 | 0.011141 | 339.75 | 362.24 | 260.66 | 293.43 |
| ADNP2 | ADNP homeobox 2 | 1.316 | 0.001487 | 816.56 | 835.89 | 1154.3 | 1024.1 |
| CALU | calumenin | 1.8612 | 2.19E-07 | 789.97 | 711.14 | 1497.2 | 1299.8 |
| ITGB1 | integrin. beta 1 (fibronectin receptor. beta polypeptide. antigen CD29 includes MDF2. MSK12) | 1.0232 | 0.73959 | 661.29 | 718.71 | 711.14 | 699.71 |
| VPS33B | vacuolar protein sorting 33 homolog B (yeast) | -1.0212 | 0.64233 | 282.98 | 272.15 | 273.79 | 269.74 |
| CTDSPL | CTD (carboxy-terminal domain. RNA polymerase II. polypeptide A) small phosphatase-like | -1.0804 | 0.11204 | 640.32 | 674.57 | 592.49 | 624.57 |
| AURKB | aurora kinase B | 1.4939 | 1.98E-05 | 200.15 | 182.34 | 298.82 | 272.55 |
| C8orf42 | chromosome 8 open reading frame 42 | -1.0478 | 0.31913 | 375.66 | 316.36 | 325.1 | 332.94 |
| SCNN1A | sodium channel. nonvoltage-gated 1 alpha | -1.4377 | 2.07E-05 | 4636.9 | 4697.9 | 3142.4 | 3353.6 |
| ABHD2 | abhydrolase domain containing 2 | -1.1294 | 0.12096 | 513.35 | 482.05 | 452.9 | 428.32 |
| EFNA1 | ephrin-A1 | -1.0584 | 0.10391 | 3356.7 | 3444.4 | 3175.5 | 3249.9 |
| DOCK5 | dedicator of cytokinesis 5 | 1.0525 | 0.29033 | 198.66 | 184.87 | 205.59 | 197.91 |
| ELL2 | elongation factor. RNA polymerase II. 2 | -1.5294 | 2.07E-07 | 334.21 | 324.37 | 221.3 | 209.43 |
| HIST1H4C | histone cluster 1. H4c | 1.6898 | 1.92E-05 | 5879.9 | 5996.2 | 10576 | 9519 |
| CST3 | cystatin C (amyloid angiopathy and cerebral hemorrhage) | -1.1125 | 0.010361 | 5621.3 | 6131.2 | 5166.9 | 5390.1 |
| NT5DC3 | 5'-nucleotidase domain containing 3 | -1.3216 | 7.96E-06 | 580.36 | 568.35 | 422.04 | 447.47 |
| MED30 | mediator complex subunit 30 | 1.6239 | 4.20E-06 | 415.18 | 385.6 | 658.92 | 640.72 |
| KLF9 | Kruppel-like factor 9 | 1.007 | 0.91666 | 2504.8 | 2281.8 | 2387.7 | 2427.5 |
| CTH | cystathionase (cystathionine gamma-lyase) | 1.4922 | 7.00E-08 | 1252 | 1287.7 | 1901.6 | 1887.6 |
| CKS1B | CDC28 protein kinase regulatory subunit 1B | 1.7183 | 0.00024 | 1560.8 | 1280.7 | 2712.9 | 2175.4 |
| HSPA5 | heat shock 70kDa protein 5 (glucose-regulated protein. 78kDa) | 2.28 | 2.77E-08 | 594.07 | 511.28 | 1255.1 | 1258 |
| FAT | FAT tumor suppressor homolog 1 (Drosophila) | -1.466 | 3.50E-05 | 778.33 | 757.06 | 518.76 | 528.52 |
| KRT8 | keratin 8 | -1.2482 | 0.000545 | 2314.9 | 2205.1 | 1846.6 | 1774.2 |
| AARS | alanyl-tRNA synthetase | 1.3094 | 0.003784 | 10014 | 9563.3 | 13330 | 12317 |
| CLPTM1L | CLPTM1-like | 1.1672 | 0.064258 | 1470.4 | 1563.5 | 1853.1 | 1690.3 |
| NNT | nicotinamide nucleotide transhydrogenase | -1.4469 | 0.000251 | 213.37 | 180.63 | 138.2 | 133.21 |
| BIRC3 | baculoviral IAP repeat-containing 3 | -1.9056 | 3.73E-06 | 338.97 | 299.41 | 171.43 | 163.04 |
| KIAA0182 | KIAA0182 | 1.0707 | 0.2424 | 1363.6 | 1314.4 | 1456.3 | 1410.9 |
| BAT1 | HLA-B associated transcript 1 | 1.5513 | 1.45E-06 | 1599.5 | 1425.5 | 2295.8 | 2390 |
| PHF17 | PHD finger protein 17 | 1.0495 | 0.23751 | 425.41 | 405.1 | 437.76 | 433.65 |
| FOXO3 | forkhead box O3 | -1.3973 | 0.000155 | 4029.9 | 3604.5 | 2807.4 | 2650.2 |
| NDRG1 | N-myc downstream regulated gene 1 | 1.1457 | 0.046207 | 802.47 | 723.31 | 804.51 | 946.96 |
| UNC84B | unc-84 homolog B (C. elegans) | 1.1573 | 0.023602 | 351.99 | 375.66 | 430.21 | 411.65 |
| ZNF364 | zinc finger protein 364 | -1.2837 | 0.001004 | 867.9 | 888 | 712.7 | 656.25 |
| CDCA5 | cell division cycle associated 5 | 1.5724 | 2.60E-05 | 258.83 | 227.85 | 391.95 | 372.04 |
| STK35 | serine/threonine kinase 35 | 1.0101 | 0.88019 | 927.7 | 960.37 | 971.87 | 935.25 |
| WDR60 | WD repeat domain 60 | 1.044 | 0.52582 | 295.56 | 242.65 | 288.78 | 270.7 |
| KIAA0101 | KIAA0101 | 1.488 | 0.000408 | 316.01 | 264.18 | 458.92 | 402.77 |
| TNFRSF19 | tumor necrosis factor receptor superfamily. member 19 | 1.3036 | 0.000295 | 271.24 | 257.73 | 369.31 | 321.68 |
| ARMET | arginine-rich. mutated in early stage tumors | 2.0844 | 1.58E-10 | 8043.4 | 7875.8 | 16409 | 16774 |
| DENND1A | DENN/MADD domain containing 1A | 1.4254 | 3.08E-06 | 1093.8 | 1104 | 1617.3 | 1517 |
| PDIA5 | protein disulfide isomerase family A. member 5 | 1.3444 | 0.001152 | 610.74 | 613.33 | 826.32 | 819.31 |
| PDZD8 | PDZ domain containing 8 | 1.1059 | 0.075253 | 290.96 | 280.53 | 325.31 | 306.88 |
| PHLDA1 | pleckstrin homology-like domain. family A. member 1 | 1.0022 | 0.95942 | 147.58 | 134.37 | 136.49 | 145.94 |
| ATP11B | ATPase. Class VI. type 11B | 1.1678 | 0.005625 | 191.55 | 221.49 | 241.01 | 240.08 |
| USP33 | ubiquitin specific peptidase 33 | 1.1629 | 0.033531 | 182.27 | 194.92 | 205.36 | 233.96 |
| SLC2A1 | solute carrier family 2 (facilitated glucose transporter). member 1 | 1.2474 | 0.000299 | 187.58 | 170.91 | 224.63 | 222.08 |
| PTPRM | protein tyrosine phosphatase. receptor type. M | 1.0318 | 0.58463 | 510.53 | 490.17 | 568.14 | 468.93 |
| DCXR | dicarbonyl/L-xylulose reductase | 1.2142 | 0.000812 | 3090.3 | 3437.8 | 3947.5 | 3967.8 |
| TCEAL3 | transcription elongation factor A (SII)-like 3 | 1.0211 | 0.69825 | 660.3 | 576.48 | 626.06 | 633.93 |
| FUT3 | fucosyltransferase 3 (galactoside 3(4)-L-fucosyltransferase. Lewis blood group) | 1.1216 | 0.036016 | 189.68 | 192.6 | 217.94 | 210.88 |
| TXLNA | taxilin alpha | 1.0365 | 0.61377 | 3870.5 | 3663.3 | 3897.7 | 3908.4 |
| LOX | lysyl oxidase | 1.0064 | 0.87683 | 125.75 | 117.94 | 123.82 | 121.31 |
| MMP25 | matrix metallopeptidase 25 | -1.0443 | 0.26507 | 139.21 | 140.36 | 137.53 | 130.29 |
| GBA | glucosidase. beta; acid (includes glucosylceramidase) | 1.0905 | 0.11865 | 301.29 | 308.5 | 340.97 | 324.17 |
| ADM | adrenomedullin | 1.5085 | 2.49E-06 | 217.94 | 182.74 | 302.29 | 299.8 |
| HECTD2 | HECT domain containing 2 | 1.4305 | 2.14E-08 | 200.82 | 207.27 | 289.83 | 293.87 |
| C5orf13 | chromosome 5 open reading frame 13 | -1.1359 | 0.02986 | 511.1 | 505.47 | 422.66 | 473.71 |
| SYT4 | synaptotagmin IV | 1.259 | 0.001722 | 3351 | 3473.7 | 4281.2 | 4309.9 |
| MACROD1 | MACRO domain containing 1 | 1.6744 | 2.35E-08 | 295.75 | 283.58 | 464.68 | 506 |
| CD55 | CD55 molecule. decay accelerating factor for complement (Cromer blood group) | 1.8425 | 3.60E-07 | 581.37 | 652.28 | 1083.1 | 1188.6 |
| TMED5 | transmembrane emp24 protein transport domain containing 5 | 1.7326 | 0.000321 | 2014 | 1557.6 | 3230.6 | 2914.9 |
| GLUD1 | glutamate dehydrogenase 1 | -1.0308 | 0.46008 | 4088.8 | 4254.8 | 4225.6 | 3874.9 |
| SRF | serum response factor (c-fos serum response element-binding transcription factor) | 1.8924 | 2.79E-08 | 1274 | 1151.9 | 2364.5 | 2222.8 |
| MFGE8 | milk fat globule-EGF factor 8 protein | -1.4506 | 1.08E-05 | 506.81 | 491.78 | 354.31 | 334.3 |
| MYADM | myeloid-associated differentiation marker | 1.6011 | 0.000102 | 333.94 | 351.46 | 527.4 | 570.47 |
| SDF2L1 | stromal cell-derived factor 2-like 1 | 1.7822 | 1.23E-07 | 2162.3 | 2237.2 | 4147.5 | 3704.8 |
| PUS1 | pseudouridylate synthase 1 | -1.0199 | 0.68878 | 1175 | 1110.8 | 1084.6 | 1157 |
| IFNGR1 | interferon gamma receptor 1 | -1.1016 | 0.13452 | 1182.1 | 1078.6 | 1094.9 | 959.63 |
| H2AFZ | H2A histone family. member Z | 1.0689 | 0.23076 | 13782 | 11906 | 13869 | 13518 |
| C12orf44 | chromosome 12 open reading frame 44 | -1.1804 | 0.021355 | 1073.8 | 1041.3 | 888 | 903.64 |
| PDPK1 | 3-phosphoinositide dependent protein kinase-1 | -1.0407 | 0.37549 | 355.36 | 380.18 | 340.8 | 366 |
| PLEKHC1 | pleckstrin homology domain containing. family C (with FERM domain) member 1 | -1.1394 | 0.048611 | 940.38 | 927.7 | 852.84 | 787.94 |
| C17orf48 | chromosome 17 open reading frame 48 | -1.3194 | 1.95E-06 | 458.26 | 466.85 | 362.02 | 339.46 |
| RALY | RNA binding protein. autoantigenic (hnRNP-associated with lethal yellow homolog (mouse)) | -1.1465 | 0.016486 | 1038.2 | 1079.4 | 973.32 | 875.95 |
| MYBPC1 | myosin binding protein C. slow type | -1.0842 | 0.082233 | 168.02 | 158.28 | 149.27 | 151.56 |
| ALDOC | aldolase C. fructose-bisphosphate | 1.2688 | 0.000586 | 2680.9 | 2554.8 | 3277.1 | 3364.7 |
| TSC2 | tuberous sclerosis 2 | -1.4315 | 2.98E-05 | 539.71 | 606.32 | 389.12 | 410.4 |
| STRBP | spermatid perinuclear RNA binding protein | -1.2285 | 0.039549 | 670.69 | 558.08 | 514.91 | 481.62 |
| CNN2 | calponin 2 | 1.0716 | 0.16664 | 525.24 | 497.32 | 534.44 | 561.28 |
| TSC22D3 | TSC22 domain family. member 3 | -1.6846 | 5.26E-07 | 4427.2 | 3971.9 | 2470.3 | 2508.3 |
| DNAJB11 | DnaJ (Hsp40) homolog. subfamily B. member 11 | 1.8298 | 2.32E-07 | 4360.7 | 3521.8 | 7112.7 | 7229.6 |
| MBNL1 | muscleblind-like (Drosophila) | -1.1084 | 0.17255 | 622.22 | 502.84 | 501.59 | 507.69 |
| LDLR | low density lipoprotein receptor (familial hypercholesterolemia) | 1.6199 | 3.53E-07 | 1215.6 | 1020.2 | 1791.3 | 1816.7 |
| CLEC16A | C-type lectin domain family 16. member A | -1.2477 | 0.006333 | 621.4 | 580.85 | 515.34 | 449.93 |
| TDRD9 | tudor domain containing 9 | -1.3345 | 8.12E-06 | 162.62 | 157.79 | 120.35 | 119.72 |
| CBX4 | chromobox homolog 4 (Pc class homolog. Drosophila) | 1.519 | 1.65E-07 | 1084.1 | 1111.6 | 1725 | 1612 |
| RPN1 | ribophorin I | 1.2115 | 0.00183 | 6399.7 | 6354.3 | 8015.2 | 7446.4 |
| LGMN | legumain | 1.0551 | 0.42771 | 1343.4 | 1530.8 | 1454.7 | 1573.8 |
| PDHA1 | pyruvate dehydrogenase (lipoamide) alpha 1 | -1.0751 | 0.19937 | 1830.1 | 2021.1 | 1756.6 | 1821.7 |
| KLF6 | Kruppel-like factor 6 | 1.1913 | 0.018171 | 741.43 | 767.04 | 877.89 | 919.42 |
| BCKDHB | branched chain keto acid dehydrogenase E1. beta polypeptide (maple syrup urine disease) | 1.4013 | 0.000167 | 417.54 | 417.02 | 620.39 | 551.13 |
| AXUD1 | AXIN1 up-regulated 1 | -1.2533 | 0.001188 | 773.35 | 711.7 | 608.06 | 576.26 |
| SLC38A2 | solute carrier family 38. member 2 | -1.0867 | 0.47297 | 2136.1 | 2125.4 | 1787.1 | 2151.2 |
| SGSM2 | small G protein signaling modulator 2 | -1.0709 | 0.21204 | 1980.9 | 1931.1 | 1813.9 | 1838.8 |
| SLC38A1 | solute carrier family 38. member 1 | -1.1672 | 0.004352 | 2377.7 | 2270 | 1982.1 | 1998.8 |
| FLVCR2 | feline leukemia virus subgroup C cellular receptor family. member 2 | -1.053 | 0.27878 | 296.09 | 274.65 | 289.29 | 253.52 |
| SPOCK1 | sparc/osteonectin. cwcv and kazal-like domains proteoglycan (testican) 1 | -1.2021 | 0.000597 | 174.89 | 164.81 | 141.72 | 140.74 |
| TPCN2 | two pore segment channel 2 | 1.0446 | 0.36687 | 321.22 | 311.75 | 336.68 | 324.54 |
| TGIF1 | TGFB-induced factor homeobox 1 | 1.5242 | 4.35E-06 | 436.21 | 420.29 | 684.77 | 621.99 |
| DDC | dopa decarboxylase (aromatic L-amino acid decarboxylase) | 1.5455 | 5.77E-05 | 5057.9 | 6040.7 | 8769.2 | 8321.8 |
| SSR1 | signal sequence receptor. alpha (translocon-associated protein alpha) | 1.5534 | 3.68E-05 | 3094.5 | 2651.9 | 4437.4 | 4462.7 |
| DDOST | dolichyl-diphosphooligosaccharide-protein glycosyltransferase | 1.5069 | 9.56E-07 | 2919.8 | 3277.1 | 4532 | 4793.9 |
| CDR2 | cerebellar degeneration-related protein 2. 62kDa | 1.6936 | 1.31E-06 | 389.12 | 365.55 | 644.97 | 632.58 |
| PPP3R1 | protein phosphatase 3 (formerly 2B). regulatory subunit B. alpha isoform | 1.518 | 4.41E-06 | 841.86 | 855.48 | 1337 | 1241.2 |
| SPSB1 | splA/ryanodine receptor domain and SOCS box containing 1 | -1.449 | 8.47E-07 | 233.26 | 214.7 | 159.57 | 149.47 |
| TP53BP2 | tumor protein p53 binding protein. 2 | 1.123 | 0.1026 | 648.35 | 584.04 | 726.45 | 657.38 |
| F2RL1 | coagulation factor II (thrombin) receptor-like 1 | -1.4117 | 0.001428 | 938.6 | 875.95 | 669.76 | 615.97 |
| SLC39A1 | solute carrier family 39 (zinc transporter). member 1 | 1.1718 | 0.034662 | 614.2 | 582.89 | 697.67 | 704.63 |
| FAM58A | family with sequence similarity 58. member A | -1.0497 | 0.23669 | 503.22 | 464.18 | 477.73 | 443.72 |
| GRB10 | growth factor receptor-bound protein 10 | 1.3643 | 0.000398 | 970.16 | 1116.3 | 1466.6 | 1374.5 |
| CRELD2 | cysteine-rich with EGF-like domains 2 | 1.7633 | 6.54E-08 | 2930.7 | 2780.7 | 4858.8 | 5215 |
| ERICH1 | glutamate-rich 1 | -1.0951 | 0.03769 | 296.76 | 298.92 | 269.38 | 274.59 |
| ZCCHC11 | zinc finger. CCHC domain containing 11 | 1.0733 | 0.30962 | 340.26 | 306.06 | 360.21 | 333.02 |
| FLJ37464 | hypothetical protein FLJ37464 | -1.0995 | 0.16452 | 151.92 | 137.65 | 128.53 | 134.58 |
| GOLPH3L | golgi phosphoprotein 3-like | 1.598 | 2.84E-06 | 957.48 | 938.6 | 1551.7 | 1478.9 |
| LMO4 | LIM domain only 4 | 1.2603 | 0.008211 | 1186.4 | 1004.1 | 1440.2 | 1313.7 |
| MYO6 | myosin VI | 1.4203 | 0.000914 | 861.69 | 904.42 | 1353.8 | 1161.2 |
| HYOU1 | hypoxia up-regulated 1 | 1.6256 | 3.56E-08 | 354.67 | 351.81 | 564.99 | 583.64 |
| ZNF23 | zinc finger protein 23 (KOX 16) | 1.0131 | 0.78788 | 218.11 | 216.96 | 210.31 | 230.95 |
| CDH15 | cadherin 15. M-cadherin (myotubule) | 1.4018 | 4.52E-07 | 177.36 | 178.04 | 248.07 | 250.15 |
| ENPP4 | ectonucleotide pyrophosphatase/phosphodiesterase 4 (putative function) | 1.2898 | 0.003726 | 396.21 | 342.7 | 499.92 | 451.79 |
| ERN1 | endoplasmic reticulum to nucleus signaling 1 | -1.1386 | 0.006599 | 1195.6 | 1059.3 | 1004.1 | 972.84 |
| PYGB | phosphorylase. glycogen; brain | -1.2274 | 0.001418 | 1807 | 1780.5 | 1504 | 1420 |
| ACTB | actin. beta | 1.1107 | 0.25395 | 8471.8 | 7314.3 | 8969.7 | 8522.1 |
| SEC23B | Sec23 homolog B (S. cerevisiae) | 1.3386 | 1.79E-06 | 2130.5 | 1997.3 | 2837.2 | 2687.4 |
| PEG3 | paternally expressed 3 | 1.3449 | 0.000133 | 333.52 | 324.06 | 467.99 | 417.74 |
| LRRFIP2 | leucine rich repeat (in FLII) interacting protein 2 | -1.0972 | 0.033924 | 594.9 | 549.87 | 510 | 532.78 |
| TMED9 | transmembrane emp24 protein transport domain containing 9 | 1.5877 | 5.97E-06 | 2281.8 | 2551.4 | 3848.1 | 3813.4 |
| ERGIC1 | endoplasmic reticulum-golgi intermediate compartment (ERGIC) 1 | 1.4226 | 0.000499 | 2881 | 3302.7 | 4385.4 | 4391.3 |
| BBS1 | Bardet-Biedl syndrome 1 | -1.0706 | 0.1302 | 390.07 | 422.23 | 392.52 | 366.09 |
| SOCS2 | suppressor of cytokine signaling 2 | -1.117 | 0.013409 | 217.61 | 214.04 | 191.37 | 195.09 |
| MDC1 | mediator of DNA damage checkpoint 1 | 1.5065 | 5.95E-06 | 456.24 | 418.53 | 654.39 | 662.28 |
| TFF3 | trefoil factor 3 (intestinal) | 1.385 | 0.000169 | 347.29 | 403.67 | 509.18 | 528.15 |
| GOT1 | glutamic-oxaloacetic transaminase 1. soluble (aspartate aminotransferase 1) | 1.2924 | 0.002047 | 4025.7 | 3428.4 | 4827.4 | 4775.6 |
| B2M | beta-2-microglobulin | -1.2979 | 0.018551 | 3740.6 | 4001.9 | 2753.7 | 3226.8 |
| DC2 | DC2 protein | 1.548 | 5.58E-07 | 3011 | 2973 | 4775.6 | 4491.6 |
| TF | transferrin | -1.0175 | 0.66483 | 131.43 | 132.75 | 129.49 | 130.14 |
| EAF1 | ELL associated factor 1 | -1.0852 | 0.61497 | 344.35 | 430.3 | 314.6 | 399.91 |
| ZMYND8 | zinc finger. MYND-type containing 8 | 1.0767 | 0.078342 | 305.29 | 281.38 | 315.83 | 315.31 |
| TSPAN3 | tetraspanin 3 | -1.1249 | 0.028568 | 1030.6 | 1158.2 | 964.55 | 978.02 |
| SH3PX3 | SH3 and PX domain containing 3 | -1.0728 | 0.04625 | 163.64 | 159.39 | 154.38 | 146.8 |
| ABCD3 | ATP-binding cassette. sub-family D (ALD). member 3 | -1.1784 | 0.000592 | 354.58 | 365.46 | 302.74 | 308.23 |
| FNDC3B | fibronectin type III domain containing 3B | 1.3282 | 0.000956 | 374.78 | 387.05 | 530.87 | 482.05 |
| CCPG1 | cell cycle progression 1 | 1.2839 | 0.008674 | 483.96 | 451.49 | 623.23 | 577.9 |
| NBN | nibrin | -1.0834 | 0.33794 | 651.17 | 586.46 | 599.75 | 542.44 |
| PALLD | palladin. cytoskeletal associated protein | 1.0794 | 0.3268 | 834.48 | 679.58 | 834.85 | 791.47 |
| PDLIM1 | PDZ and LIM domain 1 (elfin) | -1.2412 | 0.009734 | 325.21 | 362.34 | 281.56 | 271.65 |
| CDKN3 | cyclin-dependent kinase inhibitor 3 (CDK2-associated dual specificity phosphatase) | 1.2055 | 0.072038 | 380.89 | 313.89 | 422.99 | 410.78 |
| TMEM61 | transmembrane protein 61 | -1.0088 | 0.79925 | 146.2 | 148.1 | 146.9 | 144.84 |
| ACAT2 | acetyl-Coenzyme A acetyltransferase 2 (acetoacetyl Coenzyme A thiolase) | 1.0772 | 0.016408 | 1326.7 | 1350.7 | 1468.9 | 1415.6 |
| ARG1 | arginase. liver | -1.4027 | 2.32E-07 | 166.28 | 174.06 | 120.83 | 121.74 |
| RANBP10 | RAN binding protein 10 | 1.0083 | 0.87238 | 244.67 | 255.21 | 256.85 | 247.13 |
| IL2RB | interleukin 2 receptor. beta | -1.0833 | 0.1077 | 164.53 | 158.15 | 157 | 141.22 |
| PRAC | small nuclear protein PRAC | 1.4697 | 4.01E-06 | 10313 | 10166 | 15777 | 14353 |
| WDR54 | WD repeat domain 54 | 1.2378 | 0.001716 | 1243.4 | 1373.5 | 1562.6 | 1674.5 |
| AKR1D1 | aldo-keto reductase family 1. member D1 (delta 4-3-ketosteroid-5-beta-reductase) | -1.0825 | 0.01516 | 129.69 | 124.12 | 117.65 | 116.75 |
| ZNF165 | zinc finger protein 165 | 1.5044 | 2.22E-05 | 296.46 | 272.52 | 401.09 | 455.9 |
| EGFLAM | EGF-like. fibronectin type III and laminin G domains | -1.0101 | 0.72407 | 133.22 | 133.27 | 130.86 | 132.95 |
| USP38 | ubiquitin specific peptidase 38 | 1.1423 | 0.048414 | 551.72 | 522.14 | 582.13 | 645.76 |
| ITPR3 | inositol 1.4.5-triphosphate receptor. type 3 | -1.1171 | 0.030551 | 1257.4 | 1147.6 | 1091.6 | 1059.3 |
| C6orf85 | chromosome 6 open reading frame 85 | -1.4344 | 2.96E-05 | 583.81 | 588.67 | 437.28 | 381.96 |
| PNPLA8 | patatin-like phospholipase domain containing 8 | 1.0398 | 0.67573 | 273.42 | 292.5 | 275.6 | 313.75 |
| C15orf23 | chromosome 15 open reading frame 23 | -1.0661 | 0.33712 | 238.69 | 216.25 | 232.4 | 195.43 |
| NLGN1 | neuroligin 1 | 1.442 | 3.74E-07 | 183.14 | 173.13 | 262.57 | 251.12 |
| VIL2 | villin 2 (ezrin) | -1.2949 | 7.04E-05 | 2091.9 | 2134.7 | 1682.3 | 1583 |
| RPS6KA2 | ribosomal protein S6 kinase. 90kDa. polypeptide 2 | -1.1374 | 0.001243 | 1334.2 | 1338 | 1175.7 | 1173.7 |
| KCNG1 | potassium voltage-gated channel. subfamily G. member 1 | -1.0115 | 0.8653 | 429.47 | 482.66 | 418.53 | 484.08 |
| ZFAND5 | zinc finger. AN1-type domain 5 | 1.0604 | 0.3125 | 4437.4 | 4244.1 | 4672.6 | 4532 |
| RGS19 | regulator of G-protein signaling 19 | -1.1856 | 0.001351 | 192.94 | 190.94 | 164.35 | 159.47 |
| KIAA1009 | KIAA1009 | 1.0262 | 0.41708 | 180.77 | 177.86 | 183.74 | 184.26 |
| BBS10 | Bardet-Biedl syndrome 10 | -1.0369 | 0.47339 | 266.85 | 255.86 | 262.67 | 241.76 |
| APBB3 | amyloid beta (A4) precursor protein-binding. family B. member 3 | 1.0025 | 0.96686 | 942.94 | 878.57 | 868.76 | 958.37 |
| DSP | desmoplakin | -1.0965 | 0.062936 | 268.29 | 271.54 | 259.3 | 233.67 |
| TXNDC5 | thioredoxin domain containing 5 | 1.5436 | 6.66E-07 | 1738.5 | 2015 | 2887.6 | 2890.5 |
| VEZF1 | vascular endothelial zinc finger 1 | 1.0298 | 0.58835 | 980.45 | 891.59 | 973.85 | 952 |
| ACTG1 | actin. gamma 1 | 1.6624 | 8.88E-07 | 2141.2 | 2130.5 | 3341.4 | 3772.8 |
| HADH | hydroxyacyl-Coenzyme A dehydrogenase | -1.0208 | 0.62349 | 2349.2 | 2405.1 | 2310.4 | 2346.7 |
| FXYD3 | FXYD domain containing ion transport regulator 3 | -1.1299 | 0.012778 | 180.27 | 174.45 | 161.86 | 152.2 |
| RCL1 | RNA terminal phosphate cyclase-like 1 | -1.0562 | 0.11935 | 421.33 | 418 | 408.28 | 386.65 |
| STK39 | serine threonine kinase 39 (STE20/SPS1 homolog. yeast) | -1.534 | 0.000168 | 4834.1 | 3659.7 | 2795.1 | 2689.9 |
| SLC4A7 | solute carrier family 4. sodium bicarbonate cotransporter. member 7 | -1.0383 | 0.6343 | 210.2 | 169.71 | 187.63 | 176.36 |
| NFIL3 | nuclear factor. interleukin 3 regulated | 1.019 | 0.69771 | 411.84 | 396.04 | 417.02 | 406.11 |
| AZI2 | 5-azacytidine induced 2 | -1.0034 | 0.94985 | 323.61 | 336.51 | 318.94 | 339.14 |
| SLC7A8 | solute carrier family 7 (cationic amino acid transporter. y+ system). member 8 | 1.0804 | 0.025927 | 329.59 | 316.84 | 346.08 | 352.23 |
| SESN1 | sestrin 1 | -1.5418 | 6.73E-06 | 959.63 | 996.31 | 677.86 | 593.36 |
| PRKCE | protein kinase C. epsilon | -1.0596 | 0.12852 | 173.08 | 173.41 | 165.77 | 161.26 |
| CDK2AP2 | CDK2-associated protein 2 | 1.5468 | 8.67E-08 | 845.22 | 858.36 | 1295.8 | 1339.6 |
| STYK1 | serine/threonine/tyrosine kinase 1 | -1.0935 | 0.091405 | 194.87 | 165.88 | 169.34 | 159.64 |
| ADFP | adipose differentiation-related protein | -1.0442 | 0.36653 | 155.94 | 143.26 | 149.37 | 137.16 |
| SSR2 | signal sequence receptor. beta (translocon-associated protein beta) | 1.6408 | 7.70E-05 | 1731.4 | 2103.4 | 2957.9 | 3314.7 |
| ABHD6 | abhydrolase domain containing 6 | -1.111 | 0.061224 | 295.69 | 291.02 | 277.51 | 251.21 |
| NOTCH2 | Notch homolog 2 (Drosophila) | -1.2354 | 0.055851 | 256.96 | 299.74 | 220.45 | 228.91 |
| ABCC8 | ATP-binding cassette. sub-family C (CFTR/MRP). member 8 | -1.4236 | 2.72E-06 | 199.74 | 192.35 | 137.8 | 137.58 |
| SMPDL3B | sphingomyelin phosphodiesterase. acid-like 3B | -1.1722 | 0.002176 | 365.1 | 384.37 | 309.56 | 329.92 |
| C12orf24 | chromosome 12 open reading frame 24 | 1.1669 | 0.002457 | 1313.7 | 1260.9 | 1445 | 1560.8 |
| CCDC6 | coiled-coil domain containing 6 | -1.0853 | 0.18794 | 1717.1 | 1783.3 | 1547.2 | 1680.1 |
| KCNH2 | potassium voltage-gated channel. subfamily H (eag-related). member 2 | -1.0878 | 0.020705 | 147.9 | 142.38 | 129.25 | 137.69 |
| ASCC1 | activating signal cointegrator 1 complex subunit 1 | -1.1171 | 0.034068 | 1294.9 | 1306.6 | 1123.2 | 1207.2 |
| USP10 | ubiquitin specific peptidase 10 | -1.0059 | 0.93746 | 554.79 | 486.36 | 541.73 | 492.27 |
| ADAMTS8 | ADAM metallopeptidase with thrombospondin type 1 motif. 8 | -1.0577 | 0.3988 | 147.19 | 133.61 | 136.01 | 129.26 |
| SNAI2 | snail homolog 2 (Drosophila) | -1.2418 | 8.80E-05 | 170.75 | 159.34 | 130.42 | 135.28 |
| MNX1 | motor neuron and pancreas homeobox 1 | 1.5951 | 1.27E-06 | 243.93 | 249.11 | 372.33 | 415.26 |
| MSX2 | msh homeobox 2 | -1.0225 | 0.59082 | 143.47 | 142.37 | 138.21 | 141.36 |
| JAG1 | jagged 1 (Alagille syndrome) | -1.2665 | 1.26E-05 | 243.36 | 236.12 | 193.19 | 185.42 |
| C1orf122 | chromosome 1 open reading frame 122 | 1.0096 | 0.88593 | 1125.9 | 1063.8 | 1050.7 | 1161.9 |
| MCM7 | minichromosome maintenance complex component 7 | 1.16 | 0.021659 | 1332 | 1614.6 | 1663.3 | 1739.6 |
| CYR61 | cysteine-rich. angiogenic inducer. 61 | 1.1836 | 0.000636 | 165.94 | 163.43 | 195.88 | 193.95 |
| SLC35C1 | solute carrier family 35. member C1 | -1.0962 | 0.087179 | 277.69 | 255.72 | 240.79 | 245.42 |
| KLHL2 | kelch-like 2. Mayven (Drosophila) | -1.0317 | 0.64316 | 302.88 | 269.9 | 272.37 | 281.96 |
| PRKAG2 | protein kinase. AMP-activated. gamma 2 non-catalytic subunit | -1.1359 | 0.00325 | 388.09 | 375.21 | 336.95 | 334.95 |
| MAP1LC3B | microtubule-associated protein 1 light chain 3 beta | 1.2825 | 0.000449 | 1482.1 | 1275.3 | 1785.8 | 1741 |
| LIFR | leukemia inhibitory factor receptor alpha | -1.0801 | 0.19456 | 144.16 | 142.85 | 128.37 | 137.52 |
| AKAP14 | A kinase (PRKA) anchor protein 14 | 1.0372 | 0.37906 | 119.81 | 121.62 | 128.91 | 121.58 |
| STAT3 | signal transducer and activator of transcription 3 (acute-phase response factor) | -1.0739 | 0.11423 | 390.41 | 398.36 | 381.96 | 353.05 |
| DUSP4 | dual specificity phosphatase 4 | 1.1396 | 0.01261 | 221.69 | 233.55 | 269.02 | 249.94 |
| HES6 | hairy and enhancer of split 6 (Drosophila) | 1.3602 | 0.001267 | 5198.8 | 4360.7 | 6254.6 | 6706.4 |
| KIAA1199 | KIAA1199 | -1.3997 | 0.000127 | 270.1 | 257.56 | 188 | 188.87 |
| EDG3 | endothelial differentiation. sphingolipid G-protein-coupled receptor. 3 | -1.1314 | 0.004332 | 157.33 | 155.58 | 142.51 | 134.18 |
| PHLDA2 | pleckstrin homology-like domain. family A. member 2 | -1.6619 | 1.56E-06 | 477.61 | 422.52 | 270.48 | 270.14 |
| C1orf198 | chromosome 1 open reading frame 198 | -1.1232 | 0.013959 | 432.85 | 484.89 | 403.67 | 412.13 |
| SAFB2 | scaffold attachment factor B2 | -1.279 | 0.000583 | 678.51 | 623.56 | 492.27 | 525.4 |
| C1RL | complement component 1. r subcomponent-like | -1.2608 | 0.007355 | 497.38 | 387.8 | 354.58 | 342.2 |
| FKBP11 | FK506 binding protein 11. 19 kDa | 1.5739 | 1.05E-06 | 1226.3 | 1359.7 | 1965.5 | 2101.3 |
| TBC1D8 | TBC1 domain family. member 8 (with GRAM domain) | -1.3084 | 2.11E-06 | 261.13 | 254.3 | 191.39 | 202.69 |
| ANAPC10 | anaphase promoting complex subunit 10 | 1.5273 | 7.52E-06 | 352.67 | 341.34 | 538.38 | 521.57 |
| TACC2 | transforming. acidic coiled-coil containing protein 2 | -1.2361 | 0.025908 | 3242.6 | 2644.8 | 2545.2 | 2205.1 |
| ELOVL1 | elongation of very long chain fatty acids (FEN1/Elo2. SUR4/Elo3. yeast)-like 1 | 1.0725 | 0.29532 | 397.11 | 412.38 | 434.51 | 433.51 |
| RNASEH1 | ribonuclease H1 | 1.5129 | 3.69E-07 | 573.46 | 535.49 | 855.98 | 821.18 |
| B4GALT1 | UDP-Gal:betaGlcNAc beta 1.4- galactosyltransferase. polypeptide 1 | -1.2799 | 0.069747 | 500.79 | 636.5 | 412 | 472.3 |
| HARS | histidyl-tRNA synthetase | -1.0506 | 0.24886 | 1988.3 | 2090.6 | 2017.6 | 1866.6 |
| FBXO18 | F-box protein. helicase. 18 | -1.1098 | 0.031627 | 1124.2 | 1185.8 | 1017.9 | 1063.3 |
| PHYHD1 | phytanoyl-CoA dioxygenase domain containing 1 | -1.0666 | 0.052063 | 137.12 | 134.44 | 129.48 | 125.14 |
| PHACTR2 | phosphatase and actin regulator 2 | -1.1351 | 0.034587 | 363.81 | 339.46 | 314.98 | 304.3 |
| CLINT1 | clathrin interactor 1 | 1.2351 | 0.006166 | 1054.8 | 927.24 | 1266.1 | 1178.4 |
| LEPREL1 | leprecan-like 1 | -1.16 | 0.00075 | 205.44 | 198.84 | 177.53 | 171 |
| PLCG1 | phospholipase C. gamma 1 | -1.2125 | 0.007172 | 380.76 | 364.74 | 293.78 | 321.53 |
| ZNF57 | zinc finger protein 57 | -1.0223 | 0.61041 | 158.69 | 168.55 | 156.76 | 163.27 |
| KIAA1324L | KIAA1324-like | -1.3206 | 0.000713 | 483.66 | 447.47 | 372.49 | 333.16 |
| HBQ1 | hemoglobin. theta 1 | 1.3697 | 4.48E-06 | 285.55 | 276.39 | 391.65 | 378.04 |
| JAK2 | Janus kinase 2 (a protein tyrosine kinase) | 1.0301 | 0.59117 | 250.02 | 226.07 | 241.94 | 247.89 |
| NCKIPSD | NCK interacting protein with SH3 domain | -1.3164 | 0.013241 | 462.22 | 486.49 | 331.41 | 391.52 |
| POFUT2 | protein O-fucosyltransferase 2 | 1.1251 | 0.035316 | 502.84 | 492.45 | 582.89 | 537.77 |
| SEC13 | SEC13 homolog (S. cerevisiae) | 1.1186 | 0.30148 | 603.09 | 699.08 | 678 | 778.05 |
| FNTB | farnesyltransferase. CAAX box. beta | -1.1175 | 0.11775 | 277.22 | 247.8 | 237.07 | 232.06 |
| MBOAT2 | membrane bound O-acyltransferase domain containing 2 | -1.4903 | 8.60E-06 | 458.92 | 517.95 | 325.39 | 328.91 |
| NMU | neuromedin U | 1.0635 | 0.43949 | 272.66 | 229.39 | 281.5 | 251.3 |
| PDCD10 | programmed cell death 10 | 1.1796 | 0.042303 | 1168.9 | 900.11 | 1180.4 | 1240.3 |
| C1orf112 | chromosome 1 open reading frame 112 | 1.1319 | 0.010305 | 282.94 | 266.9 | 326.61 | 296.23 |
| TUFT1 | tuftelin 1 | 1.6439 | 8.30E-08 | 1058.4 | 962.53 | 1634.7 | 1684.1 |
| MAN2B2 | mannosidase. alpha. class 2B. member 2 | -1.1989 | 0.005456 | 1540.8 | 1648.3 | 1311.4 | 1347.4 |
| MED10 | mediator complex subunit 10 | 1.0437 | 0.3605 | 1474.1 | 1398.1 | 1489.2 | 1507.5 |
| IL13RA1 | interleukin 13 receptor. alpha 1 | 1.0207 | 0.61421 | 612.4 | 613.55 | 636.03 | 615.47 |
| OCEL1 | occludin/ELL domain containing 1 | -1.1478 | 0.000868 | 669.13 | 667.93 | 571.25 | 593.9 |
| CRYAB | crystallin. alpha B | -1.1396 | 0.013414 | 229.6 | 227.8 | 194.24 | 207.34 |
| SCRG1 | scrapie responsive protein 1 | -1.0724 | 0.13527 | 144.29 | 151.76 | 141.78 | 134.31 |
| HNRPD | heterogeneous nuclear ribonucleoprotein D (AU-rich element RNA binding protein 1. 37kDa) | 1.1384 | 0.047282 | 2574.8 | 2789.1 | 2969 | 3134.5 |
| LZTR1 | leucine-zipper-like transcription regulator 1 | -1.0857 | 0.073049 | 985.44 | 930.17 | 905.17 | 859.15 |
| NAGK | N-acetylglucosamine kinase | -1.0648 | 0.2074 | 840.75 | 854.1 | 770.4 | 822.09 |
| TFPI | tissue factor pathway inhibitor (lipoprotein-associated coagulation inhibitor) | 1.1829 | 0.008182 | 3447.6 | 3405.5 | 4233 | 3881.3 |
| MGMT | O-6-methylguanine-DNA methyltransferase | 1.2259 | 0.033022 | 854.1 | 986.91 | 989.92 | 1279.6 |
| NFKBIA | nuclear factor of kappa light polypeptide gene enhancer in B-cells inhibitor. alpha | -1.9966 | 6.13E-08 | 4414.2 | 3870.5 | 2082.1 | 2058.4 |
| PACSIN2 | protein kinase C and casein kinase substrate in neurons 2 | -1.3828 | 4.65E-06 | 5295.2 | 4972.7 | 3763.1 | 3659.7 |
| SIPA1L2 | signal-induced proliferation-associated 1 like 2 | -1.0757 | 0.16236 | 601.11 | 556.39 | 525.24 | 550.26 |
| EDN1 | endothelin 1 | -1.042 | 0.17918 | 138.6 | 137 | 134.62 | 129.91 |
| KIAA1191 | KIAA1191 | 1.1339 | 0.055353 | 2025.1 | 2022.7 | 2405.1 | 2189.6 |
| SORT1 | sortilin 1 | 1.055 | 0.31026 | 280.3 | 304.12 | 315.31 | 300.89 |
| LOC203547 | hypothetical protein LOC203547 | -1.0677 | 0.19742 | 1923.9 | 1918.1 | 1835.9 | 1763.2 |
| DGKD | diacylglycerol kinase. delta 130kDa | 1.0998 | 0.012389 | 192.1 | 182.9 | 201.23 | 211.18 |
| FEM1C | fem-1 homolog c (C. elegans) | 1.0784 | 0.15116 | 665.5 | 613.77 | 702.27 | 676.47 |
| TBC1D22A | TBC1 domain family. member 22A | -1.0055 | 0.88033 | 685.85 | 697.67 | 691.65 | 684.22 |
| CHCHD7 | coiled-coil-helix-coiled-coil-helix domain containing 7 | -1.053 | 0.19732 | 611.54 | 591.88 | 574.06 | 568.63 |
| GBE1 | glucan (1.4-alpha-). branching enzyme 1 (glycogen branching enzyme. Andersen disease. glycogen storage disease type IV) | -1.1399 | 0.042611 | 672.17 | 636.03 | 576.26 | 570.94 |
| PGM3 | phosphoglucomutase 3 | 1.697 | 3.12E-08 | 636.27 | 626.33 | 1066.2 | 1076.3 |
| H2AFX | H2A histone family. member X | 1.27 | 0.00776 | 657.38 | 558.29 | 818.36 | 723.31 |
| MTMR9 | myotubularin related protein 9 | -1.0392 | 0.55248 | 622.61 | 595.98 | 599.55 | 573.1 |
| C2orf30 | chromosome 2 open reading frame 30 | 1.6018 | 0.000133 | 1342.2 | 1136.9 | 2054.2 | 1906 |
| TMEM165 | transmembrane protein 165 | 1.0252 | 0.58834 | 443 | 419.4 | 424.82 | 459.66 |
| ELL | elongation factor RNA polymerase II | -1.0414 | 0.51558 | 272.37 | 284.35 | 260.97 | 273.64 |
| CDKN2B | cyclin-dependent kinase inhibitor 2B (p15. inhibits CDK4) | -1.0221 | 0.66652 | 156.66 | 151.81 | 152.54 | 149.25 |
| GIPC1 | GIPC PDZ domain containing family. member 1 | -1.0889 | 0.044807 | 215.51 | 219.52 | 194.76 | 204.87 |
| ANGPT2 | angiopoietin 2 | 1.102 | 0.053238 | 202.69 | 176.7 | 204.76 | 212.42 |
| SLC26A3 | solute carrier family 26. member 3 | -1.36 | 3.98E-05 | 174.21 | 155.32 | 118.58 | 123.37 |
| CDKN1A | cyclin-dependent kinase inhibitor 1A (p21. Cip1) | -1.1217 | 0.021074 | 6536.1 | 6116.1 | 5553.9 | 5720.1 |
| EP400 | E1A binding protein p400 | 1.0566 | 0.18977 | 283.98 | 271.79 | 296.16 | 290.96 |
| ZYX | zyxin | 1.117 | 0.09992 | 936.38 | 915.15 | 1026.9 | 1041.3 |
| SLC3A2 | solute carrier family 3 (activators of dibasic and neutral amino acid transport). member 2 | 1.2255 | 0.000691 | 2867.7 | 2890.5 | 3492.3 | 3564.9 |
| HSP90AB1 | heat shock protein 90kDa alpha (cytosolic). class B member 1 | 1.3325 | 5.42E-05 | 3669.1 | 4078.9 | 4866 | 5460.5 |
| RPS29 | ribosomal protein S29 | 1.1532 | 0.022599 | 1795.7 | 1630.7 | 1957.1 | 1989.7 |
| ABCC4 | ATP-binding cassette. sub-family C (CFTR/MRP). member 4 | 1.0569 | 0.5444 | 3311.6 | 3209.6 | 3595.1 | 3302.7 |
| KIAA0513 | KIAA0513 | -1.0559 | 0.33072 | 264.85 | 249.79 | 246.14 | 241.06 |
| RWDD2A | RWD domain containing 2A | 1.1298 | 0.042875 | 1583 | 1376 | 1669.4 | 1665.4 |
| PCSK6 | proprotein convertase subtilisin/kexin type 6 | 1.0147 | 0.64451 | 154.75 | 148.72 | 156.1 | 151.81 |
| TMEM120A | transmembrane protein 120A | 1.009 | 0.89256 | 566.53 | 557.06 | 565.58 | 568.14 |
| SLC22A18 | solute carrier family 22 (organic cation transporter). member 18 | 1.0329 | 0.65261 | 386.24 | 385.53 | 401.36 | 395.8 |
| IFRD1 | interferon-related developmental regulator 1 | -1.0522 | 0.38444 | 1513.8 | 1250.7 | 1308.7 | 1306.6 |
| MYL5 | myosin. light chain 5. regulatory | 1.1563 | 0.003031 | 1042.4 | 1128.3 | 1204.7 | 1305.5 |
| BCAP31 | B-cell receptor-associated protein 31 | -1.0144 | 0.74582 | 3659.7 | 3903.7 | 3635.7 | 3818.4 |
| LIN7B | lin-7 homolog B (C. elegans) | -1.1805 | 0.001257 | 254.97 | 262.03 | 215.06 | 222.94 |
| C14orf132 | chromosome 14 open reading frame 132 | -1.4723 | 5.33E-05 | 387.49 | 338.6 | 256.33 | 236.12 |
| PARP4 | poly (ADP-ribose) polymerase family. member 4 | -1.0066 | 0.89437 | 2358.9 | 2191.7 | 2290.3 | 2227.9 |
| TBC1D19 | TBC1 domain family. member 19 | 1.0417 | 0.25117 | 258.33 | 263.62 | 276.61 | 267.17 |
| BSPRY | B-box and SPRY domain containing | -1.0053 | 0.85474 | 643.22 | 627.52 | 629.35 | 634.65 |
| SDC4 | syndecan 4 | -1.3495 | 0.000135 | 930.61 | 1048.4 | 683.06 | 784.35 |
| TAF5 | TAF5 RNA polymerase II. TATA box binding protein (TBP)-associated factor. 100kDa | 1.5247 | 1.74E-07 | 232.31 | 240.93 | 365.78 | 355.7 |
| SPSB3 | splA/ryanodine receptor domain and SOCS box containing 3 | -1.0031 | 0.93317 | 832.37 | 846.64 | 822.87 | 851.05 |
| IQGAP1 | IQ motif containing GTPase activating protein 1 | -1.1186 | 0.27421 | 241.65 | 250.52 | 207.63 | 233.02 |
| SAR1B | SAR1 gene homolog B (S. cerevisiae) | -1.0816 | 0.26744 | 3202.7 | 3058 | 2950.6 | 2837.2 |
| SLC6A3 | solute carrier family 6 (neurotransmitter transporter. dopamine). member 3 | -1.0793 | 0.018651 | 136.73 | 135.48 | 124.38 | 127.86 |
| HMGCS2 | 3-hydroxy-3-methylglutaryl-Coenzyme A synthase 2 (mitochondrial) | 1.1166 | 0.016211 | 663.97 | 679.82 | 755.93 | 744.52 |
| CDR2L | cerebellar degeneration-related protein 2-like | -1.1148 | 0.016177 | 260.78 | 249.51 | 229.2 | 228.45 |
| IMPDH2 | IMP (inosine monophosphate) dehydrogenase 2 | 1.3205 | 3.40E-05 | 4704.8 | 4926.2 | 6451.5 | 6263.9 |
| ARRDC1 | arrestin domain containing 1 | -1.1934 | 0.004163 | 318.62 | 303.3 | 258.67 | 262.33 |
| PRMT1 | protein arginine methyltransferase 1 | 1.2169 | 5.53E-05 | 2660 | 2642 | 3209.6 | 3242.6 |
| PEA15 | phosphoprotein enriched in astrocytes 15 | -1.1867 | 0.004033 | 1785.8 | 1618.5 | 1390.7 | 1475.9 |
| MOSC1 | MOCO sulphurase C-terminal domain containing 1 | 1.1004 | 0.10339 | 1848.1 | 1763.2 | 2076.7 | 1899.8 |
| ZXDB | zinc finger. X-linked. duplicated B | 1.0422 | 0.62808 | 428.18 | 397.97 | 433.37 | 427.11 |
| GDPD5 | glycerophosphodiester phosphodiesterase domain containing 5 | -1.0483 | 0.22266 | 154.05 | 152.96 | 150.42 | 142.54 |
| P4HA2 | procollagen-proline. 2-oxoglutarate 4-dioxygenase (proline 4-hydroxylase). alpha polypeptide II | -1.1975 | 0.001286 | 519.11 | 484.31 | 420.04 | 417.42 |
| SLC2A5 | solute carrier family 2 (facilitated glucose/fructose transporter). member 5 | -1.0346 | 0.37362 | 126.31 | 120.2 | 118.59 | 119.62 |
| C14orf147 | chromosome 14 open reading frame 147 | 1.1891 | 0.001586 | 646.21 | 582.52 | 725.15 | 734.01 |
| RELN | reelin | 1.3377 | 1.83E-05 | 284.35 | 279.16 | 369.23 | 384.7 |
| ITSN1 | intersectin 1 (SH3 domain protein) | -1.0481 | 0.29361 | 523.72 | 566.32 | 531.44 | 508.07 |
| PLXNA1 | plexin A1 | -1.0981 | 0.07384 | 217.07 | 202.58 | 182.52 | 199.8 |
| PIGG | phosphatidylinositol glycan anchor biosynthesis. class G | -1.0096 | 0.88136 | 287.67 | 292 | 297.13 | 277.36 |
| SERP1 | stress-associated endoplasmic reticulum protein 1 | 1.5704 | 5.93E-05 | 1012.2 | 1148.1 | 1614.6 | 1775.1 |
| ZNF541 | zinc finger protein 541 | -1.1013 | 0.008 | 161.75 | 157.27 | 146.76 | 142.92 |
| TST | thiosulfate sulfurtransferase (rhodanese) | -1.1525 | 0.005368 | 3339.3 | 3378 | 2894.1 | 2934.3 |
| FADS3 | fatty acid desaturase 3 | 1.1465 | 0.061472 | 582.33 | 566.73 | 651.17 | 666.15 |
| SYNCRIP | synaptotagmin binding. cytoplasmic RNA interacting protein | 1.2683 | 0.001085 | 1191 | 1317.8 | 1517.8 | 1663.3 |
| CPEB4 | cytoplasmic polyadenylation element binding protein 4 | -1.0372 | 0.57702 | 266.48 | 264.12 | 256.18 | 255.37 |
| SLC1A3 | solute carrier family 1 (glial high affinity glutamate transporter). member 3 | -1.0142 | 0.69689 | 127.6 | 125.81 | 124.62 | 125.23 |
| AHSA2 | AHA1. activator of heat shock 90kDa protein ATPase homolog 2 (yeast) | -1.0574 | 0.56578 | 526.85 | 457.86 | 459.94 | 469.07 |
| SLC22A16 | solute carrier family 22 (organic cation transporter). member 16 | -1.0192 | 0.55368 | 142.95 | 142.98 | 138.3 | 142.27 |
| IMPDH1 | IMP (inosine monophosphate) dehydrogenase 1 | -1.1421 | 0.015976 | 1317.1 | 1259 | 1161.2 | 1094.9 |
| C9orf46 | chromosome 9 open reading frame 46 | 1.3261 | 0.000953 | 1914.6 | 2332.1 | 2857.8 | 2747.7 |
| CALD1 | caldesmon 1 | -1.0244 | 0.79577 | 193.06 | 211.52 | 188.42 | 206.51 |
| EBPL | emopamil binding protein-like | 1.1349 | 0.08505 | 2304.1 | 2425.7 | 2831.9 | 2542.1 |
| GFPT1 | glutamine-fructose-6-phosphate transaminase 1 | 1.3688 | 0.00017 | 2667.7 | 2334.2 | 3554.5 | 3282.5 |
| ZFAND2A | zinc finger. AN1-type domain 2A | 1.1913 | 0.011725 | 1842.2 | 1679.1 | 2016.3 | 2177.4 |
| PPP1R13B | protein phosphatase 1. regulatory (inhibitor) subunit 13B | -1.2167 | 0.006659 | 631.86 | 597.66 | 511.74 | 498.47 |
| MKNK1 | MAP kinase interacting serine/threonine kinase 1 | -1.2085 | 0.000542 | 473.48 | 443 | 375.45 | 382.52 |
| NRBP2 | nuclear receptor binding protein 2 | 1.0685 | 0.33245 | 291.61 | 232.82 | 279.87 | 276.94 |
| GALNTL4 | UDP-N-acetyl-alpha-D-galactosamine:polypeptide N-acetylgalactosaminyltransferase-like 4 | -1.5804 | 5.21E-06 | 324.28 | 319.45 | 201.29 | 206.06 |
| NAP1L4 | nucleosome assembly protein 1-like 4 | 1.08 | 0.12433 | 1876.3 | 1805.7 | 2091.9 | 1889.2 |
| TP53I3 | tumor protein p53 inducible protein 3 | -1.414 | 1.46E-05 | 251.38 | 230.03 | 166.71 | 173.5 |
| STXBP5 | syntaxin binding protein 5 (tomosyn) | -1.1088 | 0.029981 | 337.55 | 314.28 | 298.34 | 289.24 |
| TGFBR3 | transforming growth factor. beta receptor III | -1.1993 | 0.011423 | 225.06 | 222.37 | 182.68 | 190.47 |
| NCAM2 | neural cell adhesion molecule 2 | 1.2708 | 0.001843 | 3936 | 3927.2 | 5067.3 | 4926.2 |
| TPM1 | tropomyosin 1 (alpha) | -1.635 | 1.41E-07 | 1697.1 | 1739.6 | 977.39 | 1129.9 |
| YWHAQ | tyrosine 3-monooxygenase/tryptophan 5-monooxygenase activation protein. theta polypeptide | -1.1273 | 0.078764 | 8996.7 | 7958.2 | 7702.9 | 7314.3 |
| TNFRSF10B | tumor necrosis factor receptor superfamily. member 10b | -1.0351 | 0.56477 | 2762.6 | 2608 | 2687.4 | 2502.3 |
| PTTG1IP | pituitary tumor-transforming 1 interacting protein | -1.0817 | 0.052256 | 1067.4 | 1152.4 | 1052 | 999.32 |
| MYH13 | myosin. heavy chain 13. skeletal muscle | -1.0318 | 0.30677 | 133.99 | 127.99 | 128.11 | 125.75 |
| ATP1A2 | ATPase. Na+/K+ transporting. alpha 2 (+) polypeptide | -1.1225 | 0.003367 | 145.29 | 140.93 | 126.3 | 128.67 |
| ATG16L1 | ATG16 autophagy related 16-like 1 (S. cerevisiae) | -1.0511 | 0.54521 | 216.1 | 237.26 | 201.93 | 229.82 |
| NAT2 | N-acetyltransferase 2 (arylamine N-acetyltransferase) | -1.1352 | 0.16025 | 168.23 | 172.84 | 149.85 | 150.58 |
| CETN2 | centrin. EF-hand protein. 2 | 1.039 | 0.24044 | 3492.3 | 3574.3 | 3574.3 | 3769.7 |
| IKBKG | inhibitor of kappa light polypeptide gene enhancer in B-cells. kinase gamma | 1.039 | 0.43912 | 1165.7 | 1039.9 | 1171.1 | 1117.4 |
| ATP1B1 | ATPase. Na+/K+ transporting. beta 1 polypeptide | -1.0466 | 0.5078 | 1222.8 | 1276.7 | 1131.3 | 1259.7 |
| DHRS13 | dehydrogenase/reductase (SDR family) member 13 | 1.13 | 0.039407 | 568.63 | 626.06 | 665.94 | 682.58 |
| IL20RB | interleukin 20 receptor beta | -1.1032 | 0.002851 | 151.42 | 145.96 | 134.97 | 134.55 |
| MYH9 | myosin. heavy chain 9. non-muscle | -1.0646 | 0.22999 | 5706.3 | 5185.2 | 5268.6 | 4955.4 |
| RBMX | RNA binding motif protein. X-linked | 1.0121 | 0.80172 | 1398.8 | 1313.7 | 1340.3 | 1404.5 |
| PBEF1 | pre-B-cell colony enhancing factor 1 | -1.2711 | 9.93E-06 | 660.05 | 647.45 | 532.78 | 496.44 |
| PPA2 | pyrophosphatase (inorganic) 2 | 1.0793 | 0.36196 | 1438.4 | 1643.9 | 1580.5 | 1742.9 |
| SPON2 | spondin 2. extracellular matrix protein | 1.0302 | 0.6903 | 697.23 | 735.43 | 744.33 | 731.06 |
| C9orf140 | chromosome 9 open reading frame 140 | -1.0813 | 0.23708 | 387.17 | 379.71 | 380.76 | 330.23 |
| TMSB10 | thymosin. beta 10 | -1.1937 | 0.001535 | 3456.2 | 3265.3 | 2667.7 | 2969 |
| MGC14376 | hypothetical protein MGC14376 | 1.6511 | 4.91E-05 | 674.57 | 480.73 | 918.43 | 962.53 |
| SLC22A5 | solute carrier family 22 (organic cation transporter). member 5 | -1.1278 | 0.001791 | 503.02 | 516.53 | 467.87 | 436.59 |
| NPC1 | Niemann-Pick disease. type C1 | -1.2773 | 0.003146 | 784.35 | 704.63 | 578.54 | 585.53 |
| PTPLA | protein tyrosine phosphatase-like (proline instead of catalytic arginine). member A | -1.0419 | 0.2472 | 150.47 | 143.09 | 136.81 | 144.98 |
| FIG4 | FIG4 homolog (S. cerevisiae) | -1.1909 | 0.004955 | 524.77 | 458.81 | 425.16 | 399.27 |
| C21orf34 | chromosome 21 open reading frame 34 | 1.3452 | 0.000271 | 1591.8 | 1627.8 | 2071.8 | 2263.1 |
| FNBP1L | formin binding protein 1-like | -1.5018 | 8.36E-05 | 1327.8 | 1150.5 | 786.08 | 861.69 |
| CREG1 | cellular repressor of E1A-stimulated genes 1 | -1.0043 | 0.9525 | 1376 | 1498.3 | 1482.1 | 1379.3 |
| IGF2BP2 | insulin-like growth factor 2 mRNA binding protein 2 | -1.0983 | 0.052877 | 151.76 | 147.69 | 134.52 | 138.12 |
| PIGH | phosphatidylinositol glycan anchor biosynthesis. class H | 1.0647 | 0.32285 | 355.22 | 399.09 | 382.78 | 419.79 |
| ABCA4 | ATP-binding cassette. sub-family A (ABC1). member 4 | 1.0783 | 0.080455 | 156 | 137.49 | 156.71 | 159.14 |
| PNLIPRP3 | pancreatic lipase-related protein 3 | -1.1504 | 0.022482 | 153.08 | 140.14 | 128.33 | 126.31 |
| TPD52L1 | tumor protein D52-like 1 | -1.2422 | 0.013129 | 2657.7 | 2438.6 | 2171 | 1934.8 |
| TGIF2 | TGFB-induced factor homeobox 2 | 1.2007 | 0.000765 | 271.43 | 295.87 | 337.16 | 343.42 |
| CENPN | centromere protein N | 1.0108 | 0.80113 | 5390.1 | 5325 | 5479.7 | 5351.5 |
| PPP1R15A | protein phosphatase 1. regulatory (inhibitor) subunit 15A | 1.2694 | 0.000592 | 364.48 | 349.04 | 433.09 | 473.3 |
| PRODH | proline dehydrogenase (oxidase) 1 | 1.0054 | 0.86474 | 123.46 | 127.11 | 127.32 | 124.6 |
| SLC9A1 | solute carrier family 9 (sodium/hydrogen exchanger). member 1 (antiporter. Na+/H+. amiloride sensitive) | 1.047 | 0.3352 | 2861.7 | 2937.9 | 2995.3 | 3077 |
| C6orf108 | chromosome 6 open reading frame 108 | 1.2416 | 0.01628 | 2945.9 | 3539.8 | 4084.1 | 3936 |
| C6orf81 | chromosome 6 open reading frame 81 | -1.2103 | 0.000225 | 175.67 | 170.28 | 142.95 | 142.85 |
| WDR41 | WD repeat domain 41 | -1.0826 | 0.17468 | 638.46 | 565.33 | 578.96 | 531.94 |
| D4S234E | DNA segment on chromosome 4 (unique) 234 expressed sequence | -1.3033 | 8.76E-05 | 207.99 | 229.36 | 162.22 | 173.13 |
| FBXL17 | F-box and leucine-rich repeat protein 17 | 1.2661 | 0.010838 | 477.9 | 507.69 | 646.21 | 601.85 |
| FOXC1 | forkhead box C1 | -1.0284 | 0.40004 | 156.07 | 141.43 | 144.14 | 144.8 |
| RAB32 | RAB32. member RAS oncogene family | -1.1483 | 0.049222 | 341.84 | 322.93 | 275.06 | 304.37 |
| GPR1 | G protein-coupled receptor 1 | -1.1966 | 0.001336 | 206.33 | 206.33 | 180.7 | 164.54 |
| C19orf48 | chromosome 19 open reading frame 48 | 1.0206 | 0.63664 | 4491.6 | 4657 | 4802.8 | 4536.8 |
| HSD17B8 | hydroxysteroid (17-beta) dehydrogenase 8 | 1.1919 | 0.004533 | 1479.8 | 1680.1 | 1874.8 | 1883.9 |
| PRUNE2 | prune homolog 2 (Drosophila) | 1.0952 | 0.10254 | 2851.4 | 2570.1 | 3106.6 | 2829.3 |
| LOC388610 | hypothetical LOC388610 | -1.0552 | 0.21615 | 351.25 | 370.82 | 348.33 | 335.82 |
| MSX1 | msh homeobox 1 | -1.1774 | 0.046064 | 1487.3 | 1399.7 | 1285.5 | 1168.1 |
| ATF3 | activating transcription factor 3 | 1.7643 | 3.35E-06 | 3260.6 | 2621.6 | 5460.5 | 4873 |
| SCARB1 | scavenger receptor class B. member 1 | 1.1441 | 0.012872 | 2730.3 | 2773.1 | 3323 | 2982.6 |
| TRIOBP | TRIO and F-actin binding protein | -1.0112 | 0.74721 | 445.32 | 450.29 | 439.96 | 445.77 |
| CLDN3 | claudin 3 | -1.033 | 0.53699 | 919.42 | 958.37 | 910.85 | 906.57 |
| DECR1 | 2.4-dienoyl CoA reductase 1. mitochondrial | -1.3469 | 0.000698 | 3378 | 3769.7 | 2526.6 | 2778.3 |
| SLC45A3 | solute carrier family 45. member 3 | 1.3835 | 7.11E-05 | 952 | 976.51 | 1296.7 | 1372.2 |
| EPHX2 | epoxide hydrolase 2. cytoplasmic | -1.0029 | 0.9491 | 711.36 | 779.52 | 741.43 | 743.57 |
| TMTC4 | transmembrane and tetratricopeptide repeat containing 4 | 1.091 | 0.10236 | 574.58 | 571.44 | 630.15 | 620.18 |
| ELF1 | E74-like factor 1 (ets domain transcription factor) | -1.3022 | 2.44E-05 | 1475.9 | 1385.8 | 1129.4 | 1067.9 |
| MT1X | metallothionein 1X | 1.9991 | 1.46E-06 | 3802.6 | 3256.5 | 7160.8 | 6910.8 |
| SCGN | secretagogin. EF-hand calcium binding protein | -1.0823 | 0.046979 | 143.25 | 135.8 | 132.05 | 125.77 |
| GLIPR1 | GLI pathogenesis-related 1 (glioma) | -1.0254 | 0.42448 | 140.83 | 144.63 | 138.78 | 139.58 |
| NUDT5 | nudix (nucleoside diphosphate linked moiety X)-type motif 5 | -1.2539 | 0.001275 | 2206 | 2398.9 | 1769.9 | 1901.6 |
| SELM | selenoprotein M | -1.0249 | 0.72131 | 1739.6 | 1944.4 | 1870.4 | 1721.5 |
| NME3 | non-metastatic cells 3. protein expressed in | 1.0477 | 0.49377 | 1576.3 | 1669.4 | 1758.9 | 1642.3 |
| FSTL3 | follistatin-like 3 (secreted glycoprotein) | -1.0379 | 0.51331 | 176.02 | 181.82 | 174.24 | 170.51 |
| ACPP | acid phosphatase. prostate | -1.0017 | 0.97722 | 548.98 | 625.1 | 603.52 | 566.73 |
| PROS1 | protein S (alpha) | -1.282 | 7.13E-05 | 281.01 | 264.47 | 214.35 | 210.95 |
| TGM1 | transglutaminase 1 (K polypeptide epidermal type I. protein-glutamine-gamma-glutamyltransferase) | -1.0415 | 0.42631 | 156.95 | 157.88 | 147.98 | 154.38 |
| ACTG2 | actin. gamma 2. smooth muscle. enteric | -1.1393 | 0.001359 | 153.07 | 155.15 | 135.03 | 135.49 |
| ACY1 | aminoacylase 1 | 1.0411 | 0.36863 | 1780.5 | 1934.8 | 2002.8 | 1864.3 |
| C1orf80 | chromosome 1 open reading frame 80 | 1.2659 | 0.003574 | 339.31 | 422.4 | 477.61 | 480.85 |
| CALCB | calcitonin-related polypeptide. beta | -1.0114 | 0.70086 | 141.34 | 145.1 | 144.45 | 138.8 |
| AHNAK | AHNAK nucleoprotein | -2.0594 | 3.64E-07 | 709.43 | 604.22 | 323.31 | 312.61 |
| ACSM3 | acyl-CoA synthetase medium-chain family member 3 | 1.0843 | 0.30186 | 704.32 | 644.41 | 757.6 | 704.32 |
| TPM4 | tropomyosin 4 | 1.108 | 0.07896 | 217.76 | 215.47 | 229.67 | 250.8 |
| C6orf32 | chromosome 6 open reading frame 32 | -1.7346 | 1.99E-08 | 548.61 | 594.9 | 330.92 | 327.79 |
| CKMT1A | creatine kinase. mitochondrial 1A | -1.0634 | 0.09296 | 453.08 | 487.61 | 438.61 | 445.45 |
| SRM | spermidine synthase | 1.2131 | 0.021912 | 1279.6 | 1415.2 | 1741 | 1530.8 |
| DNM1L | dynamin 1-like | -1.3004 | 0.003629 | 635.71 | 589.57 | 479.9 | 461.85 |
| PRKCD | protein kinase C. delta | -1.8087 | 8.55E-10 | 2023.9 | 1864.3 | 1049.5 | 1098.9 |
| LEO1 | Leo1. Paf1/RNA polymerase II complex component. homolog (S. cerevisiae) | 1.0454 | 0.47814 | 621.73 | 603.09 | 646.77 | 633.62 |
| ACTN1 | actinin. alpha 1 | -1.3341 | 0.004186 | 1701.2 | 1784.6 | 1244.1 | 1371.1 |
| TFDP1 | transcription factor Dp-1 | 1.0506 | 0.2536 | 732.18 | 781.38 | 758.71 | 832.37 |
| ARG2 | arginase. type II | 1.4753 | 4.08E-07 | 386.86 | 409.22 | 598.77 | 575.43 |
| PFKM | phosphofructokinase. muscle | 1.0759 | 0.093213 | 844.35 | 888.67 | 923.28 | 940.77 |
| MCCC2 | methylcrotonoyl-Coenzyme A carboxylase 2 (beta) | 1.0977 | 0.081279 | 406.11 | 390.81 | 454.55 | 420.73 |
| FLJ40852 | hypothetical protein FLJ40852 | -1.0348 | 0.39988 | 222.65 | 216.35 | 216.62 | 207.67 |
| AUH | AU RNA binding protein/enoyl-Coenzyme A hydratase | 1.2241 | 0.00017 | 991.86 | 925.68 | 1203.3 | 1143.4 |
| IYD | iodotyrosine deiodinase | -1.2093 | 8.76E-05 | 205.47 | 211.45 | 173.46 | 171.29 |
| CPNE3 | copine III | 1.1594 | 0.016819 | 6484.7 | 6263.9 | 7480.8 | 7298.6 |
| DERA | 2-deoxyribose-5-phosphate aldolase homolog (C. elegans) | -1.452 | 2.98E-07 | 1955.7 | 2002.8 | 1349.5 | 1376.7 |
| TMCO3 | transmembrane and coiled-coil domains 3 | 1.2348 | 0.000138 | 6381.3 | 5862.6 | 7388 | 7720.4 |
| RDH11 | retinol dehydrogenase 11 (all-trans/9-cis/11-cis) | 1.0163 | 0.83274 | 6149 | 6606.4 | 5911.7 | 7096.8 |
| ATIC | 5-aminoimidazole-4-carboxamide ribonucleotide formyltransferase/IMP cyclohydrolase | 1.0727 | 0.10332 | 7587.7 | 7229.6 | 7847.4 | 8043.4 |
| WDR1 | WD repeat domain 1 | -1.1119 | 0.046207 | 3772.8 | 3772.8 | 3282.5 | 3507.2 |
| CPNE1 | copine I | 1.119 | 0.044787 | 800.26 | 746.3 | 859.94 | 869.7 |
| CXCR7 | chemokine (C-X-C motif) receptor 7 | 1.0284 | 0.62884 | 2111.9 | 2276 | 2154.9 | 2358.9 |
| DCUN1D3 | DCN1. defective in cullin neddylation 1. domain containing 3 (S. cerevisiae) | -1.1277 | 0.050065 | 484.46 | 432.43 | 428.62 | 384.37 |
| GAMT | guanidinoacetate N-methyltransferase | -1.004 | 0.93341 | 2416.8 | 2436.9 | 2521.8 | 2317 |
| PRR7 | proline rich 7 (synaptic) | -1.0563 | 0.24918 | 420.88 | 429.94 | 416.82 | 389.12 |
| SOX8 | SRY (sex determining region Y)-box 8 | -1.1555 | 0.019304 | 449.47 | 459.94 | 407.28 | 380.18 |
| CYB5A | cytochrome b5 type A (microsomal) | -1.0662 | 0.15333 | 7563.8 | 8677 | 7542.6 | 7654.4 |
| MYC | v-myc myelocytomatosis viral oncogene homolog (avian) | -1.0265 | 0.64896 | 2215.9 | 2510 | 2125.4 | 2483.8 |
| MT1G | metallothionein 1G | 2.394 | 2.10E-08 | 445.45 | 387.49 | 968.24 | 1021.7 |
| ACTA2 | actin. alpha 2. smooth muscle. aorta | -1.8575 | 3.03E-07 | 708.14 | 609.82 | 354.88 | 352.67 |
| C5orf32 | chromosome 5 open reading frame 32 | -1.1425 | 0.056045 | 593.9 | 601.32 | 539.54 | 507.06 |
| 3-Sep | septin 3 | -1.0033 | 0.94317 | 555.12 | 547.51 | 532.37 | 567.17 |
| TNFRSF10D | tumor necrosis factor receptor superfamily. member 10d. decoy with truncated death domain | -1.4295 | 3.40E-05 | 260.72 | 267.85 | 186.49 | 183.25 |
| TRIB3 | tribbles homolog 3 (Drosophila) | 1.2094 | 0.013022 | 8151.8 | 7112.7 | 9671.2 | 8769.2 |
| E2F5 | E2F transcription factor 5. p130-binding | 1.0848 | 0.097513 | 370.69 | 369.5 | 404.19 | 398.75 |
| CABC1 | chaperone. ABC1 activity of bc1 complex homolog (S. pombe) | -1.0524 | 0.31837 | 903.64 | 1058.4 | 950.57 | 908.44 |
| FOLH1 | folate hydrolase (prostate-specific membrane antigen) 1 | 1.0863 | 0.40636 | 1049.5 | 1211.6 | 1116 | 1344.4 |
| ZNF323 | zinc finger protein 323 | 1.9535 | 1.03E-08 | 307.55 | 295.29 | 580.18 | 597.36 |
| THOP1 | thimet oligopeptidase 1 | 1.1462 | 0.021918 | 891.59 | 851.57 | 978.38 | 1019.5 |
| ZNF511 | zinc finger protein 511 | -1.0988 | 0.054962 | 2554.8 | 2498.9 | 2326.8 | 2272.4 |
| CGNL1 | cingulin-like 1 | -1.071 | 0.10162 | 623.82 | 601.57 | 585.84 | 558.49 |
| HMOX1 | heme oxygenase (decycling) 1 | 1.5905 | 7.03E-07 | 197.85 | 177.46 | 280.91 | 316.2 |
| BOP1 | block of proliferation 1 | -1.1646 | 0.019724 | 1725 | 1826.9 | 1519.9 | 1528.9 |
| CXCL16 | chemokine (C-X-C motif) ligand 16 | 1.0593 | 0.23691 | 1070.4 | 1115.2 | 1189.3 | 1126.3 |
| LRRN1 | leucine rich repeat neuronal 1 | -1.1022 | 0.13434 | 343.58 | 360.12 | 333.16 | 305.71 |
| SDC1 | syndecan 1 | -1.779 | 8.24E-10 | 498.83 | 469.7 | 268.8 | 275.4 |
| C4orf18 | chromosome 4 open reading frame 18 | -1.0433 | 0.61505 | 1293.1 | 1519.9 | 1369.2 | 1318.8 |
| DKC1 | dyskeratosis congenita 1. dyskerin | 1.0964 | 0.080959 | 2435 | 2364.5 | 2516.1 | 2750.9 |
| HOMER2 | homer homolog 2 (Drosophila) | -1.6828 | 8.63E-08 | 1269.5 | 1246.2 | 767.57 | 727.79 |
| VARS | valyl-tRNA synthetase | -1.0323 | 0.42316 | 2064.8 | 2289.4 | 2141.2 | 2071.8 |
| CACYBP | calcyclin binding protein | 1.1051 | 0.04795 | 778.05 | 709.78 | 829.16 | 813.41 |
| JAG2 | jagged 2 | -1.0511 | 0.32045 | 1063.8 | 1054.5 | 1013.5 | 1001.8 |
| EFNB2 | ephrin-B2 | 1.0057 | 0.94501 | 1100.2 | 952.95 | 1000.5 | 1059.8 |
| WDR57 | WD repeat domain 57 (U5 snRNP specific) | 1.0742 | 0.18452 | 931.65 | 994.46 | 1082.1 | 988.01 |
| CMTM7 | CKLF-like MARVEL transmembrane domain containing 7 | -1.1411 | 0.009759 | 560.3 | 498.58 | 468.57 | 457.86 |
| CCND1 | cyclin D1 | -1.0704 | 0.17513 | 3874.9 | 3728.9 | 3543.1 | 3559.1 |
| APOD | apolipoprotein D | -1.713 | 8.52E-07 | 636.5 | 619.78 | 352.67 | 381.21 |
| RPL13A | ribosomal protein L13a | -1.0753 | 0.33193 | 12700 | 14770 | 12595 | 12881 |
| MRPL24 | mitochondrial ribosomal protein L24 | -1.0072 | 0.88068 | 4265.3 | 4636.9 | 4628.4 | 4212.3 |
| CDK4 | cyclin-dependent kinase 4 | 1.0849 | 0.092842 | 1979.5 | 2070.6 | 2066.7 | 2334.2 |
| RRAS | related RAS viral (r-ras) oncogene homolog | -1.515 | 4.09E-07 | 816.98 | 856.99 | 550.85 | 553.76 |
| TNFRSF12A | tumor necrosis factor receptor superfamily. member 12A | -1.1869 | 0.10034 | 333.16 | 323.17 | 258.26 | 295.94 |
| C1orf128 | chromosome 1 open reading frame 128 | 1.112 | 0.008414 | 734.01 | 764.01 | 816.09 | 849.65 |
| VWF | von Willebrand factor | -1.1127 | 0.085204 | 338.26 | 346.75 | 330.1 | 286.98 |
| AKR1A1 | aldo-keto reductase family 1. member A1 (aldehyde reductase) | 1.0508 | 0.32724 | 3452.1 | 3669.1 | 3688.2 | 3791.9 |
| ATP5D | ATP synthase. H+ transporting. mitochondrial F1 complex. delta subunit | -1.041 | 0.29159 | 5797.2 | 5694.1 | 5543.5 | 5494.8 |
| ARHGEF2 | rho/rac guanine nucleotide exchange factor (GEF) 2 | -1.0059 | 0.88812 | 2027 | 1849.8 | 1958.8 | 1891.8 |
| SRXN1 | sulfiredoxin 1 homolog (S. cerevisiae) | 1.5834 | 8.35E-06 | 971.17 | 963.36 | 1576.3 | 1488.2 |
| D15Wsu75e | DNA segment. Chr 15. Wayne State University 75. expressed | 1.1429 | 0.036412 | 1039.6 | 968.24 | 1189.7 | 1105.2 |
| NOL5A | nucleolar protein 5A (56kDa with KKE/D repeat) | -1.1779 | 0.073977 | 2978.6 | 3339.3 | 2557.9 | 2802.6 |
| ALS2CR13 | amyotrophic lateral sclerosis 2 (juvenile) chromosome region. candidate 13 | 1.0618 | 0.36091 | 1596.6 | 1604.6 | 1766.9 | 1634.7 |
| SERPINB6 | serpin peptidase inhibitor. clade B (ovalbumin). member 6 | -1.1985 | 0.001616 | 7033.3 | 7742.2 | 6097.4 | 6217.7 |
| PDCD2L | programmed cell death 2-like | -1.0736 | 0.23709 | 729.98 | 735.11 | 686.7 | 678 |
| TNFRSF14 | tumor necrosis factor receptor superfamily. member 14 (herpesvirus entry mediator) | -1.0136 | 0.83951 | 909.77 | 892.75 | 864.35 | 914.69 |
| CA12 | carbonic anhydrase XII | -2.0362 | 6.91E-09 | 423.1 | 376.49 | 204.06 | 188.28 |
| SLC7A5 | solute carrier family 7 (cationic amino acid transporter. y+ system). member 5 | 1.084 | 0.083454 | 789.66 | 811.2 | 863.32 | 871.83 |
| CTXN1 | cortexin 1 | -1.0927 | 0.15525 | 2133.4 | 2051.6 | 1848.1 | 1983.6 |
| CDCA7 | cell division cycle associated 7 | -1.0478 | 0.4564 | 331.33 | 337.64 | 317.19 | 321.22 |
| GEMIN4 | gem (nuclear organelle) associated protein 4 | -1.1153 | 0.035207 | 960.37 | 999.32 | 902.71 | 854.67 |
| PUS7 | pseudouridylate synthase 7 homolog (S. cerevisiae) | -1.0796 | 0.18187 | 831.09 | 748.2 | 753.36 | 708.14 |
| ALCAM | activated leukocyte cell adhesion molecule | -1.5099 | 9.05E-06 | 1460.8 | 1609.4 | 952.95 | 1082.1 |
| SARS2 | seryl-tRNA synthetase 2. mitochondrial | -1.0043 | 0.92768 | 1161.2 | 1281.8 | 1215.6 | 1214 |
| RAB26 | RAB26. member RAS oncogene family | -1.1608 | 0.030798 | 1138 | 1066.2 | 896.3 | 1004.6 |
| GCLM | glutamate-cysteine ligase. modifier subunit | 2.0407 | 1.19E-06 | 1128.3 | 939.83 | 2298 | 1921.7 |
| TUBB3 | tubulin. beta 3 | 1.7855 | 5.67E-06 | 358.24 | 318.58 | 591.38 | 615.24 |
| ATAD4 | ATPase family. AAA domain containing 4 | -1.7737 | 1.48E-07 | 1116.3 | 1173.2 | 625.51 | 665.5 |
| CCDC58 | coiled-coil domain containing 58 | -1.0803 | 0.42274 | 823.67 | 912.44 | 748.82 | 859.94 |
| ID2 | inhibitor of DNA binding 2. dominant negative helix-loop-helix protein | -1.7117 | 1.43E-07 | 430.44 | 382.78 | 250.89 | 224.14 |
| ANK3 | ankyrin 3. node of Ranvier (ankyrin G) | -1.264 | 0.000113 | 2823.2 | 2640.3 | 2133.4 | 2186.8 |
| COQ2 | coenzyme Q2 homolog. prenyltransferase (yeast) | -1.0404 | 0.36886 | 1307.4 | 1295.8 | 1275.3 | 1227.2 |
| MRPL12 | mitochondrial ribosomal protein L12 | -1.0664 | 0.28136 | 735.66 | 771.2 | 723.63 | 689.47 |
| OPTN | optineurin | -1.1798 | 0.000823 | 202.13 | 185.13 | 162.76 | 165.15 |
| SAMD13 | sterile alpha motif domain containing 13 | -1.0641 | 0.09824 | 308.08 | 298.58 | 291.61 | 278.56 |
| SH3BP4 | SH3-domain binding protein 4 | -1.1023 | 0.037108 | 1216.1 | 1235.7 | 1116.7 | 1107.4 |
| FKBP4 | FK506 binding protein 4. 59kDa | -1.0678 | 0.44729 | 1561.8 | 1972 | 1581.7 | 1707.9 |
| CAPN13 | calpain 13 | -1.8598 | 1.79E-07 | 582.73 | 500.24 | 296.63 | 284.1 |
| FAM60A | family with sequence similarity 60. member A | -1.0862 | 0.15694 | 2066.7 | 1870.4 | 1818.2 | 1802 |
| LYSMD2 | LysM. putative peptidoglycan-binding. domain containing 2 | 1.0844 | 0.049235 | 769.59 | 714.05 | 795.8 | 811.98 |
| POLD2 | polymerase (DNA directed). delta 2. regulatory subunit 50kDa | -1.0666 | 0.17068 | 11579 | 11202 | 10502 | 10855 |
| MGC23985 | similar to AVLV472 | -1.2873 | 5.22E-06 | 184.58 | 178.52 | 136.08 | 146.12 |
| ZMIZ1 | zinc finger. MIZ-type containing 1 | -1.2489 | 0.010143 | 1220.4 | 1059.8 | 956.5 | 866.89 |
| MAFB | v-maf musculoaponeurotic fibrosarcoma oncogene homolog B (avian) | -2.0102 | 6.23E-10 | 1849.8 | 1705.6 | 866.89 | 900.61 |
| PSME2 | proteasome (prosome. macropain) activator subunit 2 (PA28 beta) | -1.0958 | 0.07783 | 2010.4 | 1962.8 | 1922.7 | 1709.3 |
| GBL | G protein beta subunit-like | -1.0652 | 0.22687 | 1505.1 | 1496.1 | 1448.4 | 1370 |
| PRKD1 | protein kinase D1 | 1.0069 | 0.88472 | 2675.3 | 2446.3 | 2605.6 | 2546.3 |
| C18orf56 | chromosome 18 open reading frame 56 | -1.2636 | 0.016287 | 1242.7 | 1023.4 | 940.77 | 846.64 |
| PAICS | phosphoribosylaminoimidazole carboxylase. phosphoribosylaminoimidazole succinocarboxamide synthetase | -1.0289 | 0.54634 | 4964.2 | 5140.7 | 5149.6 | 4681.4 |
| MARCKSL1 | MARCKS-like 1 | -1.1021 | 0.13796 | 3256.5 | 3808.8 | 3184.9 | 3206.3 |
| FOXA1 | forkhead box A1 | 1.0322 | 0.6747 | 10206 | 10066 | 10724 | 10206 |
| UGDH | UDP-glucose dehydrogenase | 1.5949 | 7.66E-06 | 979.25 | 949.38 | 1441.1 | 1641 |
| RETSAT | retinol saturase (all-trans-retinol 13.14-reductase) | -1.3476 | 0.002913 | 473.71 | 441.64 | 356.47 | 323.17 |
| AR | androgen receptor (dihydrotestosterone receptor; testicular feminization; spinal and bulbar muscular atrophy; Kennedy disease) | -1.0986 | 0.076078 | 647.68 | 715.79 | 597.19 | 643.22 |
| SEPP1 | selenoprotein P. plasma. 1 | -1.6145 | 3.93E-05 | 554.12 | 638.27 | 353.47 | 383.85 |
| NFIX | nuclear factor I/X (CCAAT-binding transcription factor) | -1.1033 | 0.15696 | 3480.9 | 3736.7 | 2989.2 | 3574.3 |
| GATA2 | GATA binding protein 2 | -1.3227 | 0.00026 | 620.39 | 666.15 | 480.03 | 492.12 |
| RBPJ | recombination signal binding protein for immunoglobulin kappa J region | -1.4895 | 1.64E-06 | 397.02 | 343.66 | 251.79 | 244.26 |
| MT1A | metallothionein 1A | 1.7761 | 7.92E-06 | 3615.8 | 2976.5 | 5826.7 | 5826.7 |
| SELENBP1 | selenium binding protein 1 | -1.5263 | 0.000293 | 1562.6 | 1765.8 | 1041.8 | 1136.9 |
| PNPLA7 | patatin-like phospholipase domain containing 7 | 1.2506 | 0.000418 | 1246.7 | 1157.7 | 1484.4 | 1520.6 |
| BCL11B | B-cell CLL/lymphoma 11B (zinc finger protein) | 1.0427 | 0.32539 | 229.25 | 215.65 | 236.29 | 227.48 |
| LPIN1 | lipin 1 | -1.0789 | 0.37112 | 2753.7 | 1933.6 | 2044.6 | 2237.2 |
| MT2A | metallothionein 2A | 1.7344 | 1.43E-06 | 3947.5 | 3813.4 | 6438.7 | 7033.3 |
| KIAA1244 | KIAA1244 | 1.04 | 0.75441 | 630.15 | 844.35 | 719.83 | 799.42 |
| GP1BB | glycoprotein Ib (platelet). beta polypeptide | 1.0821 | 0.094683 | 1130.8 | 1142.1 | 1210.3 | 1249.4 |
| NME1 | non-metastatic cells 1. protein (NM23A) expressed in | -1.075 | 0.16953 | 8769.2 | 8321.8 | 8348.7 | 7563.8 |
| WARS | tryptophanyl-tRNA synthetase | -1.0168 | 0.85814 | 1455.6 | 1329 | 1420 | 1317.8 |
| ATP6V0E2 | ATPase. H+ transporting V0 subunit e2 | -1.1188 | 0.013567 | 6739.5 | 6981.7 | 6131.2 | 6131.2 |
| MME | membrane metallo-endopeptidase | -1.4611 | 7.65E-06 | 1698.2 | 1791.3 | 1220.4 | 1167.5 |
| F12 | coagulation factor XII (Hageman factor) | -1.1392 | 0.006877 | 2061.5 | 2048.3 | 1800.6 | 1807 |
| CAMK2B | calcium/calmodulin-dependent protein kinase (CaM kinase) II beta | -1.0513 | 0.1627 | 966.77 | 946.28 | 886.53 | 933.74 |
| IFRD2 | interferon-related developmental regulator 2 | -1.0996 | 0.044397 | 512.1 | 543.67 | 489.09 | 470.78 |
| UBE2H | ubiquitin-conjugating enzyme E2H (UBC8 homolog. yeast) | -1.1512 | 0.1386 | 739.71 | 860.45 | 624.87 | 768.64 |
| MPP6 | membrane protein. palmitoylated 6 (MAGUK p55 subfamily member 6) | -1.0432 | 0.21022 | 449.93 | 452.9 | 435.5 | 429.94 |
| KHDRBS3 | KH domain containing. RNA binding. signal transduction associated 3 | -1.2912 | 0.002346 | 645.26 | 769.1 | 560.3 | 531.27 |
| KLK3 | kallikrein-related peptidase 3 | -1.5853 | 7.89E-05 | 4254.8 | 4448.5 | 2890.5 | 2605.6 |
| GPT2 | glutamic pyruvate transaminase (alanine aminotransferase) 2 | -1.0705 | 0.20047 | 4938.2 | 4427.2 | 4421.1 | 4314.8 |
| PPP1R14B | protein phosphatase 1. regulatory (inhibitor) subunit 14B | 1.0044 | 0.93701 | 7958.2 | 8136.1 | 7984.8 | 8180.5 |
| AMHR2 | anti-Mullerian hormone receptor. type II | -1.2778 | 0.000122 | 319.84 | 314.69 | 248.96 | 247.58 |
| MFSD3 | major facilitator superfamily domain containing 3 | -1.1703 | 0.008524 | 4644.3 | 4544.1 | 3958.5 | 3892.4 |
| PAFAH1B3 | platelet-activating factor acetylhydrolase. isoform Ib. gamma subunit 29kDa | -1.1473 | 0.007502 | 514.54 | 532.37 | 448.4 | 464.06 |
| MYCBP2 | MYC binding protein 2 | 1.0001 | 0.99816 | 956.5 | 844.77 | 902.29 | 895.77 |
| LAMA3 | laminin. alpha 3 | -1.676 | 1.88E-05 | 1384.7 | 1168.9 | 798.44 | 721.7 |
| IMPA2 | inositol(myo)-1(or 4)-monophosphatase 2 | -1.083 | 0.15636 | 487.39 | 542.85 | 484.6 | 465.5 |
| PARP10 | poly (ADP-ribose) polymerase family. member 10 | -1.5041 | 0.000159 | 559.6 | 601.85 | 362.62 | 410.55 |
| SC65 | synaptonemal complex protein SC65 | -1.1243 | 0.038203 | 1136.9 | 1176.5 | 1015.6 | 1041.8 |
| CAPN5 | calpain 5 | -1.0148 | 0.67586 | 514.14 | 517.52 | 522.51 | 494.45 |
| CMBL | carboxymethylenebutenolidase homolog (Pseudomonas) | -1.0938 | 0.11038 | 2820.4 | 2719.1 | 2586.2 | 2478.4 |
| KRT80 | keratin 80 | -1.3024 | 4.61E-06 | 214.58 | 204.35 | 156.43 | 165.26 |
| RANBP1 | RAN binding protein 1 | -1.1574 | 0.004708 | 1389.9 | 1344.4 | 1179.1 | 1183 |
| LOC153364 | similar to metallo-beta-lactamase superfamily protein | 1.019 | 0.68851 | 346.19 | 364.48 | 367.98 | 356.06 |
| KLK4 | kallikrein-related peptidase 4 | -1.1678 | 0.01971 | 6995.1 | 6342.1 | 5351.5 | 6078.4 |
| SDCCAG3 | serologically defined colon cancer antigen 3 | -1.2104 | 0.000892 | 1312.9 | 1348.5 | 1087.8 | 1110.8 |
| C15orf52 | chromosome 15 open reading frame 52 | -1.5165 | 0.000164 | 715.45 | 575.69 | 430.3 | 416.22 |
| PAK4 | p21(CDKN1A)-activated kinase 4 | -1.2305 | 0.001139 | 1084.6 | 1271 | 930.61 | 978.38 |
| TACC1 | transforming. acidic coiled-coil containing protein 1 | -1.1448 | 0.00451 | 3897.7 | 3620.9 | 3290.9 | 3272 |
| STEAP1 | six transmembrane epithelial antigen of the prostate 1 | -1.1986 | 0.00407 | 7204.7 | 7257.6 | 6263.9 | 5810.2 |
| ST7 | suppression of tumorigenicity 7 | -1.1673 | 0.031072 | 1311.4 | 1622.2 | 1260.9 | 1238.1 |
| RPL29 | ribosomal protein L29 | -1.1443 | 0.023544 | 1106.2 | 1121.5 | 1011.8 | 936.38 |
| PEX10 | peroxisome biogenesis factor 10 | -1.4271 | 9.45E-06 | 3360.6 | 3396.2 | 2421 | 2314.9 |
| PTPLB | protein tyrosine phosphatase-like (proline instead of catalytic arginine). member b | -1.2632 | 0.008487 | 1431.9 | 1477.3 | 1110.3 | 1193.9 |
| NUAK1 | NUAK family. SNF1-like kinase. 1 | -1.2206 | 0.000192 | 365.24 | 348.62 | 308.4 | 277.11 |
| C9orf58 | chromosome 9 open reading frame 58 | -1.0753 | 0.21055 | 2481.5 | 2798.5 | 2361 | 2543.6 |
| MT1F | metallothionein 1F | 1.913 | 4.23E-09 | 737.17 | 716.88 | 1373 | 1408.5 |
| KIF22 | kinesin family member 22 | -1.1685 | 0.007397 | 369.82 | 424.28 | 336.01 | 342 |
| ADAMTS1 | ADAM metallopeptidase with thrombospondin type 1 motif. 1 | -1.1747 | 0.031123 | 1124.9 | 1136 | 942.28 | 982.65 |
| PSPH | phosphoserine phosphatase | -1.0861 | 0.27462 | 1229.8 | 1275.9 | 1240.3 | 1072.5 |
| COASY | Coenzyme A synthase | -1.3317 | 0.000298 | 2989.2 | 2678.2 | 2235.4 | 2019.5 |
| EFCAB4A | EF-hand calcium binding domain 4A | -1.1289 | 0.123 | 805.37 | 851.92 | 712.23 | 755.93 |
| IQGAP2 | IQ motif containing GTPase activating protein 2 | -1.2797 | 5.24E-05 | 280.45 | 273.47 | 221.08 | 211.83 |
| 9-Sep | septin 9 | -1.1333 | 0.033897 | 5836.3 | 5911.7 | 5132 | 5234.3 |
| GULP1 | GULP. engulfment adaptor PTB domain containing 1 | 1.0456 | 0.52556 | 742.77 | 702.93 | 816.56 | 699.08 |
| MAP2 | microtubule-associated protein 2 | -1.3796 | 2.21E-06 | 255.21 | 235.92 | 179.33 | 176.41 |
| IFI6 | interferon. alpha-inducible protein 6 | -1.5513 | 7.04E-06 | 2262.1 | 2050.1 | 1413.1 | 1363.6 |
| BEST1 | bestrophin 1 | -1.5099 | 8.81E-05 | 744.33 | 691.44 | 495.77 | 455.33 |
| AMACR | alpha-methylacyl-CoA racemase | -1.2335 | 0.00133 | 422.52 | 451.34 | 355.08 | 352.96 |
| SASH1 | SAM and SH3 domain containing 1 | -1.0703 | 0.22301 | 833.99 | 808.21 | 767.04 | 767.04 |
| C8orf55 | chromosome 8 open reading frame 55 | -1.3207 | 5.54E-05 | 6366.7 | 6553.1 | 4728.9 | 5057.9 |
| RFX5 | regulatory factor X. 5 (influences HLA class II expression) | -1.1537 | 0.02212 | 851.05 | 853.41 | 745.24 | 732.18 |
| SPIRE1 | spire homolog 1 (Drosophila) | 1.0767 | 0.33192 | 1703.7 | 1534.5 | 1783.3 | 1699.5 |
| SLC27A5 | solute carrier family 27 (fatty acid transporter). member 5 | -1.1699 | 0.006095 | 651.47 | 671.89 | 575.43 | 555.8 |
| TMSL3 | thymosin-like 3 | -1.5972 | 1.46E-07 | 1753.8 | 1706.8 | 1024.7 | 1145.1 |
| RAMP1 | receptor (G protein-coupled) activity modifying protein 1 | -1.2717 | 0.000576 | 1072 | 1001.8 | 832.91 | 797.26 |
| AK2 | adenylate kinase 2 | -1.267 | 0.023804 | 4784.2 | 5810.2 | 3777.2 | 4584.5 |
| ZNF30 | zinc finger protein 30 | -1.1881 | 0.002509 | 468.19 | 454.24 | 395.71 | 380.7 |
| TMPRSS2 | transmembrane protease. serine 2 | -1.0661 | 0.071291 | 3853.5 | 4166.1 | 3798 | 3719 |
| RTN1 | reticulon 1 | -1.9235 | 1.96E-09 | 455.08 | 456.24 | 244.9 | 229.15 |
| CYP11A1 | cytochrome P450. family 11. subfamily A. polypeptide 1 | -1.496 | 9.26E-06 | 230.65 | 197.48 | 143.41 | 141.93 |
| PPAP2A | phosphatidic acid phosphatase type 2A | -1.5548 | 2.00E-08 | 273.29 | 290.12 | 187.38 | 175.05 |
| SQSTM1 | sequestosome 1 | 1.323 | 6.16E-05 | 3319.6 | 3002.4 | 4365.4 | 3996.1 |
| ALDH1A3 | aldehyde dehydrogenase 1 family. member A3 | -1.3621 | 0.000224 | 599.55 | 590.85 | 459.37 | 415.63 |
| C1orf93 | chromosome 1 open reading frame 93 | -1.2415 | 4.24E-05 | 686.7 | 713.76 | 567.56 | 560.3 |
| STMN3 | stathmin-like 3 | -1.2284 | 2.88E-05 | 389.01 | 407.52 | 317.9 | 330.51 |
| OVGP1 | oviductal glycoprotein 1. 120kDa (mucin 9. oviductin) | -1.6464 | 5.79E-09 | 462.95 | 443.23 | 283.53 | 266.99 |
| KIAA0746 | KIAA0746 protein | 1.1802 | 0.010174 | 3116 | 3018.3 | 3785.8 | 3460.6 |
| CASP4 | caspase 4. apoptosis-related cysteine peptidase | -1.0922 | 0.022443 | 1063.3 | 1092.9 | 970.16 | 1004.1 |
| BAMBI | BMP and activin membrane-bound inhibitor homolog (Xenopus laevis) | -1.1398 | 0.000803 | 1410.9 | 1392.3 | 1216.1 | 1243.4 |
| TAX1BP3 | Tax1 (human T-cell leukemia virus type I) binding protein 3 | -1.6225 | 1.13E-05 | 1306.6 | 1405.8 | 808.21 | 863.32 |
| ABHD7 | abhydrolase domain containing 7 | -1.4039 | 8.46E-06 | 273.72 | 274.35 | 182.11 | 209.24 |
| IDH1 | isocitrate dehydrogenase 1 (NADP+). soluble | -1.0084 | 0.85379 | 1915.5 | 1979.5 | 2014 | 1851.5 |
| ABLIM1 | actin binding LIM protein 1 | -1.1823 | 0.021434 | 1052.4 | 1076.9 | 922.42 | 879.03 |
| SLC25A10 | solute carrier family 25 (mitochondrial carrier; dicarboxylate transporter). member 10 | -1.2702 | 0.021929 | 1436.1 | 1474.1 | 1063.3 | 1233.9 |
| H2AFJ | H2A histone family. member J | -1.359 | 0.000648 | 3200.3 | 3364.7 | 2613.1 | 2231.3 |
| NQO1 | NAD(P)H dehydrogenase. quinone 1 | 1.5334 | 1.97E-06 | 2802.6 | 2747.7 | 4536.8 | 3991.2 |
| LOC493869 | similar to RIKEN cDNA 2310016C16 | -1.5934 | 5.05E-05 | 330.98 | 334.41 | 200.89 | 216.99 |
| SPECC1L | SPECC1-like | -1.2887 | 0.001893 | 4897.6 | 5077.7 | 3881.3 | 3857.8 |
| ATP6V1E2 | ATPase. H+ transporting. lysosomal 31kDa. V1 subunit E2 | -1.504 | 2.32E-08 | 650.94 | 641.98 | 433.51 | 426.15 |
| AADAT | aminoadipate aminotransferase | -1.0887 | 0.32475 | 977.39 | 849.65 | 884.86 | 791.84 |
| VIM | vimentin | -1.5114 | 2.75E-06 | 314.22 | 318.73 | 199.53 | 219.74 |
| PRPS1 | phosphoribosyl pyrophosphate synthetase 1 | -1.1893 | 0.008536 | 2002.8 | 2038.2 | 1619.5 | 1782.2 |
| TXNRD1 | thioredoxin reductase 1 | 1.6772 | 6.42E-06 | 2436.9 | 2458.3 | 4286.6 | 3931.4 |
| TMEM2 | transmembrane protein 2 | -1.1378 | 0.063489 | 776.77 | 675.48 | 666.52 | 608.06 |
| HSPB8 | heat shock 22kDa protein 8 | -1.4547 | 0.001155 | 248.73 | 262.83 | 170.63 | 181.04 |
| AMD1 | adenosylmethionine decarboxylase 1 | -1.1451 | 0.08015 | 2186.8 | 2039.1 | 1910 | 1780.5 |
| CAB39L | calcium binding protein 39-like | -1.4019 | 7.11E-05 | 1359.7 | 1548.1 | 1008.1 | 1062.3 |
| BANK1 | B-cell scaffold protein with ankyrin repeats 1 | -1.2849 | 0.000176 | 719.83 | 736.72 | 545.31 | 589.02 |
| PANX2 | pannexin 2 | 1.177 | 0.008021 | 572.4 | 581.06 | 654.99 | 703.45 |
| PPIC | peptidylprolyl isomerase C (cyclophilin C) | -1.6083 | 8.14E-06 | 772.54 | 702.27 | 479.53 | 437.42 |
| UGT2B10 | UDP glucuronosyltransferase 2 family. polypeptide B10 | -1.5393 | 4.73E-07 | 201.26 | 220.93 | 135.42 | 138.57 |
| TMEFF2 | transmembrane protein with EGF-like and two follistatin-like domains 2 | -1.2405 | 0.000252 | 1252.7 | 1341.4 | 1080.9 | 1010.3 |
| ST6GALNAC1 | ST6 (alpha-N-acetyl-neuraminyl-2.3-beta-galactosyl-1.3)-N-acetylgalactosaminide alpha-2.6-sialyltransferase 1 | 1.0039 | 0.90863 | 398.24 | 388.09 | 382.44 | 407.28 |
| COL4A5 | collagen. type IV. alpha 5 (Alport syndrome) | -1.3181 | 0.00079 | 1527.9 | 1316.5 | 1072.5 | 1079.4 |
| COL16A1 | collagen. type XVI. alpha 1 | -1.1023 | 0.13902 | 469.07 | 412.13 | 419.52 | 379.23 |
| KCNN2 | potassium intermediate/small conductance calcium-activated channel. subfamily N. member 2 | -1.3015 | 0.000151 | 1669.4 | 1583 | 1294.1 | 1205.5 |
| ZDHHC14 | zinc finger. DHHC-type containing 14 | -1.8143 | 1.99E-08 | 948.44 | 1013.5 | 543.67 | 537.11 |
| GLRX | glutaredoxin (thioltransferase) | -1.6257 | 4.31E-07 | 353.47 | 329.33 | 212.75 | 207.02 |
| TMEM158 | transmembrane protein 158 | -1.4702 | 4.82E-09 | 385.17 | 406.11 | 269.46 | 268.56 |
| MESP1 | mesoderm posterior 1 homolog (mouse) | -1.2329 | 0.000845 | 1676.3 | 1764.6 | 1358.1 | 1432.9 |
| HOXC6 | homeobox C6 | -1.3046 | 0.00031 | 2095.1 | 1840.2 | 1513.1 | 1497.2 |
| H2BFS | H2B histone family. member S | -1.2778 | 0.001408 | 1415.2 | 1288.3 | 1140.3 | 979.25 |
| LPXN | leupaxin | -1.3039 | 4.77E-05 | 525.86 | 464.06 | 383.72 | 374.05 |
| RP11-298P3.3 | CG016 | -1.1864 | 0.017314 | 1235.7 | 1015.6 | 959.63 | 929.08 |
| MAF | v-maf musculoaponeurotic fibrosarcoma oncogene homolog (avian) | -1.3778 | 4.79E-07 | 253.82 | 245.95 | 186.3 | 176.53 |
| CYP39A1 | cytochrome P450. family 39. subfamily A. polypeptide 1 | -1.3347 | 9.77E-06 | 243.82 | 235.48 | 186.91 | 172.43 |
| PPFIBP2 | PTPRF interacting protein. binding protein 2 (liprin beta 2) | -1.5452 | 6.17E-07 | 1256 | 1217.5 | 784.35 | 816.56 |
| TP53INP1 | tumor protein p53 inducible nuclear protein 1 | -1.2366 | 0.000439 | 1610.4 | 1523.5 | 1254.3 | 1279.2 |
| ENC1 | ectodermal-neural cortex (with BTB-like domain) | -1.2956 | 0.000196 | 544.26 | 491.33 | 383.85 | 415.05 |
| MRPL27 | mitochondrial ribosomal protein L27 | -1.2591 | 0.008955 | 1478.9 | 1572.2 | 1233.2 | 1189.3 |
| HIST2H2BE | histone cluster 2. H2be | -1.9032 | 6.34E-10 | 1681.2 | 1644.9 | 880.16 | 867.46 |
| PHOSPHO2 | phosphatase. orphan 2 | -1.5204 | 4.89E-09 | 542.85 | 514.78 | 347.65 | 347.74 |
| SPATA20 | spermatogenesis associated 20 | -1.3996 | 0.000239 | 2955.8 | 3084.3 | 2120.7 | 2194.4 |
| RPS15 | ribosomal protein S15 | -1.4745 | 0.001271 | 822.45 | 991.86 | 607.45 | 617.68 |
| CLDN8 | claudin 8 | -1.3258 | 1.09E-05 | 412.49 | 423.8 | 316.2 | 314.5 |
| P2RY11 | purinergic receptor P2Y. G-protein coupled. 11 | -1.7222 | 9.06E-08 | 428.06 | 428.95 | 253.19 | 244.52 |
| TP53AP1 | TP53 activated protein 1 | -1.5111 | 1.54E-05 | 632.9 | 622.41 | 438.73 | 393.2 |
| HIST1H2BD | histone cluster 1. H2bd | -1.6799 | 3.41E-08 | 516.18 | 468.93 | 295.25 | 290.51 |
| C1orf144 | chromosome 1 open reading frame 144 | -1.5965 | 6.22E-05 | 1096.2 | 1113.1 | 682.58 | 701.31 |
| DNASE2B | deoxyribonuclease II beta | -1.3758 | 3.20E-06 | 1189.7 | 1205.5 | 877.34 | 863.61 |
| ID1 | inhibitor of DNA binding 1. dominant negative helix-loop-helix protein | -1.6483 | 6.68E-09 | 237.59 | 229.91 | 143.42 | 140.19 |
| MB | myoglobin | -1.3665 | 1.51E-05 | 550.55 | 566.53 | 419.94 | 397.72 |
| SLC39A8 | solute carrier family 39 (zinc transporter). member 8 | -1.5851 | 1.28E-06 | 987.38 | 1067.9 | 651.74 | 643.92 |
| SERPINE2 | serpin peptidase inhibitor. clade E (nexin. plasminogen activator inhibitor type 1). member 2 | -1.7188 | 4.50E-06 | 511.53 | 400.04 | 262.96 | 263.4 |
| ZC3HAV1 | zinc finger CCCH-type. antiviral 1 | -2.3727 | 3.17E-10 | 2164.9 | 2183.3 | 871.09 | 963.86 |
| LCN2 | lipocalin 2 (oncogene 24p3) | -1.5355 | 1.58E-05 | 359.2 | 366.97 | 232.91 | 240.02 |
| TMEPAI | transmembrane. prostate androgen induced RNA | -1.4519 | 2.85E-06 | 1323.8 | 1382.5 | 928.63 | 934.96 |
| PKIB | protein kinase (cAMP-dependent. catalytic) inhibitor beta | -1.5899 | 3.20E-05 | 451.09 | 488.68 | 314.5 | 277.28 |
| ALDH3A2 | aldehyde dehydrogenase 3 family. member A2 | -1.1993 | 0.000206 | 3913.2 | 3936 | 3346 | 3200.3 |
| TMEM45B | transmembrane protein 45B | -1.6071 | 3.27E-06 | 601.85 | 602.57 | 367.41 | 382.16 |
| ANKRD25 | ankyrin repeat domain 25 | -1.5878 | 1.05E-08 | 412 | 408.93 | 265.14 | 252.05 |
| SH3BGRL | SH3 domain binding glutamic acid-rich protein like | -1.6773 | 2.24E-06 | 713.22 | 653.08 | 385.67 | 429.31 |
| NKX3-1 | NK3 homeobox 1 | -1.3214 | 0.066019 | 3290.9 | 4005.9 | 2425.7 | 3112.7 |
| GBP2 | guanylate binding protein 2. interferon-inducible | -2.0677 | 1.00E-07 | 470.67 | 399.49 | 211.41 | 208.02 |
| IFI35 | interferon-induced protein 35 | -1.3955 | 7.01E-05 | 714.05 | 773.02 | 521.38 | 543.67 |
| ISG15 | ISG15 ubiquitin-like modifier | -1.5907 | 4.10E-06 | 2328.1 | 2351.9 | 1526.6 | 1417.5 |
| CDT1 | chromatin licensing and DNA replication factor 1 | -1.803 | 7.86E-10 | 617.29 | 590.05 | 338.81 | 330.71 |
| GUCY1A3 | guanylate cyclase 1. soluble. alpha 3 | -1.4808 | 1.17E-07 | 1737.2 | 1691.8 | 1163.1 | 1152.4 |
| UGT2B28 | UDP glucuronosyltransferase 2 family. polypeptide B28 | -2.4805 | 3.01E-11 | 422.4 | 433.09 | 168.51 | 176.43 |
| HIST2H2AC | histone cluster 2. H2ac | -1.973 | 1.08E-07 | 2116.8 | 2111.9 | 1062.8 | 1080.6 |
| HIST1H3H | histone cluster 1. H3h | -1.6909 | 6.76E-07 | 475.26 | 402.03 | 263.98 | 253.16 |
| AK1 | adenylate kinase 1 | -1.725 | 8.98E-07 | 880.16 | 865.72 | 498.72 | 513.48 |
| GPNMB | glycoprotein (transmembrane) nmb | -1.6245 | 1.02E-08 | 391.52 | 389.77 | 240.67 | 240.25 |
| ID3 | inhibitor of DNA binding 3. dominant negative helix-loop-helix protein | -1.6593 | 5.08E-07 | 303.7 | 271.7 | 178.67 | 167.73 |
| PPFIA2 | protein tyrosine phosphatase. receptor type. f polypeptide (PTPRF). interacting protein (liprin). alpha 2 | -1.6842 | 1.19E-07 | 682 | 729.37 | 434.35 | 403.73 |
| HIST1H2AC | histone cluster 1. H2ac | -1.7899 | 2.48E-07 | 662.56 | 589.35 | 350.83 | 347.41 |
| C1orf116 | chromosome 1 open reading frame 116 | -1.1494 | 0.01256 | 3503.8 | 3387.6 | 3077 | 2919.8 |
| HIST2H2AA3 | histone cluster 2. H2aa3 | -2.0146 | 6.95E-08 | 1294.1 | 1390.7 | 679.28 | 652.75 |
| NOS3 | nitric oxide synthase 3 (endothelial cell) | -1.8453 | 2.08E-06 | 505.47 | 476.04 | 261.53 | 270.2 |
| HIST1H4H | histone cluster 1. H4h | -1.8704 | 1.17E-07 | 1279.2 | 1181.4 | 660.67 | 653.81 |
| HIST1H1C | histone cluster 1. H1c | -2.347 | 9.30E-09 | 4094.5 | 3785.8 | 1742.9 | 1614.6 |
| TRPV6 | transient receptor potential cation channel. subfamily V. member 6 | -1.541 | 2.29E-07 | 1039.9 | 961.17 | 638.07 | 659.7 |
| VCX3B | variable charge. X-linked 3B | -2.3529 | 4.27E-09 | 667.64 | 668.77 | 282.98 | 285.01 |
| VCY | variable charge. Y-linked | -2.3021 | 1.10E-08 | 645.96 | 694.23 | 297.69 | 284.25 |
| VCX2 | variable charge. X-linked 2 | -2.4635 | 3.69E-10 | 774.84 | 767.34 | 313.53 | 312.48 |
| UGT2B11 | UDP glucuronosyltransferase 2 family. polypeptide B11 | -2.8692 | 1.47E-06 | 825.72 | 993.08 | 282.52 | 352.57 |
| UGT2B17 | UDP glucuronosyltransferase 2 family. polypeptide B17 | -2.5504 | 2.25E-10 | 1211.6 | 1364.9 | 476.27 | 533.82 |
| VCX | variable charge. X-linked | -2.7182 | 1.38E-08 | 2647.7 | 2578.2 | 980.45 | 942.28 |
| UGT2B7 | UDP glucuronosyltransferase 2 family. polypeptide B7 | -3.2165 | 6.09E-12 | 3574.3 | 3583.3 | 1053 | 1175.7 |
| TXNIP | thioredoxin interacting protein | -36.396 | 9.16E-15 | 10390 | 10123 | 307.79 | 257.95 |
